# Copper catalyzed borocarbonylation of benzylidenecyclopropanes through selective proximal C–C bond cleavage: synthesis of γ-boryl-γ,δ-unsaturated carbonyl compounds†

**DOI:** 10.1039/d2sc01992b

**Published:** 2022-05-30

**Authors:** Li-Miao Yang, Hui-Hui Zeng, Xin-Lian Liu, Ai-Jun Ma, Jin-Bao Peng

**Affiliations:** School of Biotechnology and Health Sciences, Wuyi University Jiangmen Guangdong 529020 People's Republic of China pengjb_05@126.com

## Abstract

A copper catalyzed borocarbonylation of BCPs *via* proximal C–C bond cleavage for the synthesis of γ-boryl-γ,δ-unsaturated carbonyl compounds has been developed. Using substituted benzylidenecyclopropanes (BCPs) and chloroformates as starting material, a broad range of γ-boryl-γ,δ-unsaturated esters were prepared in moderate to excellent yields with excellent regio- and stereoselectivity. Besides, when aliphatic acid chlorides were used in this reaction, γ-boryl-γ,δ-unsaturated ketones could be produced in excellent yields. When substituted BCPs were used as substrates, the borocarbonylation occurred predominantly at the proximal C–C bond *trans* to the phenyl group in a regio- and stereoselective manner, which leads to the *Z*-isomers as the products. This efficient methodology involves the cleavage of a C–C bond and the formation of a C–C bond as well as a C–B bond, and provides a new method for the proximal C–C bond difunctionalization of BCPs.

## Abstract 2

A copper catalyzed borocarbonylation of benzylidenecyclopropanes (BCPs) *via* proximal C–C bond cleavage for the synthesis of γ-boryl-γ,δ-unsaturated carbonyl compounds has been developed.

## Graphical abstract

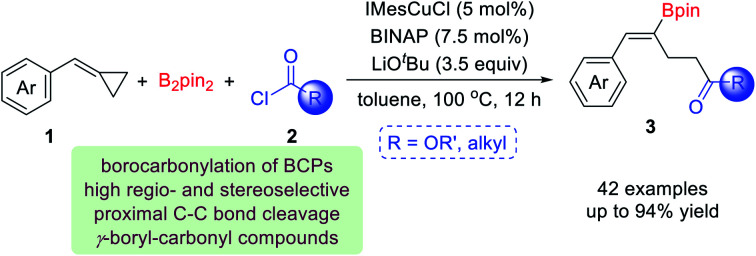

## Introduction

**Scheme 1:**
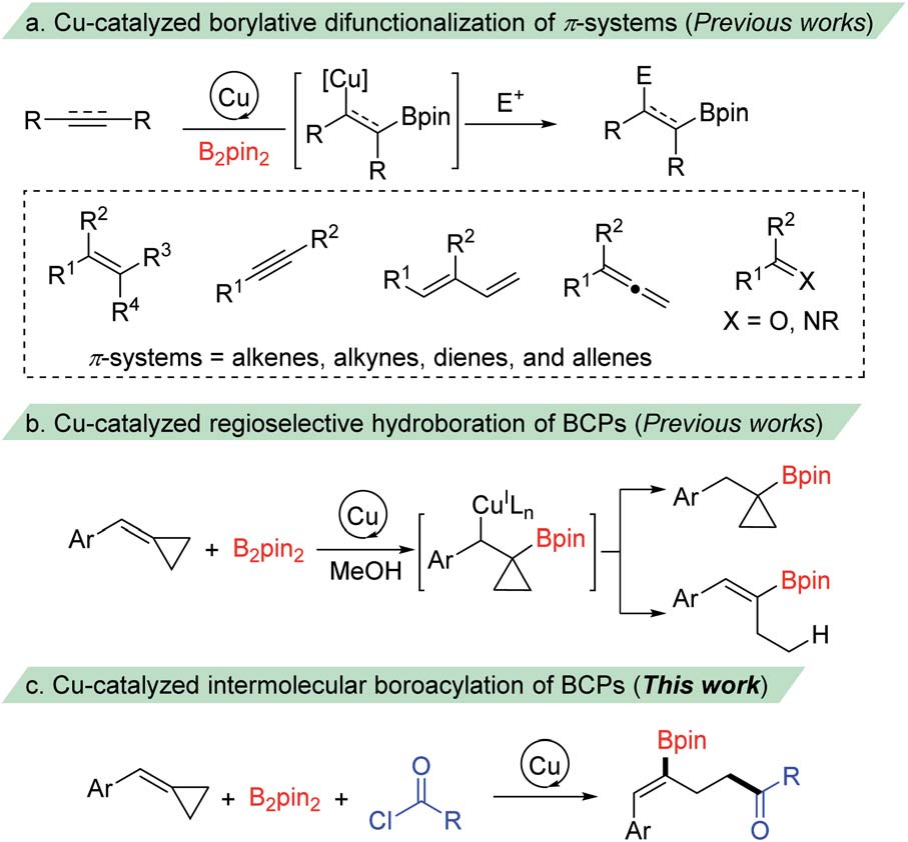
Difunctionalization of π-systems and BCPs.

Organoboron compounds have emerged as versatile building blocks in organic synthesis owing to their unique reactivity and divergent synthetic applications.^1^ They have also found wide utility in materials science^2^ and medicinal chemistry.^3^ Thus, continued research efforts have been made for their synthesis,^4^ while the most prevalent synthetic route for their preparation proceeds *via* metal-catalyzed borylation of organohalides, hydroboration of alkenes, and borylation of C–H, C–Het and C–C bonds. Recently, impressive progress has been made in copper catalyzed borylative difunctionalization of π-systems, which provides a promising strategy for rapid generation of molecular complexity to install a boron and unique groups across π-systems.^5^ These processes are particularly valuable since the resulting C–B bond could be transformed into various functional groups *via* C–C and C–heteroatom bond-forming reactions through stereospecific transformations.^6^ The key step in these reactions is the addition of Cu–Bpin species to C–C π-bonds to give the corresponding β-borylalkylcopper intermediates, which could be captured by various electrophiles to give the difunctionalized products (Scheme 1a). A range of π-systems including alkenes, allenes, dienes, and alkynes as well as some carbon–heteroatom systems such as ketones, aldehydes and imines have been examined and found to react with a copper–boryl species. These reactions employed boro-metalation of π-bonds to give 1,2- or 1,4-difunctionalized products. However, borylative difunctionalization *via* C–C σ-bond cleavage has rarely been reported.^7^

The introduction of strained small rings, which can make σ-bonds behave like π-bonds, as molecular building blocks has recently emerged as a powerful tool in organic synthesis.^8^ Among them, methylenecyclopropanes (MCPs) and benzylidenecyclopropanes (BCPs), containing a highly strained cyclopropane ring with an exo-methylene group, are valuable building blocks in organic synthesis owing to their unique structure and high reactivity.^9^ The reactions of MCPs and BCPs under transition metal catalysis have been extensively explored in the past few decades, and have been frequently used in a range of reactions, such as cycloaddition reactions, cycloisomerizations and ring-opening reactions.^10^ The cleavage of a proximal or a distal C–C single bond of the cyclopropane ring accompanied by the addition of an E^1^–E^2^ bond (such as X–H,^11*a*–*j*^ B–B,^11*k*,*l*^ B–Si^11*m*–*r*^ and other σ bonds^11*s*,*t*^) to give 1,3-difunctionalized products has also been investigated. Recently, McAlpine, Liu and Engle reported an elegant copper-catalyzed hydroboration of BCPs with B_2_pin_2_ and MeOH. The benzylcopper intermediate, formed by the addition of Cu–Bpin species to the C–C double bond, underwent a β-carbon elimination/protodecupration process and provided ring-opening hydroboration product alkenylboronates selectively through the choice of a suitable phosphine ligand (Scheme 1b).^11*j*^ However, to the best of our knowledge, the capture of the Cu–C bond from such β-carbon elimination of BCPs with a carbon electrophile other than a proton has been less reported.^12^ Herein, we report a copper catalyzed borocarbonylation^13^ of BCPs with B_2_pin_2_ and chloroformates (or acyl chlorides) through the cleavage of a proximal C–C bond of the cyclopropane ring for the synthesis of γ-boryl-γ,δ-unsaturated carbonyl compounds (Scheme 1c).^14^ Notable features of our study include: (1) regio- and stereoselective boroacylation of BCPs through selective proximal C–C bond cleavage, (2) preparation of 1,3-difunctionalized products with γ-boryl-γ,δ-unsaturated carbonyl compounds, and (3) broad substrate scopes as well as good functional group tolerance.

## Results and discussion

Initially, 2-(cyclopropylidenemethyl)naphthalene 1a and phenyl chloroformate 2a were selected as model substrates to react with B_2_pin_2_ to evaluate the feasibility of the borocarbonylation reaction. To our delight, using IMesCuCl as a catalyst, PPh_3_ as the ligand and LiO^*t*^Bu as a base, when a solution of 1a, B_2_pin_2_ and 2a in toluene was stirred at 90 °C for 12 h, the desired borocarbonylation product 3aa was successfully obtained in 58% yield (Table 1, entry 1). This reaction turned out to be highly regio- and stereoselective, and only the *Z*-isomer was obtained. Then, we examined a range of copper catalysts for this reaction. When the precatalyst was substituted with IPrCuCl, a much reduced conversion was observed and a decreased yield of 26% was obtained (Table 1, entry 2). No reaction was observed when CuBr was used (Table 1, entry 3); other copper catalysts such as CuTc and Cu(CH_3_CN)_4_PF_6_ lead to the formation of the hydroboration products (see details in the ESI†).^11*j*^ The phosphine ligands were reported to be able to affect the β-carbon elimination step.^11*j*^ Thus, we then screened a series of ligands. Indeed, a phosphine or a pyridyl ligand is essential for this reaction. Only a trace amount of product 3aa was observed in the absence of ligand (Table 1, entry 4). The yield of 3aa was improved to 67% when BINAP was used (Table 1, entry 5). Other ligands such as PCy_3_, BuPAd_2_ and dtbbpy were also effective and produced the desired product 3aa (Table 1, entry 6, see details in the ESI†). The ratio of the ligand to the catalyst also played an important role in this reaction. Decreased yield was obtained when the reaction was performed at a lower ligand/Cu ratio (Table 1, entry 7). A ligand/Cu ratio of 1.5 : 1 was found to be optimal and produced 3aa in 69% yield (Table 1, entry 8). Then, a series of bases were tested and it was found that alkali metal alkoxides including KO^*t*^Bu and NaOMe were effective and provided the desired product 3aa in 58% and 42% yields, respectively (Table 1, entries 9 and 10). The bisborylated product of 1a and small amounts of hydroborylation products were observed as the by-products in these reactions. However, when NaO^*t*^Bu was used as a base, no desired product 3aa was obtained, only the bisborylated product was observed.^11*k*^ Other bases such as K_2_CO_3_, Na_2_CO_3_ and Et_3_N were ineffective and no reaction was observed (see details in the ESI†). The yield of 3aa could be further improved to 89% when 3.5 equivalents of phenyl chloroformate 2a were used (Table 1, entry 11, see details in the ESI†). Screening of the solvent revealed that toluene is the optimal solvent. When PhCl and MeCN were used as the solvents, the yields decreased to 76% and 43%, respectively (Table 1, entries 12 and 13). Finally, the reaction temperature affected the efficiency of this reaction as well, and an excellent yield of 91% was obtained when the reaction was performed at 100 °C (Table 1, entry 14).

**Table 1:** Optimization of the reaction conditions^a^

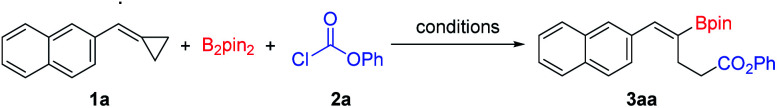					
Entry	Cu	Ligand	Base	Solvent	Yield^b^ (%)
1	IMesCuCl	PPh_3_	LiO^*t*^Bu	Toluene	58
2	IPrCuCl	PPh_3_	LiO^*t*^Bu	Toluene	26
3	CuBr	PPh_3_	LiO^*t*^Bu	Toluene	No reaction
4	IMesCuCl	—	LiO^*t*^Bu	Toluene	Trace
5	IMesCuCl	BINAP	LiO^*t*^Bu	Toluene	67
6	IMesCuCl	PCy_3_	LiO^*t*^Bu	Toluene	29
7^c^	IMesCuCl	BINAP	LiO^*t*^Bu	Toluene	57
8^d^	IMesCuCl	BINAP	LiO^*t*^Bu	Toluene	69
9^d^	IMesCuCl	BINAP	KO^*t*^Bu	Toluene	58
10^d^	IMesCuCl	BINAP	NaOMe	Toluene	42
11^d^^,^^e^	IMesCuCl	BINAP	LiO^*t*^Bu	Toluene	89
12^d^^,^^e^	IMesCuCl	BINAP	LiO^*t*^Bu	PhCl	76
13^d^^,^^e^	IMesCuCl	BINAP	LiO^*t*^Bu	MeCN	43
**14** d ^,^ e ^,^ f	**IMesCuCl**	**BINAP**	**LiO** ^***t***^ **Bu**	**Toluene**	**91**

a Reaction conditions: 1a (0.1 mmol), B_2_pin_2_ (0.15 mmol), 2a (0.15 mmol), catalyst (5 mol%), ligand (10 mol% for monodentate ligand, 5 mol% for bidentate ligand), base (1.5 equiv.), solvent (1 mL), 90 °C, 12 h.

b Isolated yields.

c BINAP (2.5 mol%).

d BINAP (7.5 mol%).

e 2a (0.35 mmol).

f 100 °C.

**Scheme 2:**
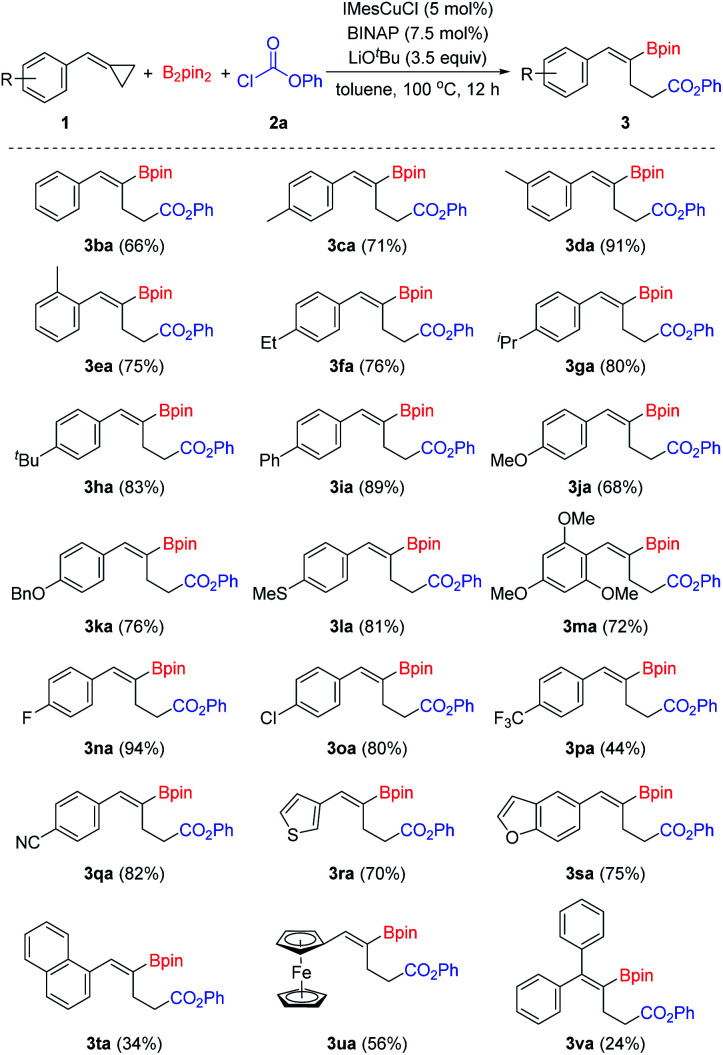
Substrate scope of the BCPs. Reaction conditions: 1 (0.1 mmol), B_2_pin_2_ (0.15 mmol), 2a (0.35 mmol), IMesCuCl (5 mol%), BINAP (7.5 mol%), LiO^*t*^Bu (3.5 equiv.), toluene (1 mL), 100 °C, 12 h, isolated yields.

With the optimized reaction conditions in hand (Table 1, entry 14), we began to investigate the substrate scope of this borocarbonylation reaction with emphasis being placed first on BCPs (Scheme 2). A wide range of substituted BCPs were tested and delivered the corresponding products in moderate to excellent yields with excellent regio- and stereoselectivities. When substituted BCPs were used as substrates, the borocarbonylation occurred selectively at the proximal C–C bond *trans* to the aryl group, leading to the formation of *Z*-isomers selectively. Only *Z*-isomers were obtained in these reactions. Both electron-donating (3ca–3ma) and electron-withdrawing groups (3na–3qa) were tolerated. Functional groups such as ether (3ja, 3ka and 3ma), thioether (3la), fluoro- (3na), chloro- (3oa) and cyano (3qa) groups were compatible in this reaction. In addition, heteroaryl-substituted BCPs were tolerated as well. For example, 3-thiophenyl and 5-benzofuranyl substituted substrates reacted successfully with B_2_pin_2_ and phenyl chloroformate 2a and provided the corresponding products 3ra and 3sa in 70% and 75% yields, respectively. When a 1-naphthyl substituted substrate was used in this reaction, the corresponding product 3ta was produced in 34% yield. Moreover, ferrocene can be tolerated as well, and the corresponding product 3ua was isolated in 57% yield. However, when diphenyl substituted methylenecyclopropane was used in this reaction, the desired product 3va was obtained in a low yield of 24%, which might have resulted from the steric effect.

**Scheme 3:**
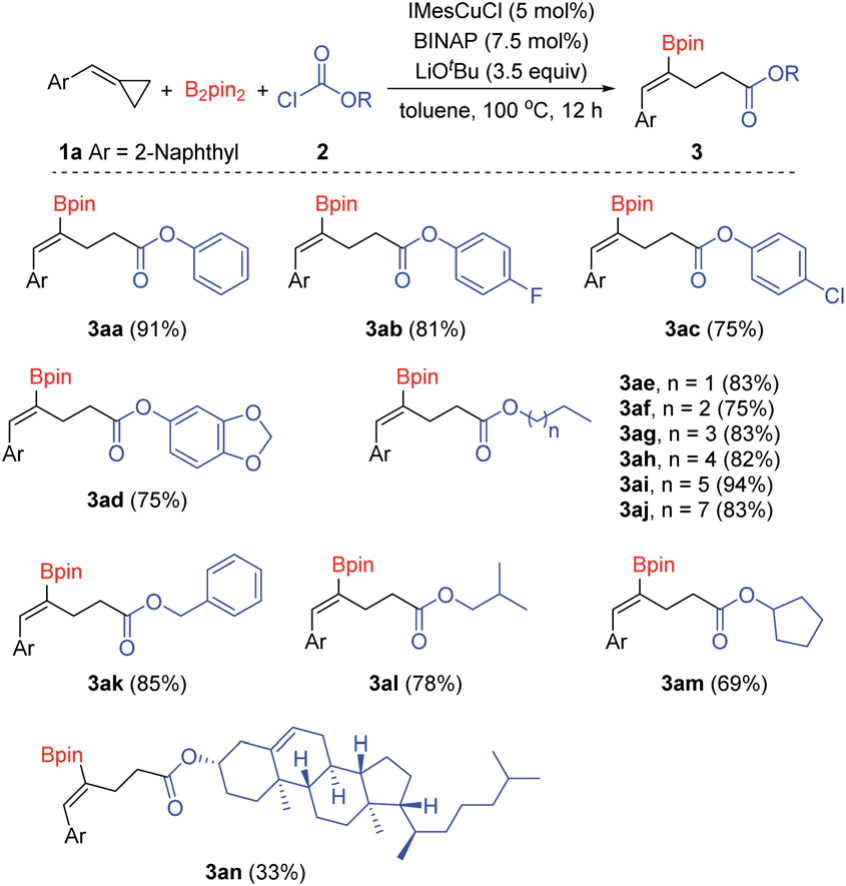
Substrate scope of chloroformates. Reaction conditions: 1a (0.1 mmol), B_2_pin_2_ (0.15 mmol), 2 (0.35 mmol), IMesCuCl (5 mol%), BINAP (7.5 mol%), LiO^*t*^Bu (3.5 equiv.), toluene (1 mL), 100 °C, 12 h, isolated yields.

Then, we began to explore the substrate scope of this borocarbonylation reaction with a series of chloroformates. As shown in Scheme 3, phenyl chloroformates with substitution of either electron-withdrawing (3ab and 3ac) or electron-donating groups (3ad) on the phenyl ring reacted smoothly to provide the corresponding products in good yields. Alkyl chloroformates were also suitable substrates: primary and secondary alkyl chloroformates reacted smoothly and afforded the corresponding γ-boryl-γ,δ-unsaturated esters in good to excellent yields (3ae–3am). Moreover, when chloroformate 2n derived from natural cholesterol was treated with B_2_pin_2_ and 1a under the standard conditions, the target product 3an was obtained in 33% yield. However, when *tert*-butyl chloroformate was used in this reaction, no desired borocarbonylation product was obtained, only the bisborylated product was observed as the main by-product.^11*k*^

**Scheme 4:**
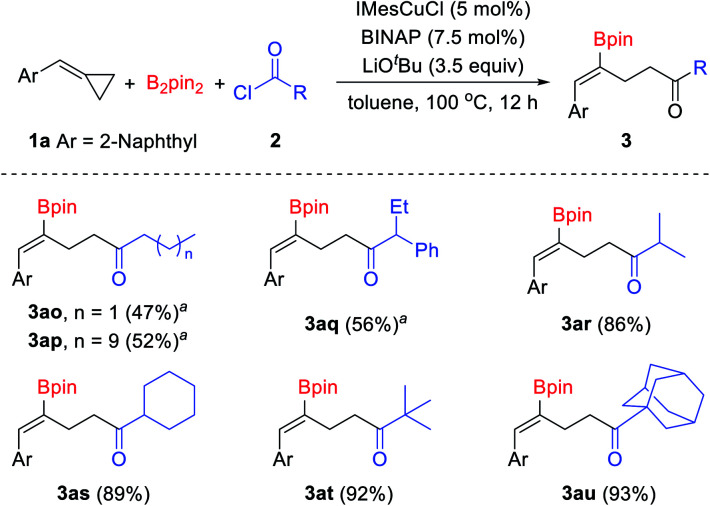
Substrate scope of acid chlorides. Reaction conditions: 1a (0.1 mmol), B_2_pin_2_ (0.15 mmol), 2 (0.35 mmol), IMesCuCl (5 mol%), BINAP (7.5 mol%), LiO^*t*^Bu (3.5 equiv.), toluene (1 mL), 100 °C, 12 h, isolated yields. ^*a*^60 °C, 24 h.

In addition to chloroformates, acid chlorides were also tested in this reaction (Scheme 4). Aliphatic acid chlorides were tolerated well in this reaction. Although low yields were obtained when primary alkanoyl chlorides were used under the standard conditions, the desired products could be obtained in acceptable yields at a lower reaction temperature.^15^ When *n*-butyryl chloride and *n*-dodecanoyl chloride were used in this reaction, the desired products 3ao and 3ap were obtained in 47% and 52% yields, respectively. Secondary and tertiary alkanoyl chlorides showed good reactivity and provided the desired products in good to excellent yields. For instance, iso-butyryl chloride, cyclohexanecarbonyl chloride, pivaloyl chloride and 1-adamantanecarbonyl chloride reacted with B_2_pin_2_ and 2-(cyclopropylidenemethyl)naphthalene 1a under the standard conditions and produced the corresponding γ-boryl-γ,δ-unsaturated ketones 3ar–3at in 86–93% yields. However, aromatic acid chlorides failed to give the desired products, inseparable mixtures of by-products were obtained when aroyl chlorides were used.

**Scheme 5:**
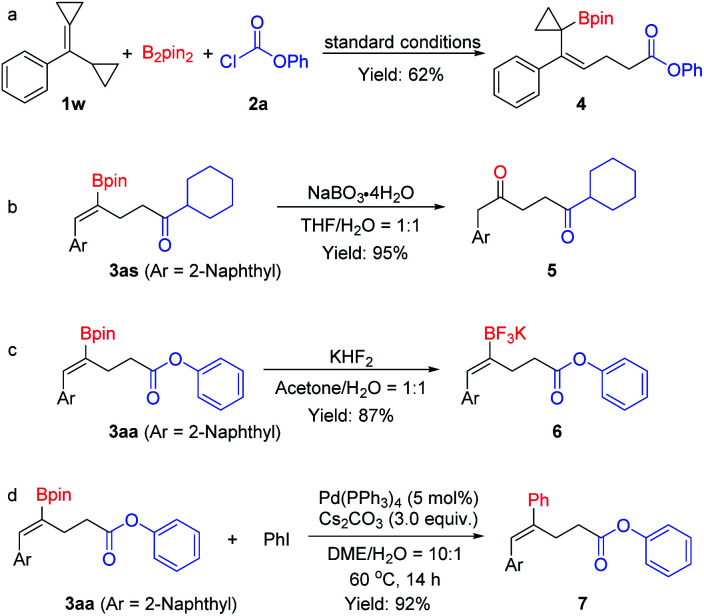
(a) Borocarbonylation of cyclopropyl substituted substrate. (b–d) Derivatization of the borocarbonylation products.

To understand the reaction pathway and to illustrate the synthetic versatility of the borocarbonylated products, the following reactions were conducted. First, when cyclopropyl substituted substrate 1w was treated with B_2_pin_2_ and phenyl chloroformate 2a under the optimal reaction conditions, a 1,5-borocarbonylated product (4) was obtained in 62% yield. This observation indicates that the reaction proceeds *via* an addition of Cu–Bpin to the C–C double bond followed by a β-C elimination and a carboxylation process (Scheme 5a). As mentioned above, the borocarbonylated products could be used in various transformations. For example, the C–B bond could be easily oxidized by NaBO_3_·4H_2_O to give the 1,4-diketone 5 in 95% yield from 3as (Scheme 5b). In another instance, treatment of 3aa with aqueous KHF_2_ delivered the corresponding potassium trifluoroborate 6 in 87% yield (Scheme 5c). Moreover, the Suzuki–Miyaura cross-coupling reaction of 3aa with iodobenzene worked smoothly to produce 7 in 92% yield (Scheme 5d).

**Scheme 6:**
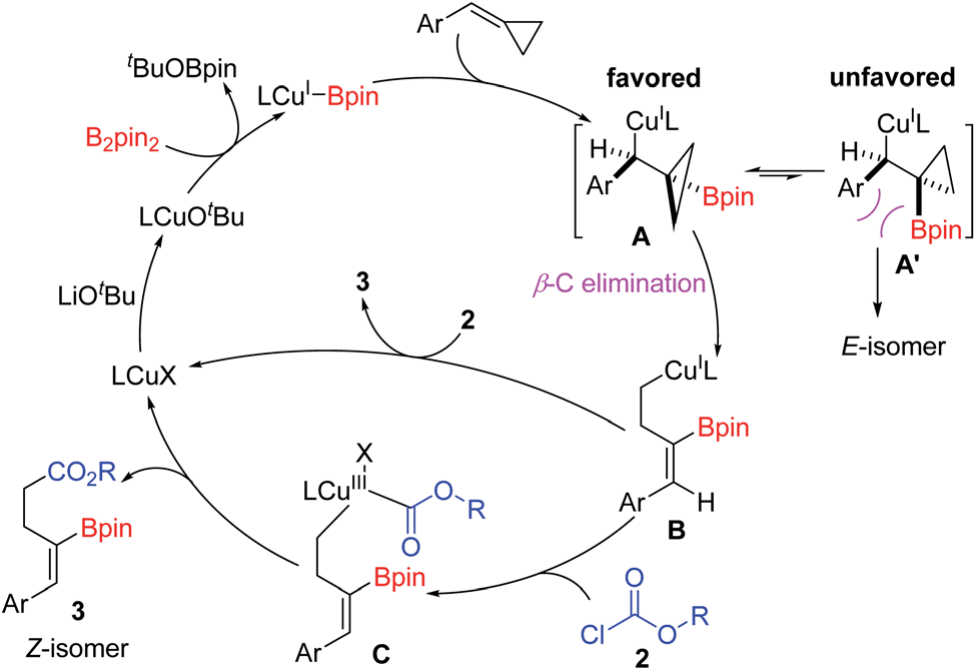
Proposed catalytic cycle.

On the basis of the experimental results and the previous literature,^11*j*^ a plausible catalytic cycle is proposed in Scheme 6. Firstly, the precatalyst IMesCuCl reacts with LiO^*t*^Bu to give an active (L)Cu–O^*t*^Bu complex, which reacts with B_2_pin_2_ to generate the key (L)Cu–Bpin species. Then, the copper–boryl intermediates react with BCPs *via syn*-1,2-migratory insertion into the C–C double-bond to afford the borylcuprated intermediate A, which undergoes a β-carbon elimination to generate a homoallylic copper(i) complex (B). The β-carbon elimination is the stereo-determining step.^16^ A σ-bond rotation should occur prior to the β-C elimination step to avoid the steric repulsion, thus favoring the formation of *Z* isomers. Subsequently, intermediate B reacts with acyl chloride to give Cu(iii) complex C*via* oxidative addition. Finally, reductive elimination of C releases the product 3 and regenerates (L)CuX, which is used for the next catalytic cycle. However, the direct nucleophilic attack of acyl chloride by the homoallylic copper(i) complex B to afford product 3 cannot be excluded.

## Conclusions

In summary, we have developed a copper catalyzed borocarbonylation of BCPs *via* proximal C–C bond cleavage for the synthesis of γ-boryl-γ,δ-unsaturated carbonyl compounds. Using substituted benzylidenecyclopropanes (BCPs) and chloroformates as starting material, a broad range of γ-boryl-γ,δ-unsaturated esters were prepared in moderate to excellent yields with excellent regio- and stereoselectivity. Besides, when aliphatic acid chlorides were used in this reaction, γ-boryl-γ,δ-unsaturated ketones could be produced in excellent yields. When substituted BCPs were used as substrates, the borocarbonylation occurred predominantly at the proximal C–C bond *trans* to the phenyl group in a regio- and stereoselective manner, which leads to the *Z*-isomers as the products. Control experiments indicate that the reaction proceeds *via* a mechanism involving migratory insertion of Cu–Bpin species to the C–C double bond of BCP and subsequent β-C elimination and acylation of the intermediary homoallylic Cu(i) complex. This efficient methodology involves the cleavage of a C–C bond and the formation of a C–C bond as well as a C–B bond, and provides a new method for the proximal C–C bond difunctionalization of BCPs.

## Data availability

All experimental data and detailed procedures are available in the ESI.†

## Author contributions

J.-B. P. conceived and directed the project. L.-M. Y. performed the experiments. H.-H. Z., X.-L. L. and A.-J. M. participated in substrate synthesis and discussions. L.-M. Y. and J.-B. P. wrote the manuscript and ESI.†

## Conflicts of interest

There are no conflicts to declare.

## Supplementary Material

SC-013-D2SC01992B-s001
