# Supplementary material for: Copper catalyzed borocarbonylation of benzylidenecyclopropanes through selective proximal C–C bond cleavage: synthesis of γ-boryl-γ,δ-unsaturated carbonyl compounds
Source: Chem Sci. 2022 May 30;13(24):7304–9. doi: 10.1039/d2sc01992b (PMC9214919; doi:10.1039/d2sc01992b)
Supplement: SC-013-D2SC01992B-s001 [file SC-013-D2SC01992B-s001.pdf]

Electronic Supplementary Material (ESI) for Chemical Science.
This journal is © The Royal Society of Chemistry 2022

Supporting information

Copper Catalyzed Borocarbonylation of Benzylidenecyclopropanes through Selective Proximal C−C Bond Cleavage: Synthesis of *γ*-Boryl-*γ*,*δ*-unsaturated Carbonyl Compounds

Li-Miao Yang,[†] Hui-Hui Zeng,[†] Xin-Lian Liu,[†] Ai-Jun Ma,[†] and Jin-Bao Peng*,[†]

[†]School of Biotechnology and Health Sciences, Wuyi University, Jiangmen, Guangdong 529020, P. R. China; orcid.org/0000-0002-0568-7740; E-mail: pengjb_05@126.com

Table of Contents

1. General Information

2. Preparation of Substrates

3. Optimization of Reaction Conditions

4. General Procedure

5. Spectroscopic Data of Products

6. Derivatization Experiments

7. References

8. Copies of NMR Spectra for Compounds

1. General Information

Reagents, solvents, and analytical methods:

Unless otherwise noted, all reactions were carried out under a nitrogen atmosphere. methylcyclopropanes (MCPs) were synthesized according to existing method. The reagents were ordered from TCI, Energy Chemical and Bidepharm used without purification. All solvents were dried by standard techniques and distilled prior to use. [1]NMR spectra were recorded on a Bruker Ascend™ 500 spectrometer (500 MHz) and chemical shifts (δ values) were reported in parts per million (ppm) relative to internal tetramethylsilane standard (TMS, 0ppm) and spectral data were reported in ppm relative to tetramethylsilane (TMS) as internal standard and $CDCl_3$ (^{1}H NMR δ 7.27, ^{13}C NMR δ 77.0) as solvent. All coupling constants (J) are reported in Hz. The following abbreviations were used to describe peak splitting patterns when appropriate: s = singlet, d = doublet, dd = double doublet, ddd = double doublet of doublets, t = triplet, dt = double triplet, q = quatriplet, m = multiplet. All highresolution mass spectra (HRMS) were obtained on a Thermo Scientific™ Q Exactive™ UHMR (Ultra High Mass Range) Hybrid Quadrupole-Orbitrap™ mass spectrometer.

2. Preparation of the Starting Materials.

2.1 Synthesis of phosphonium salts[1]

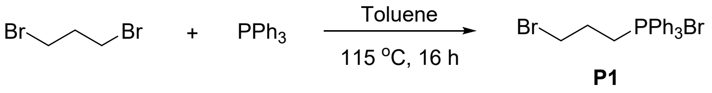

An oven-dried 250 mL two-neck round-bottom flask equipped with a Teflon magnetic stirring bar, a reflux condenser and an oil bubbler were connected to nitrogen. The system was charged with PPh_3 (25.0 g, 95.2 mmol, 1.0 equiv) and toluene (100 mL). The vessel was sealed with a rubber septum and vigorously stirred at room temperature before adding 1,3-dibromopropane (10.2 mL, 20.2 g, 100 mmol, 1.05 equiv). The system was placed on a heating block and the temperature was increased to 115 °C with vigorously stirring. After 16 h, the reaction was cooled down to room temperature. The white precipitate was filtered off in vacuo, washed with toluene (3×15 mL) and dried under reduced pressure to afford P1 as a white solid.

2.2 Synthesis of methylcyclopropanes (MCPs)[2]

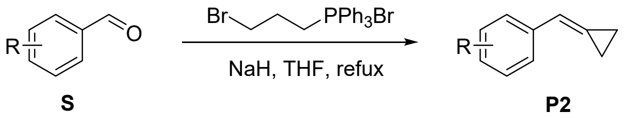

A solution of (4-bromobutyl)triphenylphosphonium bromide (6.01 g, 13 mmol) and NaH (1.04 g, 26 mmol) in THF (25 mL) was stirred at 70 ^{o}C under N_2 for 12 h. Afterwards compound S (10 mmol) in THF (5 mL) was added and the reaction solution was stirred at 70 ^{o}C until compound S was consumed completely. The reaction mixture was cooled to room temperature, and the mixture was filtered through a celite. The filtrate was concentrated under reduced pressure and the residue was purified by silica gel flash chromatography (eluent: petroleum ether) to afford the product P2 in moderate yield.

All of the chloroformate and acid chloride were from commercial sources and used without further purification(**2a to 2w**).

methylene cyclopropane:

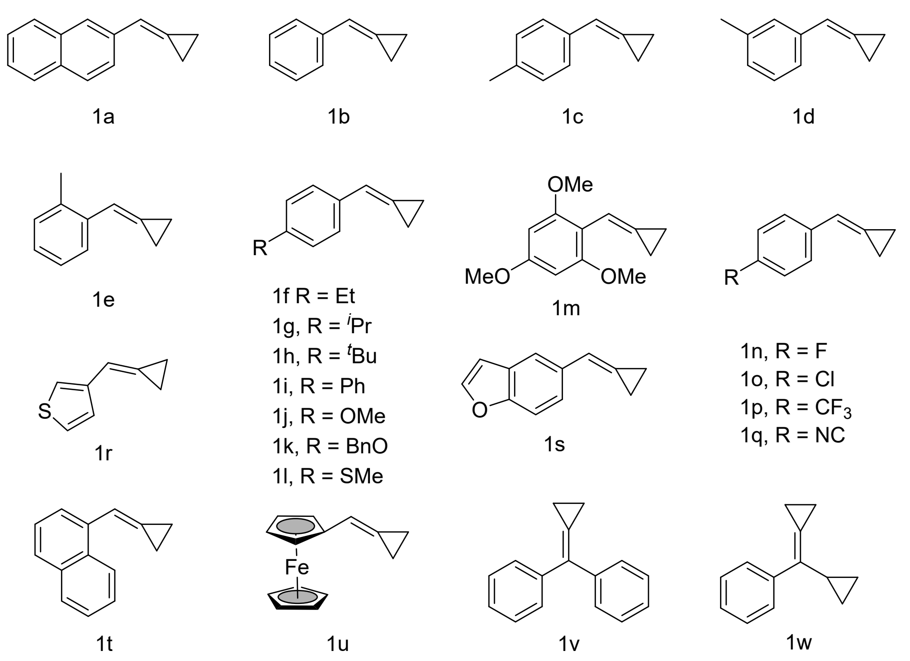

Figure S1 Substrate of Methylene Cyclopropane

Chloroformate:

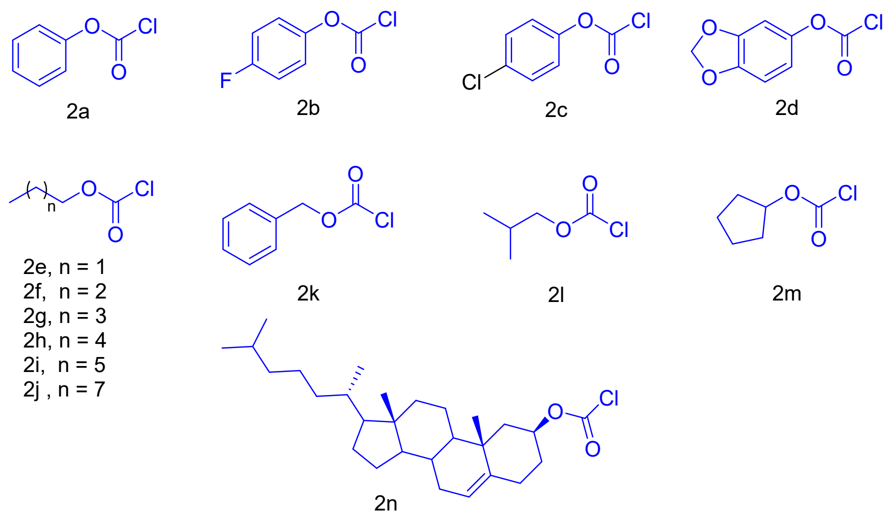

Acid Chloride:

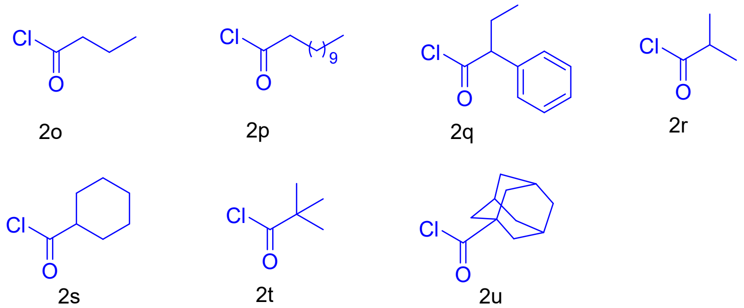

Figure S2 Substrate of Chloroformate and Acid Chloride

3. Optimization of the Reaction Conditions

Table S1. Screening of Catalysts[a]

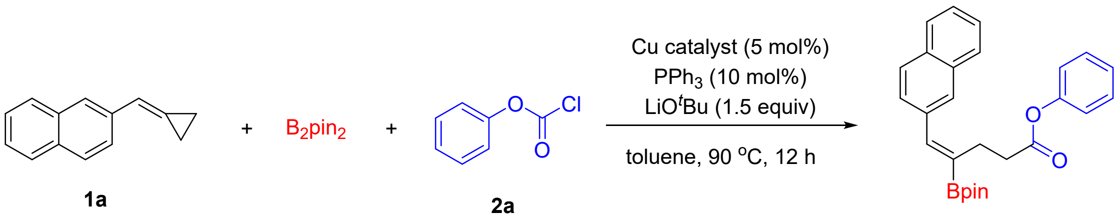

Entry	Catalyst	Yield (%)[b]
**1**	**IMesCuCl**	**58**
2	IPrCuCl	26
3	CuTc	0
4	$Cu(CH_3CN)_4PF_6$	0
5	$Cu(OAc)_2$	0

6	CuBr	n.r
7	$CuBr_2$	0
8	/	n.r

[a]Reaction conditions: **1a** (0.10 mmol), **2a** (0.15 mmol), Cu catalysit (5 mol%), PPh_3 (10 mol%), NaO^{t}Bu (0.15 mmol) in toluene (1.0 mL) at 90 °C for 12 h. [b]:Isolated yield.

Table S2. Screening of the Ligand[a]

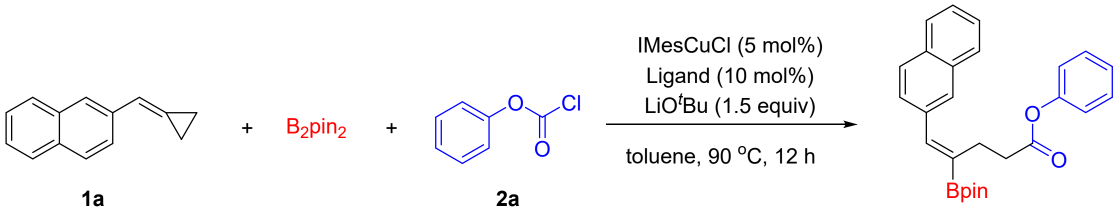

Entry	Ligand	Yield (%)[b]
1	PPh_3	58
2	$BuPAd_2$	29
3	DPPP	50
4	DMF	trace
**5**	**BINAP**	**67**
6	Xantphos	trace
7	dtbbpy	59
8	/	trace

[a]Reaction conditions: **1a** (0.10 mmol), **2a** (0.15 mmol), IMesCuCl (5 mol%), Ligand (5 mol%), NaO^{t}Bu (0.15 mmol) in toluene (1.0 mL) at 90 °C for 12 h. [b]Isolated yields.

Table S3. Screening of the amount of the ligand[a]

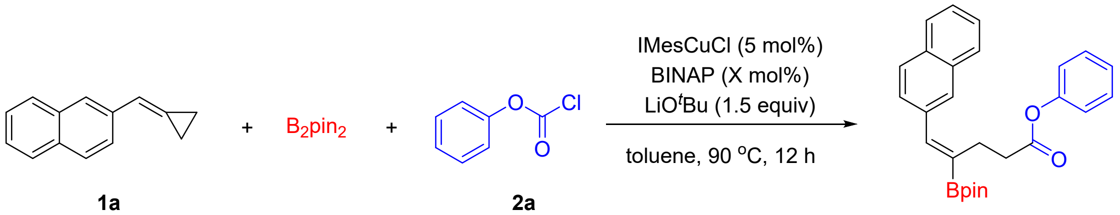

Entry	X (mol%)	Yield (%)[b]
1	2.5	57
2	5	67
**3**	**7.5**	**69**
4	10	59

[a]Reaction conditions: **1a** (0.10 mmol), **2a** (0.15 mmol), IMesCuCl (5 mol%), BINAP (X mol%), NaO^{t}Bu (0.15 mmol) in toluene (1.0 mL) at 90 °C. [b]:Isolated yield.

Table S4. Screening of the base[a]

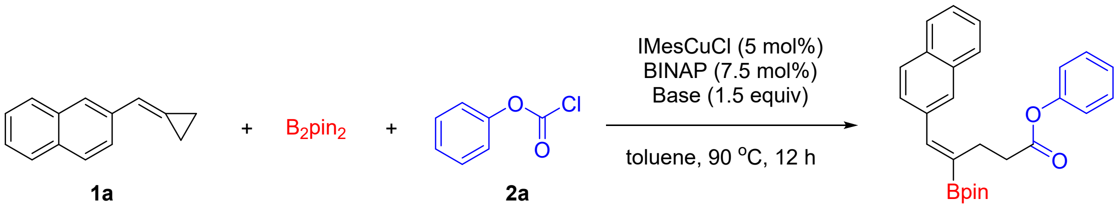

Entry	base	Yield (%)^b
1	Et_3N	n.r
2	K_2CO_3	n.r
3	Cs_2CO_3	n.r
4	Na_2CO_3	trace
**5**	**LiO^{t}Bu**	**69**
6	KO^{t}BU	58
7	NaO^{t}Bu	0
8	NaOMe	42

^{a}Reaction conditions: **1a** (0.10 mmol), **2a** (0.15 mmol), IMesCuCl (5 mol%), BINAP (7.5 mol%), base (0.15 mmol) in toluene (1.0 mL) at 90 °C. ^{b}:Isolated yield.

Table S5. Screening of the amunt of the **2a**(Phenyl chloroformate)^a

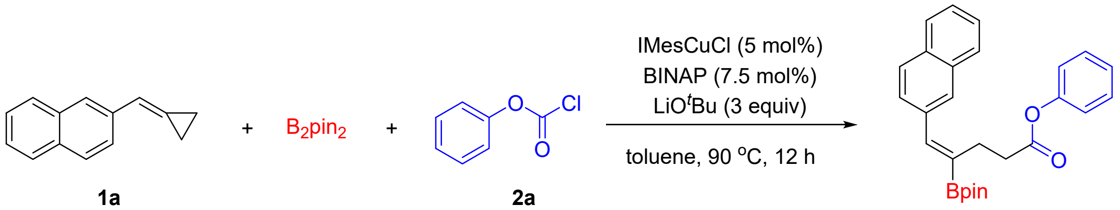

Entry	Y Equiv(SM_3)	Yield (%)^b
1	1.5	69
3	2	62
4	2.5	63
5	3	81
**6**	**3.5**	**89**

^{a}Reaction conditions: **1a** (0.10 mmol), **2a** (Y mmol), IMesCuCl (5 mol%), BINAP (7.5 mol%), LiO^{t}Bu (Y mmol) in toluene (1.0 mL) at 90 °C. ^{b}:Isolated yield.

Table S6. Screening of the Solvent^a

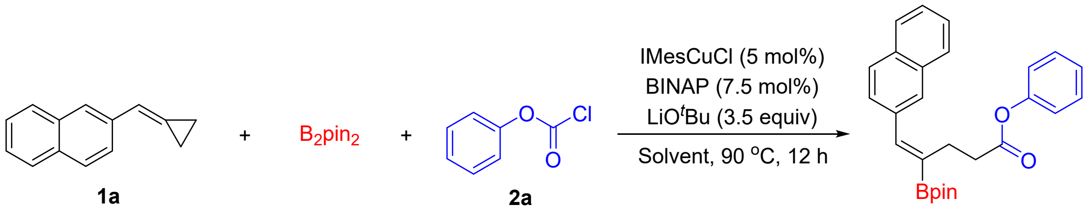

Entry	Solvent	Yield (%)^b
**1**	**toluent**	**89**
2	PhCl	76

3	DCM	19
4	DMSO	n.r
5	DMF	trace
6	MeCN	43
7	THF	trace
8	Dioxane	trace

[a]Reaction conditions: **1a** (0.10 mmol), **2a** (0.35 mmol), IMesCuCl (5 mol%), BINAP (7.5 mol%), LiO^{t}Bu (0.35 mmol) in toluene (1.0 mL) at 90 °C. [b]:Isolated yield.

Table S7. Screening of the Temperature[a]

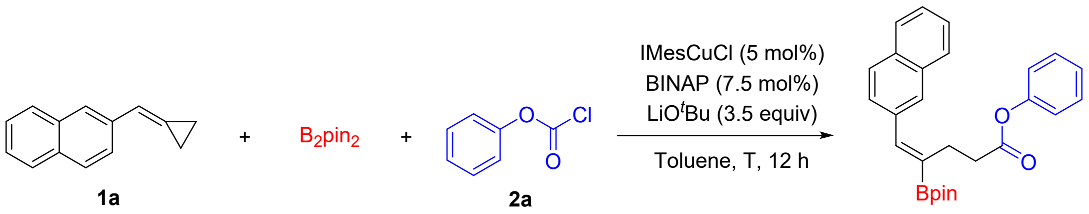

Entry	Temp.(℃)	Yield (%)[b]
1	70	77
2	80	84
3	90	89
**4**	**100**	**91**
4[c]	100	86
5	110	79

[a]Reaction conditions: **1a** (0.10 mmol), **2a** (0.35 mmol), IMesCuCl (5 mol%), BINAP (7.5 mol%), LiO^{t}Bu (0.35 mmol) in toluene (1.0 mL) at 100 °C for 12 h. [b]:Isolated yield. [c]:3.0 equiv 2a.

Table S8. Screening of the Additive[a]

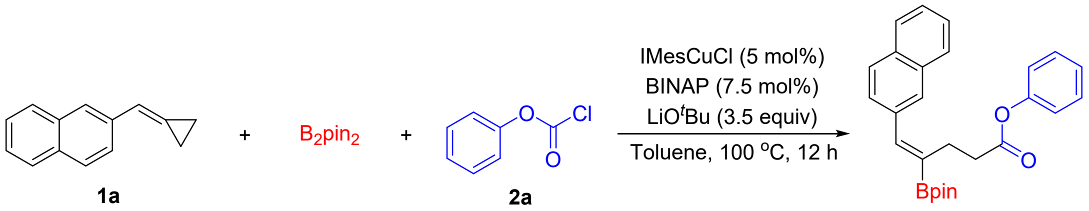

Entry	Additive	Yield (%)[b]
1	3Å Ms	89
2	4Å Ms	81
3	5Å Ms	80

[a]Reaction conditions: **1a** (0.10 mmol), **2a** (0.35 mmol), IMesCuCl (5 mol%), BINAP (7.5 mol%), LiO^{t}Bu (0.35 mmol) in toluene (1.0 mL) at 100 °C for 12 h. [b]:Isolated yield.

4. General Procedure

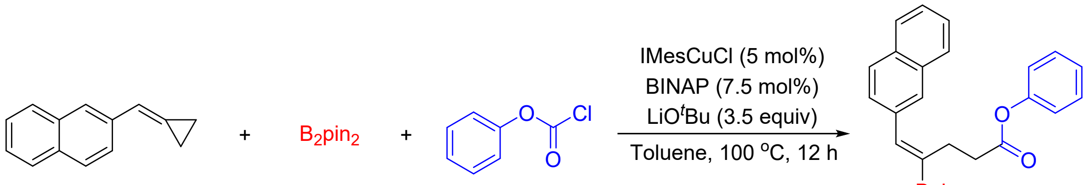

1a **2a**

Under nitrogen atmosphere, **1a** (18.0 mg, 0.10 mmol), B_2pin_2 (38.1 mg, 0.15 mmol), IMesCuCl (2.1 mg, 5 mol %), BINAP (4.7 mg, 7.5 mol%) and LiO^{t}Bu (24 mg, 0.35 mmol) were added to an oven-dried 15 mL sealed tube. Then, **2a** (44µl, 0.35 mmol) and Toluene (1 mL) were added to the reaction. The tube was sealed and the mixture was stirred at 100 °C for 12 h. After the reaction was completed, the reaction mixture was filtered and concentrated under vacuum. The crude product was purified by column chromatography on silica gel to afford the corresponding product **3aa** to **3ua**.

1 mmol scale: A 15 mL sealed tube containing **1a** (0.18 g, 1.0 mmol), B_2pin_2 (0.38 g, 1.5 mmol), IMesCuCl (0.02 g mol %), BINAP (0.05 g, 7.5 mol%) and LiO^{t}Bu (0.24g, 0.35 mmol) were added to an oven-dried 15 mL sealed tube. Then, **2a** (0.4 mL, 0.35 mmol) and Toluene (3 mL) were added to the reaction. The tube was sealed and the mixture was stirred at 100 °C for 12 h. After the reaction was completed, the reaction mixture was filtered and concentrated under vacuum. The crude product was purified by column chromatography on silica gel to afford the **3aa** product(0.31 g, 72%).

5. Characterization Data of the Corresponding Products

Phenyl (*Z*)-5-(naphthalen-2-yl)-4-(4,4,5,5-tetramethyl-1,3,2-dioxaborolan-2-yl)pent-4-enoate (**3aa**)

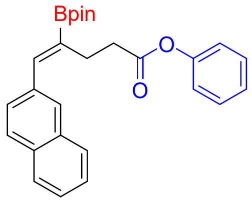

The title compound was prepared from the reaction of **1a** (18.0 mg, 0.10 mmol) and **2a** (44µl, 0.35 mmol) according to the General Procedure. It was purified by flash columnchromatography on silica gel (40:1 petroleum ether/ethyl acetate) 38.9 mg (91% yield) a colorless oil.

^{1}H NMR (500 MHz, CDCl$_3$) δ 7.95-7.78 (m, 4H), 7.64-7.42 (m, 4H), 7.36 (t, J = 7.9 Hz, 2H), 7.23 (t, J = 7.4 Hz, 1H), 7.07 (d, J = 7.7 Hz, 2H), 3.09-2.95 (m, 2H), 2.93-2.79 (m, 2H), 1.38 (s, 12H); **^{13}C NMR (126 MHz, CDCl$_3$)** δ 171.9, 150.8, 143.9, 134.9, 133.3, 132.6, 129.6, 129.3, 128.3, 128.2, 127.9, 127.6, 127.1, 126.2, 125.7, 121.7, 83.8, 34.3, 25.1, 24.9 (quaternary carbon next to boron

could not be observed because of quadrupolar coupling effects); **HRMS (ESI)**: calcd. for $C_{27}H_{29}BO_4$ $[M+Na]^+$: 451.2059, found 451.2053.

4-Fluorophenyl(*Z*)-5-(naphthalen-2-yl)-4-(4,4,5,5-tetramethyl-1,3,2-dioxaborolan-2-yl)pent-4-enoate (**3ab**)

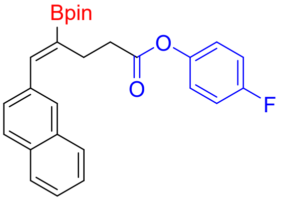

The title compound was prepared from the reaction of **1a** (18.0 mg, 0.10 mmol) and **2b** (46µl, 0.35 mmol) according to the General Procedure. It was purified by flash columnchromatography on silica gel (40:1 petroleum ether/ethyl acetate) 38.9 mg (81% yield) a colorless oil.

^{1}H NMR (500 MHz, CDCl$_3$) δ 7.84 (dd, *J* = 8.9, 2.8 Hz, 4H), 7.53 (s, 1H), 7.52-7.43 (m, 3H), 7.05-6.93 (m, 4H), 3.02-2.93 (m, 2H), 2.89-2.77 (m, 2H), 1.37 (s, 12H); **^{13}C NMR (126 MHz, CDCl$_3$)** δ 171.8, 160.1 (d, $J_{(C\text{-}F)}$ = 244.5 Hz), 146.5 (d, $J_{(C\text{-}F)}$ = 0.35Hz), 143.9, 134.8, 133.2, 132.5, 128.2, 128.1, 127.8, 127.6, 126.9, 126.1, 123.0, 122.9, 115.9 (d, $J_{(C\text{-}F)}$ = 23.2 Hz), 83.7, 34.2, 25.0, 24.8 (quaternary carbon next to boron could not be observed because of quadrupolar coupling effects); **^{19}F NMR (471 MHz, CDCl$_3$)** δ -117.3; **HRMS (ESI)**: calcd. for $C_{27}H_{28}BFO_4$ $[M+Na]^+$: 469.1965, found 469.1965.

4-Chlorophenyl(*Z*)-5-(naphthalen-2-yl)-4-(4,4,5,5-tetramethyl-1,3,2-dioxaborolan-2-yl)pent-4-enoate (**3ac**)

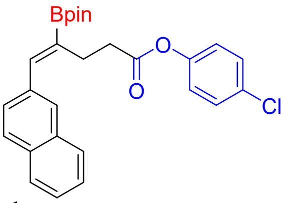

The title compound was prepared from the reaction of **1a** (18.0 mg, 0.10 mmol) and **2c** (49µl, 0.35 mmol) according to the General Procedure. It was purified by flash columnchromatography on silica gel (40:1 petroleum ether/ethyl acetate) 25.1 mg (75% yield) a colorless oil.

^{1}H NMR (500 MHz, CDCl$_3$) δ 7.83 (dd, *J* = 7.6, 5.2 Hz, 4H), 7.53 (s, 1H), 7.51-7.45 (m, 4H), 7.00-6.94 (m, 3H), 3.00-2.93 (m, 2H), 2.82 (t, *J* = 7.9 Hz, 2H), 1.41-1.32 (m, 12H); **^{13}C NMR (126 MHz, CDCl$_3$)** δ 171.5, 149.2, 144.0, 134.8, 133.2, 132.5, 130.9, 129.3, 128.2, 128.1, 127.8, 127.6, 127.0, 126.2, 123.0, 116.6, 83.7, 29.7, 24.8, 14.1(quaternary carbon next to boron could not be observed because of quadrupolar coupling effects); **HRMS (ESI)**: calcd. for $C_{27}H_{28}BClO_4$ $[M+K]^+$: 501.1404, found 501.1405.

Benzo[*d*][1,3]dioxol-5-yl(*Z*)-5-(naphthalen-2-yl)-4-(4,4,5,5-tetramethyl-1,3,2-dioxaborolan-2-yl)pent-4-enoate (**3ad**)

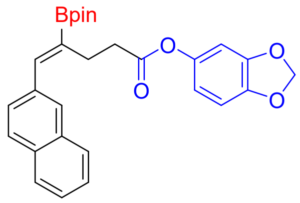

The title compound was prepared from the reaction of **1a** (18.0 mg, 0.10 mmol) and **2d** (70.2 mg, 0.35 mmol) according to the General Procedure. It was purified by flash columnchromatography on silica gel (25:1 petroleum ether/ethyl acetate) 35.4 mg (75% yield) a colorless oil.

^{1}H NMR (500 MHz, CDCl$_3$) δ 7.83 (dd, J = 6.9, 2.9 Hz, 4H), 7.50 (ddd, J = 9.6, 5.5, 3.2 Hz, 4H), 6.75-6.71 (m, 1H), 6.58 (d, J = 2.3 Hz, 1H), 6.48 (dd, J = 8.4, 2.3 Hz, 1H), 5.98 (s, 2H), 2.99-2.89 (m, 2H), 2.85-2.74 (m, 2H), 1.36 (s, 12H); **^{13}C NMR (126 MHz, CDCl$_3$)** δ 172.1, 147.8, 145.0, 143.8, 134.8, 133.2, 132.5, 128.2, 128.1, 127.8, 127.5, 127.0, 126.1, 113.9, 108.0, 107.8, 103.8, 103.2, 101.6, 83.7, 34.1, 25.0, 24.8 (quaternary carbon next to boron could not be observed because of quadrupolar coupling effects); **HRMS (ESI)**: calcd. for $C_{28}H_{29}BO_6$ [M+Na]$^+$: 495.1958, found 495.1959.

Propyl (*Z*)-5-(naphthalen-2-yl)-4-(4,4,5,5-tetramethyl-1,3,2-dioxaborolan-2-yl)pent-4-enoate (**3ae**)

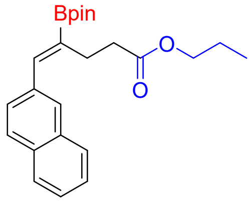

The title compound was prepared from the reaction of **1a** (18.0 mg, 0.10 mmol) and **2e** (39μl, 0.35 mmol) according to the General Procedure. It was purified by flash columnchromatography on silica gel (40:1 petroleum ether/ethyl acetate) 32.7 mg (83% yield) a colorless oil.

^{1}H NMR (500 MHz, CDCl$_3$) δ 7.89-7.77 (m, 4H), 7.58-7.39 (m, 4H), 4.01 (t, J = 6.8 Hz, 2H), 3.00-2.74 (m, 2H), 2.67-2.52 (m, 2H), 1.61 (dd, J = 14.3, 7.0 Hz, 2H), 1.35 (s, 12H), 1.30-1.25 (m, 2H), 0.91 (t, J = 7.4 Hz, 3H); **^{13}C NMR (126 MHz, CDCl$_3$)** δ 173.4, 143.3, 135.0, 133.2, 132.5, 128.2, 128.1, 127.7, 127.5, 127.0, 126.0, 83.6, 65.9, 34.3, 25.1, 24.8, 21.9, 10.4(quaternary carbon next to boron could not be observed because of quadrupolar coupling effects); **HRMS (ESI)**: calcd. for $C_{24}H_{31}BO_4$ [M+Na]$^+$: 417.2214, found 417.2213.

Butyl (*Z*)-5-(naphthalen-2-yl)-4-(4,4,5,5-tetramethyl-1,3,2-dioxaborolan-2-yl)pent-4-enoate (**3af**)

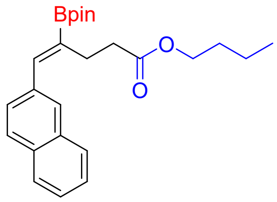

The title compound was prepared from the reaction of **1a** (18.0 mg, 0.10 mmol) and **2f** (45μl, 0.35 mmol) according to the General Procedure. It was purified by flash columnchromatography on silica gel (40:1 petroleum ether/ethyl acetate) 30.6 mg (75% yield) a colorless oil.

^{1}H NMR (500 MHz, CDCl$_3$) *δ* 7.83 (dd, *J* = 9.0, 5.7 Hz, 4H), 7.47 (dd, *J* = 11.5, 8.1 Hz, 4H), 4.04 (t, *J* = 6.7 Hz, 2H), 2.86-2.75 (m, 2H), 2.65-2.47 (m, 2H), 1.65-1.48 (m, 2H), 1.35 (s, 12H), 1.27 (d, *J* = 4.2 Hz, 2H), 0.90 (t, *J* = 7.4 Hz, 3H); **^{13}C NMR (126 MHz, CDCl$_3$)** δ 173.4, 143.3, 135.0, 133.2, 132.5, 128.2, 128.1, 127.7, 127.5, 127.0, 126.1, 122.7, 83.6, 64.2, 34.3, 30.6, 25.12, 2.1, 13.7 (quaternary carbon next to boron could not be observed because of quadrupolar coupling effects); **HRMS (ESI)**: calcd. for $C_{25}H_{33}BO_4$ $[M+Na]^+$: 431.2371, found 431.2374.

Pentyl (*Z*)-5-(naphthalen-2-yl)-4-(4,4,5,5-tetramethyl-1,3,2-dioxaborolan-2-yl)pent-4-enoate (**3ag**)

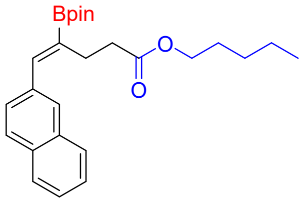

The title compound was prepared from the reaction of **1a** (18.0 mg, 0.10 mmol) and **2g** (49μl, 0.35 mmol) according to the General Procedure. It was purified by flash columnchromatography on silica gel (40:1 petroleum ether/ethyl acetate) 35.0 mg (83% yield) a colorless oil.

^{1}H NMR (500 MHz, CDCl$_3$) *δ* **^{1}H NMR (500 MHz, CDCl$_3$)** δ 7.87-7.78 (m, 4H), 7.54-7.41 (m, 4H), 4.03 (t, *J* = 6.8 Hz, 2H), 2.88- 2.74 (m, 2H), 2.64-2.47 (m, 2H), 1.66-1.45 (m, 2H), 1.35 (s, 12H), 1.30 (dd, *J* = 7.3, 3.8 Hz, 4H), 0.88 (t, *J* = 7.0 Hz, 3H); **^{13}C NMR (126 MHz, CDCl$_3$)** δ 173.4, 143.3, 143.3, 134.9, 133.2, 132.5, 128.2, 128.1, 127.7, 127.5, 127.0, 126.0, 83.6, 64.5, 34.3, 28.3, 28.0, 25.1, 24.8, 22.3, 13.9 (quaternary carbon next to boron could not be observed because of quadrupolar coupling effects); **HRMS (ESI)**: calcd. for $C_{26}H_{35}BO_4$ $[M+Na]^+$: 445.2528, found 445.2527.

Hexyl (*Z*)-5-(naphthalen-2-yl)-4-(4,4,5,5-tetramethyl-1,3,2-dioxaborolan-2-yl)pent-4-enoate (**3ah**)

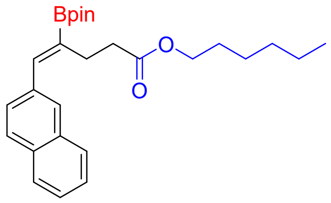

The title compound was prepared from the reaction of **1a** (18.0 mg, 0.10 mmol) and **2h** (51μl, 0.35 mmol) according to the General Procedure. It

was purified by flash columnchromatography on silica gel (40:1 petroleum ether/ethyl acetate) 35.7 mg (82% yield) a colorless oil.

^{1}H NMR (500 MHz, CDCl$_3$) δ 7.83 (dd, J = 8.8, 6.0 Hz, 4H), 7.47 (dd, J = 10.7, 8.6 Hz, 4H), 4.03 (t, J = 6.8 Hz, 2H), 2.90-2.77 (m, 2H), 2.66-2.46 (m, 2H), 1.72-1.48 (m, 2H), 1.34 (s, 12H), 1.30-1.24 (m, 6H), 0.88 (t, J = 6.8 Hz, 3H); **^{13}C NMR (126 MHz, CDCl$_3$)** δ 173.4, 143.3, 134.9, 133.2, 132.5, 132.5, 128.2, 128.1, 127.7, 127.5, 127.0, 83.6, 64.5, 34.3, 31.4, 28.5, 25.6, 25.1, 24.8, 22.5, 14.0(quaternary carbon next to boron could not be observed because of quadrupolar coupling effects); **HRMS (ESI)**: calcd. for $C_{27}H_{37}BO_4$ [M+Na]$^+$: 459.2685, found 459.2685.

Heptyl (*Z*)-5-(naphthalen-2-yl)-4-(4,4,5,5-tetramethyl-1,3,2-dioxaborolan-2-yl)pent-4-enoate (**3ai**)

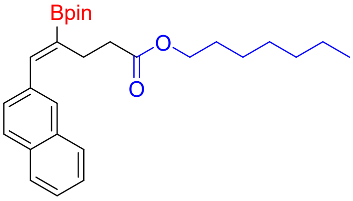

The title compound was prepared from the reaction of **1a** (18.0 mg, 0.10 mmol) and **2i** (63μl, 0.35 mmol) according to the General Procedure. It was purified by flash columnchromatography on silica gel (35:1 petroleum ether/ethyl acetate) 42.3 mg (94% yield) a colorless oil.

^{1}H NMR (500 MHz, CDCl$_3$) δ 7.90-7.77 (m, 4H), 7.48 (ddd, J = 13.0, 8.2, 6.0 Hz, 4H), 4.03 (t, J = 6.8 Hz, 2H), 2.89-2.74 (m, 2H), 2.58 (dd, J = 9.1, 7.1 Hz, 2H), 1.68-1.49 (m, 3H), 1.35 (s, 12H), 1.27 (s, 6H), 0.88 (t, J = 7.0 Hz, 4H); **^{13}C NMR (126 MHz, CDCl$_3$)** δ 173.4, 143.3, 134.9, 133.2, 132.5, 132.5, 128.2, 128.1, 127.7, 127.5, 127.0, 126.0, 83.6, 64.5, 34.3, 31.4, 28.5, 25.6, 25.1, 24.8, 22.5, 14.0 (quaternary carbon next to boron could not be observed because of quadrupolar coupling effects); **HRMS (ESI)**: calcd. for $C_{28}H_{39}BO_4$ [M+Na]$^+$: 473.2842, found 473.2843.

Nonyl (*Z*)-5-(naphthalen-2-yl)-4-(4,4,5,5-tetramethyl-1,3,2-dioxaborolan-2-yl)pent-4-enoate (**3aj**)

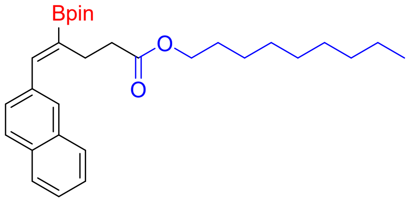

The title compound was prepared from the reaction of **1a** (18.0 mg, 0.10 mmol) and **2j** (75μl, 0.35 mmol) according to the General Procedure. It was purified by flash columnchromatography on silica gel (40:1 petroleum ether/ethyl acetate) 39.7 mg (83% yield) a colorless oil.

^{1}H NMR (500 MHz, CDCl$_3$) δ 7.83 (dd, J = 8.7, 6.0 Hz, 4H), 7.53-7.38 (m, 4H), 4.03 (t, J = 6.8 Hz, 2H), 2.86-2.75 (m, 2H), 2.64-2.50 (m, 2H), 1.57 (d, J = 7.2 Hz, 2H), 1.35 (s, 12H), 1.26 (s, 12H),

0.89 (t, J = 6.9 Hz, 3H); **^{13}C NMR (126 MHz, CDCl$_3$)** δ 173.4, 143.3, 134.9, 133.2, 132.5, 128.2, 128.1, 127.7, 127.5, 127.0, 126.0, 127.5, 83.6, 64.5, 34.3, 31.8, 29.4, 29.2, 29.2, 28.6, 25.9, 25.1, 24.8, 22.6, 14.1(quaternary carbon next to boron could not be observed because of quadrupolar coupling effects); **HRMS (ESI)**: calcd. for $C_{30}H_{43}BO_4$ [M+Na]$^+$: 501.3157, found 501.3155.

Benzyl (*Z*)-5-(naphthalen-2-yl)-4-(4,4,5,5-tetramethyl-1,3,2-dioxaborolan-2-yl)pent-4-enoate (**3ak**)

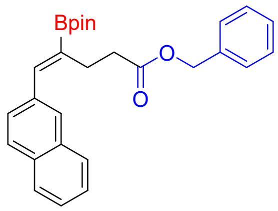

The title compound was prepared from the reaction of **1a** (18.0 mg, 0.10 mmol) and **2k** (49μl, 0.35 mmol) according to the General Procedure. It was purified by flash columnchromatography on silica gel (40:1 petroleum ether/ethyl acetate) 37.6 mg (85% yield) a colorless oil.

^{1}H NMR (500 MHz, CDCl$_3$) δ 7.86-7.75 (m, 4H), 7.48 (dd, J = 7.7, 4.0 Hz, 4H), 7.32 (t, J = 2.7 Hz, 5H), 5.09 (s, 2H), 2.94-2.79 (m, 2H), 2.69-2.55 (m, 2H), 1.33 (s, 12H); **^{13}C NMR (126 MHz, CDCl$_3$)** δ 173.1, 143.5, 136.0, 134.9, 133.2, 132.5, 132.5, 128.5, 128.2, 128.2, 128.1, 127.7, 127.5, 127.0, 126.1, 83.6, 66.1, 34.3, 25.1, 24.8 (quaternary carbon next to boron could not be observed because of quadrupolar coupling effects); **HRMS (ESI)**: calcd. for $C_{28}H_{31}BO_4$ [M+Na]$^+$: 465.2216, found 465.2213.

Isobutyl (*Z*)-5-(naphthalen-2-yl)-4-(4,4,5,5-tetramethyl-1,3,2-dioxaborolan-2-yl)pent-4-enoate (**3al**)

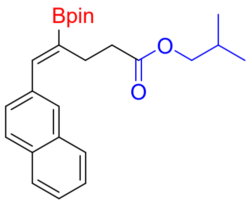

The title compound was prepared from the reaction of **1a** (18.0 mg, 0.10 mmol) and **2l** (48μ, 0.35 mmol) according to the General Procedure. It was purified by flash columnchromatography on silica gel (40:1 petroleum ether/ethyl acetate) 31.8 mg (78% yield) a colorless oil.

^{1}H NMR (500 MHz, CDCl$_3$) δ 7.86-7.76 (m, 4H), 7.51-7.46 (m, 3H), 7.45 (s, 1H), 3.83 (d, J = 6.7 Hz, 2H), 2.91-2.78 (m, 2H), 2.66-2.50 (m, 2H), 1.89 (dt, J = 13.4, 6.7 Hz, 1H), 1.35 (s, 12H), 0.90 (d, J = 6.7 Hz, 6H); **^{13}C NMR (126 MHz, CDCl$_3$)** δ 173.4, 143.3, 134.9, 133.2, 132.5, 128.2, 128.1, 127.7, 127.5, 127.0, 126.0, 83.6, 70.4, 34.3, 27.6, 25.2, 24.8, 19.1 (quaternary carbon next to boron could not be observed because of quadrupolar coupling effects); **HRMS (ESI)**: calcd. for $C_{25}H_{33}BO_4$ [M+Na]$^+$: 431.2371, found 431.2372.

Cyclopentyl (*Z*)-5-(naphthalen-2-yl)-4-(4,4,5,5-tetramethyl-1,3,2-dioxaborolan-2-yl)pent-4-enoate (**3am**)

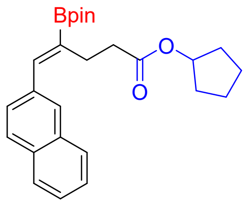

The title compound was prepared from the reaction of **1a** (18.0 mg, 0.10 mmol) and **2m** (44μl, 0.35 mmol) according to the General Procedure. It was purified by flash columnchromatography on silica gel (40:1 petroleum ether/ethyl acetate) 29.0 mg (69% yield) a colorless oil.

^{1}H NMR (500 MHz, CDCl$_3$) *δ* 7.94-7.75 (m, 4H), 7.63-7.31 (m, 4H), 5.14 (td, *J* = 6.2, 3.2 Hz, 1H), 2.89-2.76 (m, 2H), 2.61-2.42 (m, 2H), 1.82 (dt, *J* = 11.8, 6.0 Hz, 2H), 1.75-1.61 (m, 4H), 1.55 (dd, *J* = 5.9, 4.0 Hz, 2H), 1.35 (s, 12H); **^{13}C NMR (126 MHz, CDCl$_3$)** δ 173.2, 143.2, 134.9, 133.2, 132.5, 128.2, 128.2, 127.7, 127.5, 127.1, 126.0, 83.6, 34.5, 32.6, 25.2, 24.8, 23.7(quaternary carbon next to boron could not be observed because of quadrupolar coupling effects); **HRMS (ESI)**: calcd. for $C_{26}H_{33}BO_4$ $[M+Na]^+$: 443.2372, found 443.2371.

(2*S*,9*S*,13*R*)-9,13-dimethyl-17-((*R*)-6-methylheptan-2-yl)-2,3,4,8,9,9,10,11,12,13,14,15,16,17-tetradecahydro-1H-cyclopenta[*c*]phenanthren-2-yl(*Z*)-5-(naphthalen-2-yl)-4-(4,4,5,5-tetramethyl-1,3,2-dioxaborolan-2-yl)pent-4-enoate (**3an**)

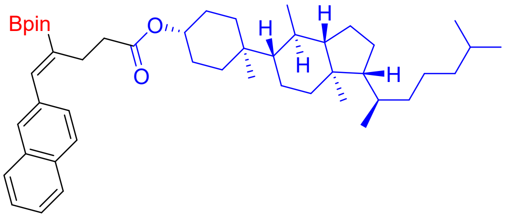

The title compound was prepared from the reaction of **1a** (18.0 mg, 0.10 mmol) and **2n** (157.2 mg, 0.35 mmol) according to the General Procedure. It was purified by flash columnchromatography on silica gel (30:1 petroleum ether/ethyl acetate) 23.8 mg (33% yield) a colorless oil.

^{1}H NMR (500 MHz, CDCl$_3$) *δ* 7.87-7.78 (m, 4H), 7.52-7.46 (m, 3H), 7.44 (s, 1H), 5.35 (d, *J* = 4.9 Hz, 1H), 4.65-4.54 (m, 1H), 2.88-2.79 (m, 2H), 2.62- 2.49 (m, 2H), 2.33-2.22 (m, 2H), 2.06-1.90 (m, 3H), 1.87-1.77 (m, 4H), 1.58-1.40 (m, 9H),1.35 (s, 12H), 1.13 (ddd, *J* = 19.2, 11.2, 5.3 Hz, 9H), 0.99 (s, 3H), 0.93 (d, *J* = 6.5 Hz, 4H), 0.88 (dd, *J* = 6.6, 2.3 Hz, 6H), 0.69 (s, 3H); **^{13}C NMR (126 MHz, CDCl$_3$)** δ 172.8, 143.3, 139.8, 134.9, 133.2, 132.5, 128.2, 128.2, 127.7, 127.5, 127.1, 126.0, 122.5, 83.6, 73.8, 56.7, 56.1, 50.0, 42.3, 39.7, 39.5, 38.1, 37.0, 36.5, 36.2, 35.8, 34.6, 31.9, 31.8, 28.2, 28.0, 27.7, 25.2, 24.8, 24.3, 23.8, 22.8, 22.6, 21.0, 19.3, 18.7, 11.8(quaternary carbon next to boron could

not be observed because of quadrupolar coupling effects); **HRMS (ESI)**: calcd. for $C_{48}H_{69}BO_4$ $[M+Na]^+$: 743.5191, found 743.5198.

(Z)-8-(naphthalen-2-yl)-7-(4,4,5,5-tetramethyl-1,3,2-dioxaborolan-2-yl)oct-7-en-4-one (**3ao**)

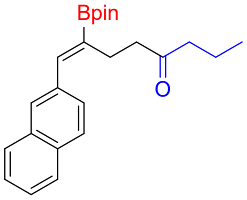

The title compound was prepared from the reaction of **1a** (18.0 mg, 0.10 mmol) and **2o** (42μl, 0.35 mmol) according to the General Procedure. It was purified by flash columnchromatography on silica gel (50:1 petroleum ether/ethyl acetate) 9.1 mg (24% yield) a colorless oil.

^{1}H NMR (500 MHz, CDCl$_3$) δ 7.81 (dd, J = 8.9, 4.2 Hz, 1H), 7.78 (s, 1H), 7.49-7.44 (m, 1H), 7.42 (s, 1H), 2.78-2.72 (m, 1H), 2.66-2.59 (m, 1H), 2.36 (t, J = 7.4 Hz, 1H), 1.60 (d, J = 7.3 Hz, 2H), 1.39-1.29 (m, 5H), 0.89 (t, J = 7.4 Hz, 2H); **^{13}C NMR (126 MHz, CDCl$_3$)** δ 210.9, 142.9, 128.2, 128.1, 127.7, 127.5, 127.0, 126.1, 126.0, 83.6, 44.6, 42.6, 24.8, 24.0, 17.3, 13.8. (quaternary carbon next to boron could not be observed because of quadrupolar coupling effects); **HRMS (ESI)**: calcd. for $C_{24}H_{31}BO_3$ $[M+H]^+$: 377.2293, found 377.2291.

(Z)-1-(naphthalen-2-yl)-2-(4,4,5,5-tetramethyl-1,3,2-dioxaborolan-2-yl)hexadec-1-en-5-one (**3ap**)

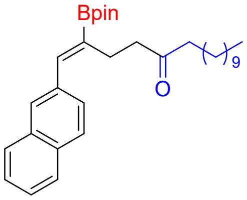

The title compound was prepared from the reaction of **1a** (18.0 mg, 0.10 mmol) and **2p** (73μl, 0.35 mmol) according to the General Procedure. It was purified by flash columnchromatography on silica gel (50:1 petroleum ether/ethyl acetate) 23.9 mg (49% yield) a colorless oil.

^{1}H NMR (500 MHz, CDCl$_3$) δ 7.87-7.71 (m, 4H), 7.46 (dd, J = 8.7, 7.6 Hz, 3H), 7.41 (s, 1H), 2.80-2.69 (m, 2H), 2.67-2.55 (m, 2H), 2.37 (t, J = 7.5 Hz, 2H), 1.33 (s, 12H), 1.24 (s, 18H), 0.88 (t, J = 6.9 Hz, 3H).; **^{13}C NMR (126 MHz, CDCl$_3$)** δ 211.1, 142.9, 135.0, 133.2, 132.5, 128.2, 128.1, 127.7, 127.5, 127.0, 126.1, 126.0, 83.6, 42.7, 42.6, 31.9, 29.6, 29.5, 29.4, 29.3, 29.3, 24.8, 24.0, 23.9, 22.7, 14.1(quaternary carbon next to boron could not be observed because of quadrupolar coupling effects); **HRMS (ESI)**: calcd. for $C_{32}H_{47}BO_3$ $[M+H]^+$: 489.3545, found 489.3540.

(*Z*)-8-(naphthalen-2-yl)-3-phenyl-7-(4,4,5,5-tetramethyl-1,3,2-dioxaborolan-2-yl)oct-7-en-4-one (**3aq**)

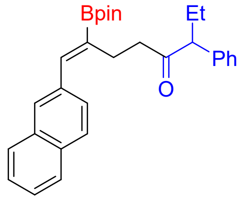

The title compound was prepared from the reaction of **1a** (18.0 mg, 0.10 mmol) and **2q** (58µl, 0.35 mmol) according to the General Procedure. It was purified by flash columnchromatography on silica gel (50:1 petroleum ether/ethyl acetate) 21.7 mg (48% yield) a colorless oil.

^{1}H NMR (500 MHz, CDCl$_3$) δ 7.80 (dd, J = 6.0, 3.4 Hz, 1H), 7.73 (dd, J = 9.0, 3.9 Hz, 2H), 7.68 (s, 1H), 7.46 (dd, J = 6.2, 3.2 Hz, 2H), 7.37-7.31 (m, 2H), 7.27 (t, J = 7.3 Hz, 2H), 7.23 (d, J = 7.2 Hz, 1H), 7.17 (d, J = 7.0 Hz, 2H), 3.51 (t, J = 7.4 Hz, 1H), 2.78-2.43 (m, 4H), 2.05 (dt, J = 14.1, 7.1 Hz, 1H), 1.79-1.66 (m, 1H), 1.28 (d, J = 6.7 Hz, 12H), 0.80 (t, J = 7.4 Hz, 3H); **^{13}C NMR (126 MHz, CDCl$_3$)** δ 210.2, 142.8, 139.1, 134.9, 133.2, 132.5, 128.7, 128.3, 128.2, 128.0, 127.7, 127.5, 127.0, 126.9, 126.0, 83.5, 60.5, 41.8, 29.7, 25.1, 24.8, 24.7, 24.0, 12.1(quaternary carbon next to boron could not be observed because of quadrupolar coupling effects); **HRMS (ESI)**: calcd. for $C_{30}H_{35}BO_3$ $[M+H]^+$: 453.2606, found 453.2603.

(*Z*)-2-methyl-7-(naphthalen-2-yl)-6-(4,4,5,5-tetramethyl-1,3,2-dioxaborolan-2-yl)hept-6-en-3-one (**3ar**)

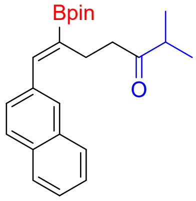

The title compound was prepared from the reaction of **1a** (18.0 mg, 0.10 mmol) and **2r** (31µl, 0.35 mmol) according to the General Procedure. It was purified by flash columnchromatography on silica gel (50:1 petroleum ether/ethyl acetate) 32.5 mg (86% yield) a colorless oil.

^{1}H NMR (500 MHz, CDCl$_3$) δ 7.85-7.77 (m, 4H), 7.48 (dd, J = 5.9, 3.2 Hz, 3H), 7.42 (s, 1H), 2.76 (dd, J = 9.0, 6.9 Hz, 2H), 2.72-2.66 (m, 2H), 2.65-2.57 (m, 1H), 1.35 (s, 12H), 1.26 (s, 1H), 1.09 (d, J = 6.9 Hz, 6H); **^{13}C NMR (126 MHz, CDCl$_3$)** δ 214.3, 142.8, 135.0, 133.2, 132.5, 128.2, 128.1, 127.70, 127.52, 126.99, 126.05, 126.02, 83.54, 40.65, 40.15, 24.81, 24.02, 18.23(quaternary carbon next to boron could not be observed because of quadrupolar coupling effects); **HRMS (ESI)**: calcd. for $C_{24}H_{31}BO_3$ $[M+Na]^+$: 401.2265, found 401.2264.

(*Z*)-1-cyclohexyl-5-(naphthalen-2-yl)-4-(4,4,5,5-tetramethyl-1,3,2-dioxaborolan-2-yl)pent-4-en-1-one (**3as**)

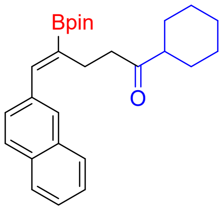

The title compound was prepared from the reaction of **1a** (18.0 mg, 0.10 mmol) and **2s** (47μl, 0.35 mmol) according to the General Procedure. It was purified by flash columnchromatography on silica gel (50:1 petroleum ether/ethyl acetate) 37.2 mg (89% yield) a white solid.

^{1}H NMR (500 MHz, $CDCl_3$) δ 7.81 (dd, J = 8.3, 4.6 Hz, 4H), 7.47 (dd, J = 8.8, 4.3 Hz, 3H), 7.42 (s, 1H), 2.74 (dd, J = 12.9, 5.4 Hz, 2H), 2.67 (dd, J = 13.1, 5.5 Hz, 2H), 2.39-2.26 (m, 1H), 1.82 (d, J = 12.8 Hz, 2H), 1.76 (dd, J = 9.1, 2.9 Hz, 2H), 1.66 (d, J = 9.1 Hz, 2H), 1.35 (s, 12H), 1.27 (s, 2H), 1.01-0.79 (m, 2H); **^{13}C NMR (126 MHz, $CDCl_3$)** δ 213.7, 142.8, 135.0, 133.2, 132.5, 128.2, 128.0, 127.7, 127.5, 127.0, 126.1, 126.0, 83.5, 50.6, 40.5, 29.7, 28.5, 25.9, 25.7, 24.8, 24.0(quaternary carbon next to boron could not be observed because of quadrupolar coupling effects); **HRMS (ESI)**: calcd. for $C_{27}H_{35}BO_3$ $[M+Na]^+$: 441.2580, found 441.2581.

(*Z*)-2,2-dimethyl-7-(naphthalen-2-yl)-6-(4,4,5,5-tetramethyl-1,3,2-dioxaborolan-2-yl)hept-6-en-3-one (**3at**)

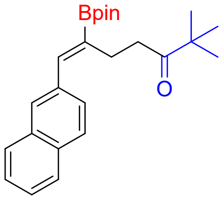

The title compound was prepared from the reaction of **1a** (18.0 mg, 0.10 mmol) and **2t** (66μl, 0.35 mmol) according to the General Procedure. It was purified by flash columnchromatography on silica gel (50:1 petroleum ether/ethyl acetate) 35.9 mg (92% yield) a colorless oil.

^{1}H NMR (500 MHz, $CDCl_3$) δ 7.82-7.76 (m, 4H), 7.51-7.45 (m, 3H), 7.41 (s, 1H), 2.73 (s, 4H), 1.34 (s, 12H), 1.13 (s, 9H); **^{13}C NMR (126 MHz, $CDCl_3$)** δ 215.5, 142.7, 135.1, 133.2, 132.5, 128.2, 128.1, 127.7, 127.5, 127.0, 126.0, 126.0, 83.5, 44.1, 36.5, 26.4, 24.8, 24.3(quaternary carbon next to boron could not be observed because of quadrupolar coupling effects); **HRMS (ESI)**: calcd. for $C_{25}H_{33}BO_3$ $[M+H]^+$: 391.2450, found 391.2446.

(*Z*)-1-((3*r*,5*r*,7*r*)-adamantan-1-yl)-5-(naphthalen-2-yl)-4-(4,4,5,5-tetramethyl-1,3,2-dioxaborolan-2-yl)pent-4-en-1-one (**3au**)

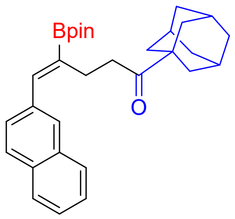

The title compound was prepared from the reaction of **1a** (18.0 mg, 0.10 mmol) and **2u** (66μl, 0.35 mmol) according to the General Procedure. It was purified by flash columnchromatography on silica gel (50:1 petroleum ether/ethyl acetate) 43.7 mg (93% yield) a colorless oil.

^{1}H NMR (500 MHz, CDCl$_3$) δ 7.81 (dd, J = 7.4, 4.9 Hz, 4H), 7.55-7.44 (m, 3H), 7.41 (s, 1H), 2.70 (t, J = 3.0 Hz, 4H), 2.03 (s, 3H), 1.81 (d, J = 2.4 Hz, 6H), 1.71 (dd, J = 36.5, 11.9 Hz, 7H), 1.35 (s, 12H); **^{13}C NMR (126 MHz, CDCl$_3$)** δ 215.2, 142.6, 135.1, 133.2, 132.5, 128.2, 128.1, 127.7, 127.5, 127.1, 126.0, 126.0, 83.5, 46.3, 38.2, 36.6, 36.0, 27.9, 24.9, 24.1(quaternary carbon next to boron could not be observed because of quadrupolar coupling effects); **HRMS (ESI)**: calcd. for $C_{31}H_{39}BO_3$ $[M+Na]^+$: 493.2895, found 493.2894.

Phenyl (*Z*)-5-phenyl-4-(4,4,5,5-tetramethyl-1,3,2-dioxaborolan-2-yl)pent-4-enoate (**3ba**)

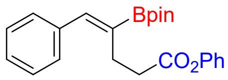

The title compound was prepared from the reaction of **1b** (13.0 mg, 0.10 mmol) and **2a** (44μl, 0.35 mmol) according to the General Procedure. It was purified by flash columnchromatography on silica gel (35:1 petroleum ether/ethyl acetate) 22.9 mg (66% yield) a colorless oil.

^{1}H NMR (500 MHz, CDCl$_3$) δ 7.41-7.34 (m, 7H), 7.30-7.27 (m, 3H), 7.22 (t, J = 7.9 Hz, 1H), 7.07 (dd, J = 8.5, 1.0 Hz, 2H), 2.93-2.85 (m, 2H), 2.83-2.75 (m, 2H), 1.34 (s, 12H); **^{13}C NMR (126 MHz, CDCl$_3$)** δ 171.8, 150.8, 143.8, 137.3, 129.3, 128.9, 128.3, 127.4, 125.6, 121.6, 83.6, 34.2, 24.9, 24.8(quaternary carbon next to boron could not be observed because of quadrupolar coupling effects); **HRMS (ESI)**: calcd. for $C_{23}H_{27}BO_4$ $[M+Na]^+$: 401.1900, found 401.1903.

Phenyl (*Z*)-4-(4,4,5,5-tetramethyl-1,3,2-dioxaborolan-2-yl)-5-(*p*-tolyl)pent-4-enoate (**3ca**)

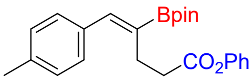

The title compound was prepared from the reaction of **1c** (14.4 mg, 0.10 mmol) and **2a** (44μl, 0.35 mmol) according to the General Procedure. It was purified by flash columnchromatography on silica gel (40:1 petroleum ether/ethyl acetate) 27.8 mg (71% yield) a colorless oil.

^{1}H NMR (500 MHz, CDCl$_3$) δ 7.37 (t, J = 7.8 Hz, 2H), 7.33 (s, 1H), 7.28 (d, J = 7.9 Hz, 2H), 7.22 (t, J = 7.6 Hz, 1H), 7.18 (d, J = 8.0 Hz, 2H), 7.07 (d, J = 8.2 Hz, 2H), 2.95-2.84 (m, 2H), 2.83-2.73

(m, 21H), 2.36 (s, 3H), 1.34 (s, 12H); **^{13}C NMR (126 MHz, CDCl$_3$)** δ 171.8, 150.8, 143.8, 137.3, 134.4, 129.3, 129.0, 128.9, 125.6, 121.6, 83.6, 34.2, 24.9, 24.8, 21.2(quaternary carbon next to boron could not be observed because of quadrupolar coupling effects); **HRMS (ESI)**: calcd. for $C_{24}H_{29}BO_4$ $[M+Na]^+$: 415.2057, found 410.2057.

Phenyl (Z)-4-(4,4,5,5-tetramethyl-1,3,2-dioxaborolan-2-yl)-5-(*m*-tolyl)pent-4-enoate (**3da**)

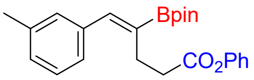

The title compound was prepared from the reaction of **1d** (14.4 mg, 0.10 mmol) and **2a** (44μl, 0.35 mmol) according to the General Procedure. It was purified by flash columnchromatography on silica gel (40:1 petroleum ether/ethyl acetate) 35.6 mg (91% yield) a colorless oil.

^{1}H NMR (500 MHz, CDCl$_3$) *δ* 7.37 (t, *J* = 7.8 Hz, 2H), 7.34-7.26 (m, 3H), 7.25-7.18 (m, 3H), 7.07 (d, *J* = 7.8 Hz, 2H), 2.91-2.84 (m, 2H), 2.82-2.75 (m, 2H), 2.50 (s, 3H), 1.34 (s, 12H); **^{13}C NMR (126 MHz, CDCl$_3$)** δ 171.8, 150.7, 143.1, 137.9, 134.0, 129.5, 129.3, 126.0, 125.6, 121.6, 83.6, 34.1, 25.0, 24.8, 15.5(quaternary carbon next to boron could not be observed because of quadrupolar coupling effects); **HRMS (ESI)**: calcd. for $C_{24}H_{29}BO_4$ $[M+Na]^+$: 415.2057, found 410.2056.

phenyl (*Z*)-4-(4,4,5,5-tetramethyl-1,3,2-dioxaborolan-2-yl)-5-(*o*-tolyl)pent-4-enoate (**3ea**)

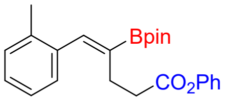

The title compound was prepared from the reaction of **1e** (14.4 mg, 0.10 mmol) and **2a** (44μl, 0.35 mmol) according to the General Procedure. It was purified by flash columnchromatography on silica gel (40:1 petroleum ether/ethyl acetate) 29.3 mg (75% yield) a colorless oil.

^{1}H NMR (500 MHz, CDCl$_3$) *δ* 7.39 (s, 1H), 7.35 (t, *J* = 7.9 Hz, 2H), 7.19 (dt, *J* = 7.7, 5.4 Hz, 5H), 7.04 (d, *J* = 7.7 Hz, 2H), 2.79-2.59 (m, 4H), 2.26 (s, 3H), 1.34 (s, 12H); **^{13}C NMR (126 MHz, CDCl$_3$)** δ 171.7, 150.7, 143.8, 136.8, 136.2, 129.9, 129.2, 128.6, 127.4, 125.6, 125.4, 121.6, 85.6, 83.5, 34.2, 25.0, 24.8, 19.9. (quaternary carbon next to boron could not be observed because of quadrupolar coupling effects); **HRMS (ESI)**: calcd. for $C_{24}H_{29}BO_4$ $[M+H]^+$: 391.2086, found 391.2081.

Phenyl (*Z*)-5-(4-ethylphenyl)-4-(4,4,5,5-tetramethyl-1,3,2-dioxaborolan-2-yl)pent-4-enoate (**3fa**)

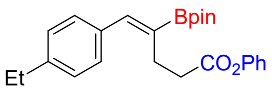

The title compound was prepared from the reaction of **1f** (15.8 mg, 0.10 mmol) and **2a** (44μl, 0.35 mmol) according to the General Procedure. It was purified by flash columnchromatography on silica gel (35:1 petroleum ether/ethyl acetate) 30.9 mg (76% yield) a colorless oil.

^{1}H NMR (500 MHz, CDCl$_3$) *δ* 7.41-7.29 (m, 5H), 7.21 (t, *J* = 6.6 Hz, 3H), 7.07 (d, *J* = 7.6 Hz, 2H), 2.95-2.85 (m, 2H), 2.84-2.75 (m, 2H), 2.67 (q, *J* = 7.6 Hz, 2H), 1.34 (s, 12H), 1.26 (t, *J* = 7.7 Hz, 3H); **^{13}C NMR (126 MHz, CDCl$_3$)** δ 171.9, 150.8, 143.8, 143.6, 134.7, 129.3, 129.1, 127.8, 125.6, 121.6, 83.6, 34.2, 28.6, 24.9, 24.8, 15.4(quaternary carbon next to boron could not be observed because of quadrupolar coupling effects); **HRMS (ESI)**: calcd. for $C_{25}H_{31}BO_4$ $[M+Na]^+$: 429.2215, found 429.2215.

Phenyl (*Z*)-5-(4-isopropylphenyl)-4-(4,4,5,5-tetramethyl-1,3,2-dioxaborolan-2-yl)pent-4-enoate (**3ga**)

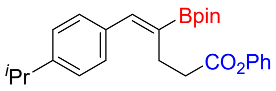

The title compound was prepared from the reaction of **1g** (17.2 mg, 0.10 mmol) and **2a** (44μl, 0.35 mmol) according to the General Procedure. It was purified by flash columnchromatography on silica gel (40:1 petroleum ether/ethyl acetate) 33.6 mg (80% yield) a colorless oil.

^{1}H NMR (500 MHz, CDCl$_3$) *δ* 7.37 (t, *J* = 7.9 Hz, 1H), 7.32 (d, *J* = 8.4 Hz, 1H), 7.23 (d, *J* = 7.9 Hz, 1H), 7.07 (d, *J* = 7.6 Hz, 1H), 2.96-2.84 (m, 1H), 2.84-2.74 (m, 1H), 1.34 (s, 6H), 1.27 (d, *J* = 6.9 Hz, 3H), 1.22 (t, *J* = 7.2 Hz, 1H); **^{13}C NMR (126 MHz, CDCl$_3$)** δ 171.9, 150.8, 148.2, 143.8, 134.8, 129.3, 129.1, 126.4, 125.6, 121.6, 83.6, 34.3, 33.8, 25.0, 24.8, 23.9(quaternary carbon next to boron could not be observed because of quadrupolar coupling effects); **HRMS (ESI)**: calcd. for $C_{26}H_{33}BO_4$ $[M+Na]^+$: 443.2372, found 443.2371.

Phenyl (*Z*)-5-(4-(tert-butyl)phenyl)-4-(4,4,5,5-tetramethyl-1,3,2-dioxaborolan-2-yl)pent-4-enoate (**3ha**)

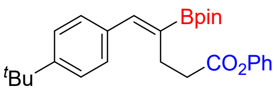

The title compound was prepared from the reaction of **1h** (18.6 mg, 0.10 mmol) and **2a** (44μl, 0.35 mmol) according to the General Procedure. It was purified by flash columnchromatography on silica gel (40:1 petroleum ether/ethyl acetate) 36.0 mg (83% yield) a colorless oil.

^{1}H NMR (500 MHz, CDCl$_3$) δ 7.43-7.35 (m, 4H), 7.35-7.30 (m, 3H), 7.23 (t, J = 7.4 Hz, 1H), 7.08 (dd, J = 8.5, 0.9 Hz, 2H), 2.95-2.86 (m, 2H), 2.84-2.74 (m, 2H), 1.34 (s, 12H); **^{13}C NMR (126 MHz, CDCl$_3$)** δ 171.9, 150.8, 150.4, 143.7, 134.4, 129.3, 128.8, 125.6, 125.2, 121.6, 83.6, 34.6, 34.3, 31.3, 25.0, 24.8(quaternary carbon next to boron could not be observed because of quadrupolar coupling effects); **HRMS (ESI)**: calcd. for $C_{27}H_{35}BO_4$ [M+Na]$^+$: 457.2529, found 457.2528.

Phenyl (*Z*)-5-([1,1'-biphenyl]-4-yl)-4-(4,4,5,5-tetramethyl-1,3,2-dioxaborolan-2-yl)pent-4-enoate (**3ia**)

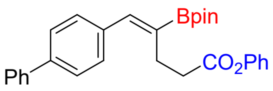

The title compound was prepared from the reaction of **1i** (20.6 mg, 0.10 mmol) and **2a** (44μl, 0.35 mmol) according to the General Procedure. It was purified by flash columnchromatography on silica gel (40:1 petroleum ether/ethyl acetate) 40.4 mg (89% yield) a colorless oil.

^{1}H NMR (500 MHz, CDCl$_3$) δ 7.62 (t, J = 7.1 Hz, 4H), 7.50-7.42 (m, 4H), 7.40-7.34 (m, 4H), 7.22 (t, J = 7.4 Hz, 1H), 7.08 (d, J = 8.6 Hz, 4H), 2.98-2.90 (m, 2H), 2.89-2.77 (m, 2H), 1.36 (s, 12H); **^{13}C NMR (126 MHz, CDCl$_3$)** δ 171.8, 150.8, 143.3, 140.6, 140.1, 136.3, 129.5, 129.3, 128.8, 127.3, 127.0, 126.9, 125.6, 121.6, 83.7, 34.2, 25.0, 24.8(quaternary carbon next to boron could not be observed because of quadrupolar coupling effects); **HRMS (ESI)**: calcd. for $C_{29}H_{31}BO_4$ [M+Na]$^+$: 477.2217, found 477.2216.

Pphenyl (*Z*)-5-(4-methoxyphenyl)-4-(4,4,5,5-tetramethyl-1,3,2-dioxaborolan-2-yl)pent-4-enoate(**3ja**)

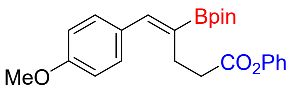

The title compound was prepared from the reaction of **1j** (16.0 mg, 0.10 mmol) and **2a** (44μl, 0.35 mmol) according to the General Procedure. It was purified by flash columnchromatography on silica gel (15:1 petroleum ether/ethyl acetate) 27.7 mg (68% yield) a colorless oil.

^{1}H NMR (500 MHz, CDCl$_3$) δ 7.40-7.33 (m, 4H), 7.30 (s, 1H), 7.23 (t, J = 7.5 Hz, 1H), 7.08 (d, J = 7.8 Hz, 2H), 6.91 (d, J = 8.7 Hz, 2H), 3.83 (s, 3H), 2.98-2.86 (m, 2H), 2.84-2.76 (m, 2H), 1.33 (s, 12H); **^{13}C NMR (126 MHz, CDCl$_3$)** δ 171.9, 158.9, 150.8, 143.3, 130.5, 129.9, 129.3, 125.6, 121.6, 113.7, 83.5, 55.2, 34.2, 24.9, 24.8(quaternary carbon next to boron could not be observed because of

quadrupolar coupling effects); **HRMS (ESI)**: calcd. for $C_{24}H_{29}BO_5$ $[M+Na]^+$: 431.2007, found 431.2005.

Pphenyl (*Z*)-5-(4-(benzyloxy)phenyl)-4-(4,4,5,5-tetramethyl-1,3,2-dioxaborolan-2-yl)pent-4-enoate (**3ka**)

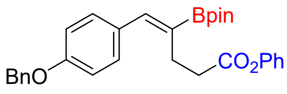

The title compound was prepared from the reaction of **1k** (23.6 mg, 0.10 mmol) and **2a** (44μl, 0.35 mmol) according to the General Procedure. It was purified by flash columnchromatography on silica gel (35:1 petroleum ether/ethyl acetate) 36.8 mg (76% yield) a colorless oil.

^{1}H NMR (500 MHz, CDCl$_3$) δ 7.46 (d, J = 7.1 Hz, 2H), 7.41 (t, J = 8.0 Hz, 3H), 7.36 (dd, J = 11.1, 4.5 Hz, 5H), 7.30 (s, 1H), 7.23 (t, J = 7.4 Hz, 1H), 7.08 (d, J = 7.6 Hz, 2H), 6.98 (d, J = 8.7 Hz, 2H), 5.09 (s, 2H), 2.98-2.87 (m, 2H), 2.85-2.75 (m, 2H), 1.34 (s, 12H); **^{13}C NMR (126 MHz, CDCl$_3$)** δ 171.9, 158.1, 150.8, 143.3, 136.8, 130.5, 130.2， 129.3, 128.6, 128.0, 127.5, 125.6, 121.6, 114.6, 83.5, 69.9, 34.2, 24.9, 24.8(quaternary carbon next to boron could not be observed because of quadrupolar coupling effects); **HRMS (ESI)**: calcd. for $C_{30}H_{33}BO_5$ $[M+Na]^+$: 507.2323, found 507.2322.

Phenyl (*Z*)-5-(4-(methylthio)phenyl)-4-(4,4,5,5-tetramethyl-1,3,2-dioxaborolan-2-yl)pent-4-enoate (**3la**)

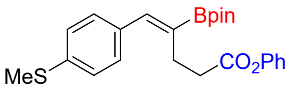

The title compound was prepared from the reaction of **1l** (17.6 mg, 0.10 mmol) and **2a** (44μl, 0.35 mmol) according to the General Procedure. It was purified by flash columnchromatography on silica gel (35:1 petroleum ether/ethyl acetate) 34.3 mg (81% yield) a colorless oil.

^{1}H NMR (500 MHz, CDCl$_3$) δ 7.37 (t, J = 7.7 Hz, 2H), 7.34-7.29 (m, 3H), 7.24 (d, J = 8.3 Hz, 3H), 7.07 (d, J = 8.2 Hz, 2H), 2.94-2.84 (m, 2H), 2.83-2.73 (m, 2H), 2.50 (s, 3H), 1.34 (s, 12H); **^{13}C NMR (126 MHz, CDCl$_3$)** δ 171.8, 150.7, 143.1, 137.9, 134.0, 129.5, 129.3, 126.0, 125.6, 121.6, 83.6, 34.1, 25.0, 24.8, 15.5(quaternary carbon next to boron could not be observed because of quadrupolar coupling effects); **HRMS (ESI)**: calcd. for $C_{24}H_{29}BO_4S$ $[M+Na]^+$: 447.1779, found 447.1778.

Phenyl (*Z*)-4-(4,4,5,5-tetramethyl-1,3,2-dioxaborolan-2-yl)-5-(2,4,6-trimethoxyphenyl)pent-4-enoate (**3ma**)

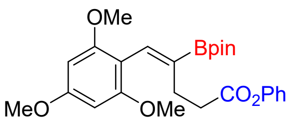

The title compound was prepared from the reaction of **1m** (22.0 mg, 0.10 mmol) and **2a** (44μl, 0.35 mmol) according to the General Procedure. It was purified by flash columnchromatography on silica gel (5:1 petroleum ether/ethyl acetate) 33.7 mg (72% yield) a colorless oil.

^{1}H NMR (500 MHz, CDCl$_3$) δ 7.36 (t, J = 7.9 Hz, 2H), 7.21 (t, J = 7.4 Hz, 1H), 7.10-7.00 (m, 3H), 6.14 (s, 2H), 3.84 (s, 3H), 3.79 (s, 6H), 2.86-2.67 (m, 2H), 2.56-2.45 (m, 2H), 1.33 (s, 12H); **^{13}C NMR (126 MHz, CDCl$_3$)** δ 172.5, 160.8, 158.3, 150.9, 135.7, 129.2, 125.5, 121.7, 107.8, 90.2, 83.3, 55.5, 55.3, 33.7, 27.3, 24.9(quaternary carbon next to boron could not be observed because of quadrupolar coupling effects); **HRMS (ESI)**: calcd. for $C_{26}H_{33}BO_7$ [M+Na]$^+$: 491.2219, found 491.2218.

Phenyl (*Z*)-4-(4,4,5,5-tetramethyl-1,3,2-dioxaborolan-2-yl)-5-(2,4,6-trimethoxyphenyl)pent-4-enoate (**3na**)

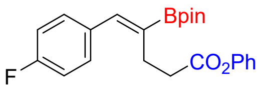

The title compound was prepared from the reaction of **1n** (14.8 mg, 0.10 mmol) and **2a** (44μl, 0.35 mmol) according to the General Procedure. It was purified by flash columnchromatography on silica gel (35:1 petroleum ether/ethyl acetate) 37.2 mg (94% yield) a colorless oil.

^{1}H NMR (500 MHz, CDCl$_3$) δ 7.41-7.32 (m, 4H), 7.31 (s, 1H), 7.23 (t, J = 7.4 Hz, 1H), 7.10-6.99 (m, 4H), 2.89-2.82 (m, 2H), 2.79 (ddd, J = 9.0, 5.2, 1.8 Hz, 2H), 1.34 (s, 12H); **^{13}C NMR (126 MHz, CDCl$_3$)** δ 171.7, 161.9 (d, $J_{(C\text{-}F)}$ = 247.8 Hz), 150.7, 142.6, 130.7 (d, $J_{(C\text{-}F)}$ = 8.1 Hz), 129.3, 125.7, 121.6, 115.3, 115.2, 83.7, 34.1, 24.8(quaternary carbon next to boron could not be observed because of quadrupolar coupling effects); **^{19}F NMR (471 MHz, CDCl$_3$)** δ -114.1; **HRMS (ESI)**: calcd. for $C_{23}H_{26}BFO_4$ [M+Na]$^+$: 419.1806, found 419.1805.

Phenyl(*Z*)-2-(1-(4-chlorophenyl)-5-(phenylperoxy)-5l2-pent-1-en-2-yl)-4,4,5,5-tetramethyl-1,3,2-dixaborolane (**3oa**)

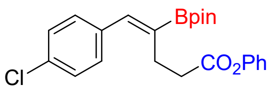

The title compound was prepared from the reaction of **1o** (16.5 mg, 0.10 mmol) and **2a** (44μl, 0.35 mmol) according to the General Procedure. It was purified by flash columnchromatography on silica gel (35:1 petroleum ether/ethyl acetate) 33.0 mg (80% yield) a colorless oil.

^{1}H NMR (500 MHz, CDCl$_3$) δ 7.41-7.36 (m, 2H), 7.33 (d, J = 8.6 Hz, 2H), 7.30 (d, J = 2.1 Hz, 3H), 7.23 (t, J = 7.4 Hz, 1H), 7.06 (d, J = 7.6 Hz, 2H), 2.84 (dd, J = 11.2, 4.8 Hz, 2H), 2.80-2.74 (m, 2H), 1.34 (s, 12H); **^{13}C NMR (126 MHz, CDCl$_3$)** δ 171.7, 150.7, 142.4, 135.7, 133.2, 130.2, 129.3, 128.5, 125.67, 121.6, 83.7, 34.0, 24.9, 24.8(quaternary carbon next to boron could not be observed because of quadrupolar coupling effects); **HRMS (ESI)**: calcd. for $C_{23}H_{26}BClO_4$ [M+Na]$^+$: 435.1514, found 435.1514.

Phenyl(*Z*)-4-(4,4,5,5-tetramethyl-1,3,2-dioxaborolan-2-yl)-5-(4-(trifluoromethyl)phenyl)pent-4-enoate (**3pa**)

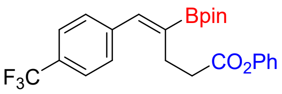

The title compound was prepared from the reaction of **1p** (19.8 mg, 0.10 mmol) and **2a** (44μl, 0.35 mmol) according to the General Procedure. It was purified by flash columnchromatography on silica gel (40:1 petroleum ether/ethyl acetate) 19.6 mg (44% yield) a colorless oil.

^{1}H NMR (500 MHz, CDCl$_3$) δ 7.62 (d, J = 8.2 Hz, 2H), 7.45 (d, J = 8.1 Hz, 2H), 7.37 (dd, J = 9.3, 6.5 Hz, 3H), 7.23 (t, J = 7.4 Hz, 1H), 7.07-7.01 (m, 2H), 2.93-2.65 (m, 4H), 1.35 (s, 12H); **^{13}C NMR (126 MHz, CDCl$_3$)** δ 171.6, 150.7, 142.2, 140.9, 129.3, 129.1, 125.7, 125.2 (d, $J_{(C\text{-}F)}$ = 3.8 Hz), 122.2 (d, $J_{(C\text{-}F)}$ = 200.8 Hz), 121.5, 83.9, 34.0, 24.91, 24.8.(quaternary carbon next to boron could not be observed because of quadrupolar coupling effects); **^{19}F NMR (471 MHz, CDCl$_3$)** δ -62.5 ; **HRMS (ESI)**: calcd. for $C_{24}H_{26}B_F3O_4$ [M+Na]$^+$: 419.1806, found 419.1805.

Phenyl (*Z*)-5-(4-cyanophenyl)-4-(4,4,5,5-tetramethyl-1,3,2-dioxaborolan-2-yl)pent-4-enoate (**3qa**)

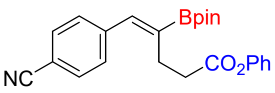

The title compound was prepared from the reaction of **1q** (15.5 mg, 0.10 mmol) and **2a** (44μl, 0.35 mmol) according to the General Procedure. It was purified by flash columnchromatography on silica gel (25:1 petroleum ether/ethyl acetate) 33.0 mg (82% yield) a colorless oil.

^{1}H NMR (500 MHz, CDCl$_3$) δ 7.65 (d, J = 8.3 Hz, 2H), 7.44 (d, J = 8.1 Hz, 2H), 7.40-7.34 (m, 2H), 7.33 (s, 1H), 7.23 (t, J = 7.4 Hz, 1H), 7.05 (d, J = 7.6 Hz, 2H), 2.81 (s, 4H), 1.34 (s, 12H); **^{13}C NMR (126 MHz, CDCl$_3$)** δ 171.5, 150.6, 142.0, 141.6, 132.1, 129.4, 129.4, 125.8, 121.5, 118.9, 110.8, 84.0, 33.8, 25.0, 24.8(quaternary carbon next to boron could not be observed because of quadrupolar coupling effects); **HRMS (ESI)**: calcd. for $C_{24}H_{26}BNO_4$ $[M+Na]^+$: 426.1854, found 426.1854.

Phenyl (*Z*)-4-(4,4,5,5-tetramethyl-1,3,2-dioxaborolan-2-yl)-5-(thiophen-3-yl)pent-4-enoate (**3ra**)

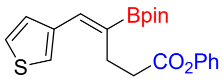

The title compound was prepared from the reaction of **1r** (13.6 mg, 0.10 mmol) and **2a** (44μl, 0.35 mmol) according to the General Procedure. It was purified by flash columnchromatography on silica gel (30:1 petroleum ether/ethyl acetate) 26.9 mg (70% yield) a colorless oil.

^{1}H NMR (500 MHz, CDCl$_3$) δ 7.43 (d, J = 2.6 Hz, 1H), 7.38 (t, J = 7.9 Hz, 2H), 7.32 (dd, J = 5.0, 2.9 Hz, 1H), 7.29 (s, 1H), 7.27-7.24 (m, 1H), 7.23 (s, 1H), 7.09 (d, J = 7.7 Hz, 2H), 2.99-2.87 (m, 2H), 2.86-2.75 (m, 2H), 1.33 (s, 12H); **^{13}C NMR (126 MHz, CDCl$_3$)** δ 171.9, 150.7, 138.5, 137.4, 129.3, 128.8, 125.7, 125.4, 125.4, 121.6, 83.6, 33.9, 25.4, 24.8(quaternary carbon next to boron could not be observed because of quadrupolar coupling effects); **HRMS (ESI)**: calcd. for $C_{21}H_{25}BO_4S$ $[M+Na]^+$: 407.1464, found 407.1463.

Phenyl (*Z*)-5-(benzofuran-5-yl)-4-(4,4,5,5-tetramethyl-1,3,2-dioxaborolan-2-yl)pent-4-enoate (**3sa**)

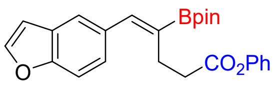

The title compound was prepared from the reaction of **1s** (17.0 mg, 0.10 mmol) and **2a** (44μl, 0.35 mmol) according to the General Procedure. It was purified by flash columnchromatography on silica gel (35:1 petroleum ether/ethyl acetate) 31.4 mg (75% yield) a colorless oil.

^{1}H NMR (500 MHz, CDCl$_3$) δ 7.66-7.61 (m, 2H), 7.51-7.45 (m, 2H), 7.37 (t, J = 7.9 Hz, 2H), 7.31 (dd, J = 8.6, 1.5 Hz, 1H), 7.22 (t, J = 7.4 Hz, 1H), 7.06 (d, J = 7.6 Hz, 2H), 6.77 (dd, J = 2.0, 0.5 Hz, 1H), 2.97-2.90 (m, 2H), 2.87-2.76 (m, 2H), 1.35 (s, 12H); **^{13}C NMR (126 MHz, CDCl$_3$)** δ 171.8, 154.2, 145.4, 144.1, 132.2, 129.3, 129.2, 127.4, 125.7, 125.6, 121.6, 121.5, 111.1, 106.8, 83.6, 34.2, 24.9, 24.8(quaternary carbon next to boron could not be observed because of quadrupolar coupling effects); **HRMS (ESI)**: calcd. for $C_{25}H_{27}BO_5$ $[M+K]^+$: 457.1591, found 457.1583.

Phenyl (*Z*)-5-(naphthalen-1-yl)-4-(4,4,5,5-tetramethyl-1,3,2-dioxaborolan-2-yl)pent-4-enoate (**3ta**)

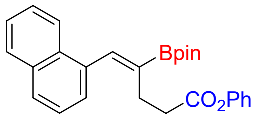

The title compound was prepared from the reaction of **1t** (18.0 mg, 0.10 mmol) and **2a** (44μl, 0.35 mmol) according to the General Procedure. It was purified by flash columnchromatography on silica gel (35:1 petroleum ether/ethyl acetate) 14.6 mg (34% yield) a colorless oil.

^{1}H NMR (500 MHz, $CDCl_3$) δ 7.98 (dd, J = 6.4, 2.6 Hz, 1H), 7.87 (dd, J = 6.2, 3.1 Hz, 1H), 7.84-7.77 (m, 2H), 7.55-7.44 (m, 3H), 7.37 (d, J = 7.0 Hz, 1H), 7.31 (t, J = 7.9 Hz, 2H), 7.18 (t, J = 7.4 Hz, 1H), 6.97 (d, J = 8.2 Hz, 2H), 2.71 (s, 4H), 1.39 (s, 12H); **^{13}C NMR (126 MHz, $CDCl_3$)** δ 171.7, 150.7, 142.7, 134.8, 133.5, 131.5, 129.2, 128.3, 127.7, 126.0, 125.9, 125.8, 125.5, 125.3, 125.26, 121.6, 83.7, 34.3, 25.4, 24.9(quaternary carbon next to boron could not be observed because of quadrupolar coupling effects); **HRMS (ESI)**: calcd. for $C_{27}H_{29}BO_4$ $[M+Na]^+$: 451.2059, found 451.2058.

Phenyl (*Z*)-5-(Ferroceneyl)-4-(4,4,5,5-tetramethyl-1,3,2-dioxaborolan-2-yl)pent-4-enoate (**3ua**)

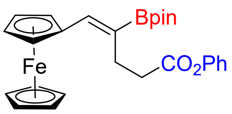

The title compound was prepared from the reaction of **1u** (23.8 mg, 0.10 mmol) and **2a** (44μl, 0.35 mmol) according to the General Procedure. It was purified by flash columnchromatography on silica gel (35:1 petroleum ether/ethyl acetate) 27.8 mg (56% yield) a yellow solid.

^{1}H NMR (500 MHz, DMSO) δ 7.42 (t, J = 6.9 Hz, 2H), 7.25 (t, J = 7.3 Hz, 1H), 7.18-7.07 (m, 2H), 6.94 (s, 1H), 4.53 (s, 2H), 4.37 (d, J = 7.3 Hz, 3H), 4.12 (s, 5H), 2.76-2.68 (m, 2H), 2.68-2.62 (m, 2H), 1.24 (s, 12H); **^{13}C NMR (126 MHz, DMSO)** δ 172.3, 151.5, 143.3, 130.4, 126.8, 122.8, 84.1, 81.1, 70.9, 70.6, 70.1, 34.4, 26.2, 25.6.(quaternary carbon next to boron could not be observed because of quadrupolar coupling effects); **HRMS (ESI)**: calcd. for $C_{27}H_{31}BFeO_4$ $[M+Na]^+$: 509.1569, found 509.1566.

Phenyl 5,5-diphenyl-4-(4,4,5,5-tetramethyl-1,3,2-dioxaborolan-2-yl)pent-4-enoate (**3va**)

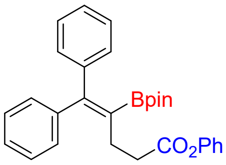

The title compound was prepared from the reaction of **1v** (18.0 mg, 0.10 mmol) and **2a** (44μl, 0.35 mmol) according to the General Procedure. It was purified by flash

columnchromatography on silica gel (35:1 petroleum ether/ethyl acetate) 10.9 mg (24% yield) a colorless oil.

^{1}H NMR (500 MHz, $CDCl_3$) δ 7.35 (dt, J = 15.2, 7.7 Hz, 4H), 7.24 (dd, J = 13.8, 9.0 Hz, 7H), 7.18 (d, J = 7.1 Hz, 2H), 7.07 (d, J = 7.8 Hz, 2H), 2.85-2.47 (m, 4H), 1.17 (s, 12H); **^{13}C NMR (126 MHz, $CDCl_3$)** δ 171.5, 153.1, 150.7, 144.2, 141.7, 129.3, 129.2, 129.0, 128.2, 127.7, 127.2, 127.0, 125.6, 121.6, 83.5, 34.5, 28.4, 24.7 (quaternary carbon next to boron could not be observed because of quadrupolar coupling effects); **HRMS (ESI)**: calcd. for $C_{29}H_{31}BO_4$ $[M+Na]^+$: 477.2217, found 477.2215.

6. Derivatization Experiments

6.1 Mechanism Exploration

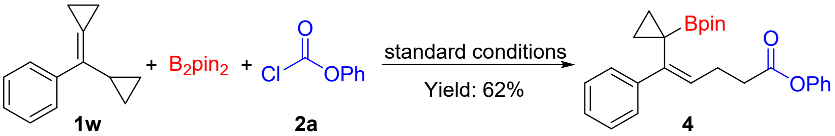

1w **2a** **4**

4 was prepared according to the following procedure. An oven-dried 15 mL sealed tube was loaded with (cyclopropyl(cyclopropylidene)methyl)benzene (0.10 mmol, 17 mg), B_2pin_2 (0.15 mmol, 38.1 mg) , 2a (0.35 mmol, 44μl), IMesCuCl (0.005mmol, 2.1mg), BINAP (0.075 mmol, 4.7 mg) and LiO^{t}Bu (0.35 mmol, 28 mg) ,then toluene (1 mL) was added. The reaction mixture was stirred at 100 °C for the 12 h. After dilution with EtOAc, the reaction mixture was filtrated on silica (EtOAc) and concentrated under reduced pressure. After a flash column chromatography (*n*-hexane/EtOAc 30:1) afforded the product **4** (25.9 mg, 62% yield) as a colorless oil.

^{1}H NMR (500 MHz, $CDCl_3$) δ 7.42-7.38 (m, 2H), 7.37-7.34 (m, 2H), 7.27 (dd, J = 13.9, 11.4 Hz, 4H), 7.21 (dd, J = 15.0, 7.8 Hz, 1H), 7.14-7.07 (m, 2H), 5.97-5.83 (m, 1H), 2.94-2.63 (m, 4H), 1.28 (s, 12H), 1.20 (dd, J = 6.0, 3.4 Hz, 2H), 0.63 (q, J = 3.5 Hz, 2H); **^{13}C NMR (126 MHz, $CDCl_3$)** δ 172.0, 150.7, 144.1, 143.2, 129.4, 129.2, 127.8, 126.5, 126.3, 125.7, 121.6, 83.6, 34.1, 24.8, 24.6, 15.5; **HRMS (ESI)**: calcd. for $C_{26}H_{31}BO_4$ $[M+Na]^+$: 441.2215, found 441.2216.

6.2 Oxidation of the **3ap**[3]

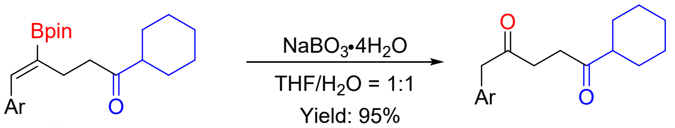

3as (Ar = 2-Naphthyl) **5**

5 was prepared according to a modified procedure. To a solution of **3as** (0.1 mmol, 41.8 mg) in THF/H_2O=1:1 was added $NaBO_3$•$4H_2O$ (0.5 mmol, 76.9mg) and the mixture was vigorously stirred at room temperature for 5 h. The reaction was quenched with water and NH_4Cl(sat) were added and the layers were separated. After extraction with EtOAc, the organic layers were dried over Na_2SO_4 and concentrated under reduced pressure. After a flash column chromatography (*n*-hexane/EtOAc 30:1) afforded the product **5** (29.3mg, 95% yield) as a colorless oil.

^{1}H NMR (500 MHz, CDCl$_3$) δ 7.83 (dt, J = 9.3, 5.2 Hz, 3H), 7.70 (s, 1H), 7.53-7.44 (m, 2H), 7.35 (dd, J = 8.4, 1.6 Hz, 1H), 3.93 (s, 2H), 2.78-2.74 (m, 2H), 2.74-2.69 (m, 2H), 2.38 (tt, J = 11.3, 3.4 Hz, 1H), 1.87 (d, J = 13.0 Hz, 2H), 1.82-1.73 (m, 2H), 1.68 (dd, J = 6.9, 5.0 Hz, 1H), 1.42-1.10 (m, 6H); **^{13}C NMR (126 MHz, CDCl$_3$)** δ 212.5, 207.2, 133.5, 132.4, 131.7, 128.3, 128.2, 127.6, 127.6, 127.5, 126.1, 125.7, 50.6, 50.2, 35.5, 34.1, 28.5, 25.8, 25.6; **HRMS (ESI)**: calcd. for $C_{21}H_{24}O_2$ $[M+Na]^+$: 331.1671, found 331.1673.

6.3 Synthesis of potassium salt.[3]

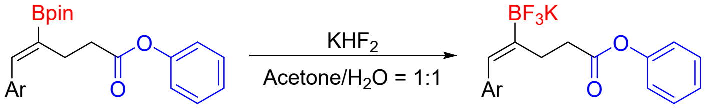

3aa (Ar = 2-Naphthyl) **6** Yield: 87%

6 was prepared according to a modified procedure. The solution of compound 3aa (85.6 mg, 0.2 mmol) and KHF_2 (5.1 equiv, 79.7 mg) in acetone: H_2O (1 mL: 1 mL) was stirred at 0 ℃ for 10 h. Then the reaction was determined by TLC analysis. After the 3aa was consumed completely, the reaction mixture was concentrated. The crude product was dissolved in MeOH, and evaporate again. Then the solids were triturated with hot acetone and filtered to remove inorganic salts. The resulting filtrate was concentrated to a minimal volume and EtOAc was added to afford **6** (35.5 mg, 87% yield) as a white solid.

^{1}H NMR (500 MHz, DMSO) δ 8.24 (dd, J = 11.6, 5.5 Hz, 3H), 8.11 (s, 1H), 7.90-7.73 (m, 5H), 7.64 (t, J = 7.9 Hz, 1H), 7.55-7.41 (m, 2H), 7.07 (s, 1H), 3.24-3.16 (m, 2H), 3.11-2.99 (m, 2H); **^{13}C NMR (126 MHz, DMSO)** δ 173.4, 151.7, 138.8, 134.2, 132.1, 130.4, 128.8, 128.6, 128.3, 128.2, 127.0, 126.8, 126.6, 126.0, 122.9, 34.8, 16.2; **HRMS (ESI): calcd. for $C_{21}H_{17}BF_3KO_2$ $[M+Na]^+$:** 431.0808, found 431.0810.

6.4 Suzuki coupling of the **3aa**[3]

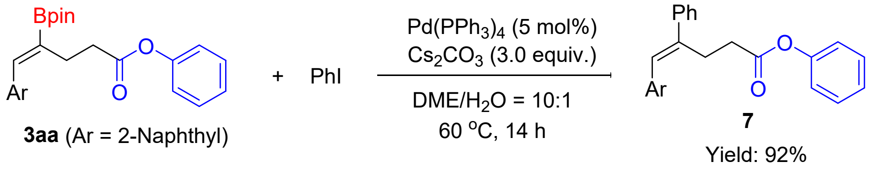

7 was prepared according to a modified procedure. A flame-dried Schlenk was loaded with $Pd(PPh_3)_4$ (0.005 mmol, 5.8 mg), iodobenzene (0.13 mmol, 20 µl) and Cs_2CO_3 (0.3 mmol, 97.8 mg). After 3 vacuum/argon cycles, dried and degassed DME or Dioxane (0.3 mL) was added, followed by vinylboron 3aa (0.1 mmol, 42.8 mg) in solution in DME (0.2 mL), then, the water (60µl) was added followed. The reaction mixture was stirred at 60 °C for the 12 h. After dilution with EtOAc, the reaction mixture was filtrated on silica (EtOAc) and concentrated under reduced pressure. After a flash column chromatography (*n*-hexane/EtOAc 35:1) afforded the product **7** (58 mg, 76% yield) as a colorless oil.

^{1}H NMR (500 MHz, $CDCl_3$) δ 7.87 (dd, J = 12.0, 5.0 Hz, 4H), 7.57 (d, J = 7.2 Hz, 2H), 7.55-7.49 (m, 3H), 7.45 (t, J = 7.6 Hz, 2H), 7.40-7.32 (m, 3H), 7.22 (t, J = 7.4 Hz, 1H), 6.99 (dd, J = 9.8, 2.1 Hz, 3H), 3.38-3.22 (m, 2H), 2.76-2.64 (m, 2H); **^{13}C NMR (126 MHz, $CDCl_3$)** δ 171.4, 150.6, 141.9, 141.0, 135.1, 133.4, 132.3, 129.8, 129.3, 128.7, 128.0, 128.0, 127.7, 127.6, 127.4, 127.1, 126.8, 126.2, 125.9, 125.7, 121.5, 33.4, 25.7; **HRMS (ESI)**: calcd. for $C_{27}H_{22}O_2$ $[M+Na]^+$: 401.1517, found 401.1522.

7. References

(1) J. Yang, Y.-Y. Liu, R.-J. Song, Z.-H. Peng, J.-H. Li, *Adv. Synth. Catal*. **2016**, *358*, 2286–2292.

(2) Z.-Z. Zhu, K. Chen, L.-Z. Yu, X.-Y. Tang, M. Shi, *Org. Lett*. **2015**, *17*, 5994–5997.

(3) J. Han, W. Zhou, P.-C. Zhang, H. Wang, H.-H. Wu, H. Zhang, *ACS Catal*. **2019**, 9, 6890–6895.

8. Copy of ^{1}H, and ^{13}CNMR Spectra of Products

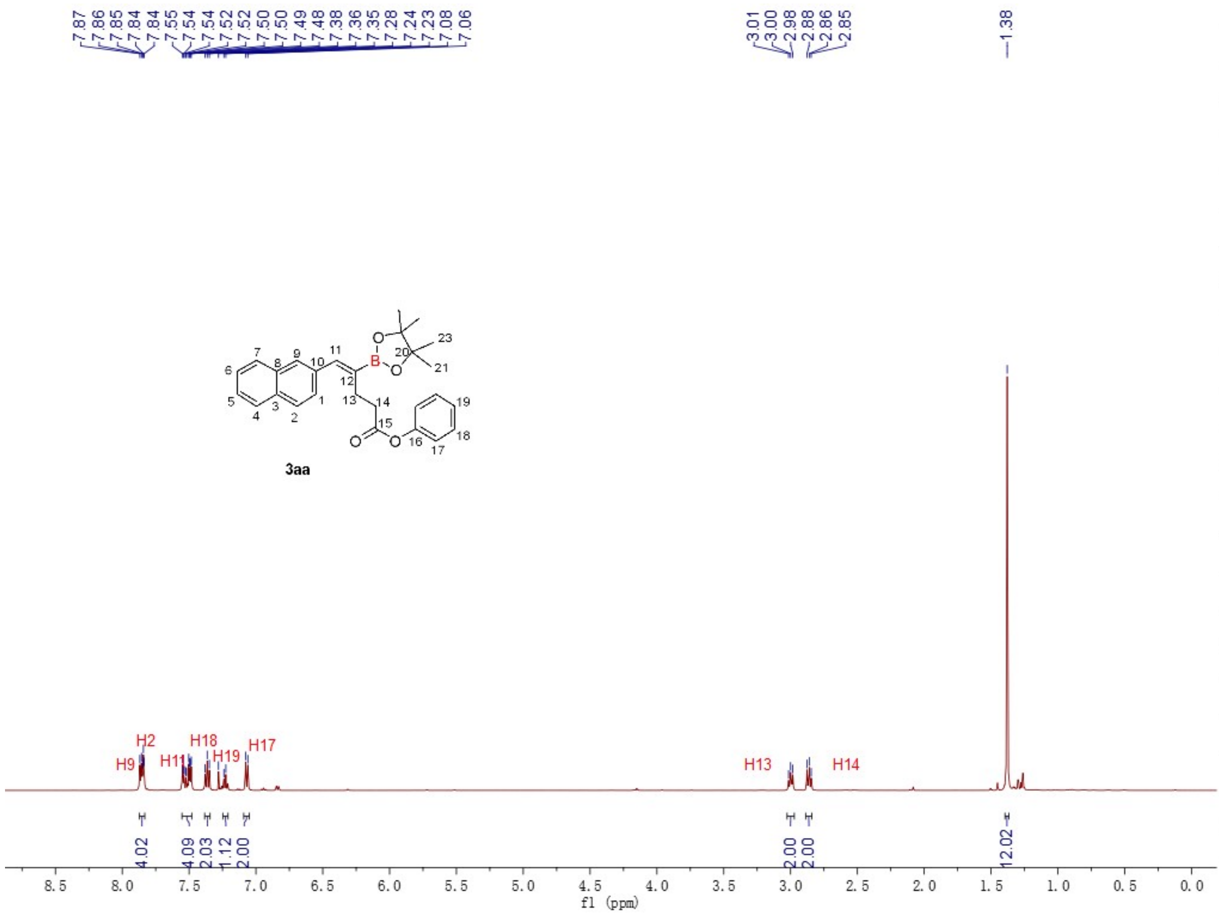
7.87 7.86 7.85 7.84 7.84 7.55 7.54 7.54 7.52 7.52 7.50 7.49 7.48 7.38 7.36 7.35 7.28 7.24 7.23 7.08 7.06
3.01 3.00 2.98 2.88 2.86 2.85
1.38
3aa
H9
H2
H11
H18
H19
H17
H13
H14
4.02 4.09 2.03 1.12 2.00
2.00 2.00
12.02
f1 (ppm)

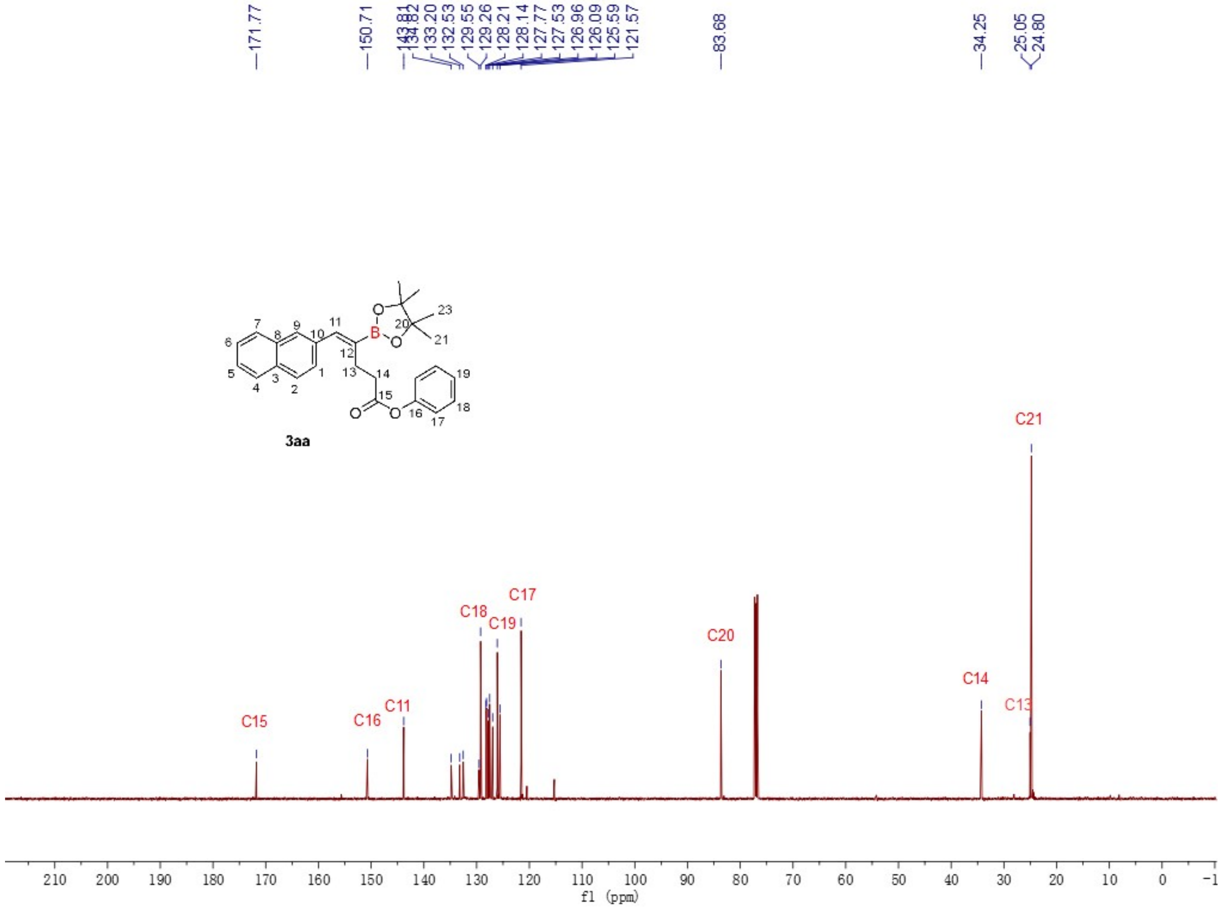
171.77
150.71
143.81 134.82 133.20 132.53 129.55 129.26 128.21 128.14 127.77 127.53 126.96 126.09 125.59 121.57
83.68
34.25
25.05 24.80
3aa
C15
C16
C11
C18
C19
C17
C20
C14
C13
C21
f1 (ppm)

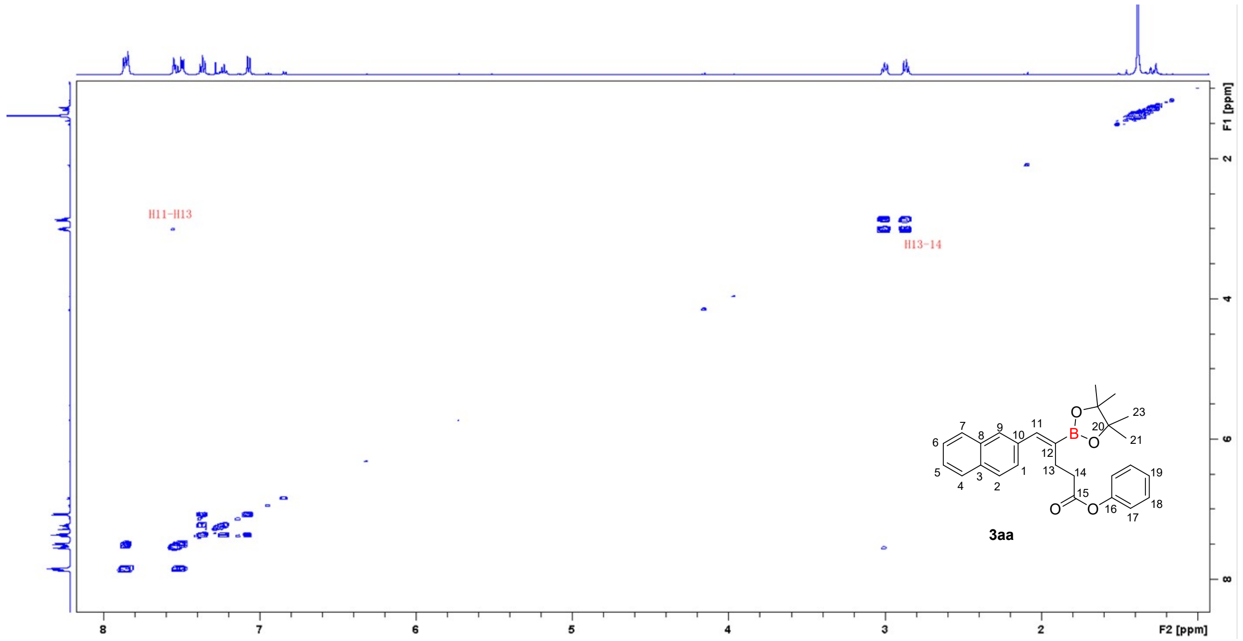

H11-H13
H13-14
3aa
F1 [ppm]
F2 [ppm]

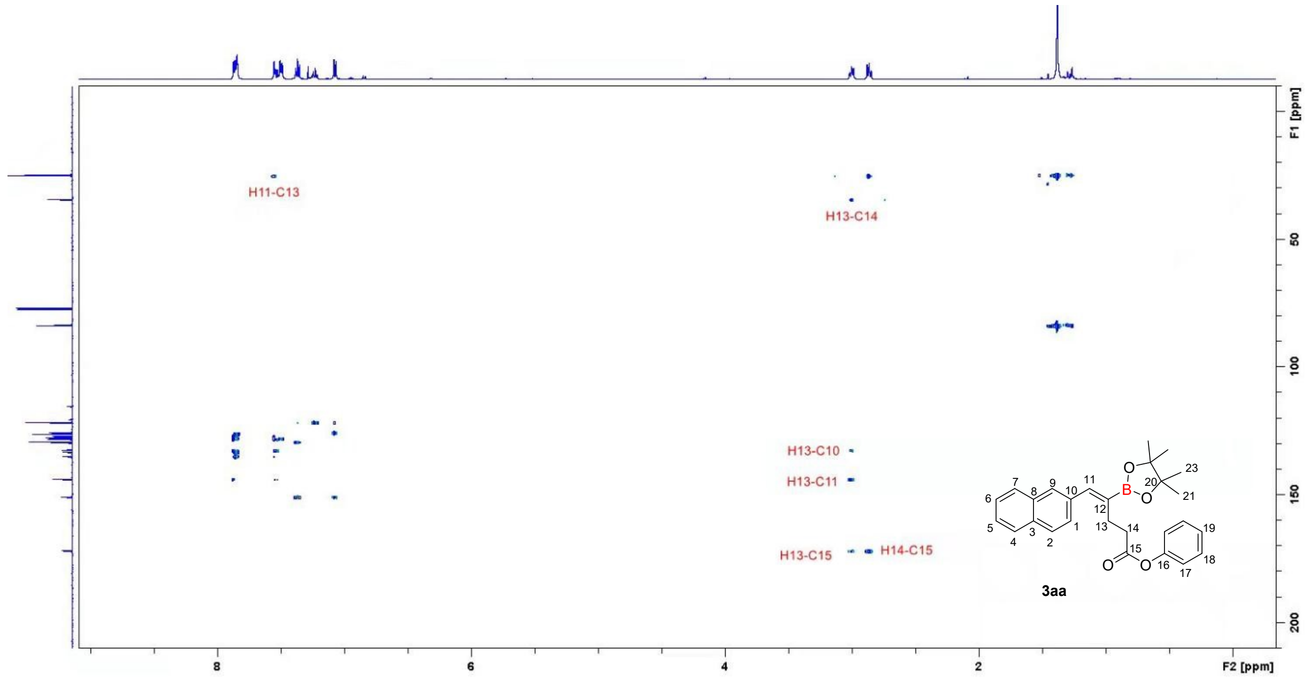

H11-C13
H13-C14
H13-C10
H13-C11
H13-C15
H14-C15
3aa
F1 [ppm]
F2 [ppm]

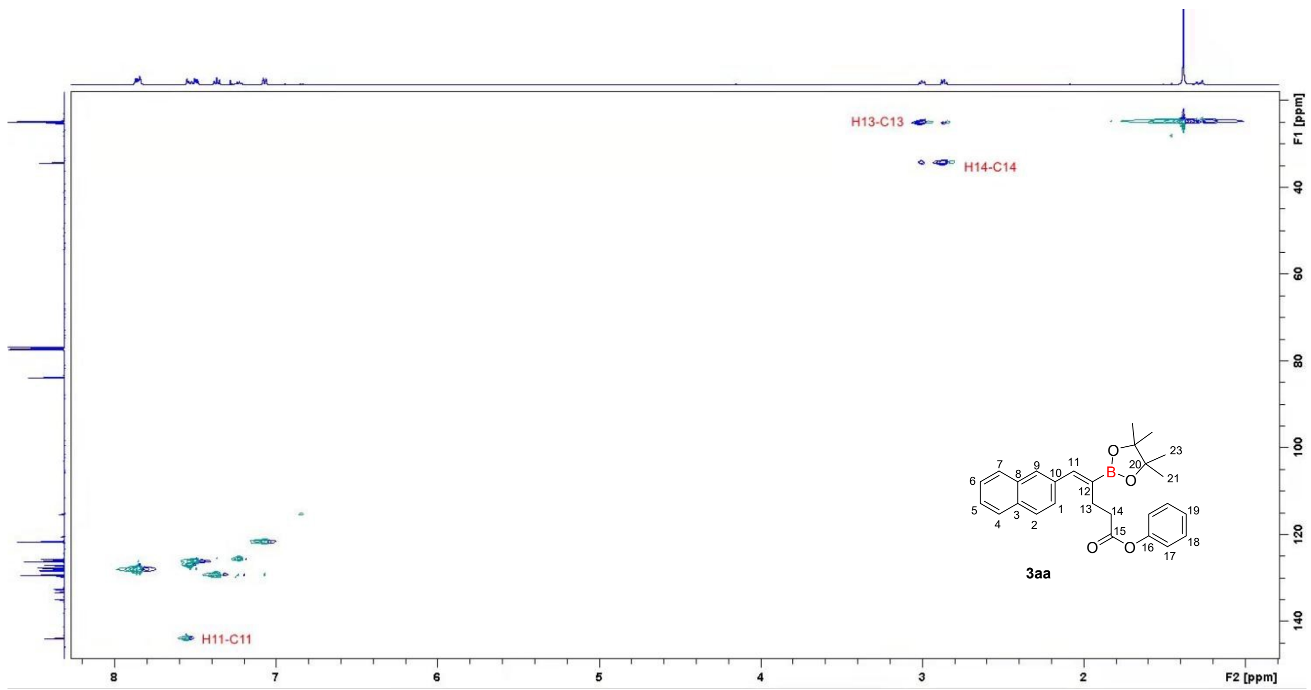

H13-C13
H14-C14
H11-C11
3aa
F1 [ppm]
F2 [ppm]

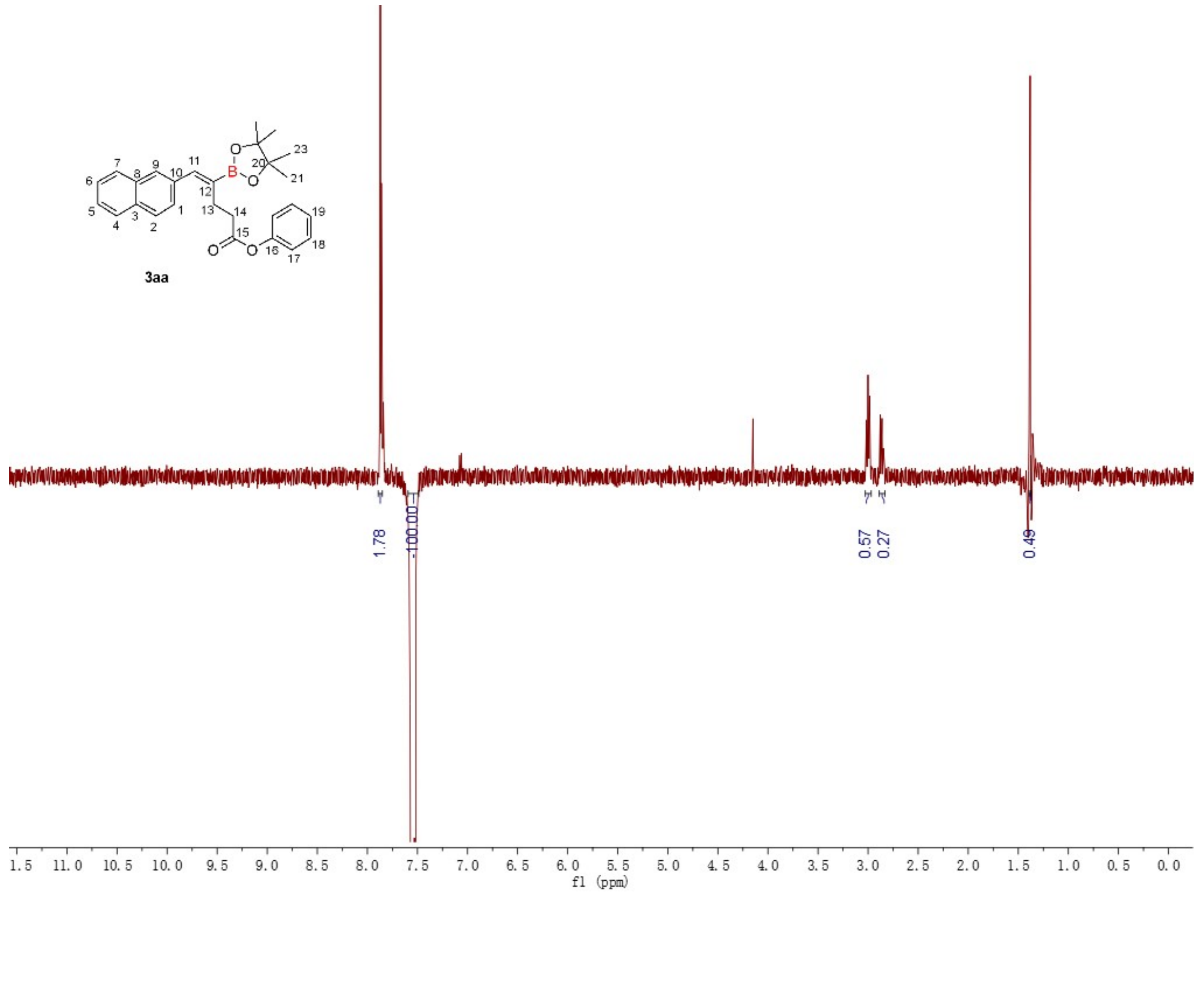

3aa
1.78
-100.00
0.57
0.27
0.49
f1 (ppm)

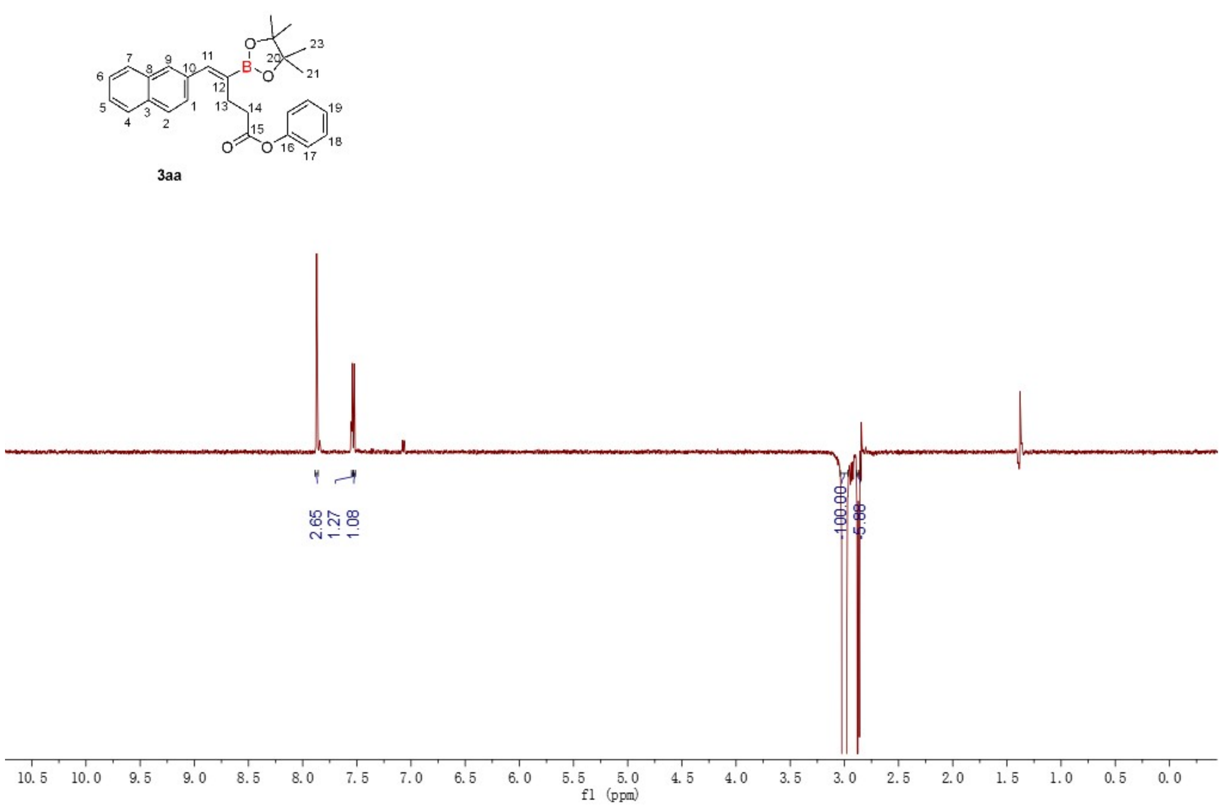
3aa
2.65
1.27
1.08
-100.00
-5.88
fl (ppm)

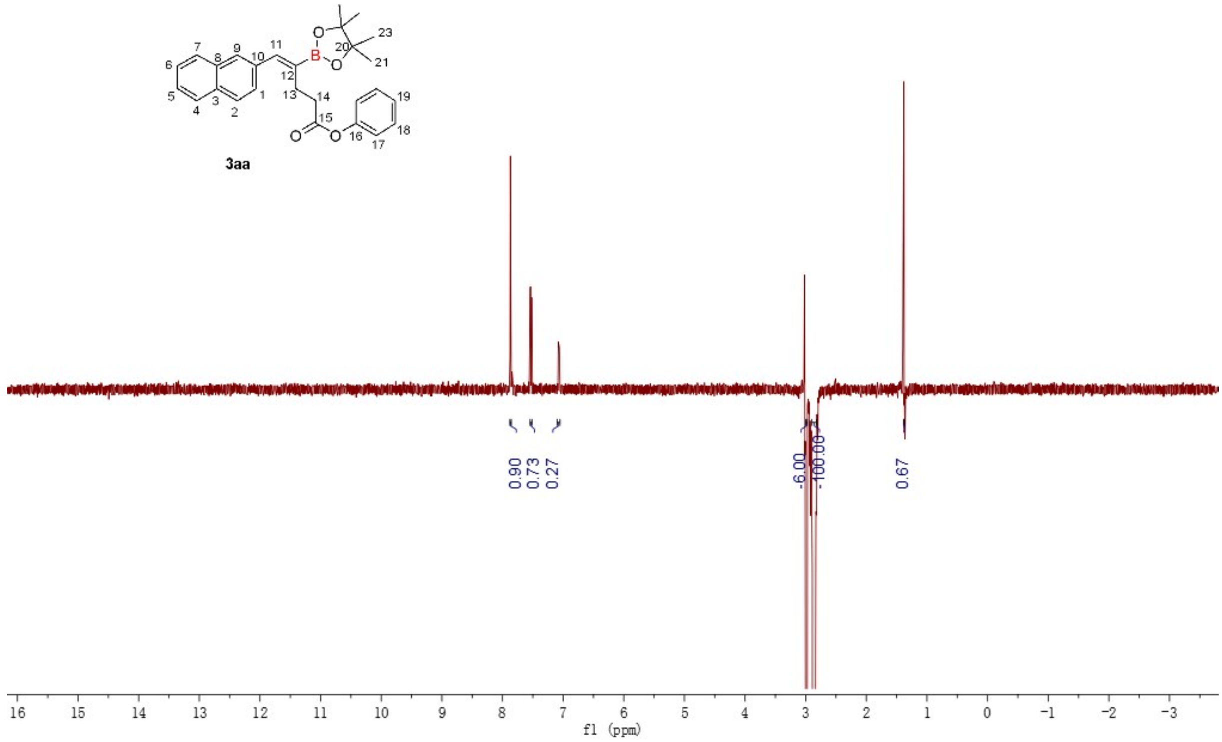
3aa
0.90
0.73
0.27
-6.00
-100.00
0.67
fl (ppm)

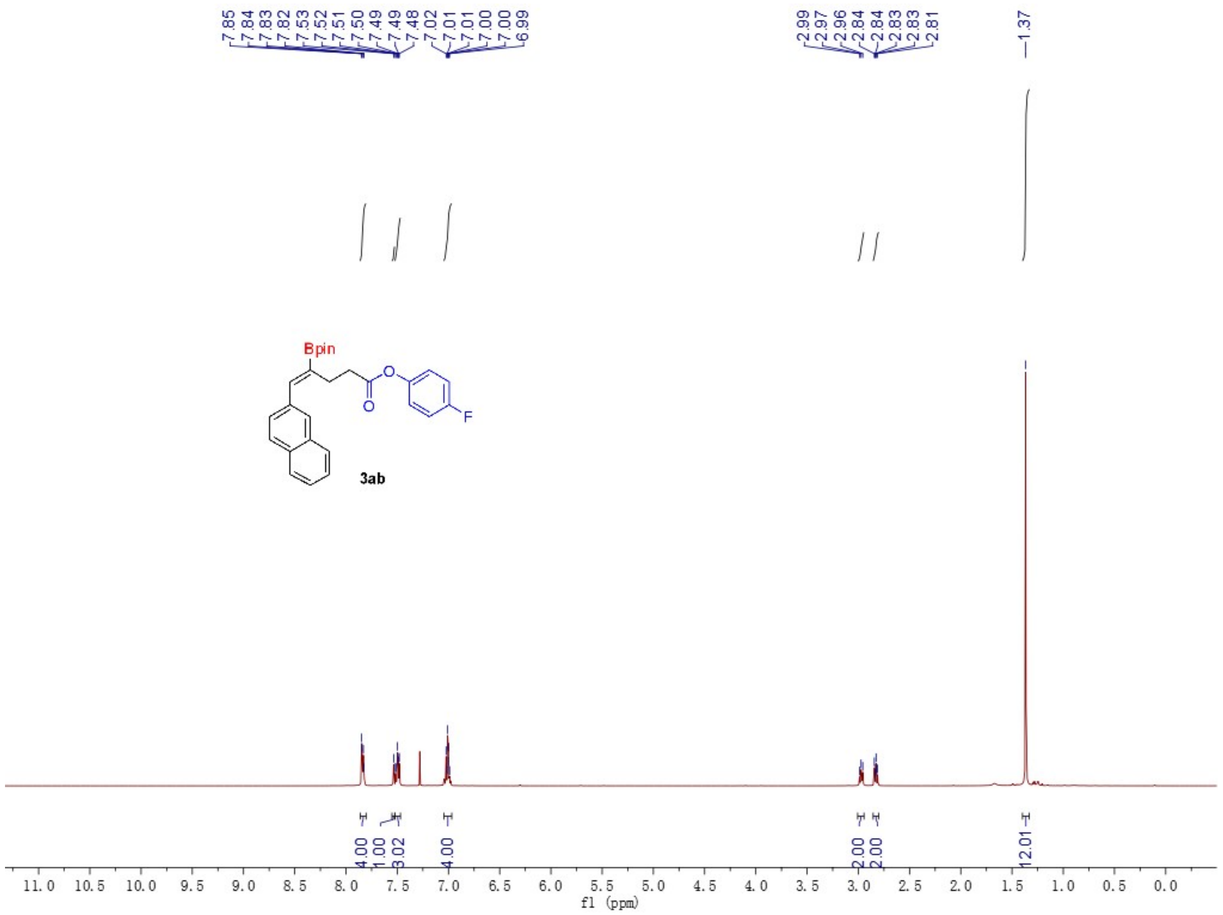
7.85 7.84 7.83 7.82 7.53 7.52 7.51 7.50 7.49 7.49 7.48 7.02 7.01 7.01 7.00 7.00 6.99
2.99 2.97 2.96 2.84 2.83 2.83 2.81
1.37
Bpin
O
F
3ab
4.00 1.00 3.02 4.00 2.00 2.00 12.01
f1 (ppm)

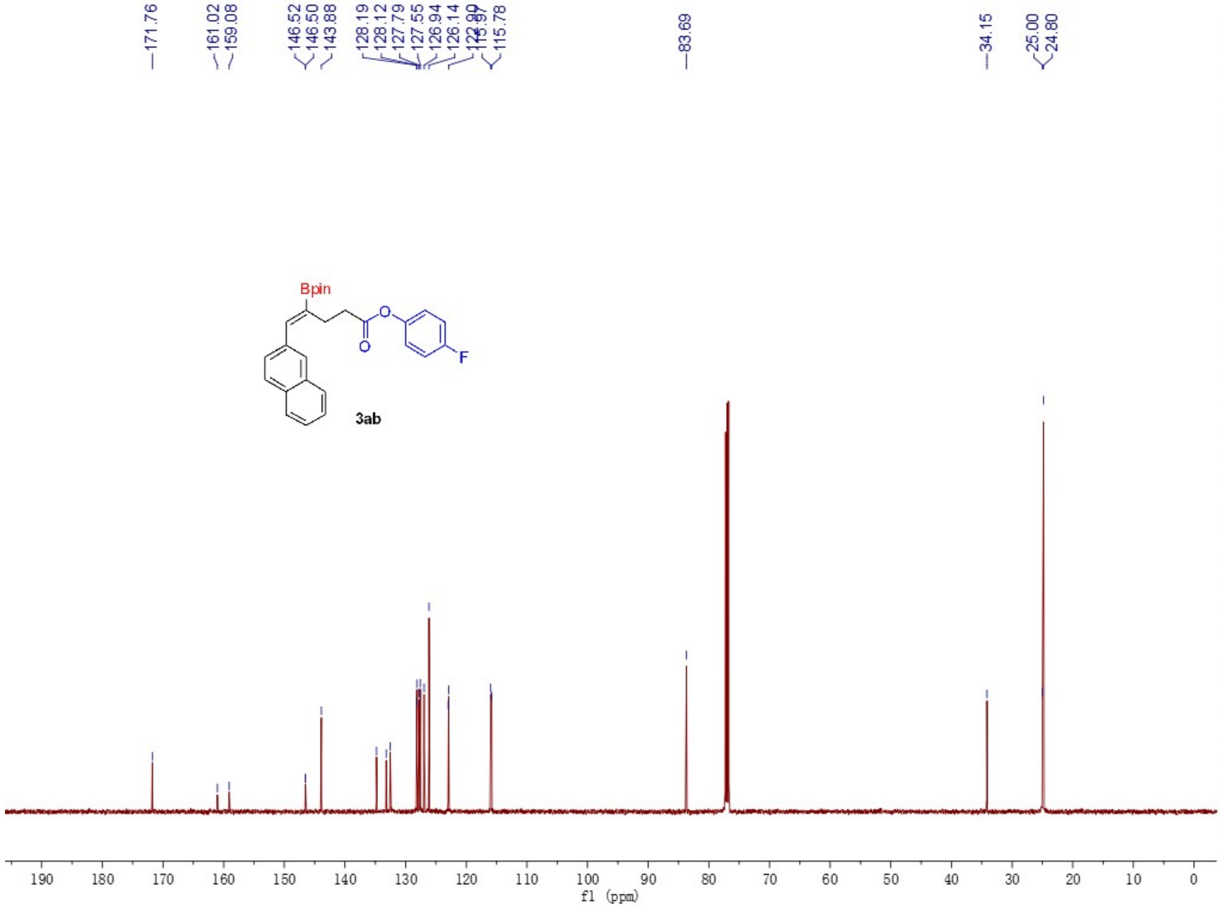
171.76
161.02 159.08
146.52 146.50 143.88
128.19 128.12 127.79 127.55 126.94 126.14
115.78
83.69
34.15
25.00 24.80
Bpin
O
F
3ab
f1 (ppm)

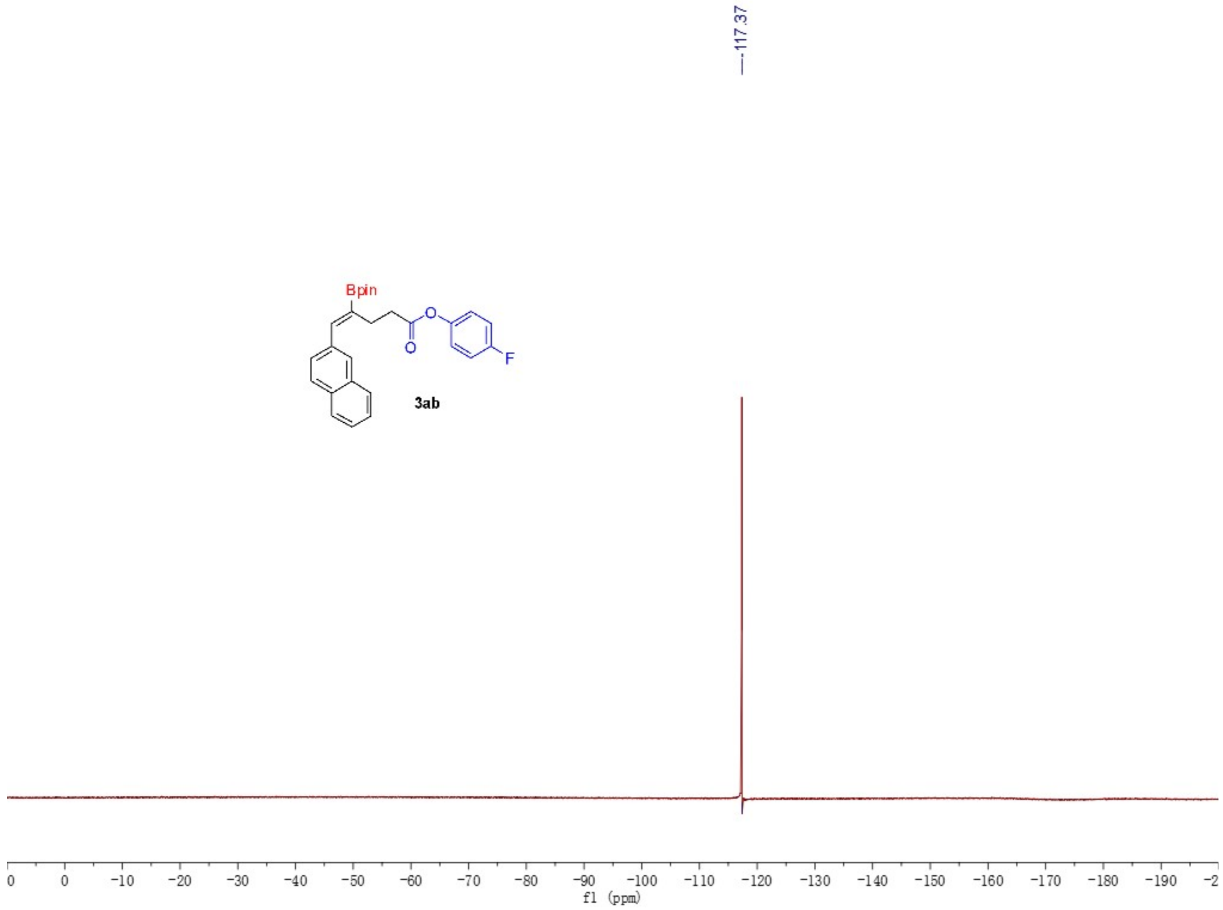
-117.37
Bpin
O
O
F
3ab
0
0
-10
-20
-30
-40
-50
-60
-70
-80
-90
-100
-110
-120
-130
-140
-150
-160
-170
-180
-190
-2
f1 (ppm)

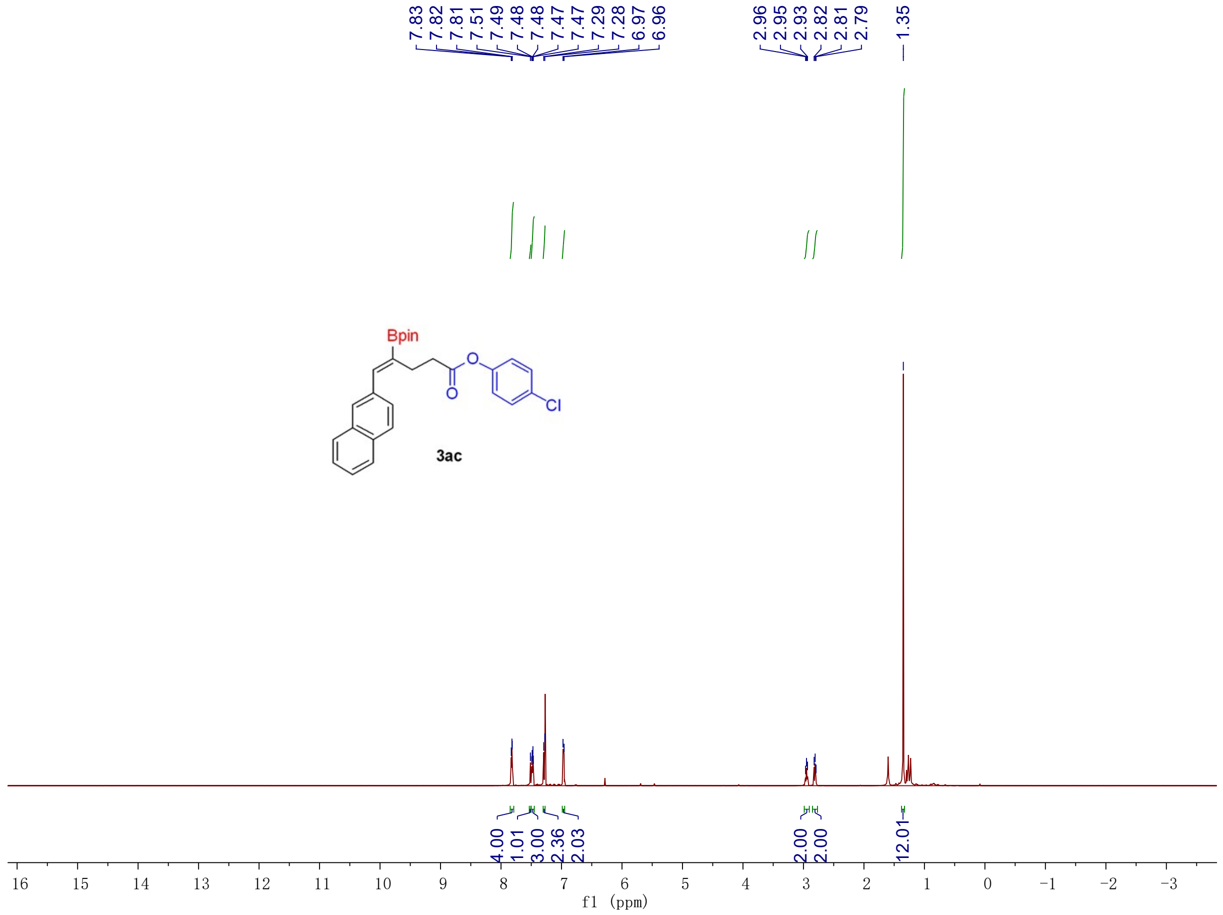
7.83
7.82
7.81
7.51
7.49
7.48
7.48
7.47
7.47
7.29
7.28
6.97
6.96
2.96
2.95
2.93
2.82
2.81
2.79
1.35
Bpin
O
O
Cl
3ac
4.00
1.01
3.00
2.36
2.03
2.00
2.00
12.01
16
15
14
13
12
11
10
9
8
7
6
5
4
3
2
1
0
-1
-2
-3
f1 (ppm)

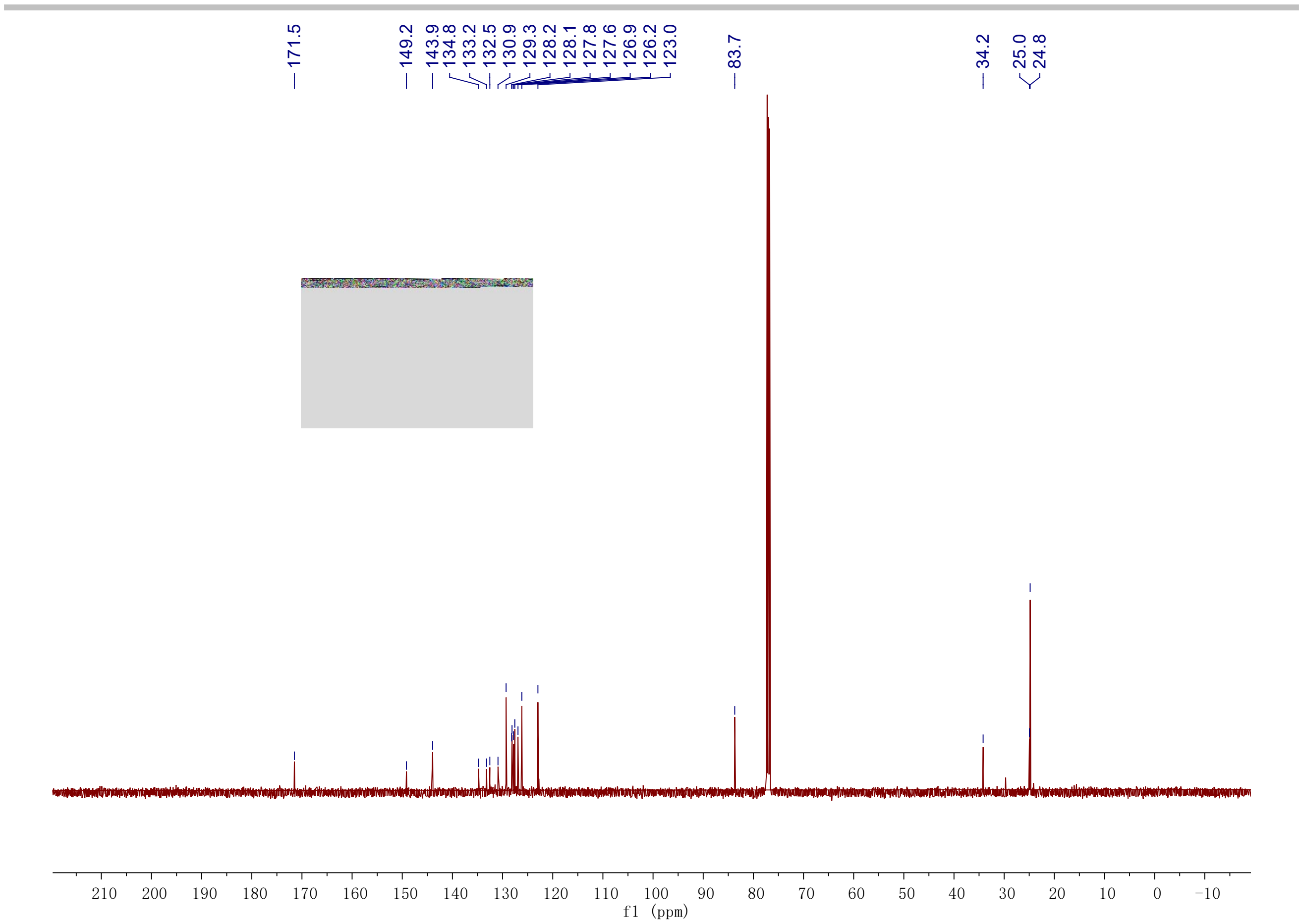
171.5
149.2
143.9
134.8
133.2
132.5
130.9
129.3
128.2
128.1
127.8
127.6
126.9
126.2
123.0
83.7
34.2
25.0
24.8
210 200 190 180 170 160 150 140 130 120 110 100 90 80 70 60 50 40 30 20 10 0 -10
f1 (ppm)

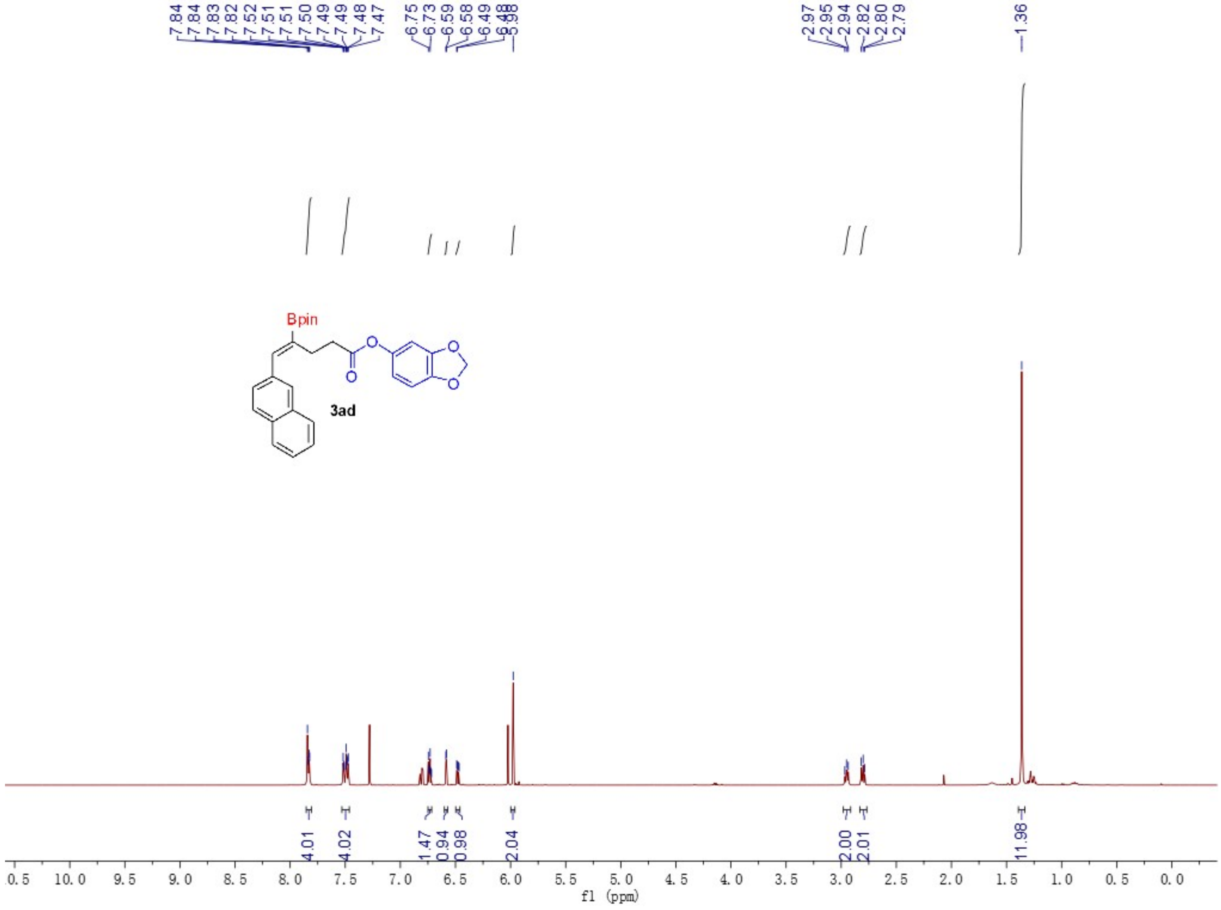
7.84
7.84
7.83
7.82
7.52
7.51
7.51
7.50
7.49
7.49
7.48
7.47
6.75
6.73
6.59
6.58
6.49
6.48
5.98
2.97
2.95
2.94
2.82
2.80
2.79
1.36
Bpin
3ad
4.01
4.02
1.47
0.94
0.98
2.04
2.00
2.01
11.98
0.5 10.0 9.5 9.0 8.5 8.0 7.5 7.0 6.5 6.0 5.5 5.0 4.5 4.0 3.5 3.0 2.5 2.0 1.5 1.0 0.5 0.0
f1 (ppm)

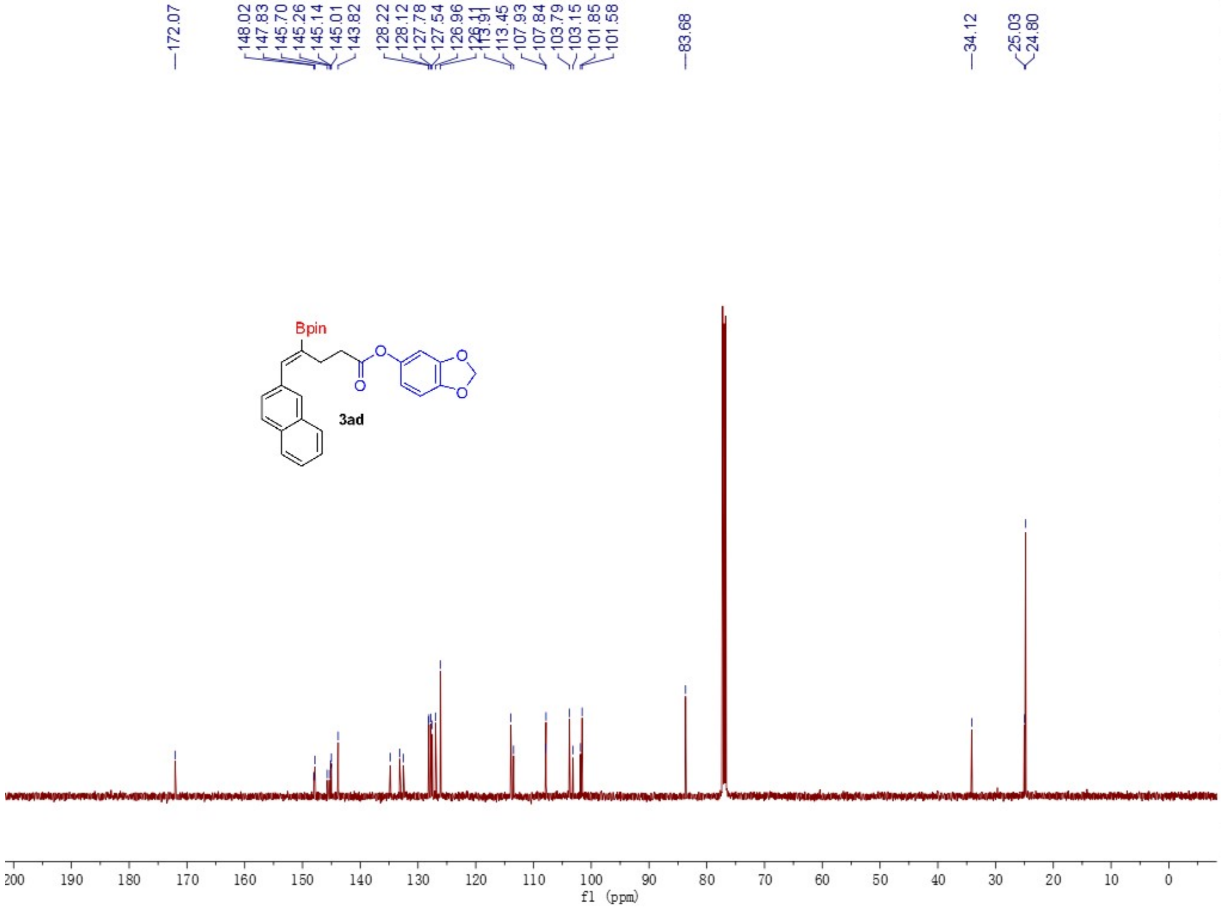
172.07
148.02
147.83
145.70
145.26
145.14
145.01
143.82
128.22
128.12
127.78
127.54
126.96
126.11
113.91
113.45
107.93
107.84
103.79
103.15
101.85
101.58
83.68
34.12
25.03
24.80
Bpin
O
O
O
O
3ad
fl (ppm)

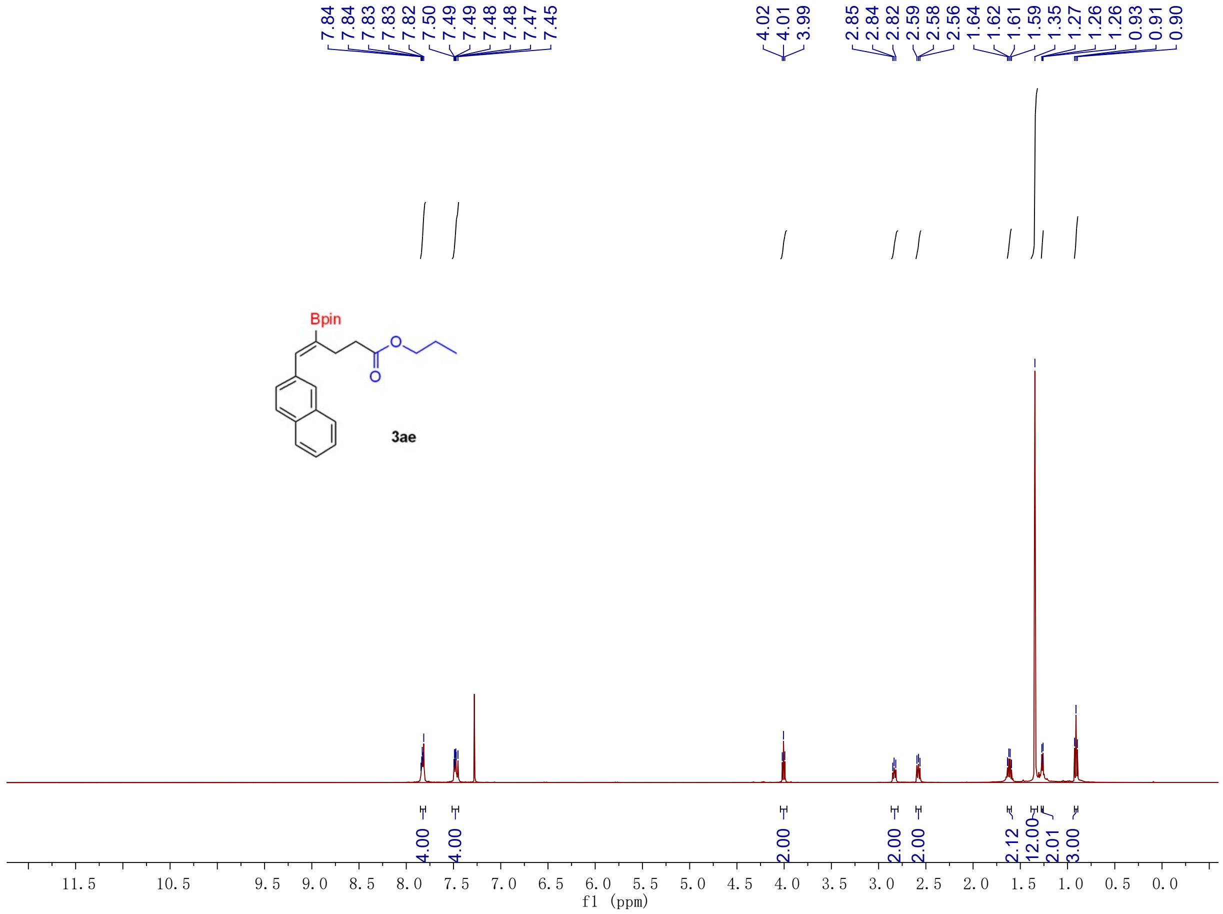
7.84
7.84
7.83
7.83
7.82
7.50
7.49
7.49
7.48
7.48
7.47
7.45
4.02
4.01
3.99
2.85
2.84
2.82
2.59
2.58
2.56
1.64
1.62
1.61
1.59
1.35
1.27
1.26
0.93
0.91
0.90
Bpin
O
O
3ae
4.00
4.00
2.00
2.00
2.00
2.12
12.00
2.01
3.00
fl (ppm)

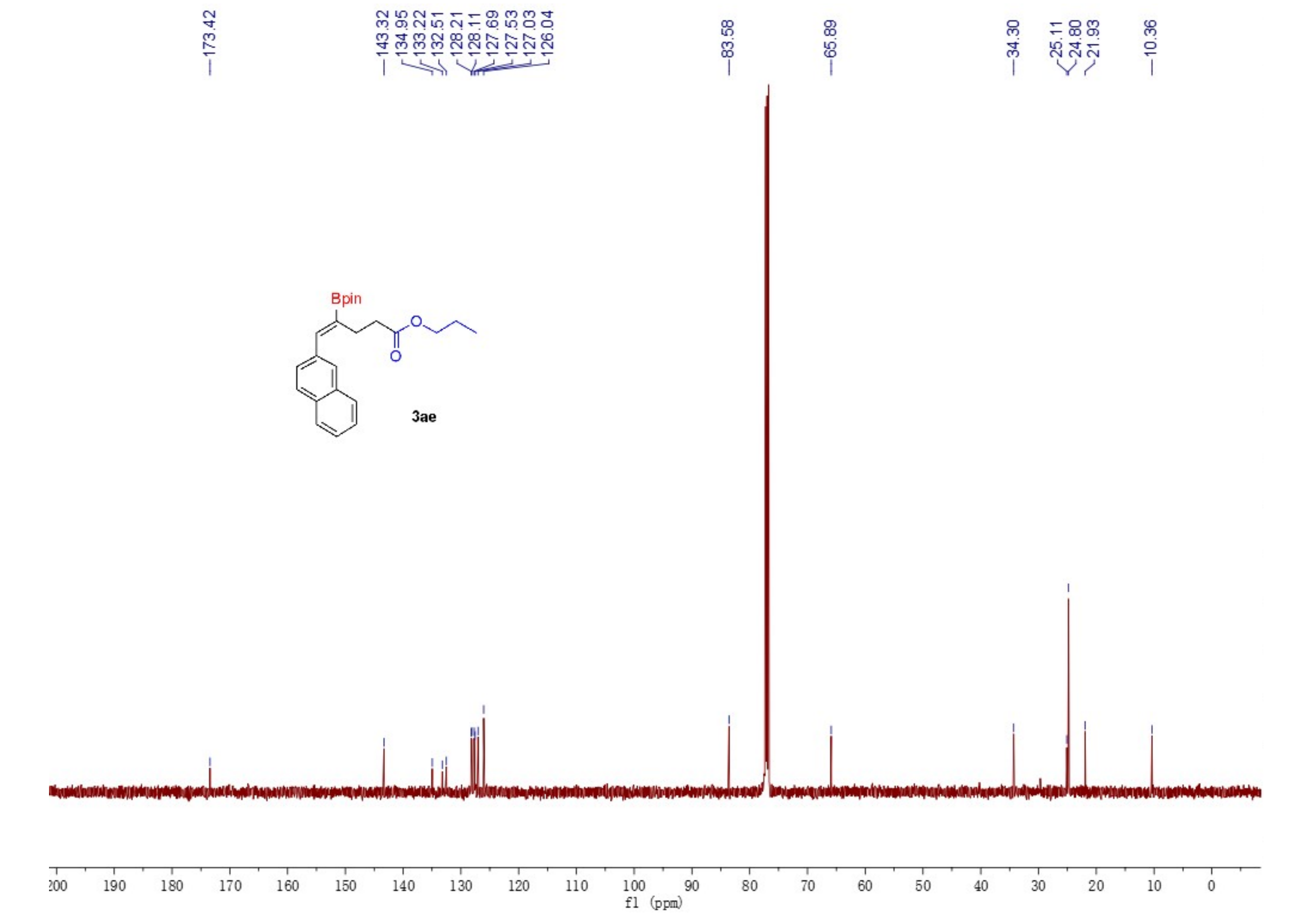
173.42
143.32
134.95
133.22
132.51
128.21
128.11
127.69
127.53
127.03
126.04
83.58
65.89
34.30
25.11
24.80
21.93
10.36
Bpin
O
O
3ae
200 190 180 170 160 150 140 130 120 110 100 90 80 70 60 50 40 30 20 10 0
f1 (ppm)

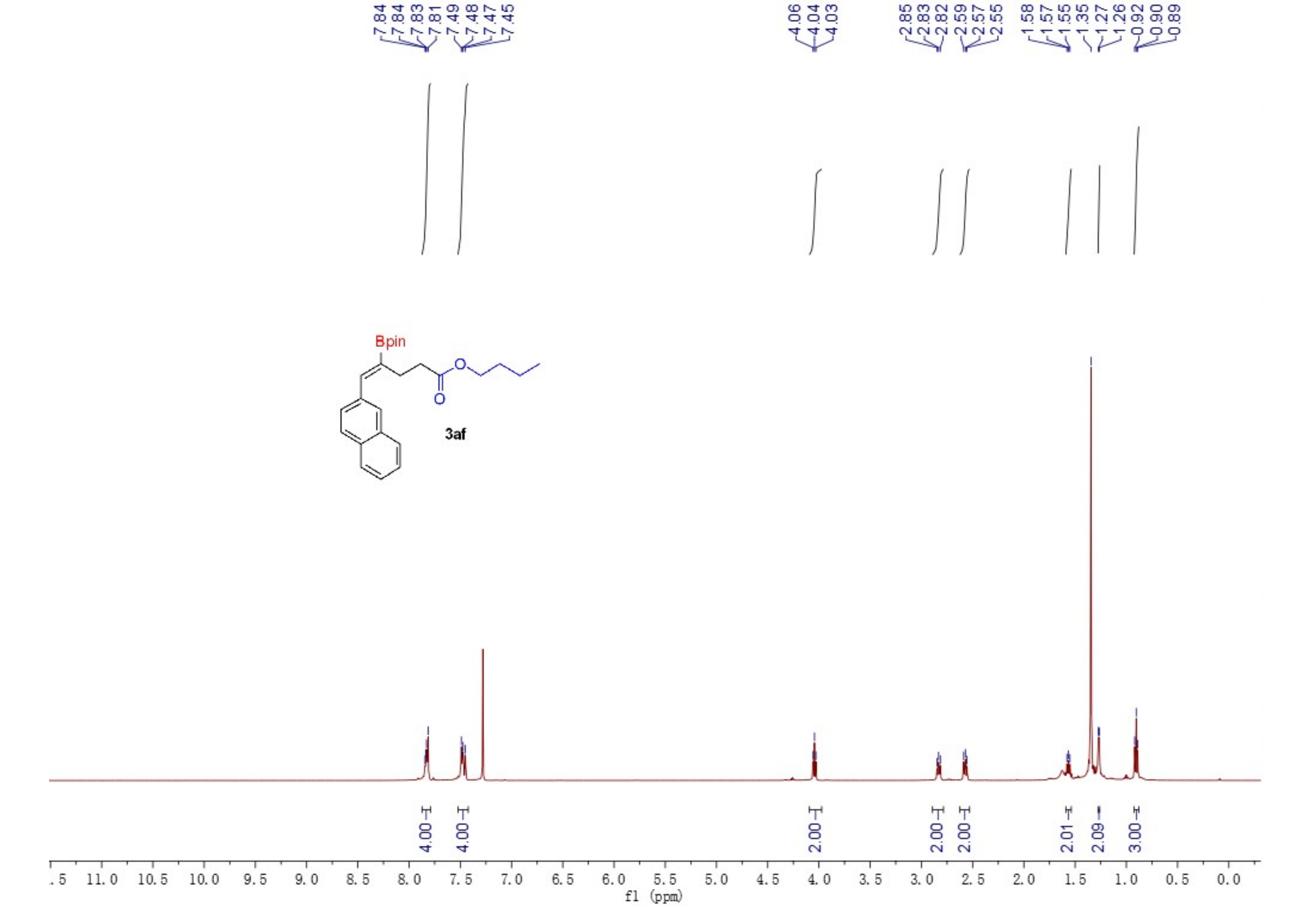
7.84
7.84
7.83
7.81
7.49
7.48
7.47
7.45
4.06
4.04
4.03
2.85
2.83
2.82
2.59
2.57
2.55
1.58
1.57
1.55
1.35
1.27
1.26
0.92
0.90
0.89
Bpin
O
O
3af
4.00
4.00
2.00
2.00
2.00
2.01
2.09
3.00
11.0 10.5 10.0 9.5 9.0 8.5 8.0 7.5 7.0 6.5 6.0 5.5 5.0 4.5 4.0 3.5 3.0 2.5 2.0 1.5 1.0 0.5 0.0
f1 (ppm)

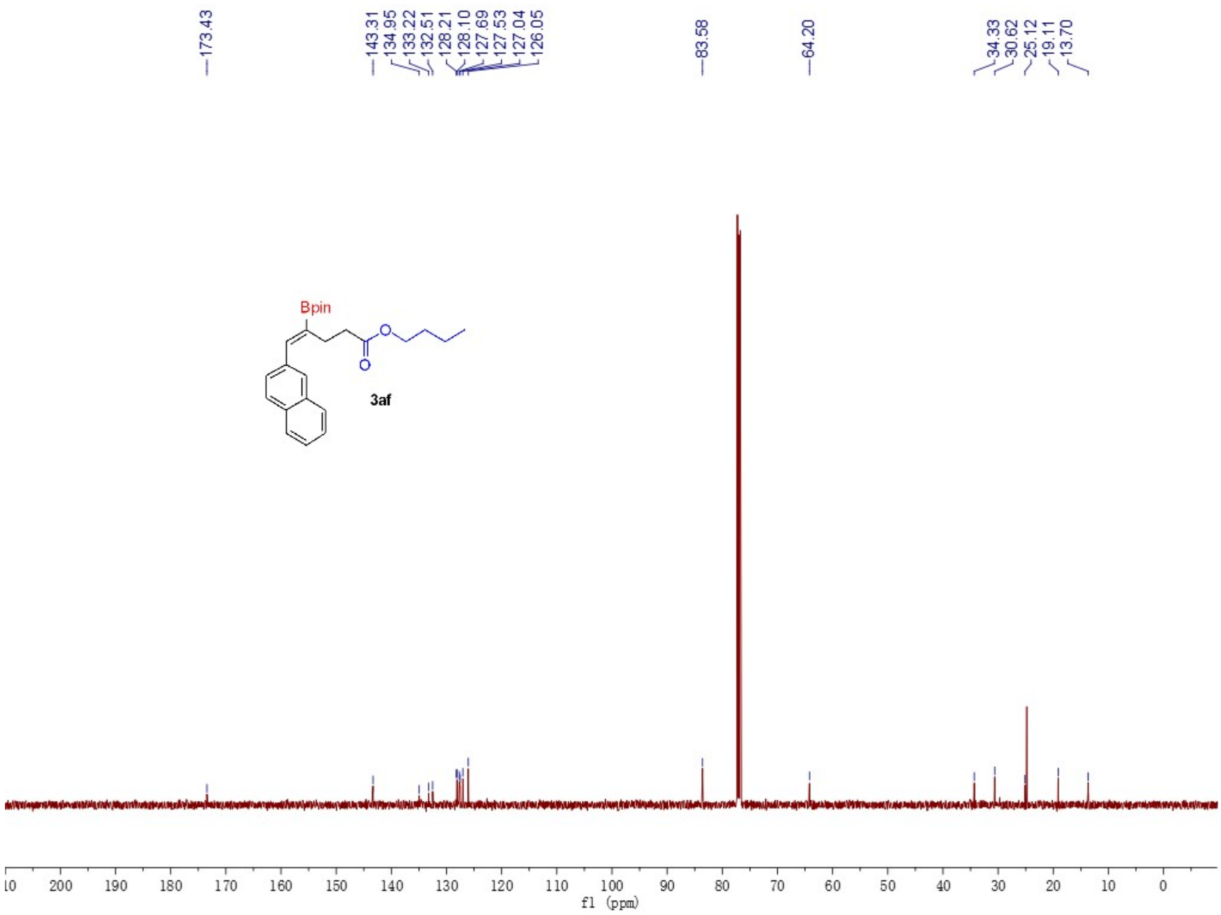
173.43
143.31
134.95
133.22
132.51
128.21
128.10
127.69
127.53
127.04
126.05
83.58
64.20
34.33
30.62
25.12
19.11
13.70
Bpin
O
O
3af
f1 (ppm)

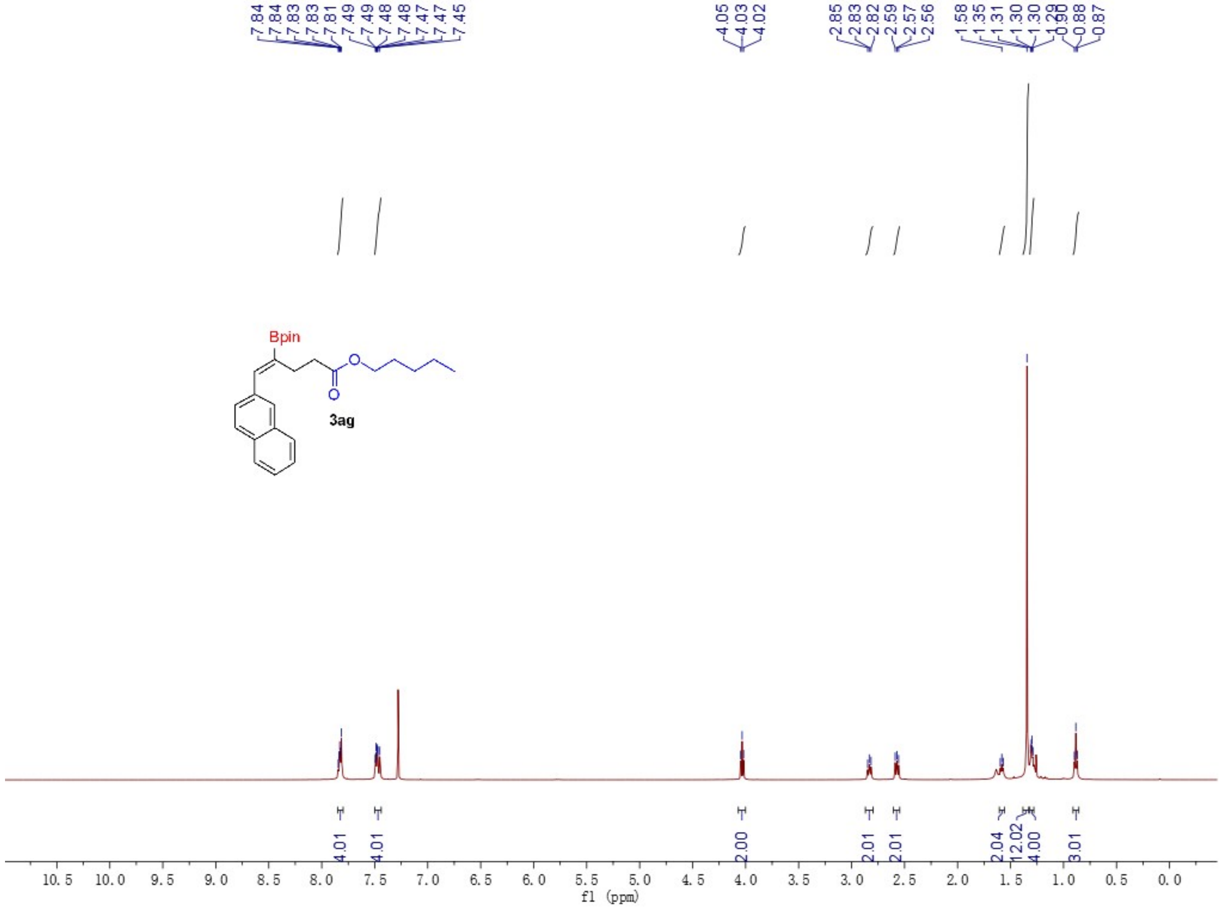
7.84
7.84
7.83
7.83
7.81
7.49
7.49
7.48
7.47
7.47
7.45
4.05
4.03
4.02
2.85
2.83
2.82
2.59
2.57
2.56
1.58
1.35
1.31
1.30
1.29
0.90
0.88
0.87
Bpin
O
O
3ag
4.01
4.01
2.00
2.01
2.01
2.04
12.02
4.00
3.01
f1 (ppm)

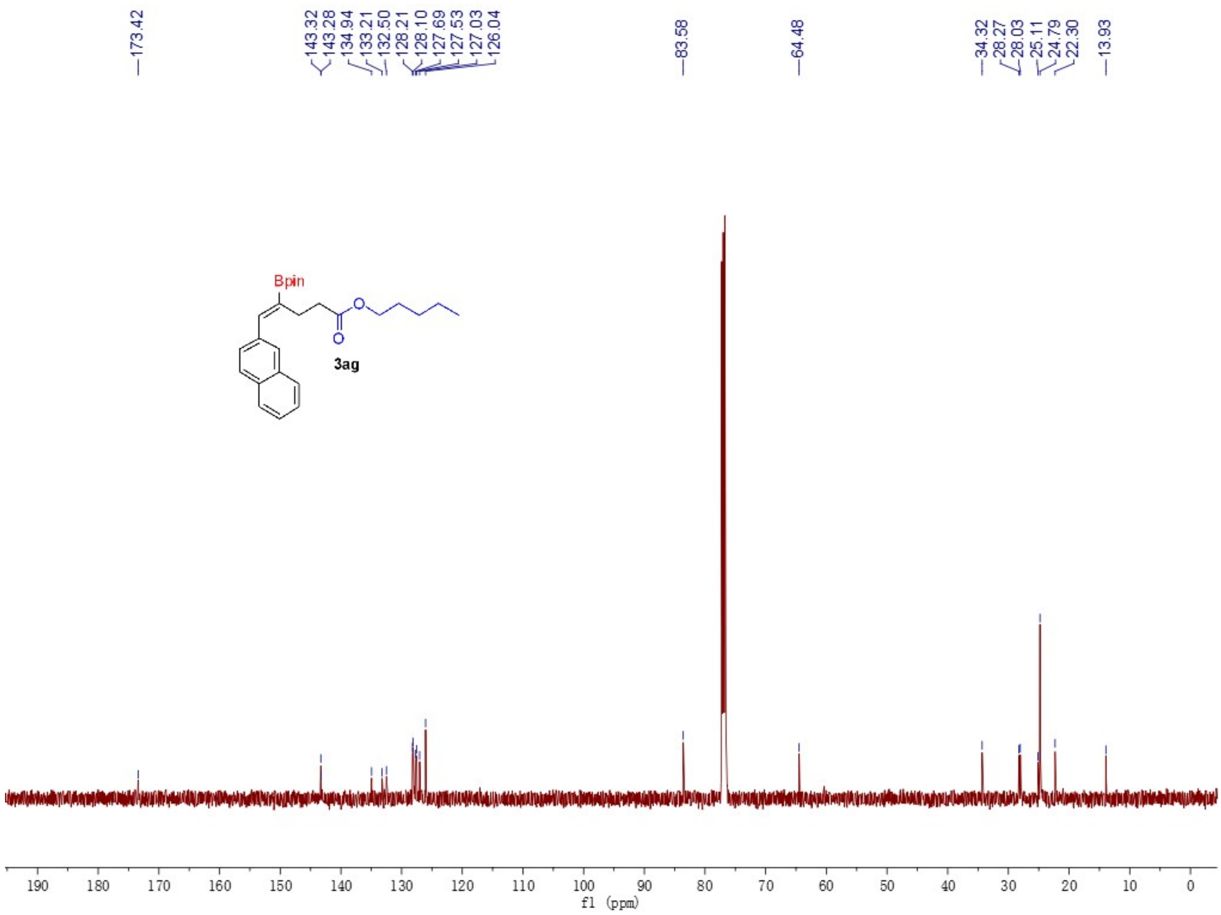
173.42
143.32
143.28
134.94
133.21
132.50
128.21
128.10
127.69
127.53
127.03
126.04
83.58
64.48
34.32
28.27
28.03
25.11
24.79
22.30
13.93
Bpin
O
O
3ag
f1 (ppm)

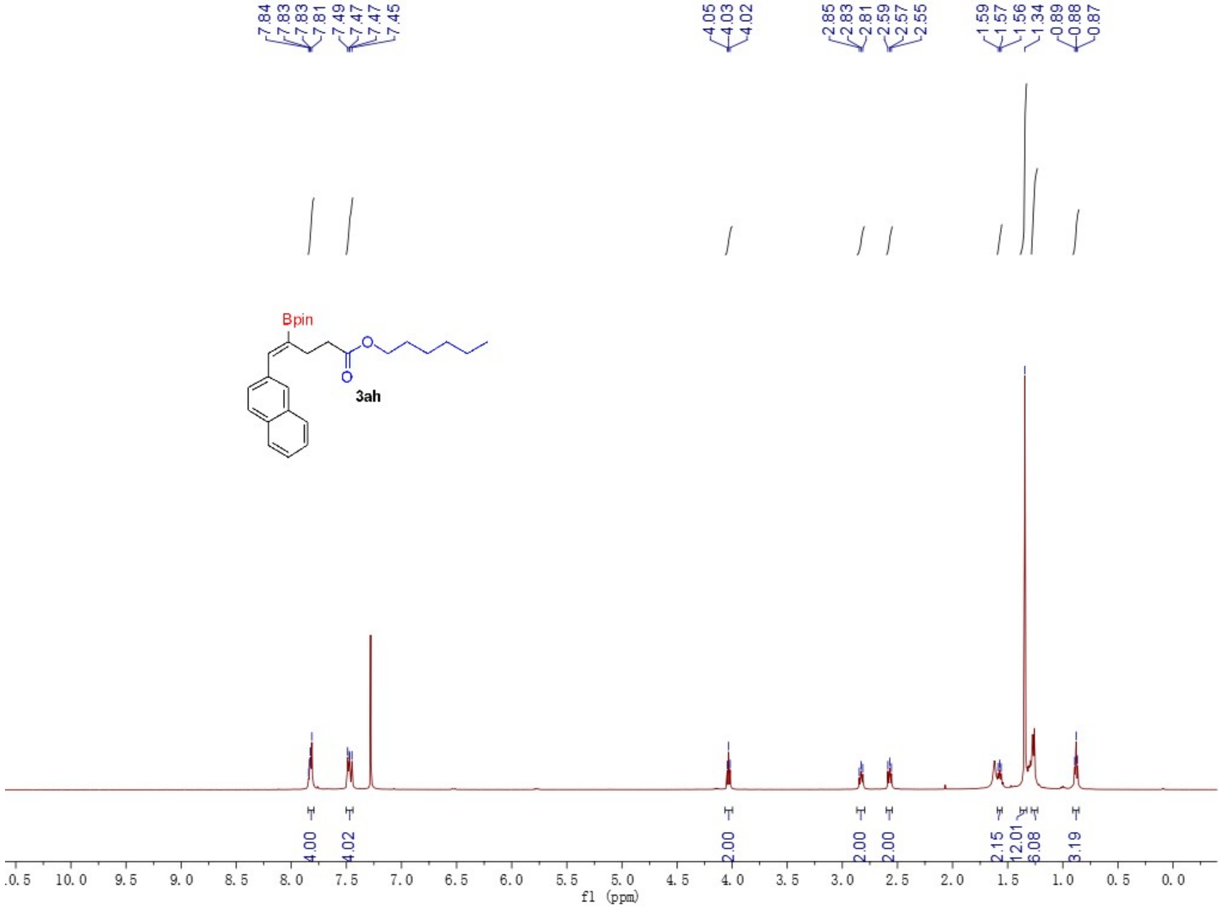
7.84
7.83
7.83
7.81
7.49
7.47
7.47
7.45
4.05
4.03
4.02
2.85
2.83
2.81
2.59
2.57
2.55
1.59
1.57
1.56
1.34
0.89
0.88
0.87
Bpin
O
O
3ah
4.00
4.02
2.00
2.00
2.00
2.15
12.01
6.08
3.19
f1 (ppm)

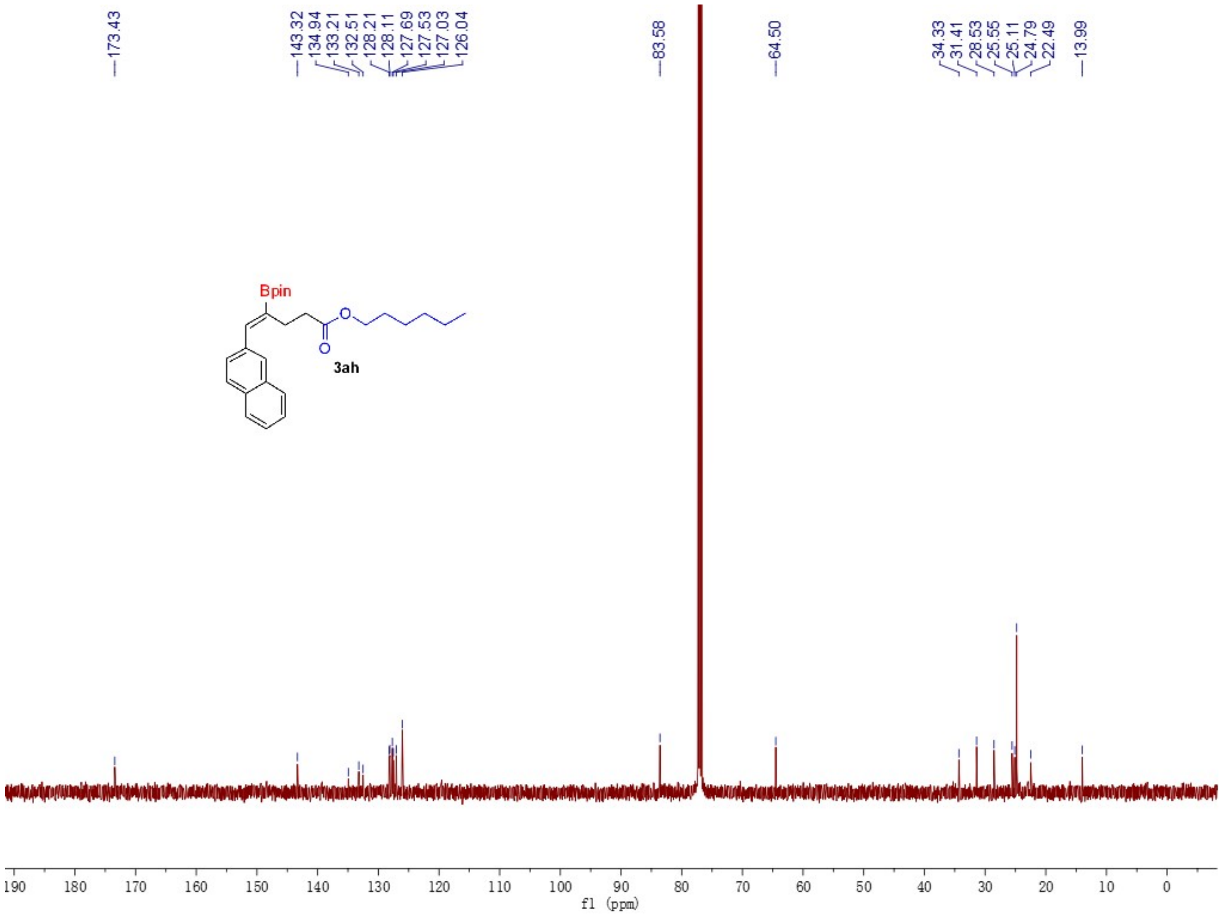
173.43
143.32
134.94
133.21
132.51
128.21
128.11
127.69
127.53
127.03
126.04
83.58
64.50
34.33
31.41
28.53
25.55
25.11
24.79
22.49
13.99
Bpin
O
O
3ah
190 180 170 160 150 140 130 120 110 100 90 80 70 60 50 40 30 20 10 0
f1 (ppm)

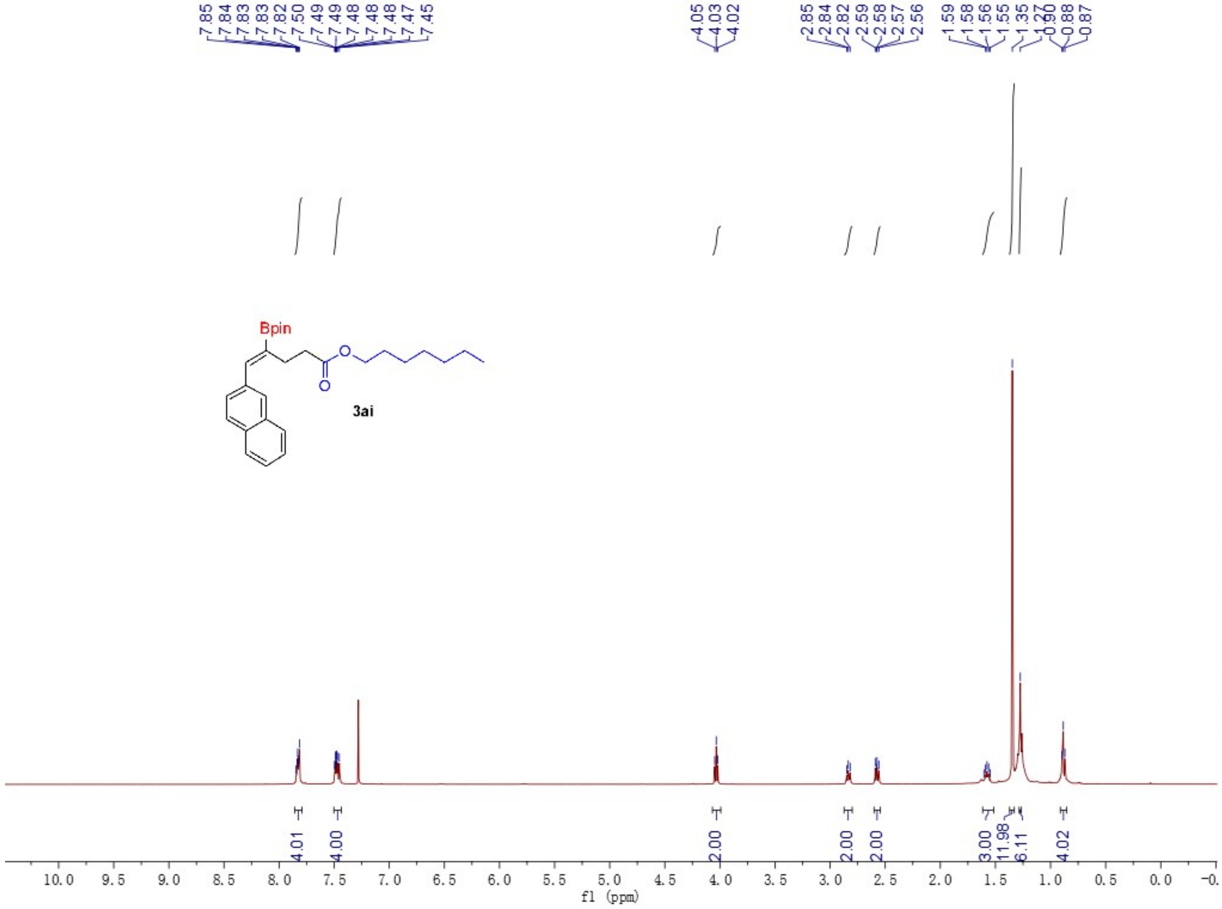
7.85
7.84
7.83
7.82
7.50
7.49
7.48
7.48
7.47
7.45
4.05
4.03
4.02
2.85
2.84
2.82
2.59
2.58
2.57
2.56
1.59
1.58
1.56
1.55
1.35
1.27
0.90
0.88
0.87
Bpin
O
O
3ai
4.01
4.00
2.00
2.00
2.00
3.00
11.98
6.11
4.02
10.0 9.5 9.0 8.5 8.0 7.5 7.0 6.5 6.0 5.5 5.0 4.5 4.0 3.5 3.0 2.5 2.0 1.5 1.0 0.5 0.0
f1 (ppm)

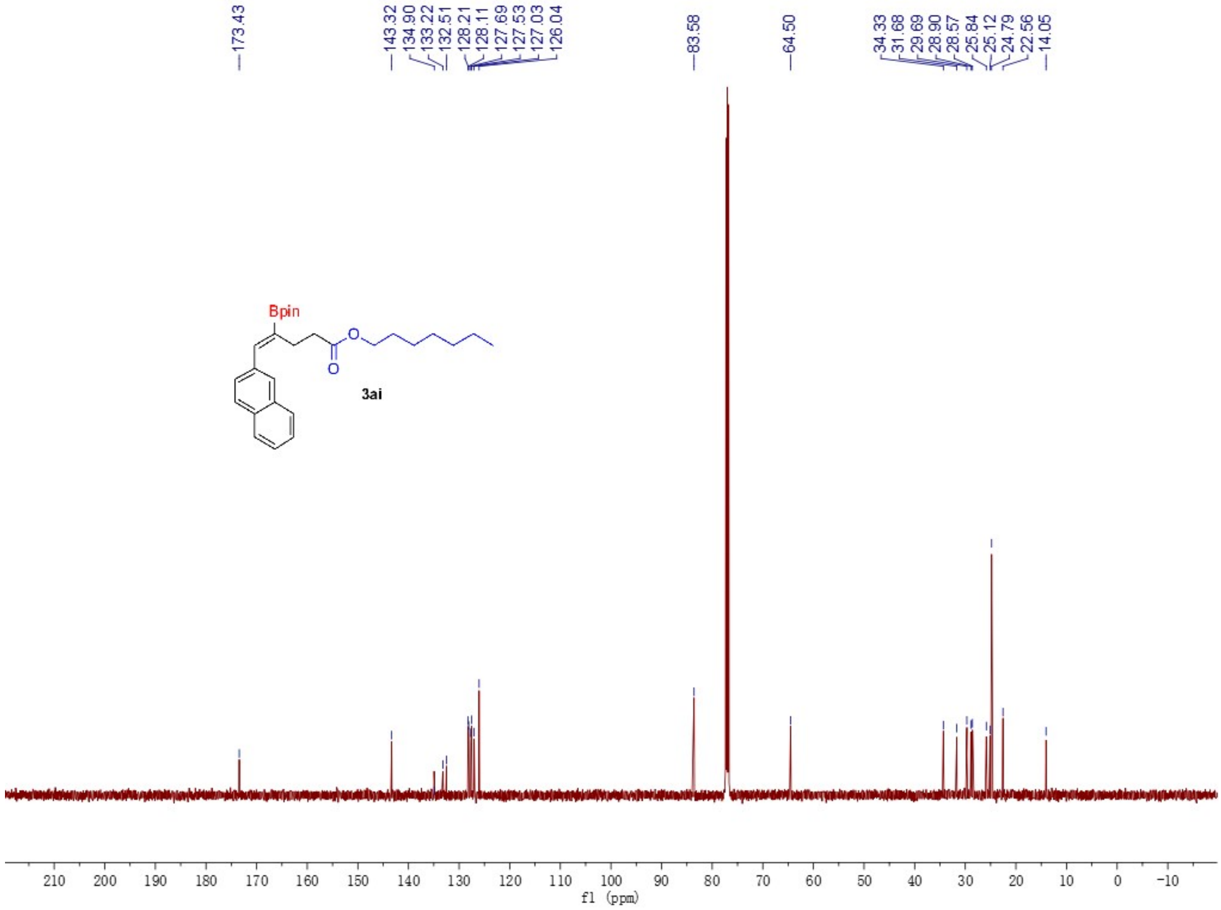
173.43
143.32
134.90
133.22
132.51
128.21
128.11
127.69
127.53
127.03
126.04
83.58
64.50
34.33
31.68
29.69
28.90
28.57
25.84
25.12
24.79
22.56
14.05
Bpin
O
3ai
f1 (ppm)

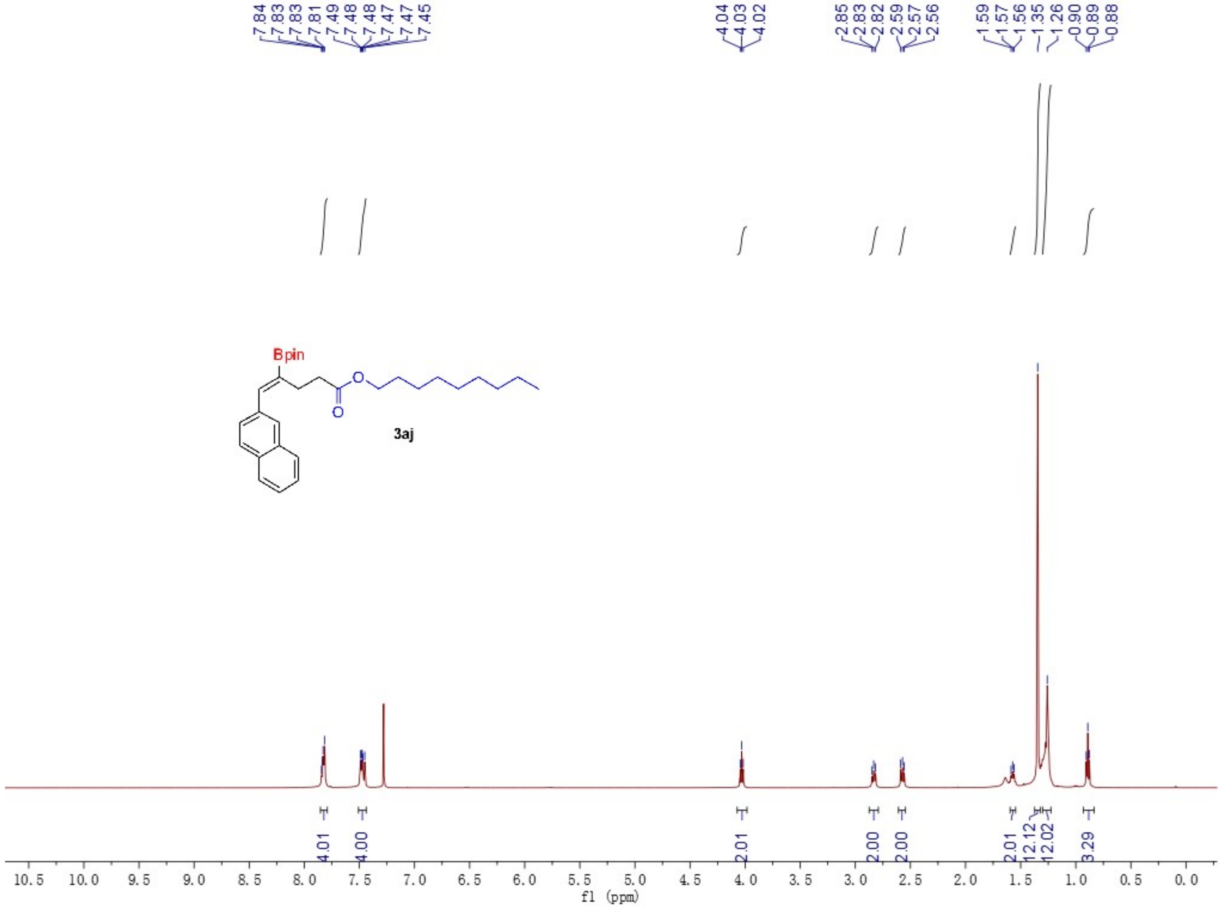
7.84
7.83
7.83
7.81
7.49
7.48
7.48
7.47
7.47
7.45
4.04
4.03
4.02
2.85
2.83
2.82
2.59
2.57
2.56
1.59
1.57
1.56
1.35
1.26
0.90
0.89
0.88
Bpin
O
3aj
4.01
4.00
2.01
2.00
2.00
2.01
12.12
12.02
3.29
f1 (ppm)

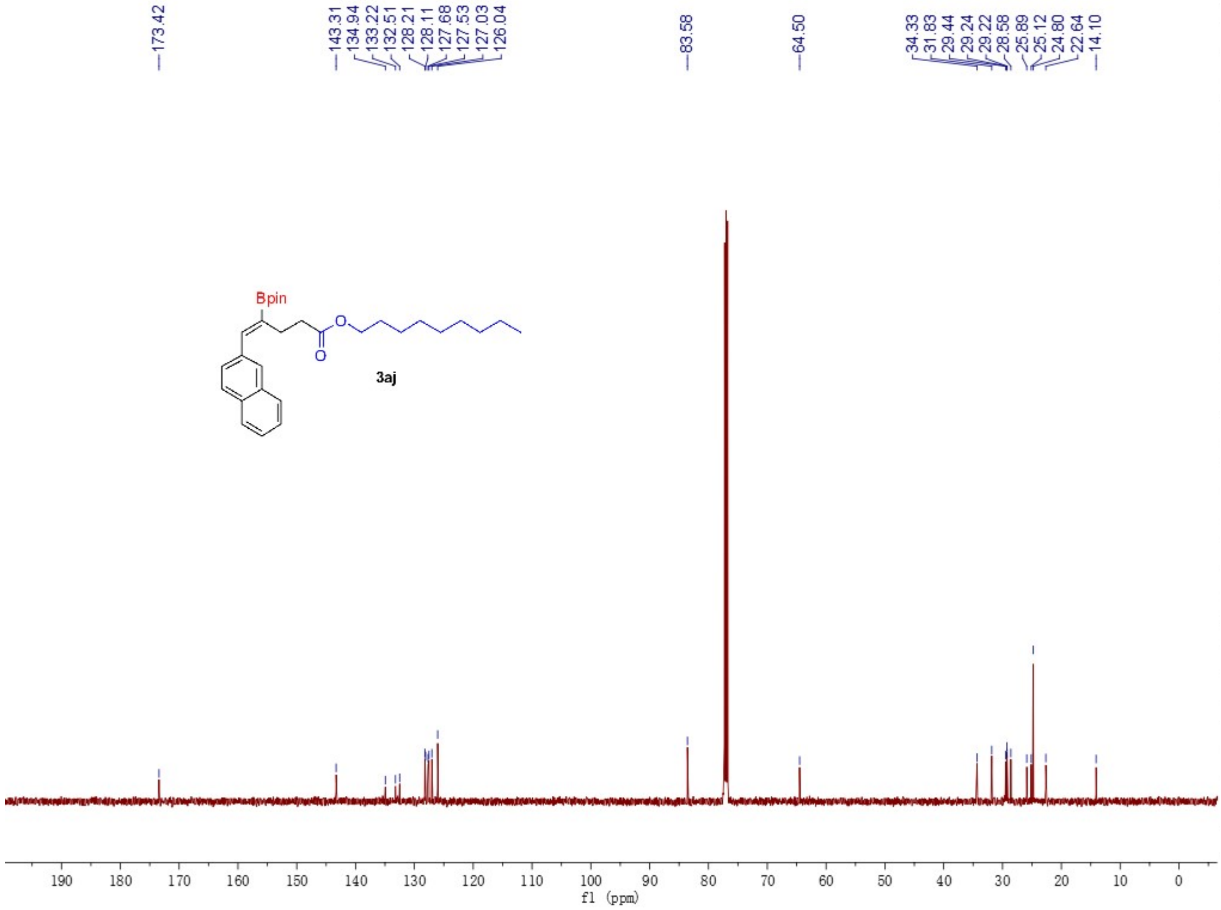
173.42
143.31
134.94
133.22
132.51
128.21
128.11
127.68
127.53
127.03
126.04
83.58
64.50
34.33
31.83
29.44
29.24
29.22
28.58
25.89
25.12
24.80
22.64
14.10
Bpin
O
O
3aj
f1 (ppm)

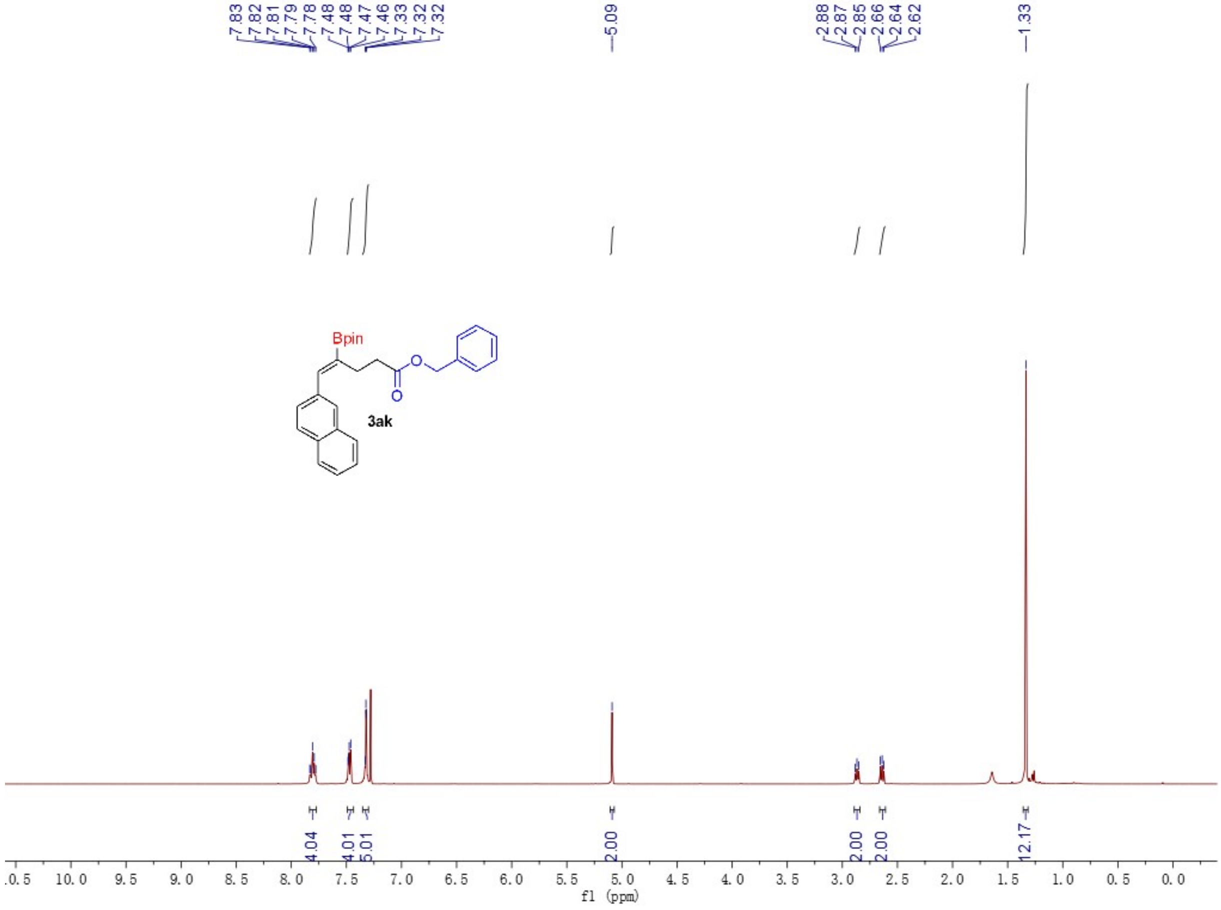
7.83
7.82
7.81
7.79
7.78
7.48
7.48
7.47
7.46
7.33
7.32
7.32
5.09
2.88
2.87
2.85
2.66
2.64
2.62
1.33
Bpin
O
O
3ak
4.04
4.01
5.01
2.00
2.00
2.00
12.17
f1 (ppm)

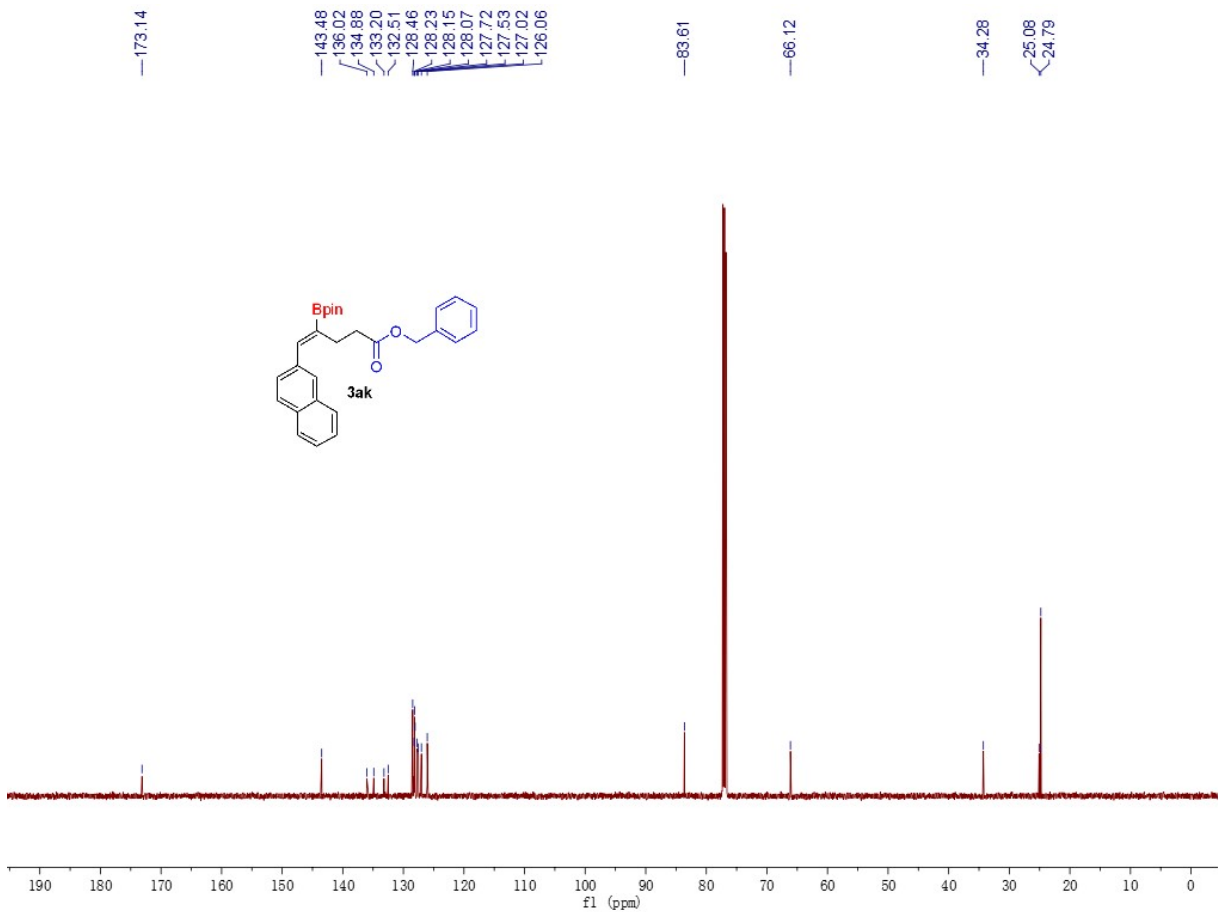

Bpin
3ak

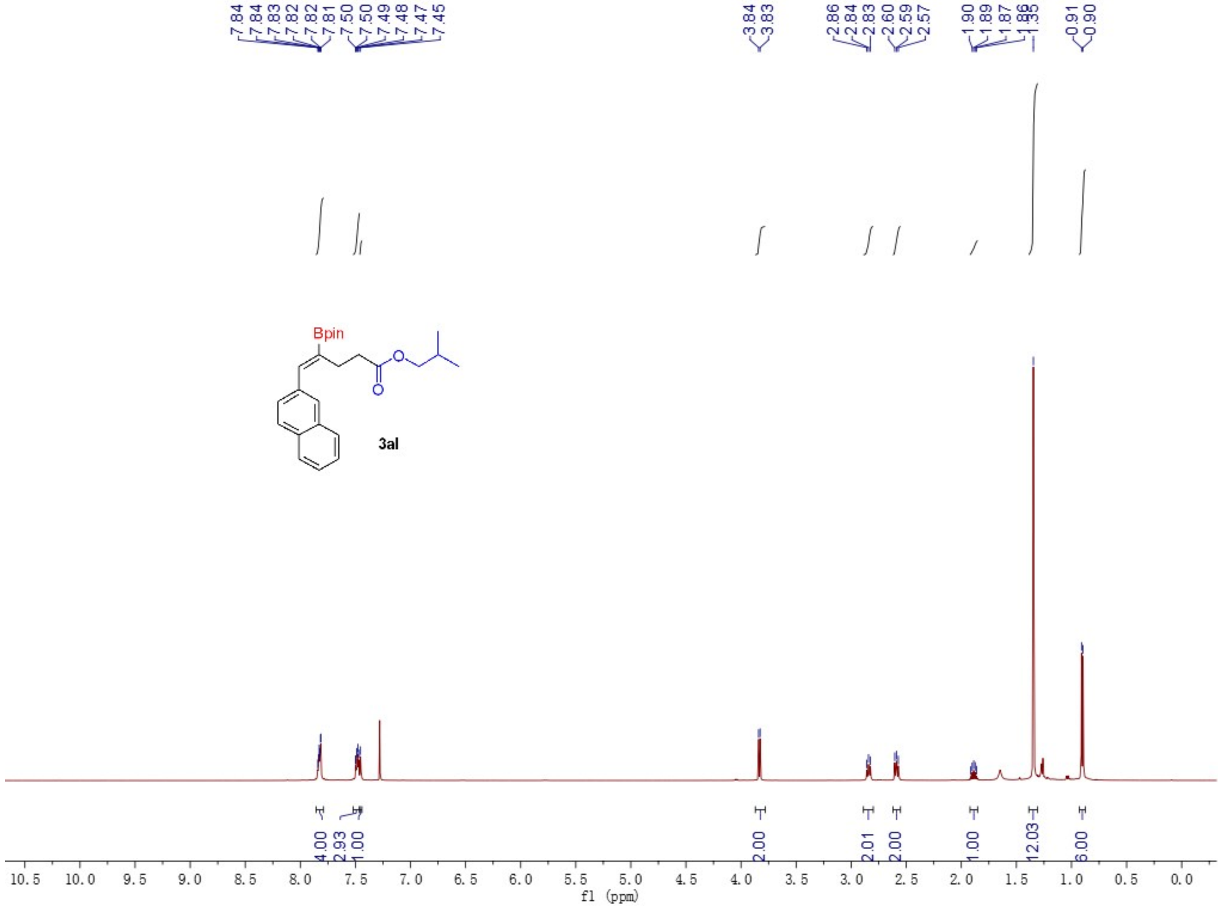

Bpin
3al

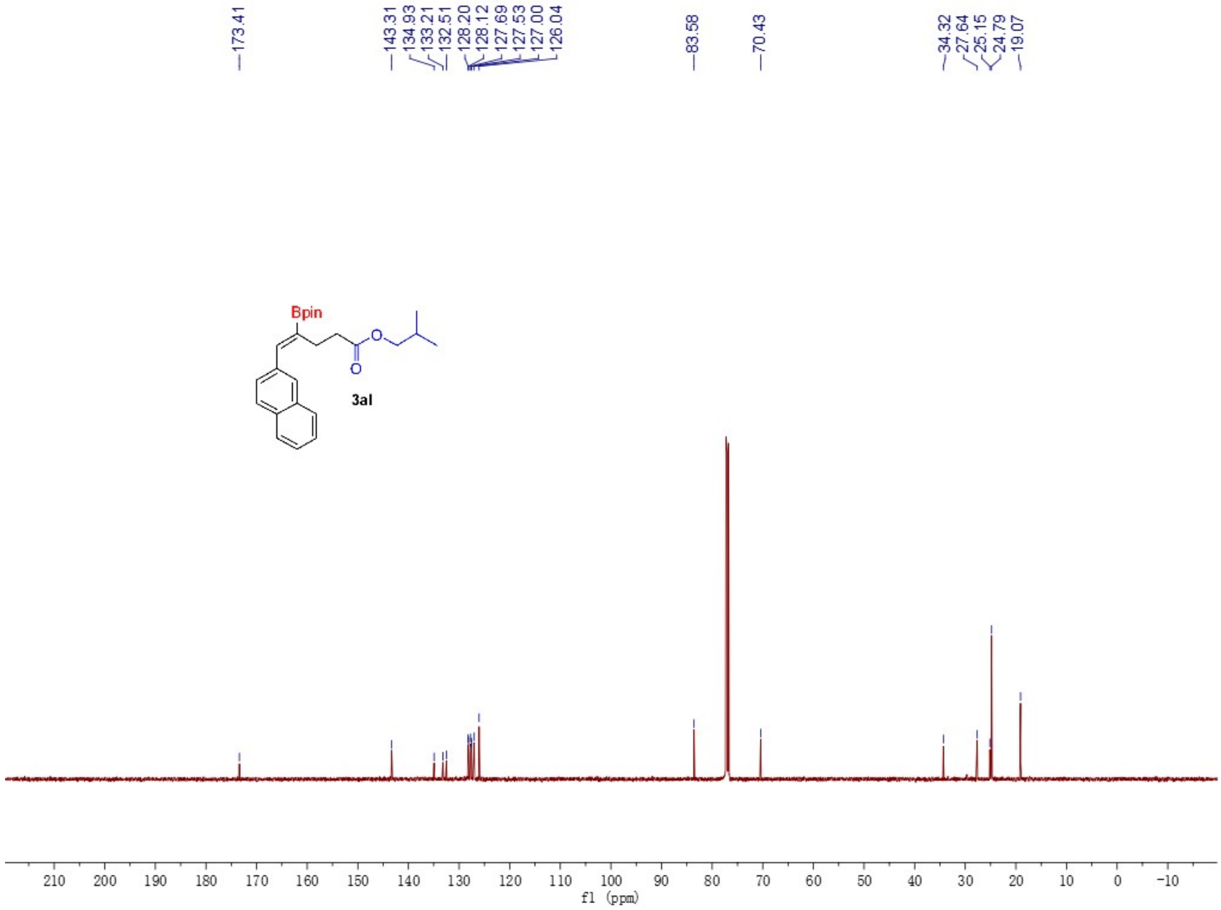
173.41
143.31
134.93
133.21
132.51
128.20
128.12
127.69
127.53
127.00
126.04
83.58
70.43
34.32
27.64
25.15
24.79
19.07
Bpin
O
O
3al
210 200 190 180 170 160 150 140 130 120 110 100 90 80 70 60 50 40 30 20 10 0 -10
f1 (ppm)

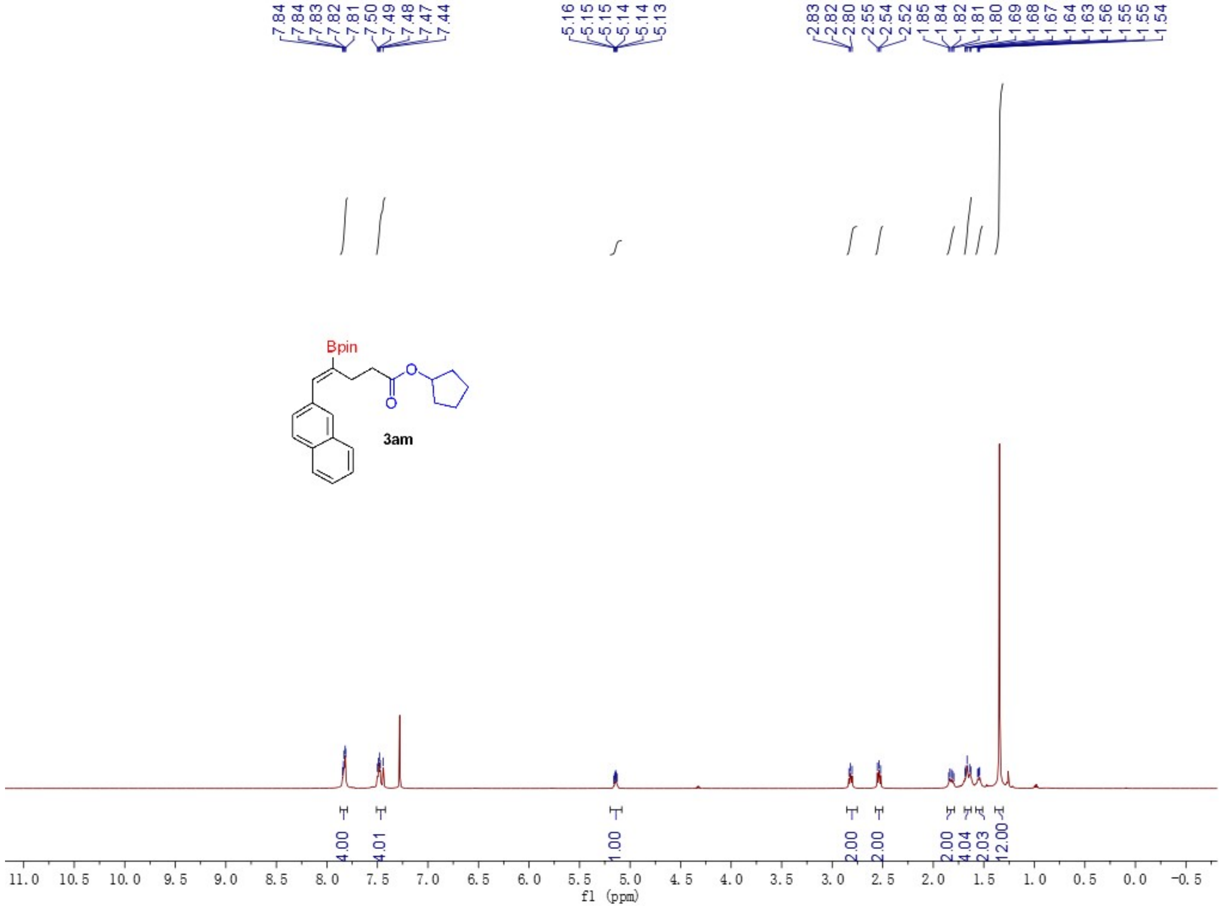
7.84
7.84
7.83
7.82
7.81
7.50
7.49
7.48
7.47
7.44
5.16
5.15
5.15
5.14
5.14
5.13
2.83
2.82
2.80
2.55
2.54
2.52
1.85
1.84
1.82
1.81
1.80
1.69
1.68
1.67
1.64
1.63
1.56
1.55
1.54
Bpin
O
O
3am
4.00
4.01
1.00
2.00
2.00
2.00
4.04
2.03
12.00
11.0 10.5 10.0 9.5 9.0 8.5 8.0 7.5 7.0 6.5 6.0 5.5 5.0 4.5 4.0 3.5 3.0 2.5 2.0 1.5 1.0 0.5 0.0 -0.5
f1 (ppm)

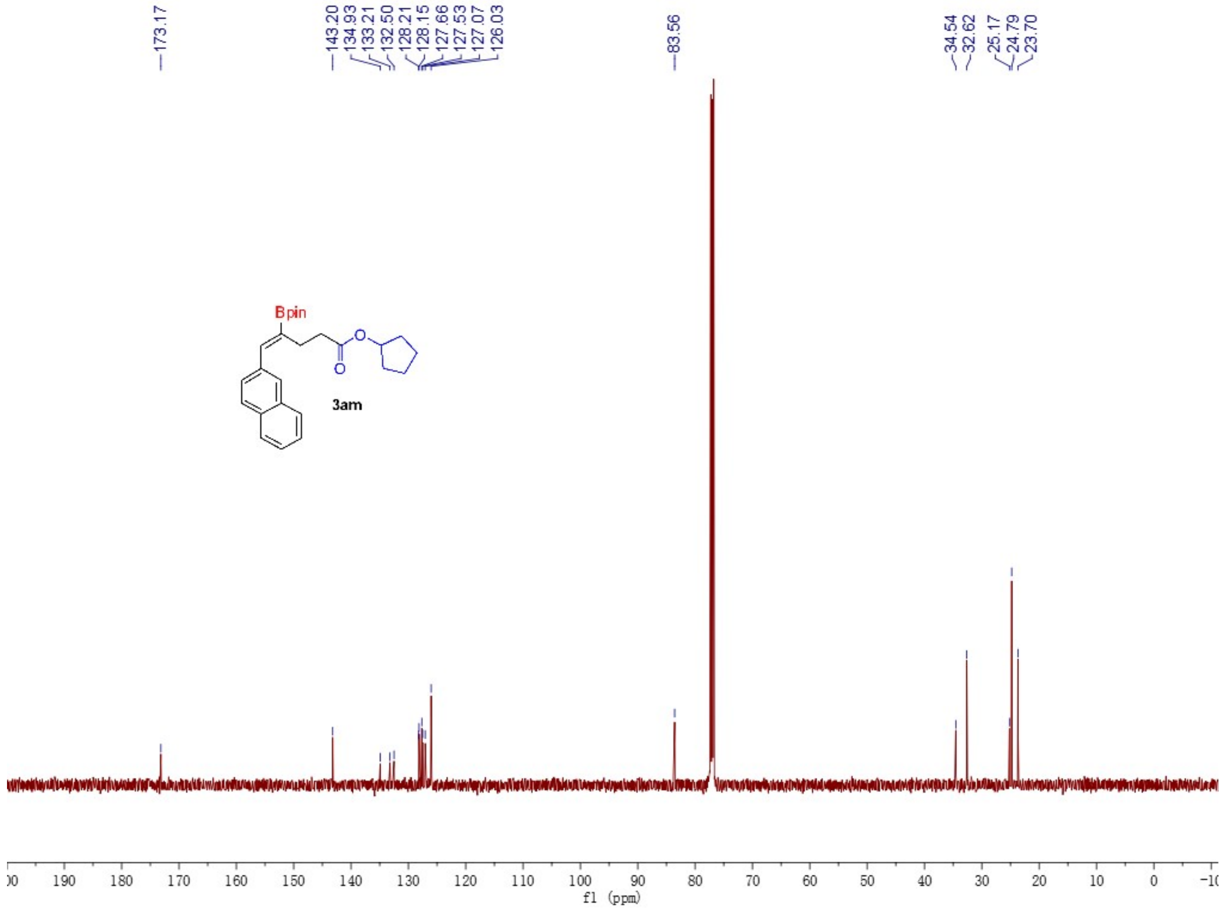
173.17
143.20
134.93
133.21
132.50
128.21
128.15
127.66
127.53
127.07
126.03
83.56
34.54
32.62
25.17
24.79
23.70
Bpin
O
O
3am
fl (ppm)

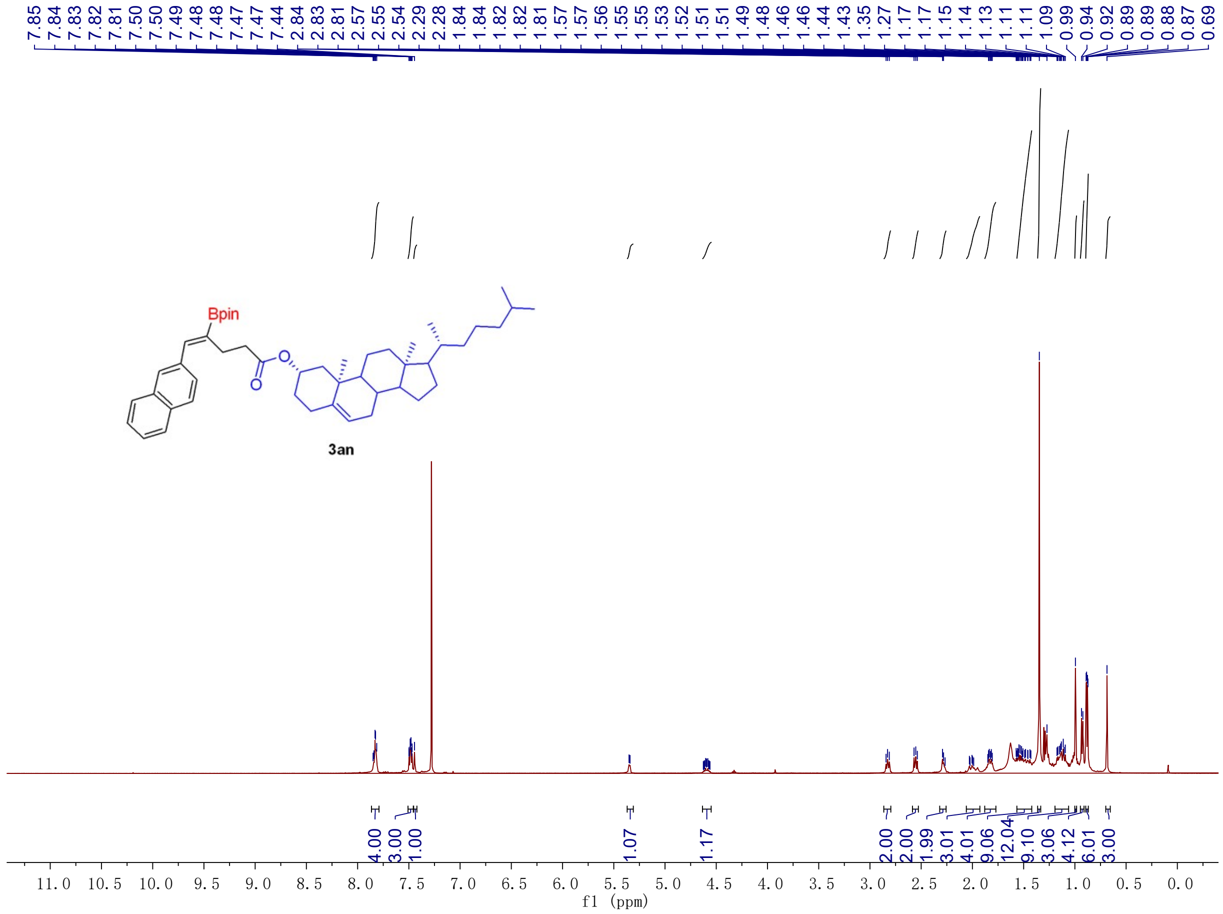
7.85
7.84
7.83
7.82
7.81
7.50
7.50
7.49
7.48
7.48
7.47
7.47
7.44
2.84
2.83
2.81
2.57
2.55
2.54
2.29
2.28
1.84
1.84
1.82
1.82
1.81
1.57
1.57
1.56
1.55
1.55
1.53
1.52
1.51
1.51
1.49
1.48
1.46
1.46
1.44
1.43
1.35
1.27
1.17
1.17
1.15
1.14
1.13
1.11
1.11
1.09
0.99
0.94
0.92
0.89
0.88
0.87
0.69
Bpin
O
O
3an
4.00
3.00
1.00
1.07
1.17
2.00
2.00
1.99
3.01
4.01
9.06
12.04
9.10
3.06
4.12
6.01
3.00
fl (ppm)

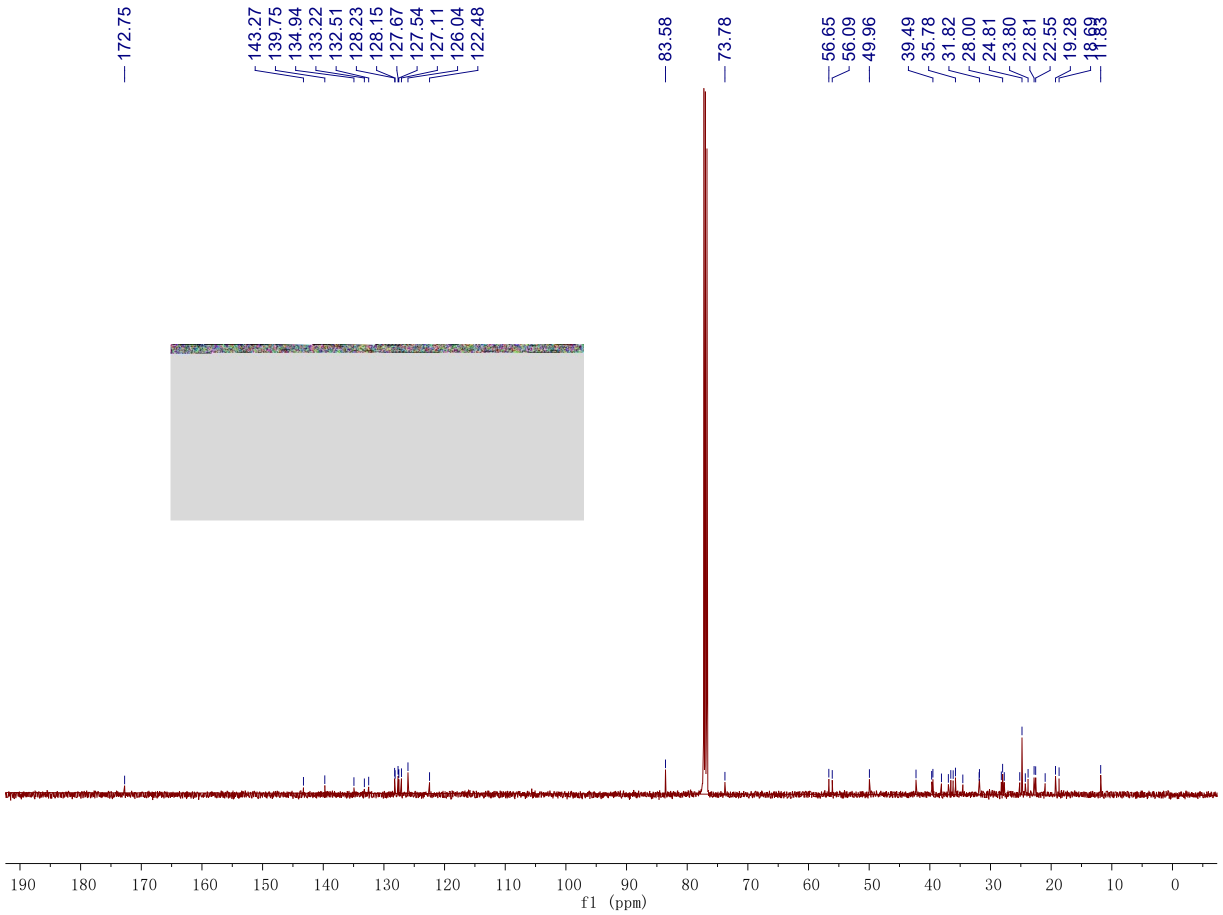
172.75
143.27 139.75 134.94 133.22 132.51 128.23 128.15 127.67 127.54 127.11 126.04 122.48
83.58
73.78
56.65 56.09 49.96
39.49 35.78 31.82 28.00 24.81 23.80 22.81 22.55 19.28 18.69 11.83
190 180 170 160 150 140 130 120 110 100 90 80 70 60 50 40 30 20 10 0
f1 (ppm)

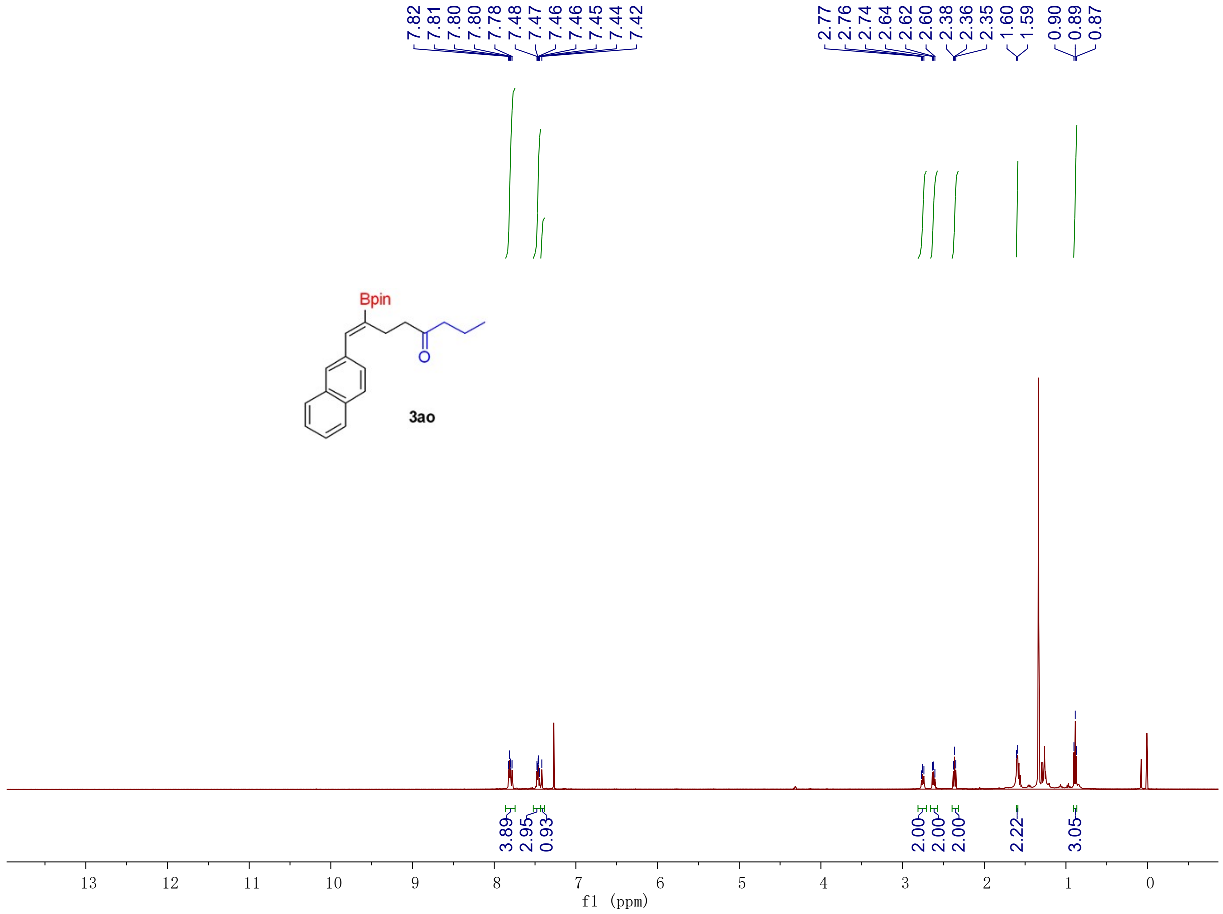
7.82 7.81 7.80 7.80 7.78 7.48 7.47 7.46 7.46 7.45 7.44 7.42
2.77 2.76 2.74 2.64 2.62 2.60 2.38 2.36 2.35 1.60 1.59 0.90 0.89 0.87
3.89 2.95 0.93
2.00 2.00 2.00 2.22 3.05
13 12 11 10 9 8 7 6 5 4 3 2 1 0
f1 (ppm)

Bpin
O

3ao

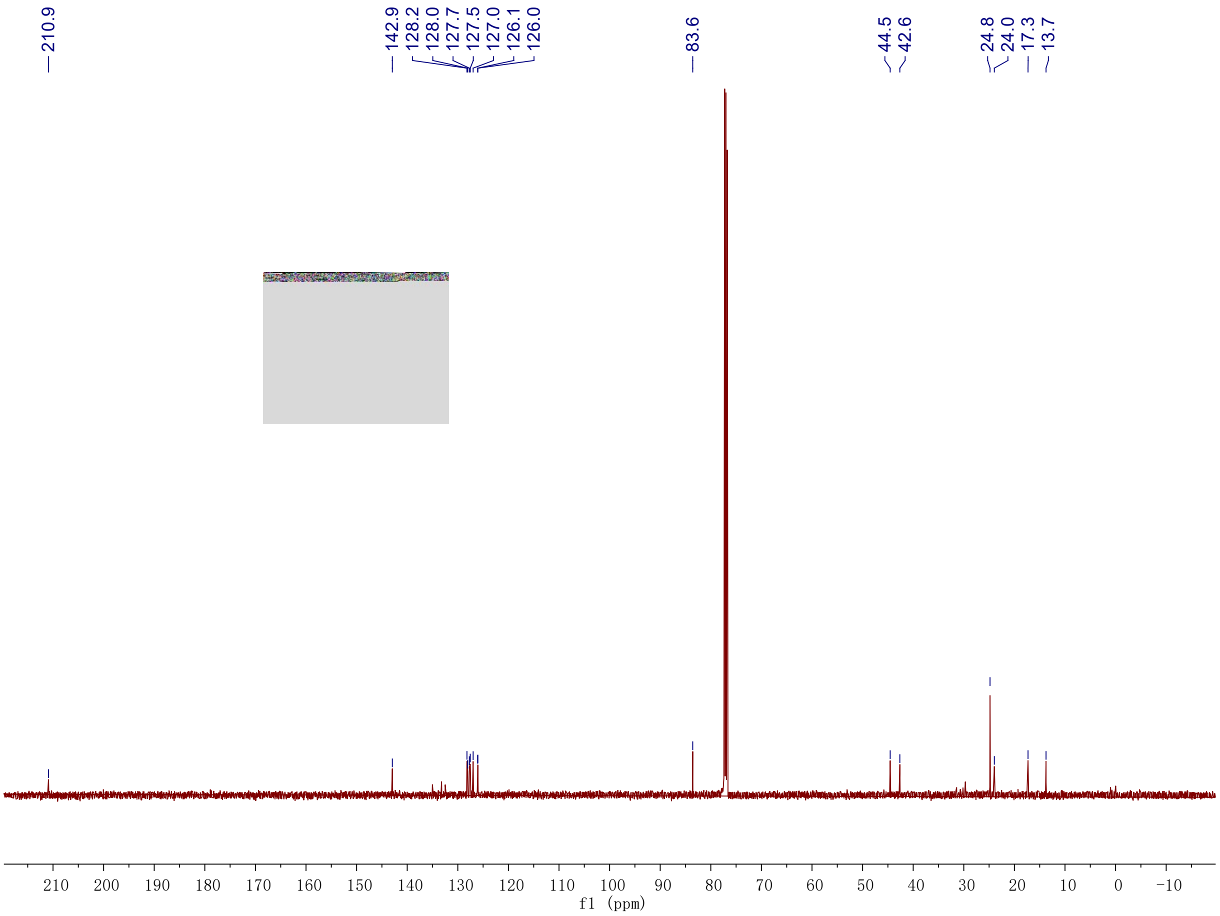
210.9
142.9 128.2 128.0 127.7 127.5 127.0 126.1 126.0
83.6
44.5 42.6
24.8 24.0 17.3 13.7
f1 (ppm)

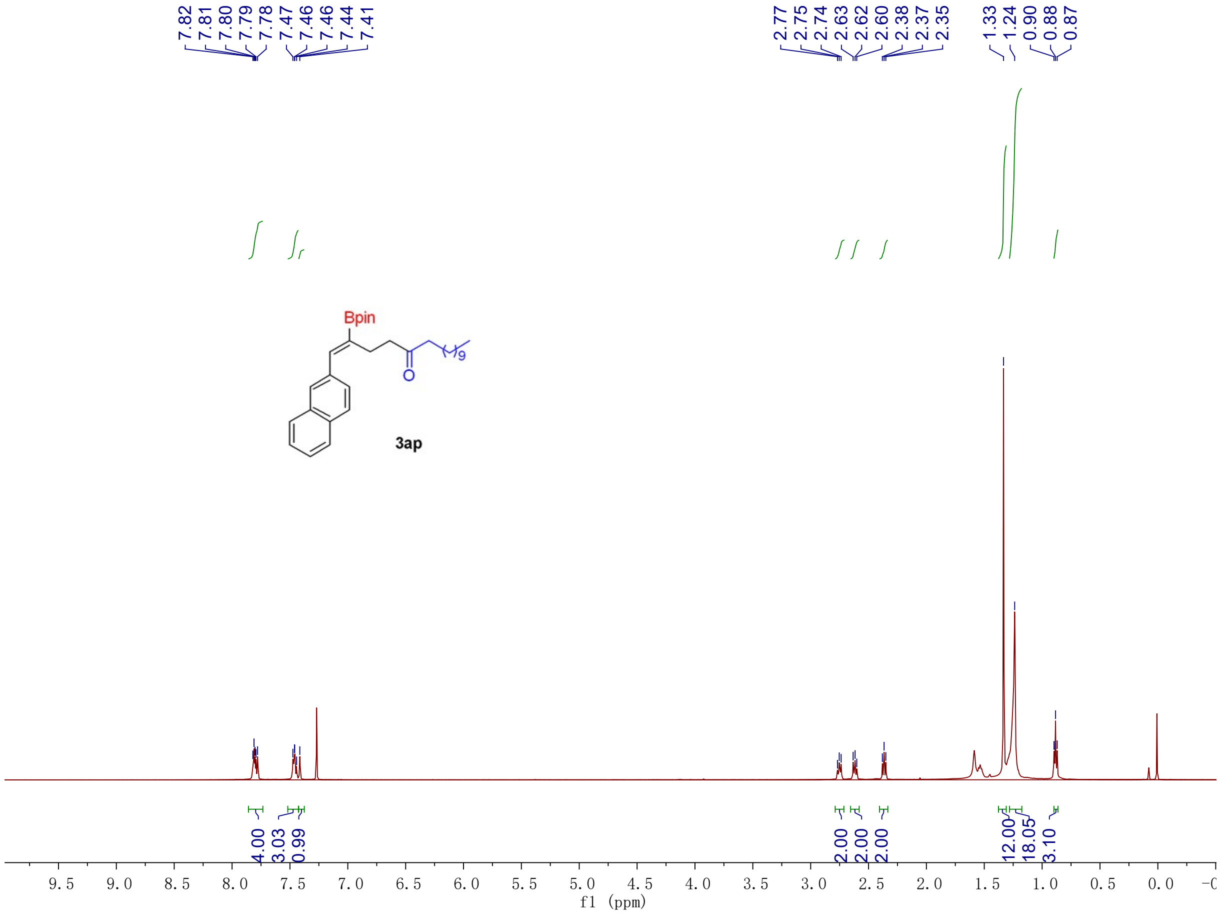
7.82 7.81 7.80 7.79 7.78 7.47 7.46 7.46 7.44 7.41
2.77 2.75 2.74 2.63 2.62 2.60 2.38 2.37 2.35
1.33 1.24 0.90 0.88 0.87
Bpin
O
9
3ap
4.00 3.03 0.99
2.00 2.00 2.00
12.00 18.05 3.10
f1 (ppm)

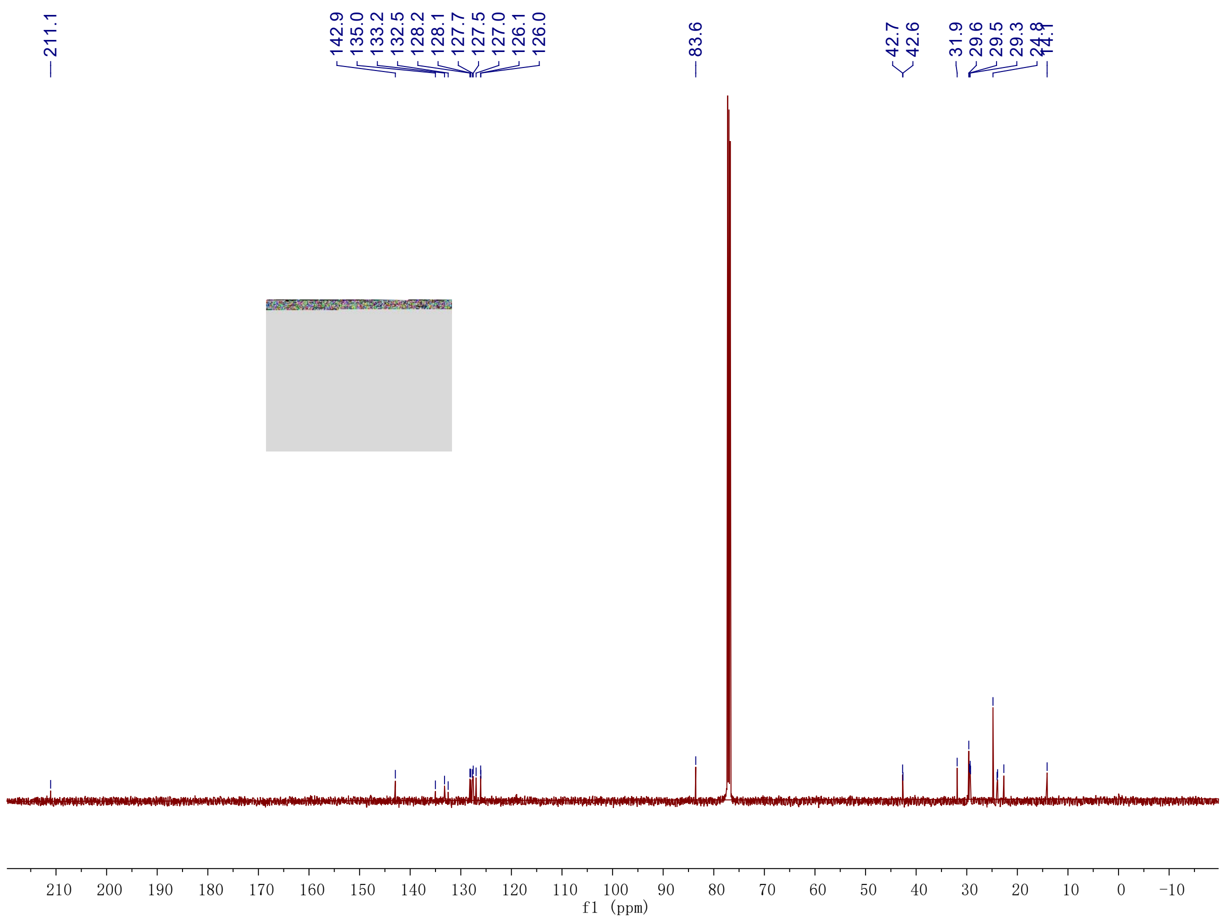
211.1
142.9 135.0 133.2 132.5 128.2 128.1 127.7 127.5 127.0 126.1 126.0
83.6
42.7 42.6
31.9 29.6 29.5 29.3 24.8 14.1
210 200 190 180 170 160 150 140 130 120 110 100 90 80 70 60 50 40 30 20 10 0 −10
f1 (ppm)

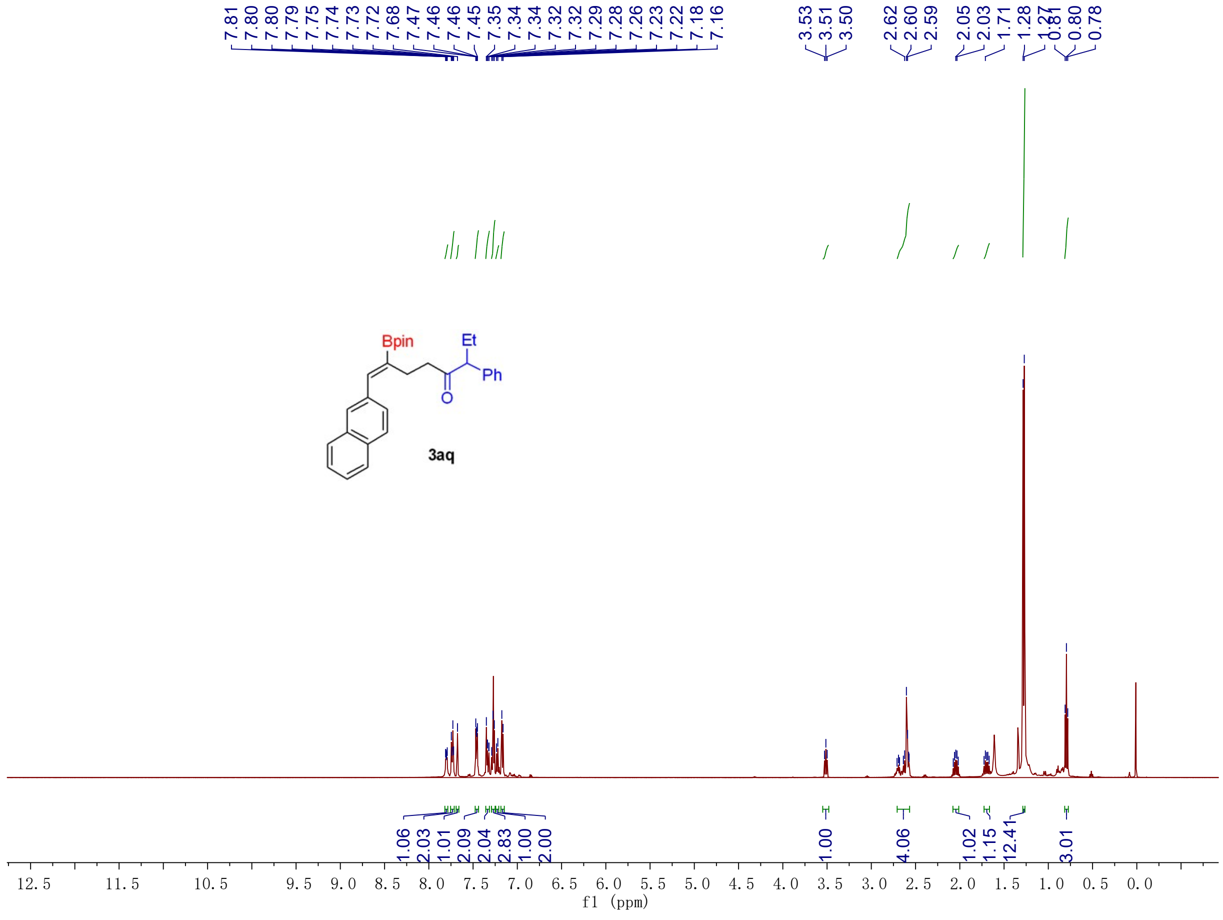
7.81 7.80 7.79 7.75 7.74 7.73 7.72 7.68 7.47 7.46 7.45 7.35 7.34 7.34 7.32 7.32 7.29 7.28 7.26 7.23 7.22 7.18 7.16
3.53 3.51 3.50
2.62 2.60 2.59
2.05 2.03 1.71 1.28 1.27 0.81 0.80 0.78
Bpin
Et
Ph
O
3aq
1.06 2.03 1.01 2.09 2.04 2.83 1.00 2.00
1.00
4.06
1.02 1.15 12.41
3.01
12.5 11.5 10.5 9.5 9.0 8.5 8.0 7.5 7.0 6.5 6.0 5.5 5.0 4.5 4.0 3.5 3.0 2.5 2.0 1.5 1.0 0.5 0.0
f1 (ppm)

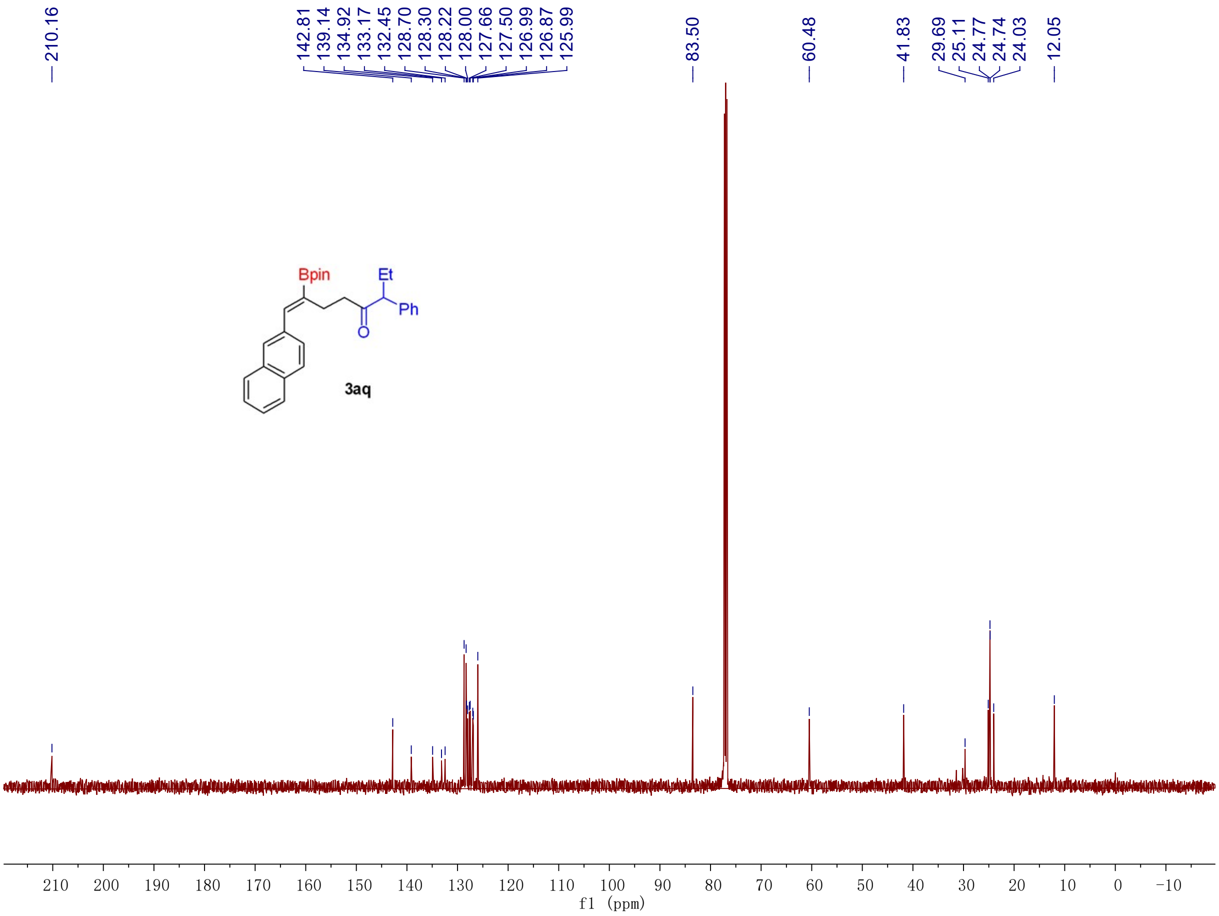
210.16
142.81
139.14
134.92
133.17
132.45
128.70
128.30
128.22
128.00
127.66
127.50
126.99
126.87
125.99
83.50
60.48
41.83
29.69
25.11
24.77
24.74
24.03
12.05
Bpin
Et
Ph
O
3aq
210 200 190 180 170 160 150 140 130 120 110 100 90 80 70 60 50 40 30 20 10 0 -10
f1 (ppm)

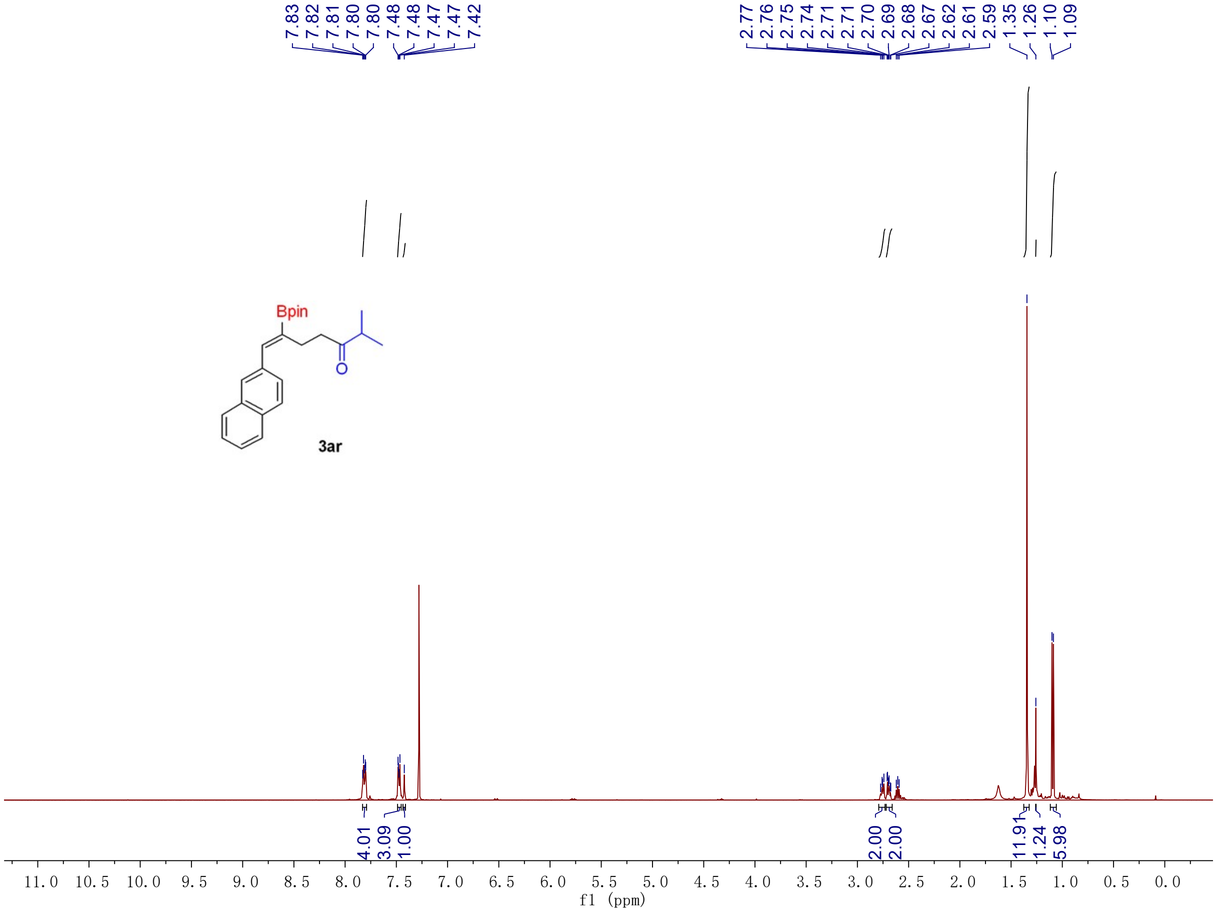
7.83
7.82
7.81
7.80
7.80
7.48
7.48
7.47
7.47
7.42
2.77
2.76
2.75
2.74
2.71
2.70
2.69
2.68
2.67
2.62
2.61
2.59
1.35
1.26
1.10
1.09
Bpin
O
3ar
4.01
3.09
1.00
2.00
2.00
11.91
1.24
5.98
11.0 10.5 10.0 9.5 9.0 8.5 8.0 7.5 7.0 6.5 6.0 5.5 5.0 4.5 4.0 3.5 3.0 2.5 2.0 1.5 1.0 0.5 0.0
f1 (ppm)

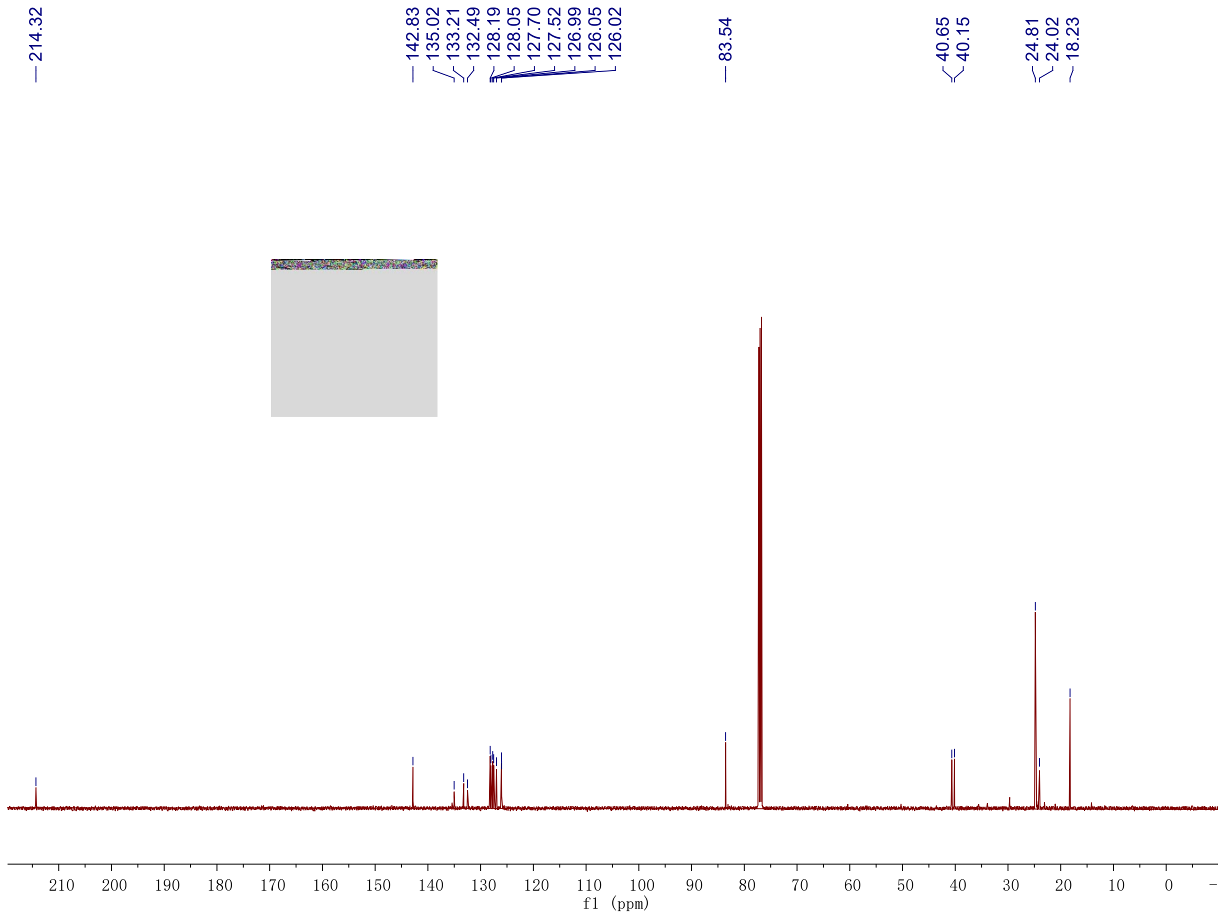

214.32
142.83
135.02
133.21
132.49
128.19
128.05
127.70
127.52
126.99
126.05
126.02
83.54
40.65
40.15
24.81
24.02
18.23
210 200 190 180 170 160 150 140 130 120 110 100 90 80 70 60 50 40 30 20 10 0
f1 (ppm)

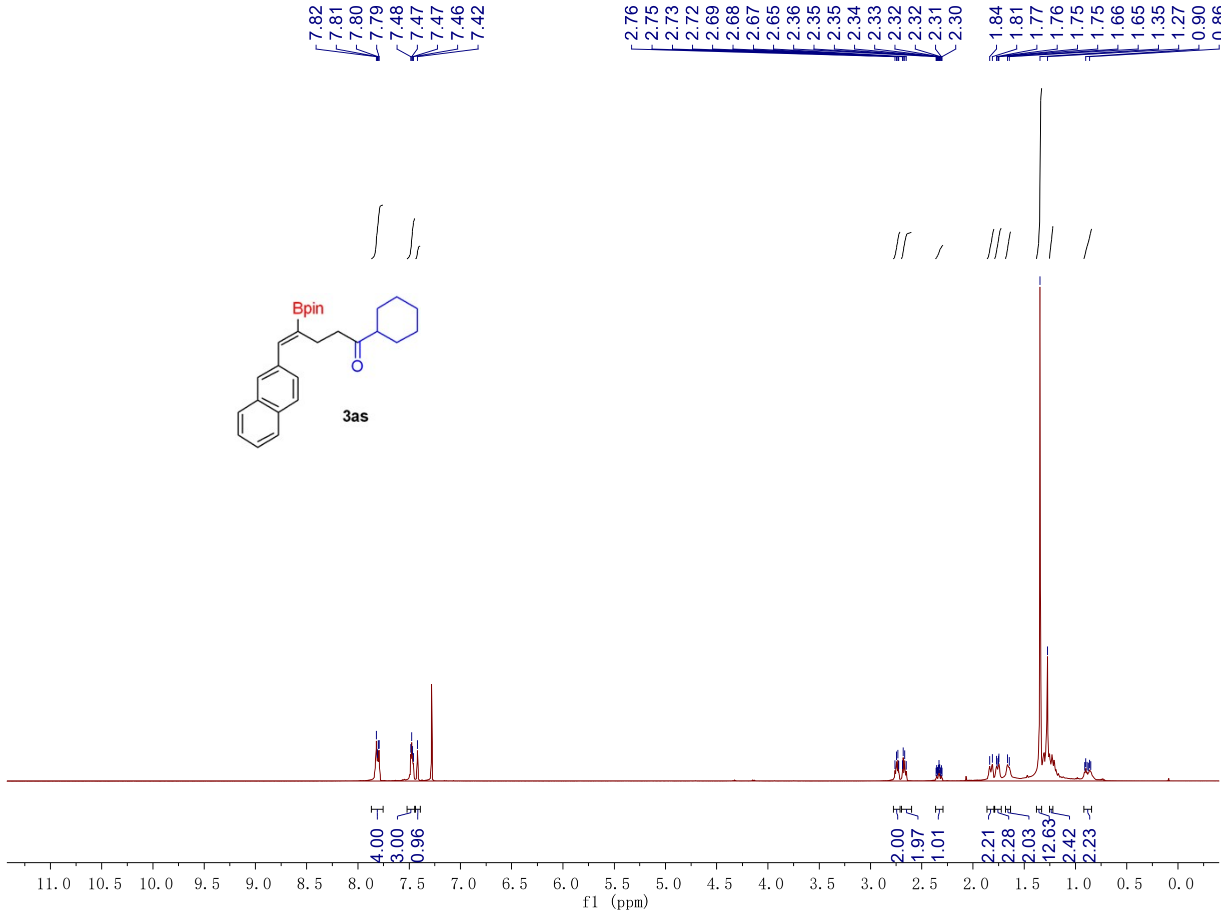

7.82
7.81
7.80
7.79
7.48
7.47
7.47
7.46
7.42
2.76
2.75
2.73
2.72
2.69
2.68
2.67
2.65
2.36
2.35
2.35
2.34
2.33
2.32
2.32
2.31
2.30
1.84
1.81
1.77
1.76
1.75
1.66
1.65
1.35
1.27
0.90
Bpin
O
3as
4.00
3.00
0.96
2.00
1.97
1.01
2.21
2.28
2.03
12.63
2.42
2.23
11.0 10.5 10.0 9.5 9.0 8.5 8.0 7.5 7.0 6.5 6.0 5.5 5.0 4.5 4.0 3.5 3.0 2.5 2.0 1.5 1.0 0.5 0.0
f1 (ppm)

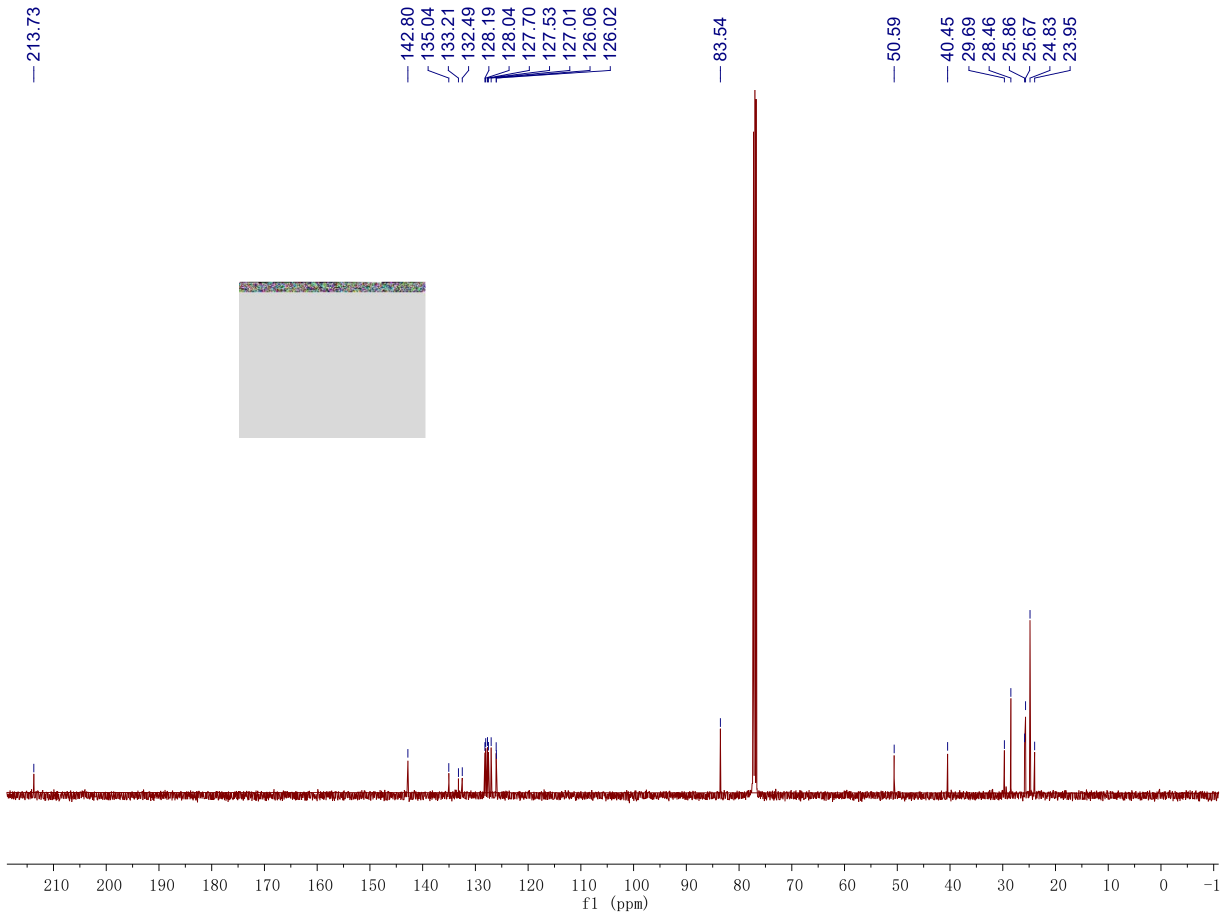
213.73
142.80
135.04
133.21
132.49
128.19
128.04
127.70
127.53
127.01
126.06
126.02
83.54
50.59
40.45
29.69
28.46
25.86
25.67
24.83
23.95
f1 (ppm)

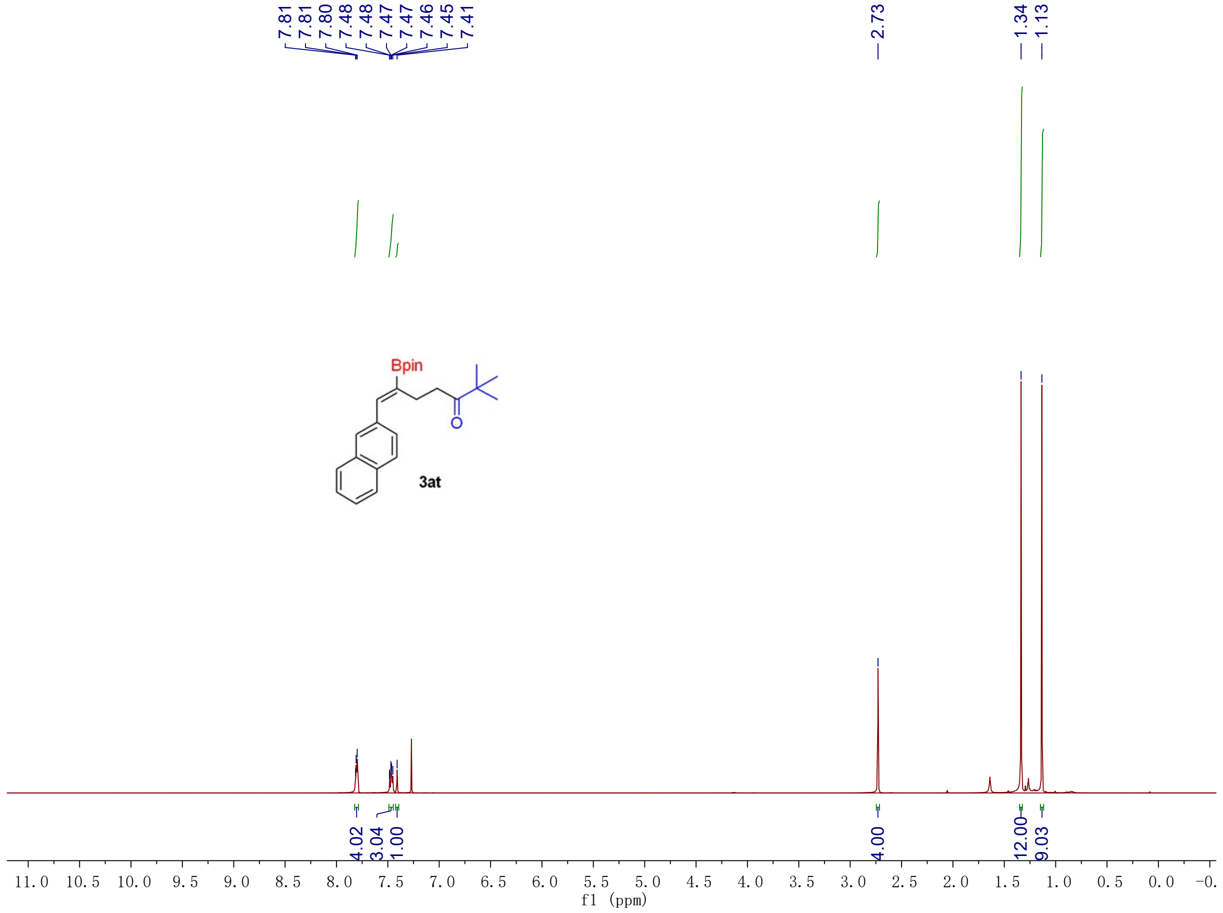
7.81
7.81
7.80
7.48
7.48
7.47
7.46
7.45
7.41
2.73
1.34
1.13
Bpin
O
3at
4.02
3.04
1.00
4.00
12.00
9.03
f1 (ppm)

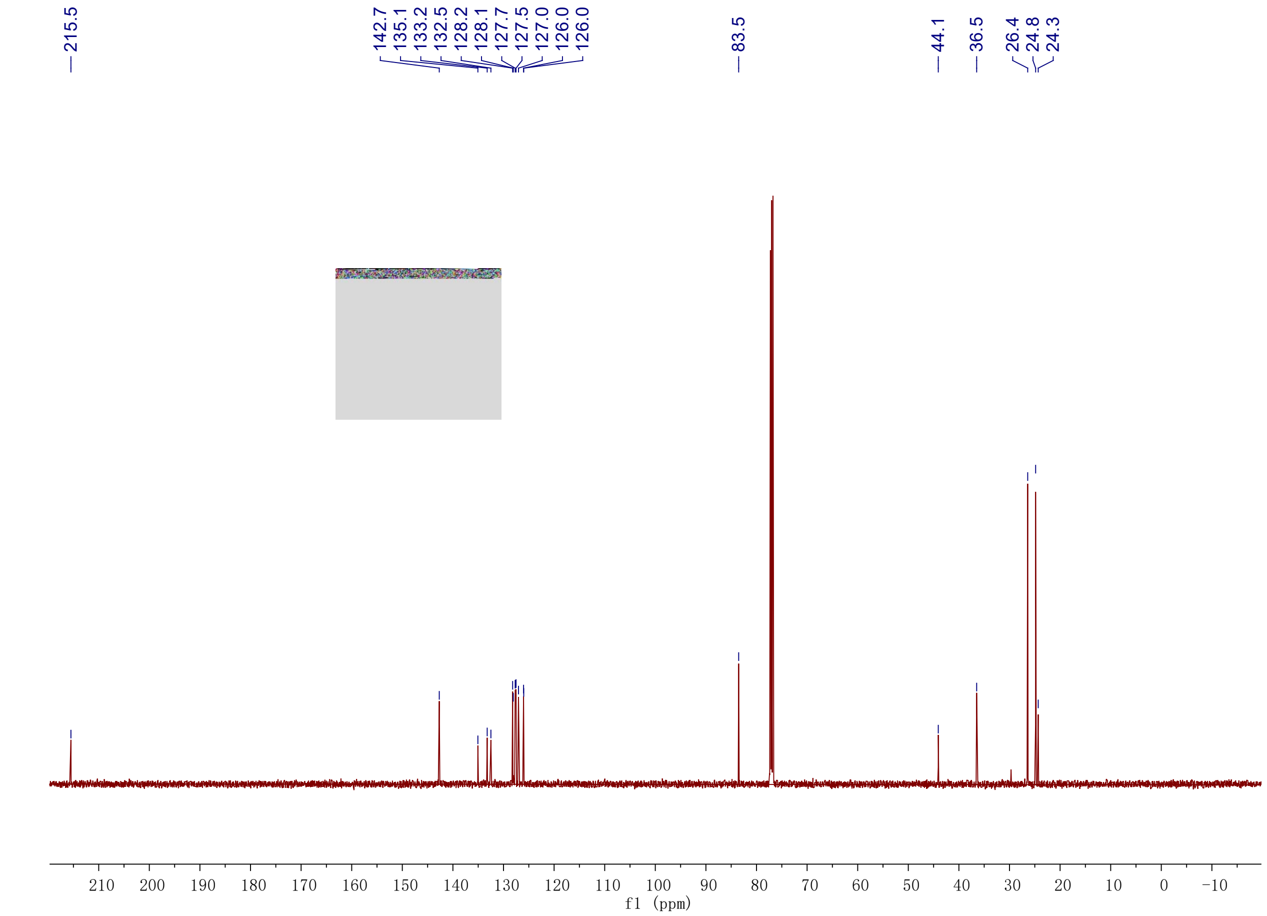
215.5
142.7
135.1
133.2
132.5
128.2
128.1
127.7
127.5
127.0
126.0
126.0
83.5
44.1
36.5
26.4
24.8
24.3
210 200 190 180 170 160 150 140 130 120 110 100 90 80 70 60 50 40 30 20 10 0 -10
f1 (ppm)

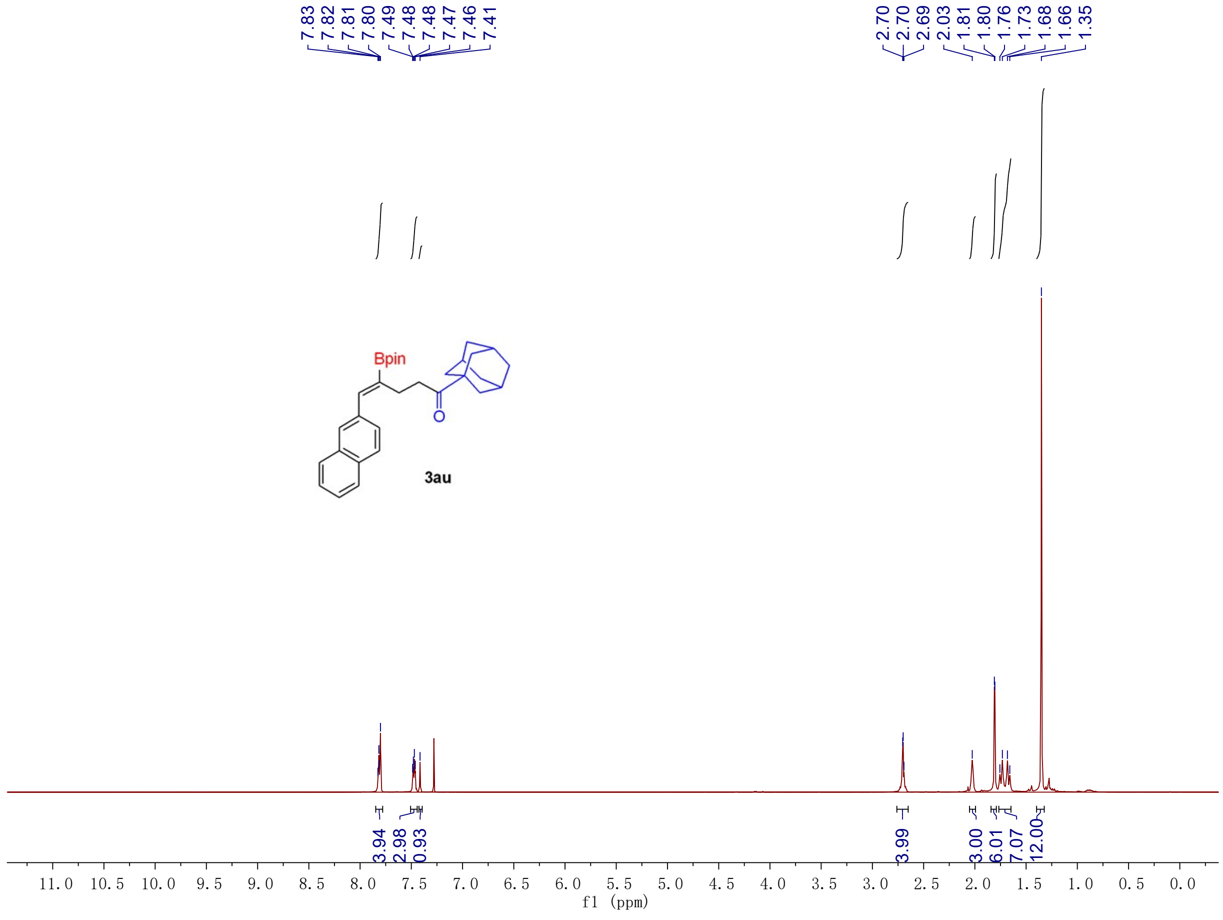
7.83
7.82
7.81
7.80
7.49
7.48
7.48
7.47
7.46
7.41
2.70
2.70
2.69
2.03
1.81
1.80
1.76
1.73
1.68
1.66
1.35
Bpin
O
3au
3.94
2.98
0.93
3.99
3.00
6.01
7.07
12.00
11.0 10.5 10.0 9.5 9.0 8.5 8.0 7.5 7.0 6.5 6.0 5.5 5.0 4.5 4.0 3.5 3.0 2.5 2.0 1.5 1.0 0.5 0.0
f1 (ppm)

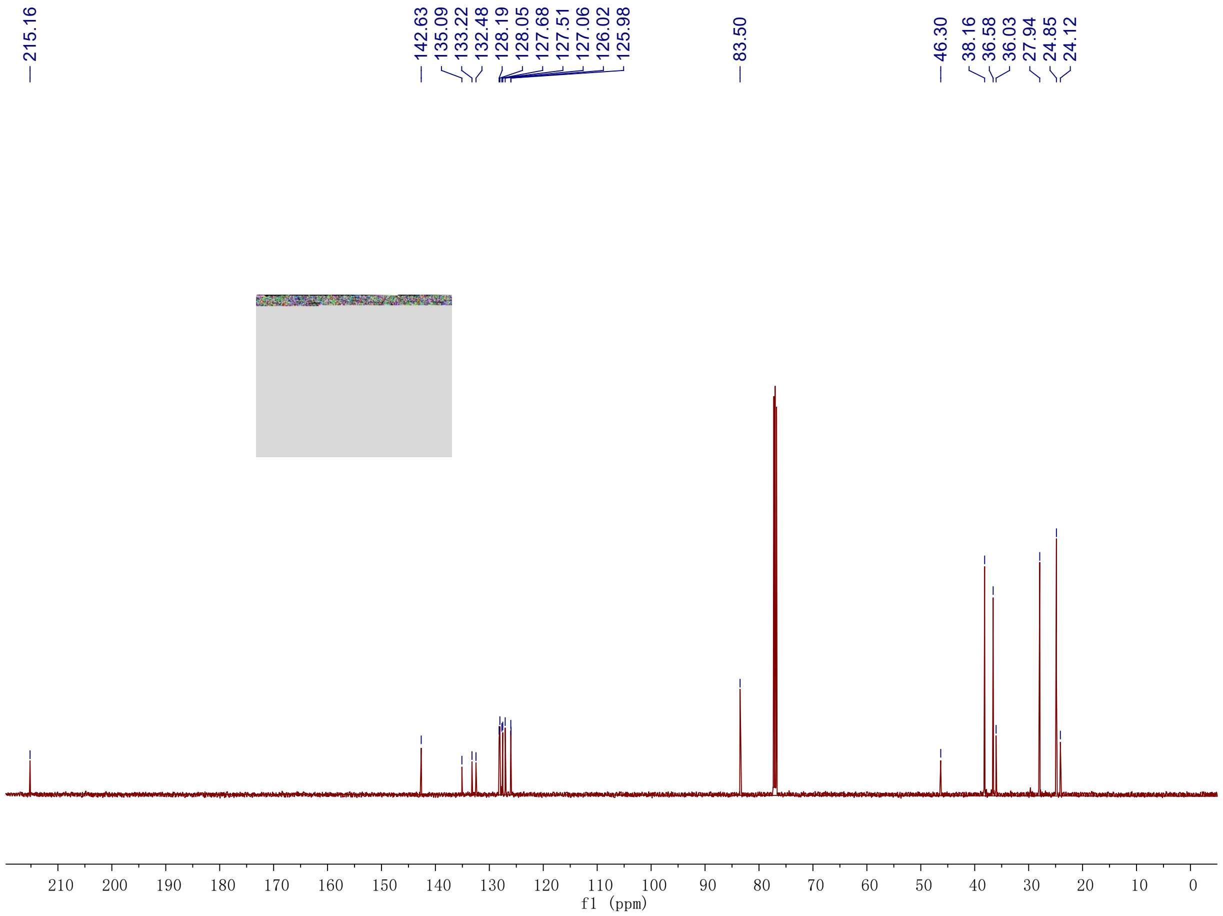
215.16
142.63
135.09
133.22
132.48
128.19
128.05
127.68
127.51
127.06
126.02
125.98
83.50
46.30
38.16
36.58
36.03
27.94
24.85
24.12
210 200 190 180 170 160 150 140 130 120 110 100 90 80 70 60 50 40 30 20 10 0
f1 (ppm)

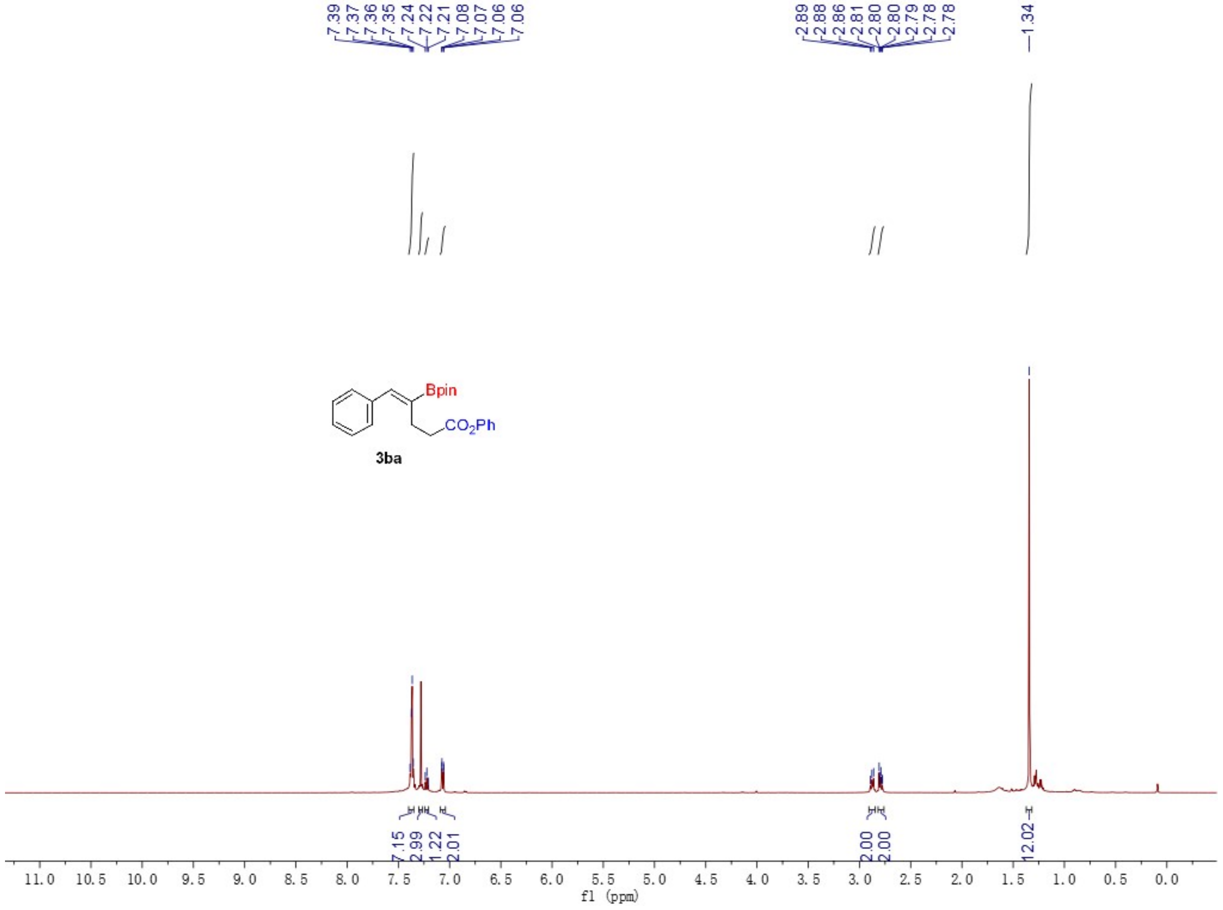
7.39
7.37
7.36
7.35
7.24
7.22
7.21
7.08
7.07
7.06
7.06
2.89
2.88
2.86
2.81
2.80
2.80
2.79
2.78
2.78
1.34
Bpin
CO2Ph
3ba
7.15
2.99
1.22
2.01
2.00
2.00
12.02
11.0 10.5 10.0 9.5 9.0 8.5 8.0 7.5 7.0 6.5 6.0 5.5 5.0 4.5 4.0 3.5 3.0 2.5 2.0 1.5 1.0 0.5 0.0
f1 (ppm)

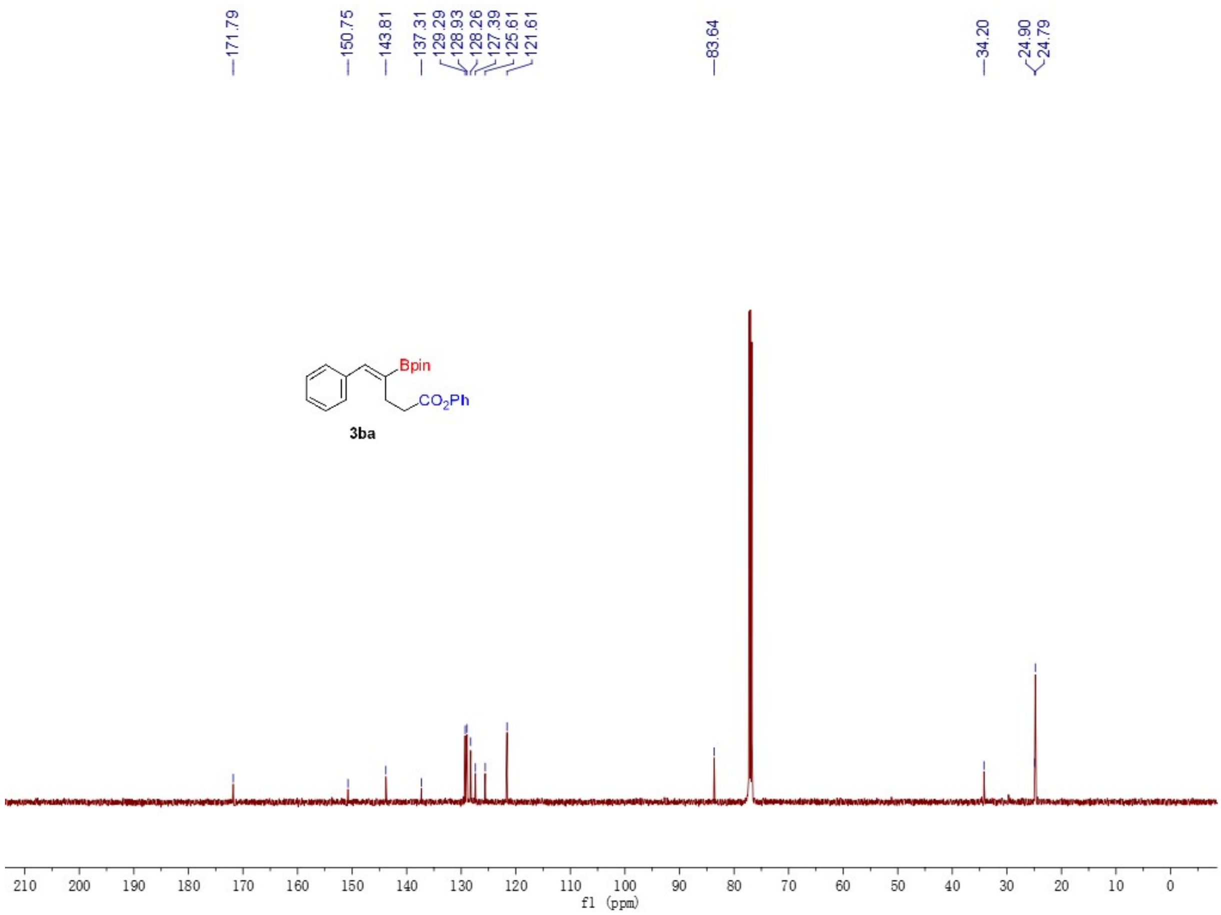
171.79
150.75
143.81
137.31
129.29
128.93
128.26
127.39
125.61
121.61
83.64
34.20
24.90
24.79
Bpin
CO2Ph
3ba
210 200 190 180 170 160 150 140 130 120 110 100 90 80 70 60 50 40 30 20 10 0
f1 (ppm)

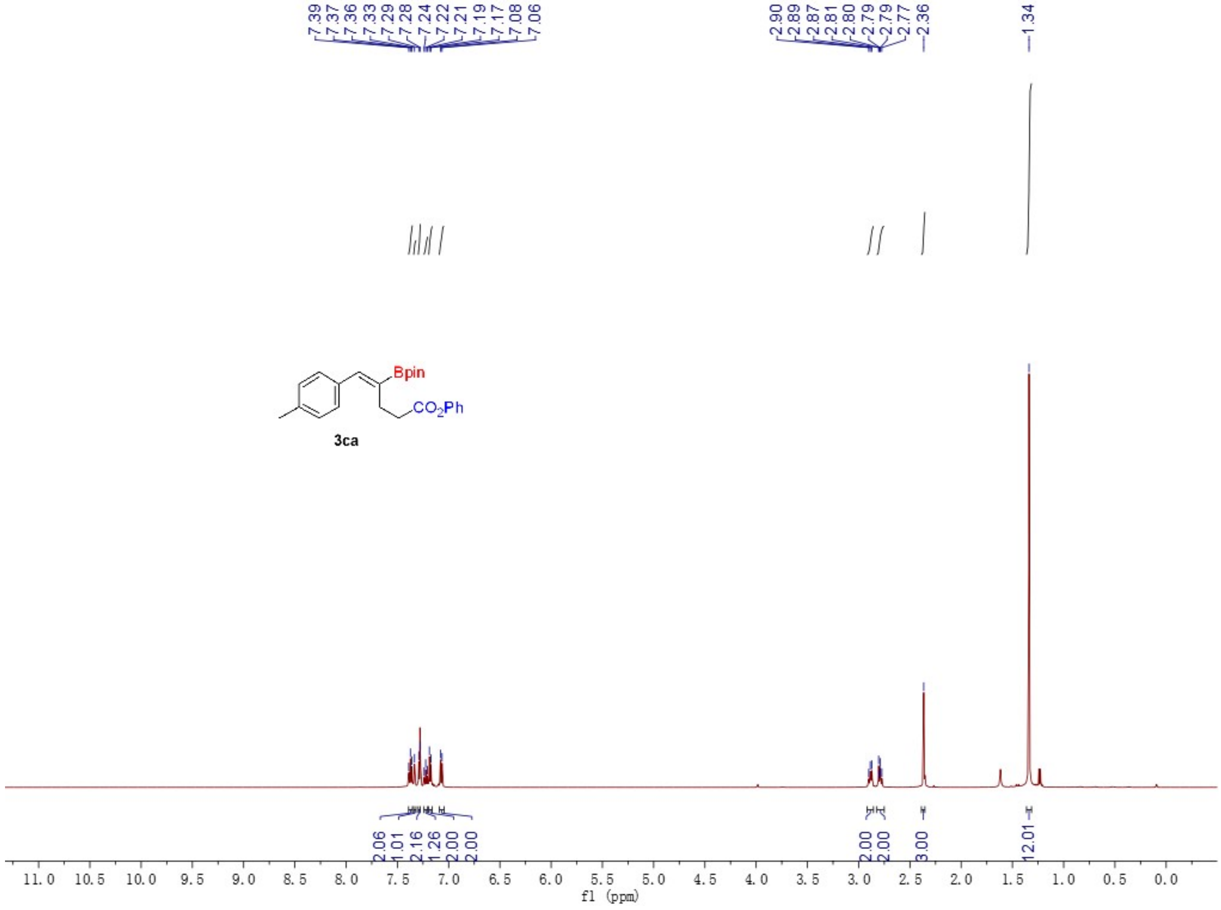
7.39
7.37
7.36
7.33
7.29
7.28
7.24
7.22
7.21
7.19
7.17
7.08
7.06
2.90
2.89
2.87
2.81
2.80
2.79
2.79
2.77
2.36
1.34
Bpin
CO2Ph
3ca
2.06
1.01
2.16
1.26
2.00
2.00
2.00
2.00
3.00
12.01
11.0 10.5 10.0 9.5 9.0 8.5 8.0 7.5 7.0 6.5 6.0 5.5 5.0 4.5 4.0 3.5 3.0 2.5 2.0 1.5 1.0 0.5 0.0
f1 (ppm)

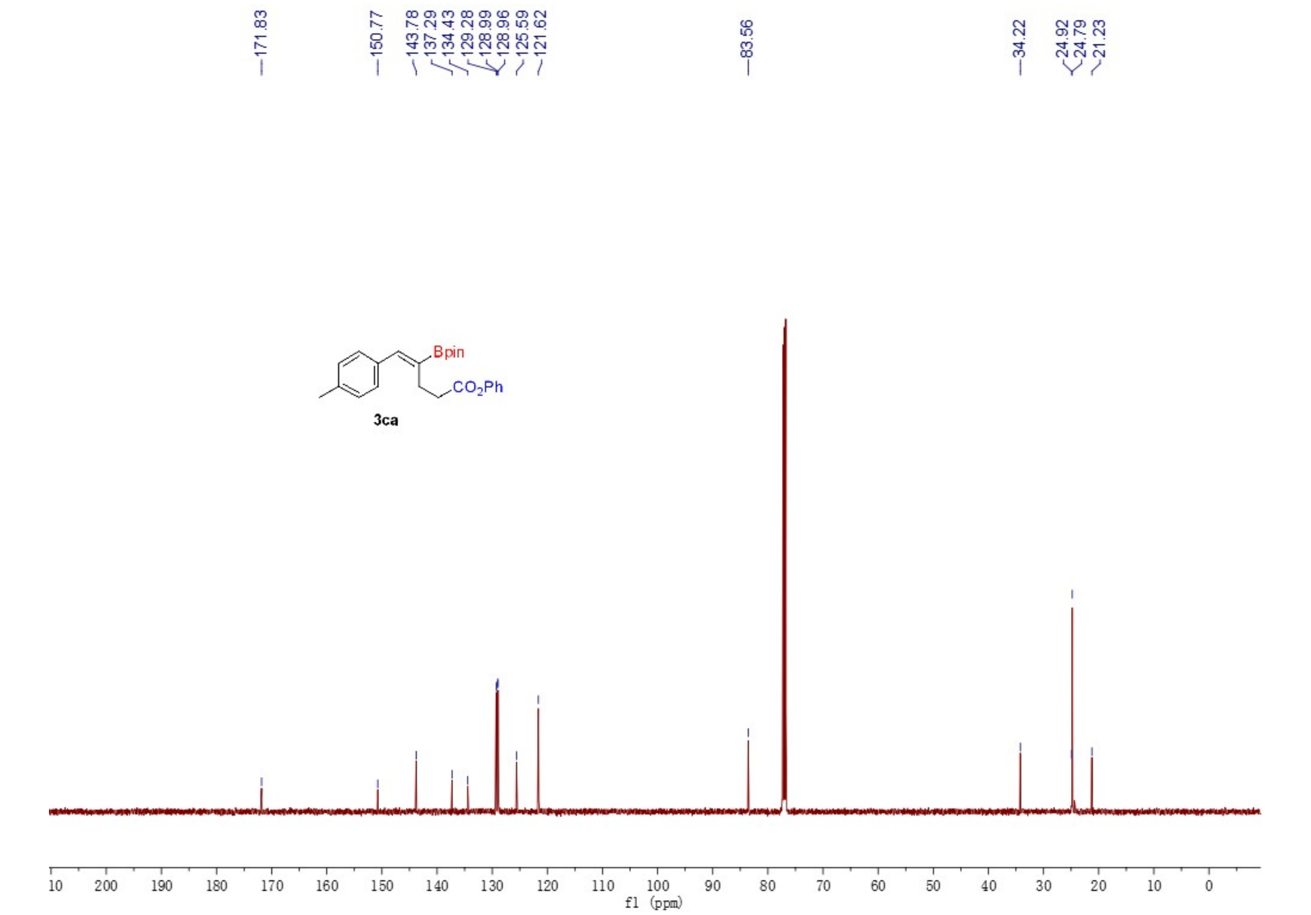
171.83
150.77
143.78
137.29
134.43
129.28
128.99
128.96
125.59
121.62
83.56
34.22
24.92
24.79
21.23
Bpin
CO2Ph
3ca
f1 (ppm)

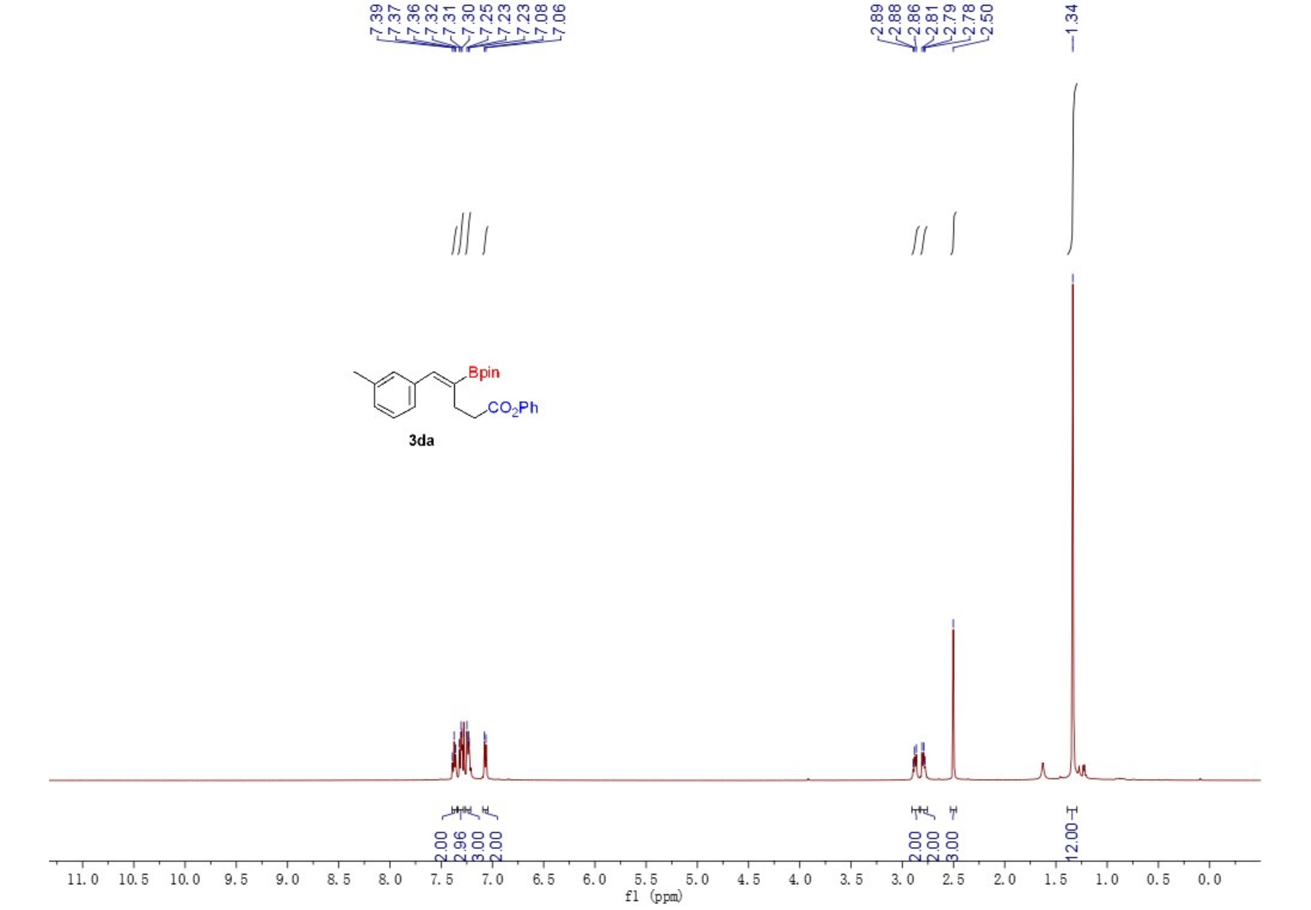
7.39
7.37
7.36
7.32
7.31
7.30
7.25
7.23
7.23
7.08
7.06
2.89
2.88
2.86
2.81
2.79
2.78
2.50
1.34
Bpin
CO2Ph
3da
2.00
2.96
3.00
2.00
2.00
2.00
3.00
12.00
f1 (ppm)

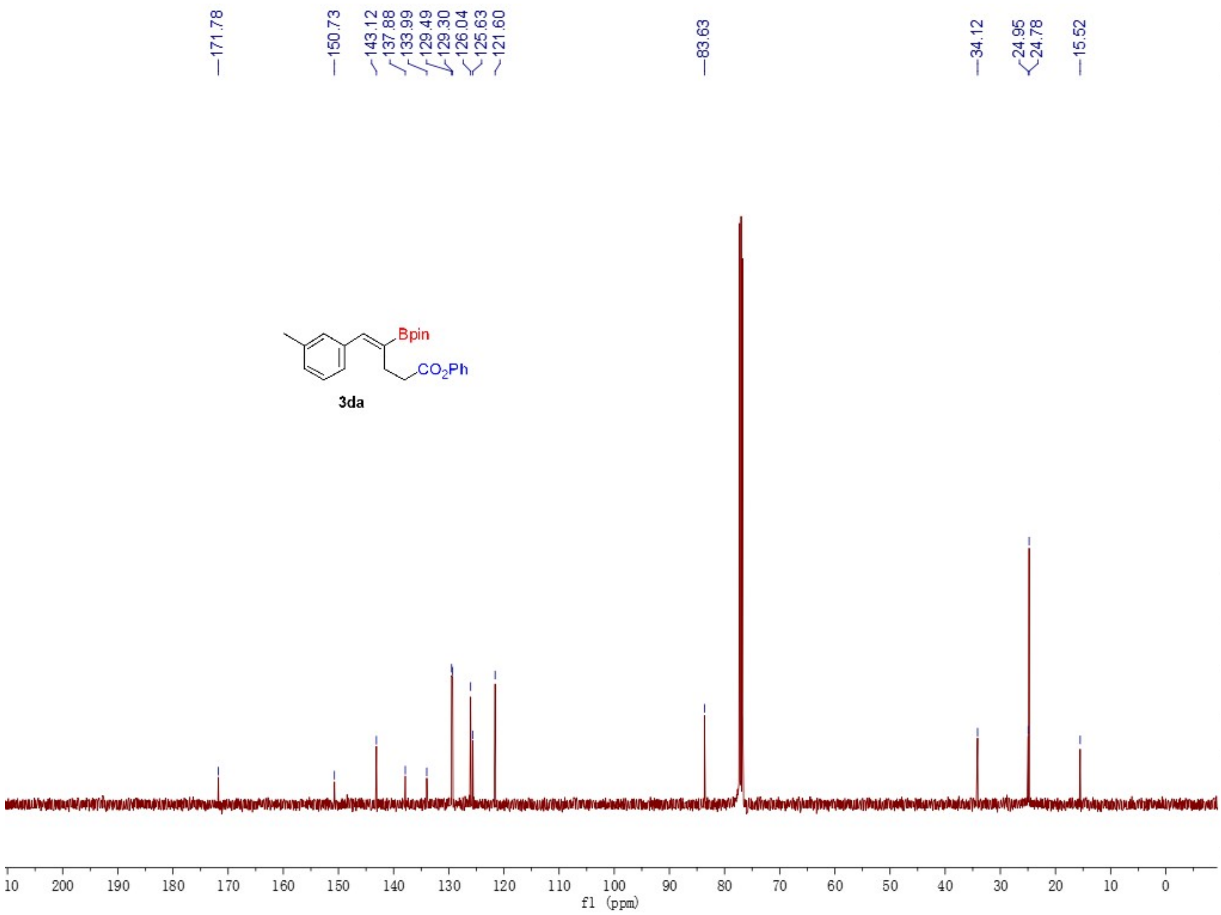

171.78
150.73
143.12
137.88
133.99
129.49
129.30
126.04
125.63
121.60
83.63
34.12
24.95
24.78
15.52
Bpin
CO2Ph
3da
f1 (ppm)

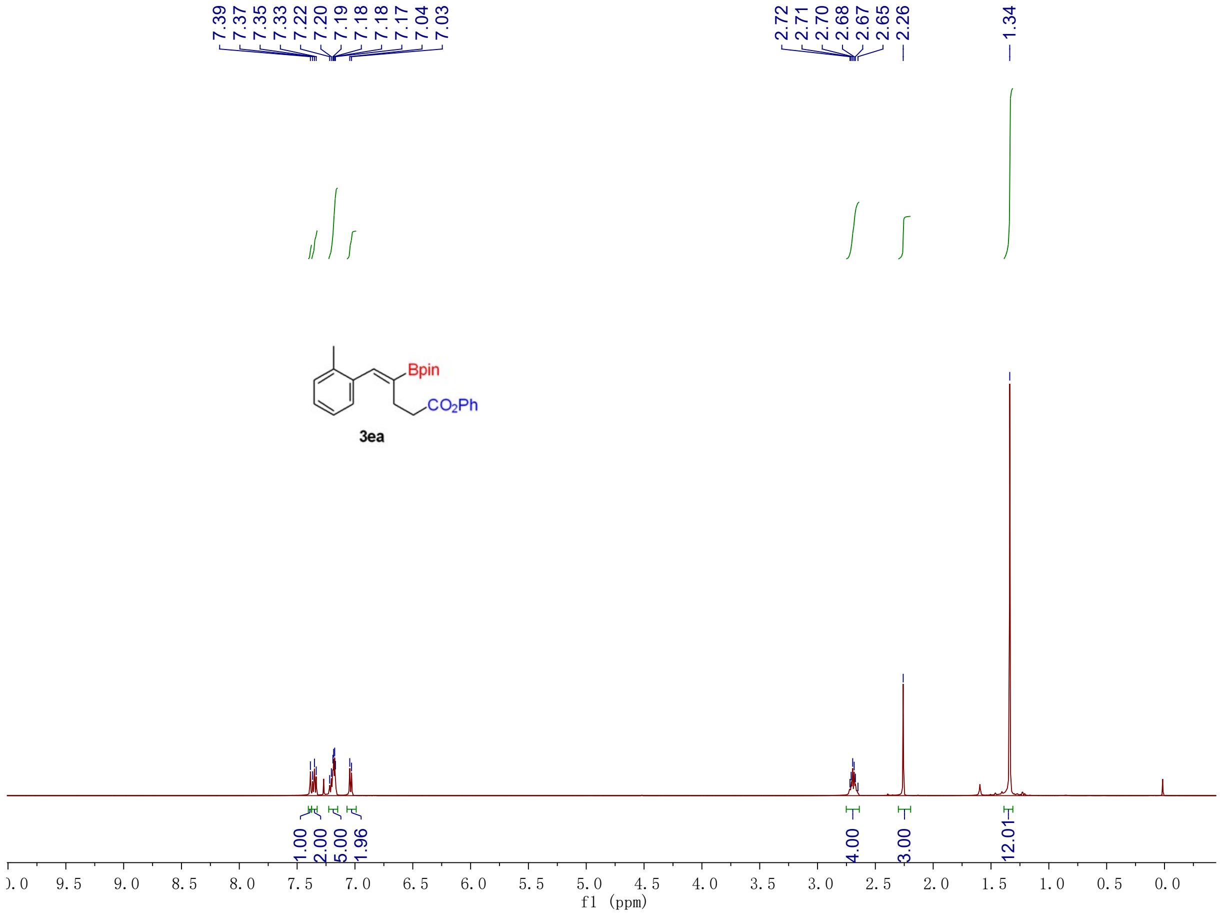

7.39
7.37
7.35
7.33
7.22
7.20
7.19
7.18
7.18
7.17
7.04
7.03
2.72
2.71
2.70
2.68
2.67
2.65
2.26
1.34
Bpin
CO2Ph
3ea
1.00
2.00
5.00
1.96
4.00
3.00
12.01
f1 (ppm)

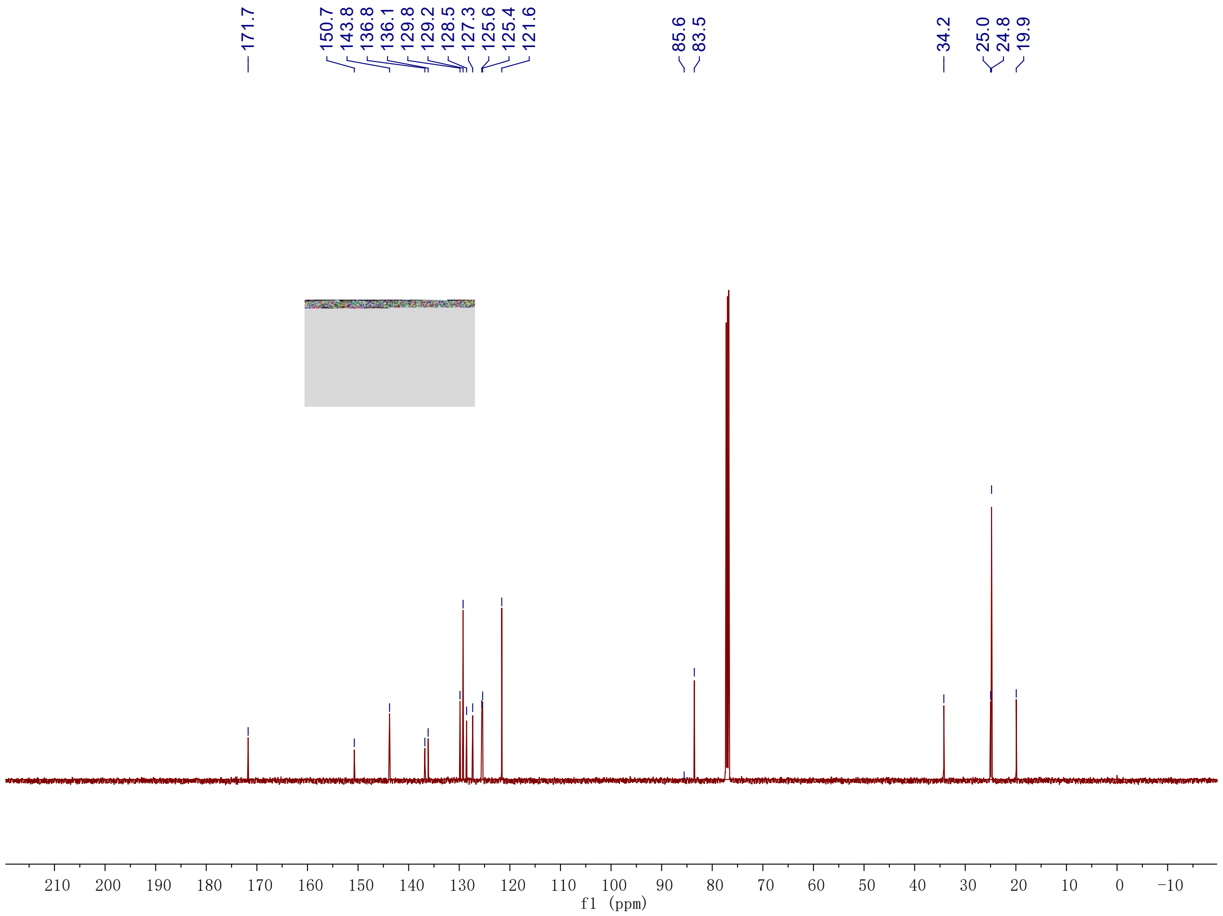
171.7
150.7 143.8 136.8 136.1 129.8 129.2 128.5 127.3 125.6 125.4 121.6
85.6 83.5
34.2
25.0 24.8 19.9
210 200 190 180 170 160 150 140 130 120 110 100 90 80 70 60 50 40 30 20 10 0 -10
f1 (ppm)

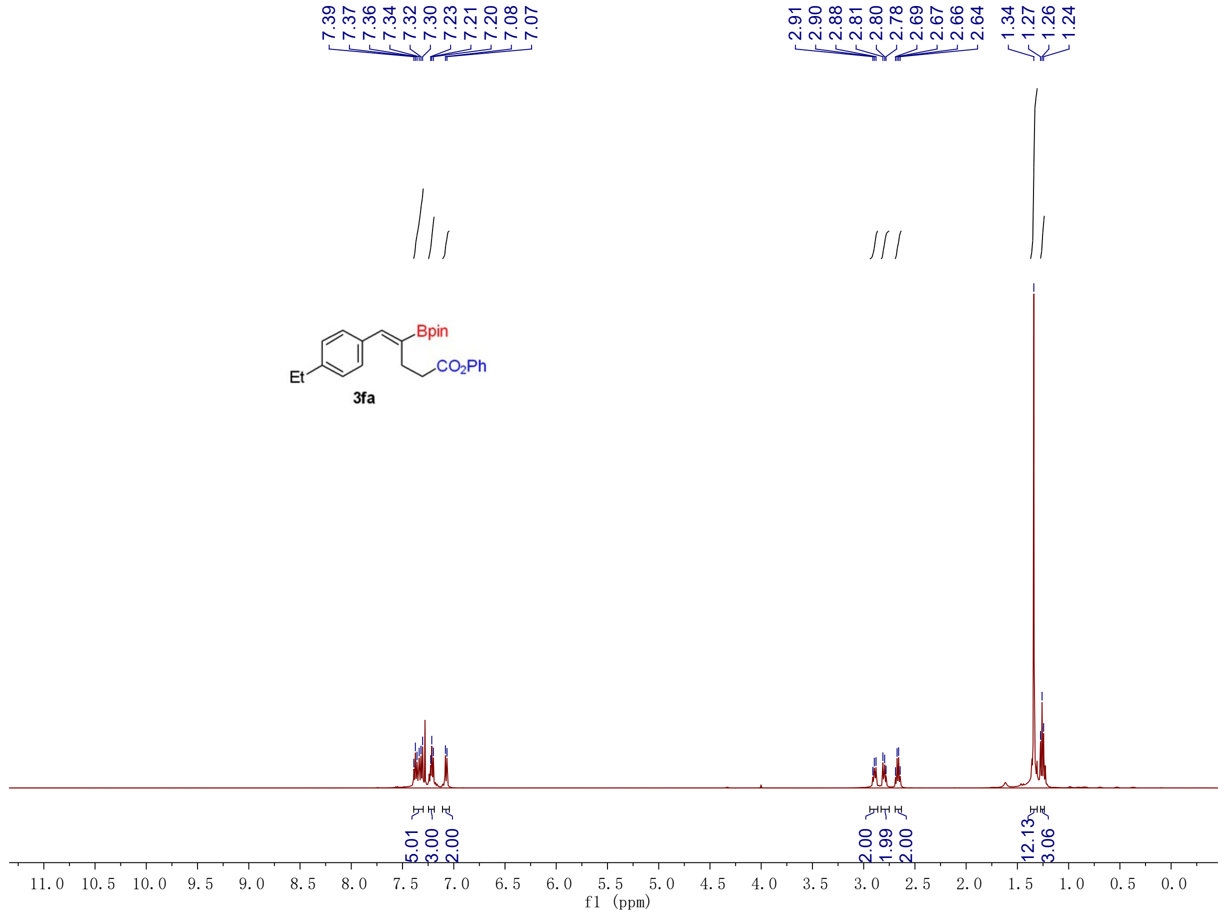
7.39 7.37 7.36 7.34 7.32 7.30 7.23 7.21 7.20 7.08 7.07
2.91 2.90 2.88 2.81 2.80 2.78 2.69 2.67 2.66 2.64
1.34 1.27 1.26 1.24
Bpin
Et
CO2Ph
3fa
5.01 3.00 2.00
2.00 1.99 2.00
12.13 3.06
11.0 10.5 10.0 9.5 9.0 8.5 8.0 7.5 7.0 6.5 6.0 5.5 5.0 4.5 4.0 3.5 3.0 2.5 2.0 1.5 1.0 0.5 0.0
f1 (ppm)

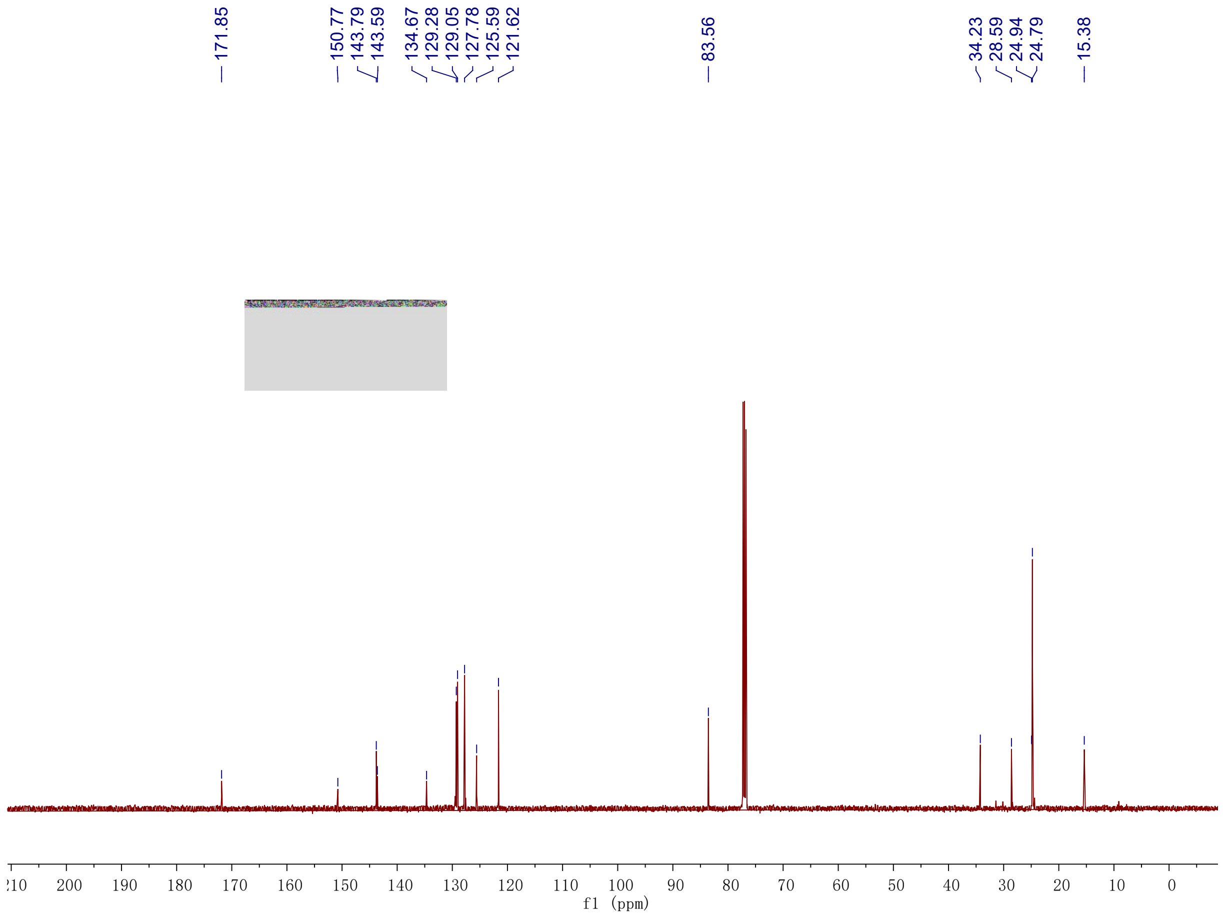
171.85
150.77
143.79
143.59
134.67
129.28
129.05
127.78
125.59
121.62
83.56
34.23
28.59
24.94
24.79
15.38
210 200 190 180 170 160 150 140 130 120 110 100 90 80 70 60 50 40 30 20 10 0
f1 (ppm)

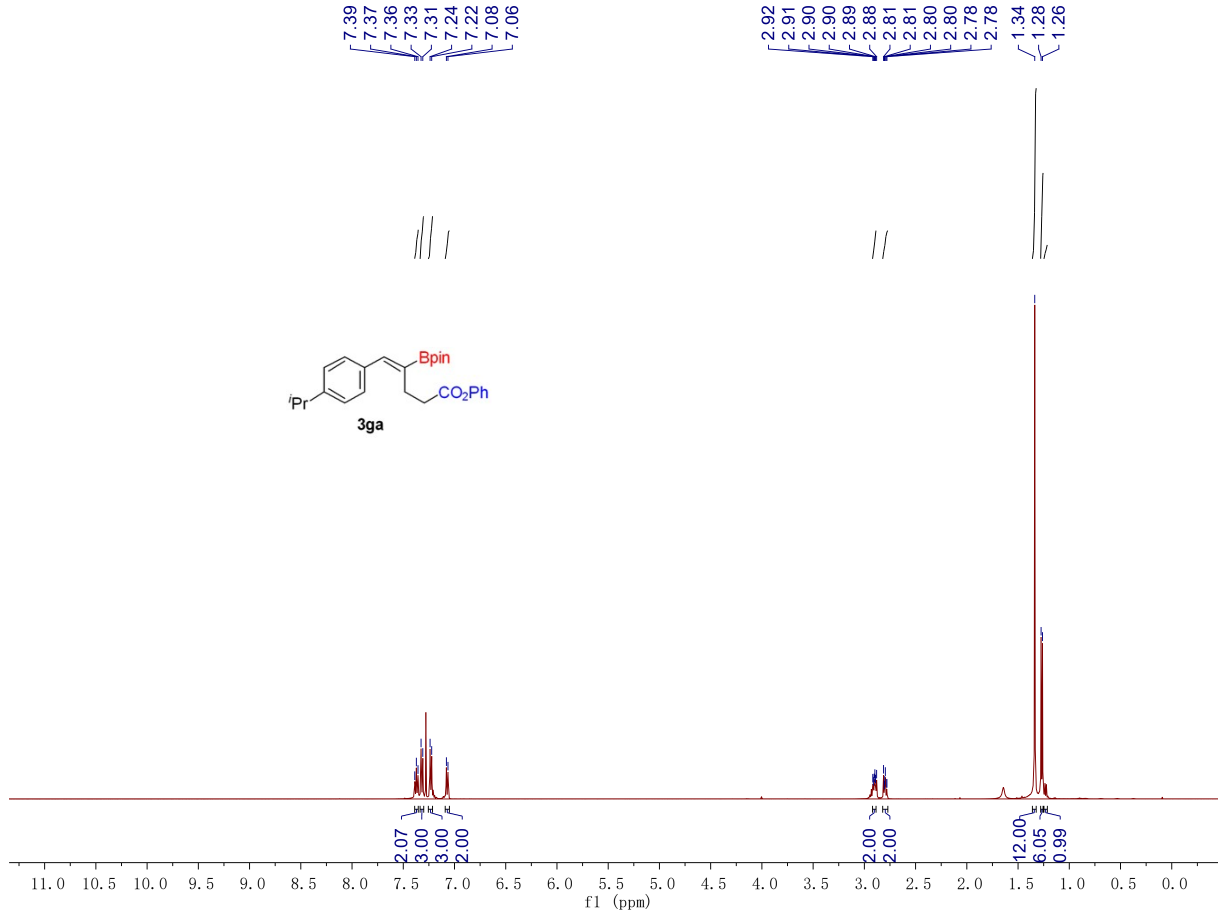
Bpin
CO2Ph
iPr
7.39
7.37
7.36
7.33
7.31
7.24
7.22
7.08
7.06
2.92
2.91
2.90
2.89
2.88
2.81
2.81
2.80
2.80
2.78
2.78
1.34
1.28
1.26
2.07
3.00
3.00
2.00
2.00
2.00
12.00
6.05
0.99
11.0 10.5 10.0 9.5 9.0 8.5 8.0 7.5 7.0 6.5 6.0 5.5 5.0 4.5 4.0 3.5 3.0 2.5 2.0 1.5 1.0 0.5 0.0
f1 (ppm)

3ga

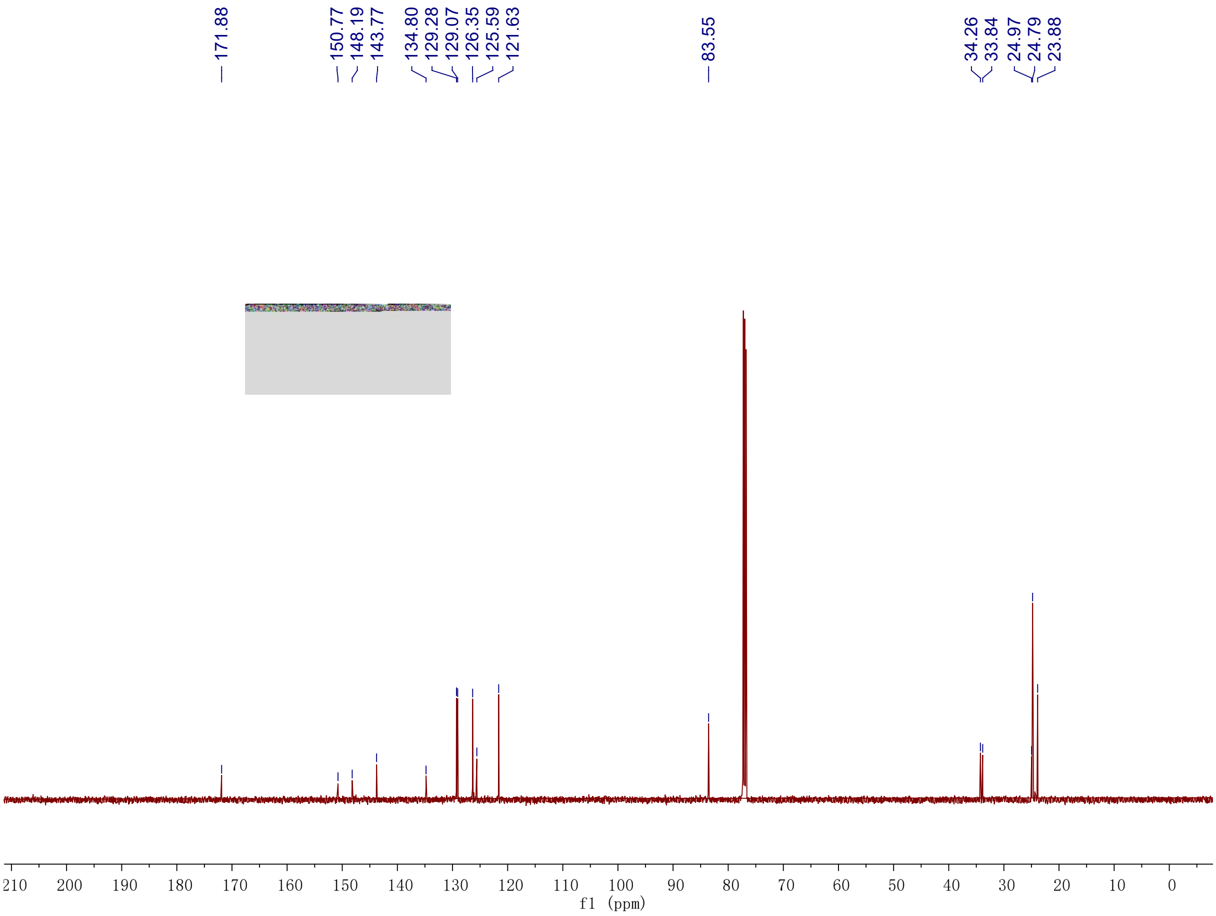
171.88
150.77
148.19
143.77
134.80
129.28
129.07
126.35
125.59
121.63
83.55
34.26
33.84
24.97
24.79
23.88
210 200 190 180 170 160 150 140 130 120 110 100 90 80 70 60 50 40 30 20 10 0
f1 (ppm)

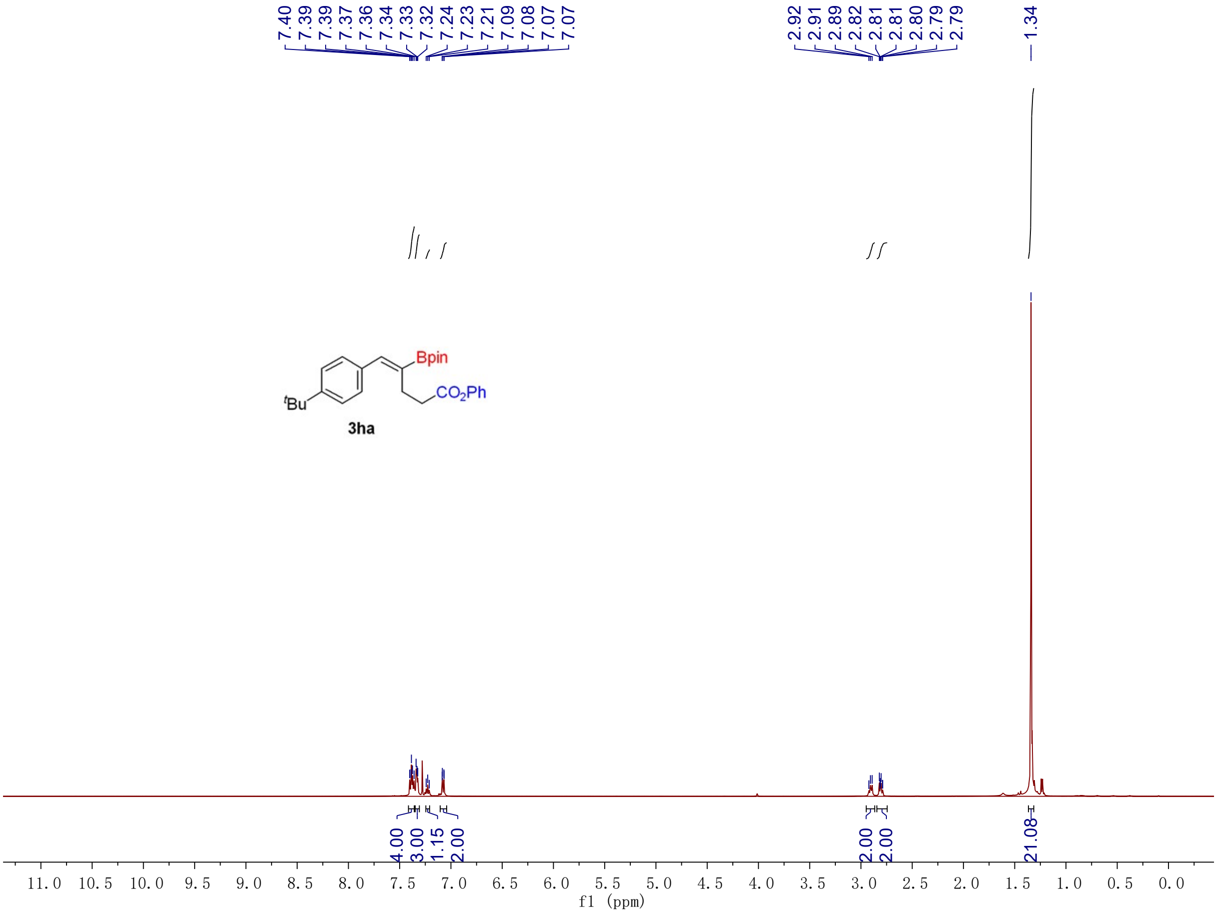
7.40
7.39
7.39
7.37
7.36
7.34
7.33
7.32
7.24
7.23
7.21
7.09
7.08
7.07
7.07
2.92
2.91
2.89
2.82
2.81
2.80
2.79
2.79
1.34
Bpin
CO2Ph
tBu
3ha
4.00
3.00
1.15
2.00
2.00
2.00
21.08
11.0 10.5 10.0 9.5 9.0 8.5 8.0 7.5 7.0 6.5 6.0 5.5 5.0 4.5 4.0 3.5 3.0 2.5 2.0 1.5 1.0 0.5 0.0
f1 (ppm)

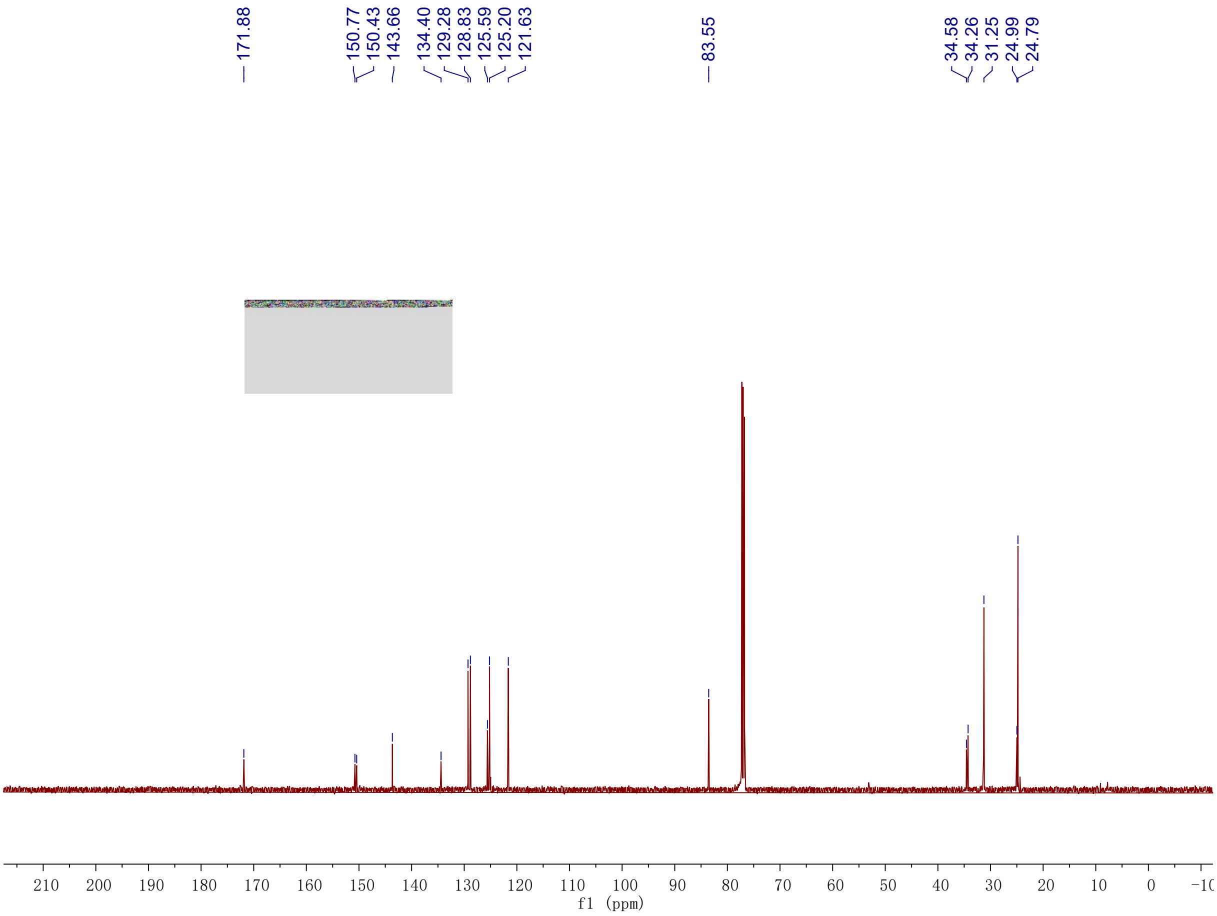

171.88
150.77
150.43
143.66
134.40
129.28
128.83
125.59
125.20
121.63
83.55
34.58
34.26
31.25
24.99
24.79
210 200 190 180 170 160 150 140 130 120 110 100 90 80 70 60 50 40 30 20 10 0
f1 (ppm)

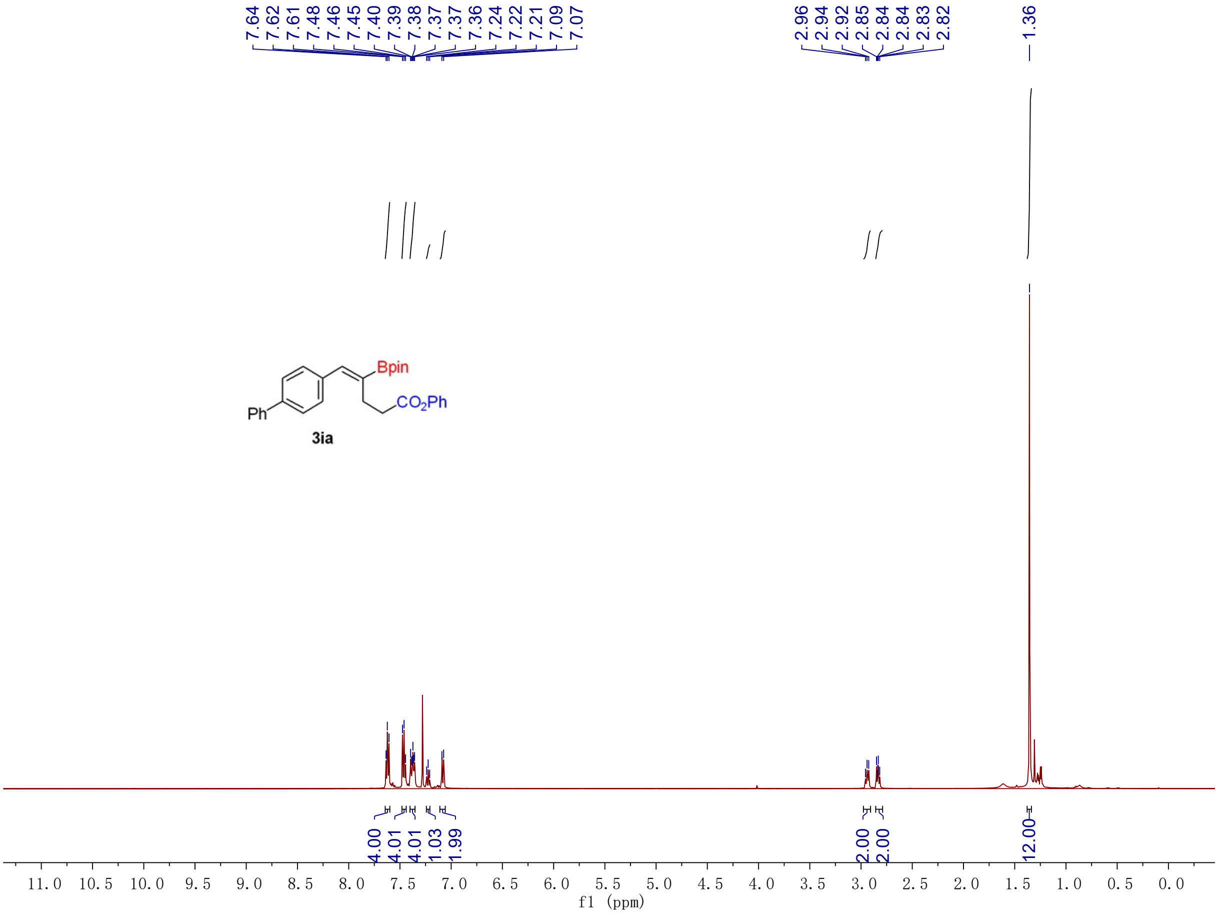

7.64
7.62
7.61
7.48
7.46
7.45
7.40
7.39
7.38
7.37
7.37
7.36
7.24
7.22
7.21
7.09
7.07
2.96
2.94
2.92
2.85
2.84
2.84
2.83
2.82
1.36
4.00
4.01
4.01
1.03
1.99
2.00
2.00
12.00
11.0 10.5 10.0 9.5 9.0 8.5 8.0 7.5 7.0 6.5 6.0 5.5 5.0 4.5 4.0 3.5 3.0 2.5 2.0 1.5 1.0 0.5 0.0
f1 (ppm)
Bpin
Ph
CO2Ph

3ia

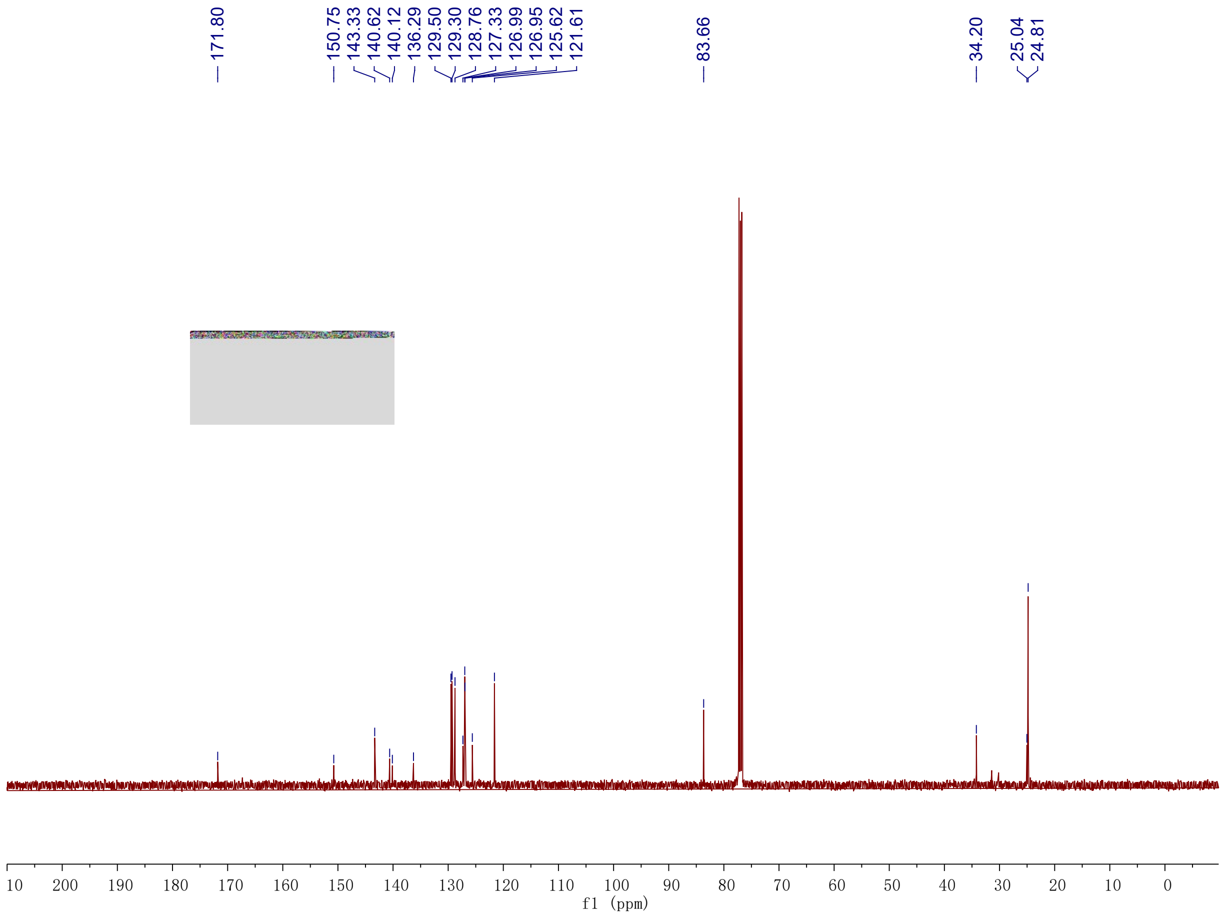

171.80
150.75
143.33
140.62
140.12
136.29
129.50
129.30
128.76
127.33
126.99
126.95
125.62
121.61
83.66
34.20
25.04
24.81
f1 (ppm)

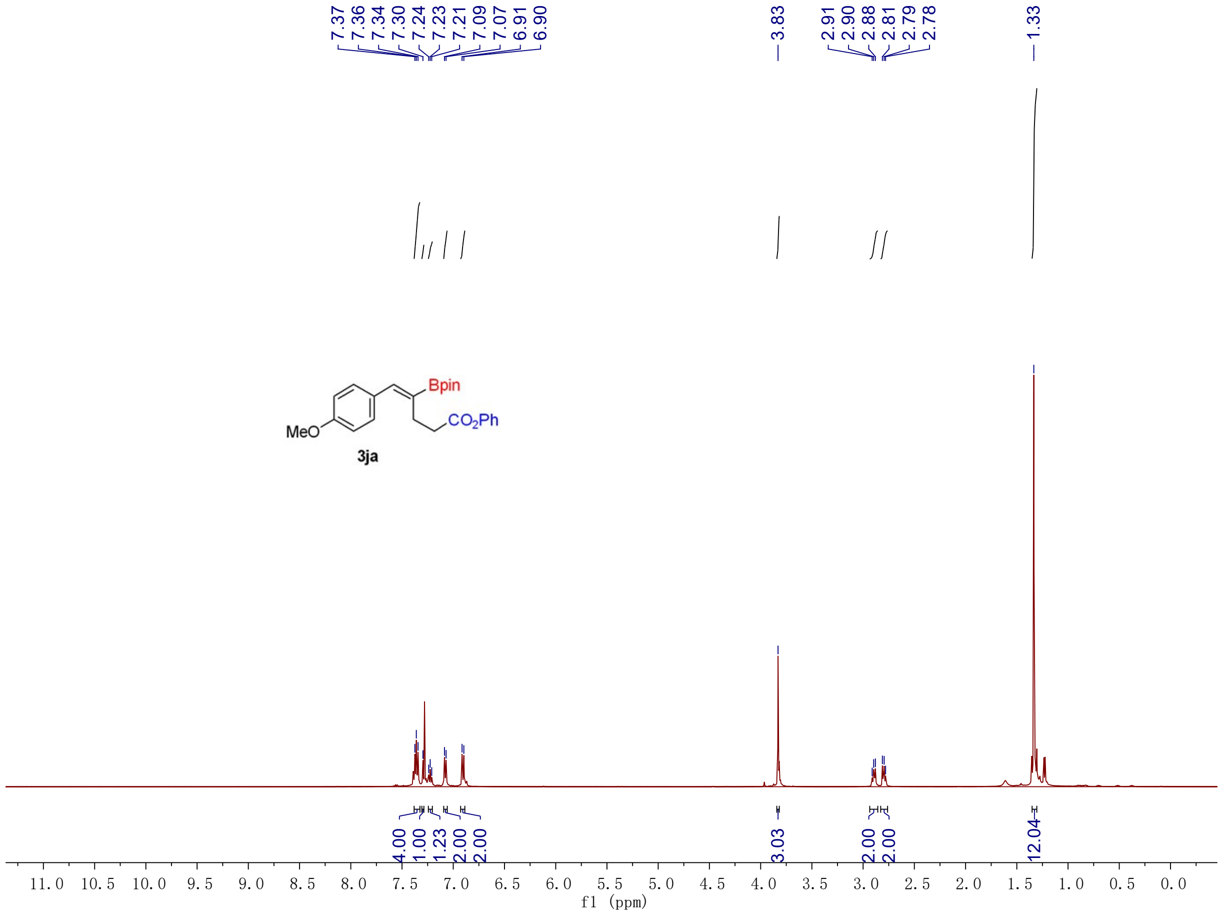

7.37
7.36
7.34
7.30
7.24
7.23
7.21
7.09
7.07
6.91
6.90
3.83
2.91
2.90
2.88
2.81
2.79
2.78
1.33
Bpin
MeO
CO2Ph
3ja
4.00
1.00
1.23
2.00
2.00
3.03
2.00
2.00
12.04
f1 (ppm)

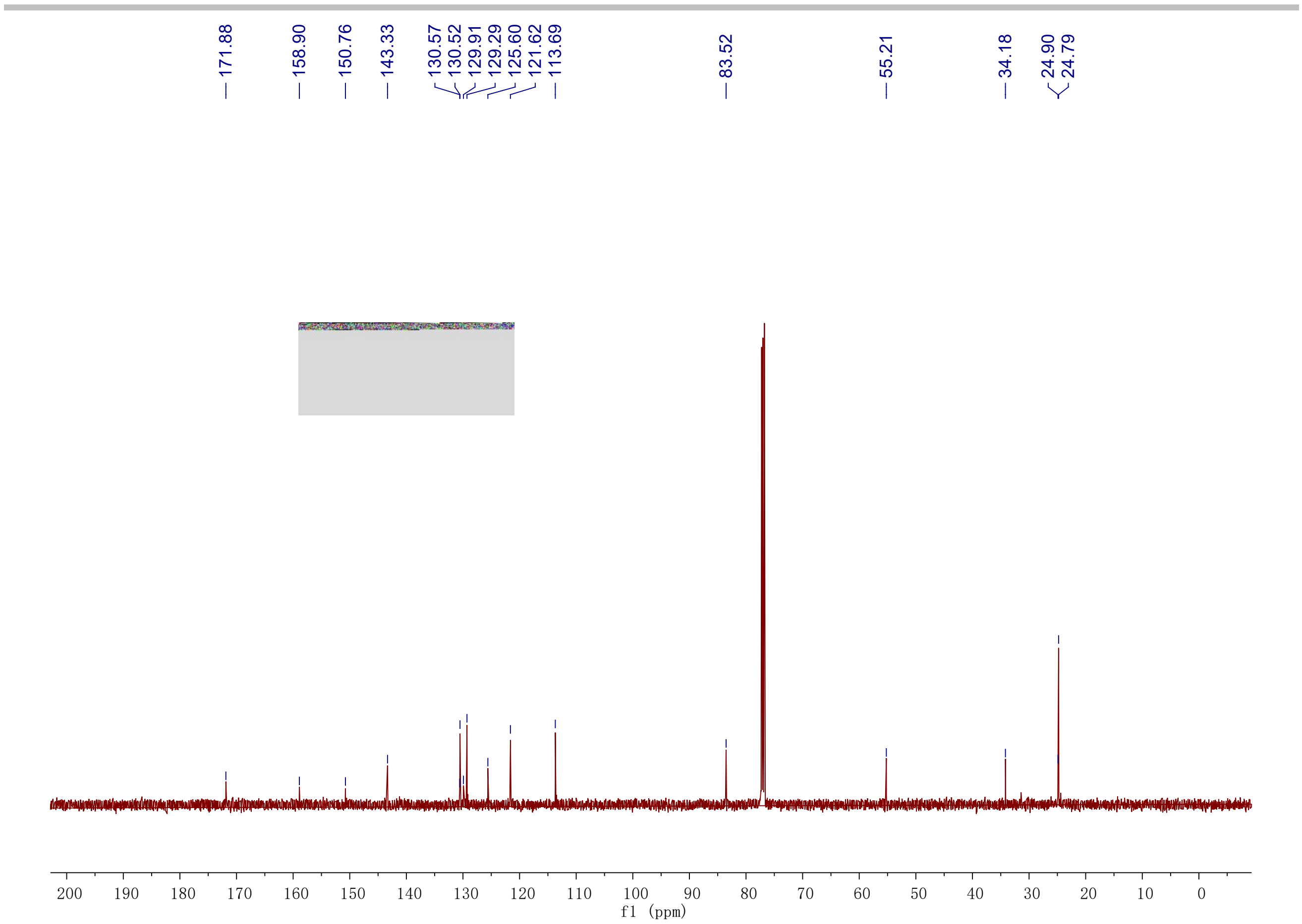
171.88
158.90
150.76
143.33
130.57
130.52
129.91
129.29
125.60
121.62
113.69
83.52
55.21
34.18
24.90
24.79
200 190 180 170 160 150 140 130 120 110 100 90 80 70 60 50 40 30 20 10 0
f1 (ppm)

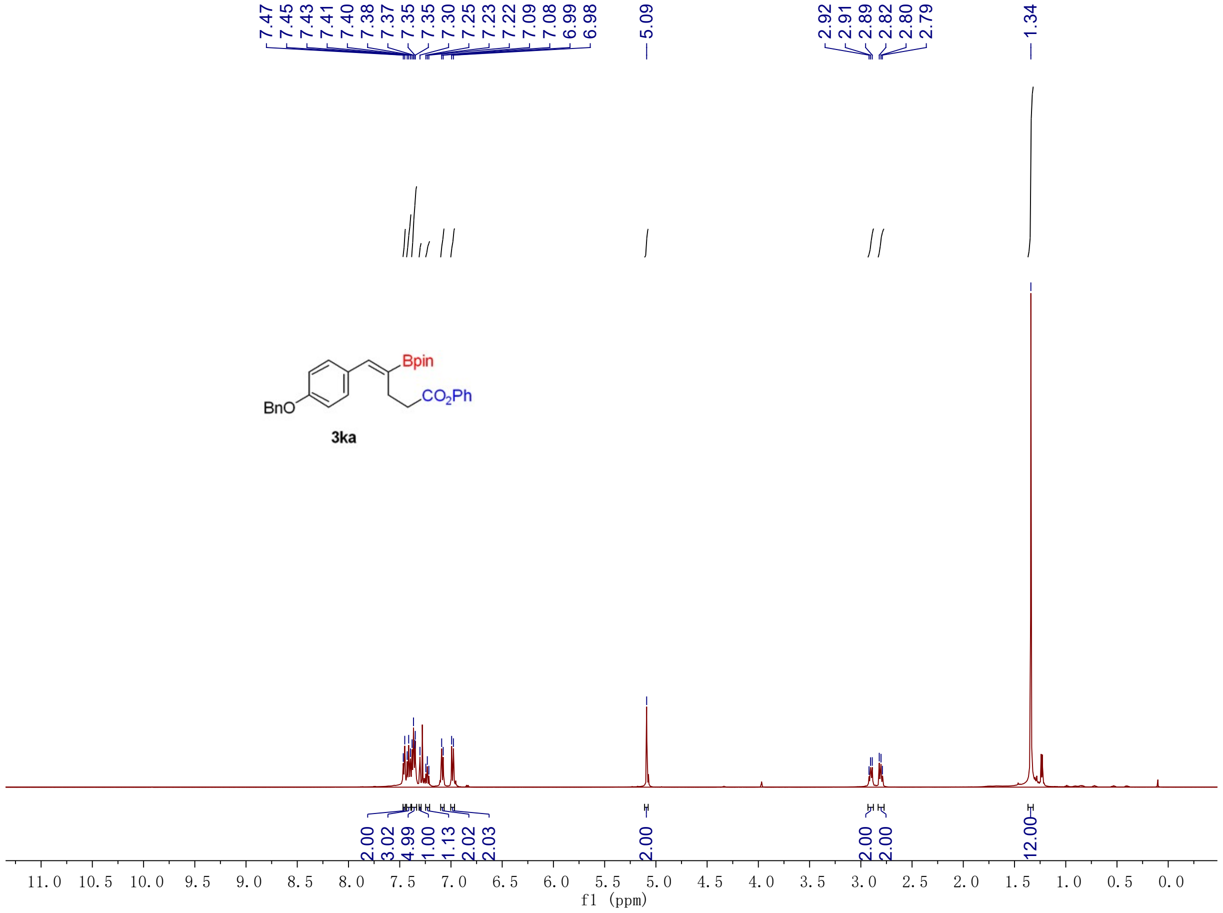
7.47
7.45
7.43
7.41
7.40
7.38
7.37
7.35
7.35
7.30
7.25
7.23
7.22
7.09
7.08
6.99
6.98
5.09
2.92
2.91
2.89
2.82
2.80
2.79
1.34
Bpin
BnO
CO2Ph
3ka
2.00
3.02
4.99
1.00
1.13
2.02
2.03
2.00
2.00
2.00
12.00
11.0 10.5 10.0 9.5 9.0 8.5 8.0 7.5 7.0 6.5 6.0 5.5 5.0 4.5 4.0 3.5 3.0 2.5 2.0 1.5 1.0 0.5 0.0
f1 (ppm)

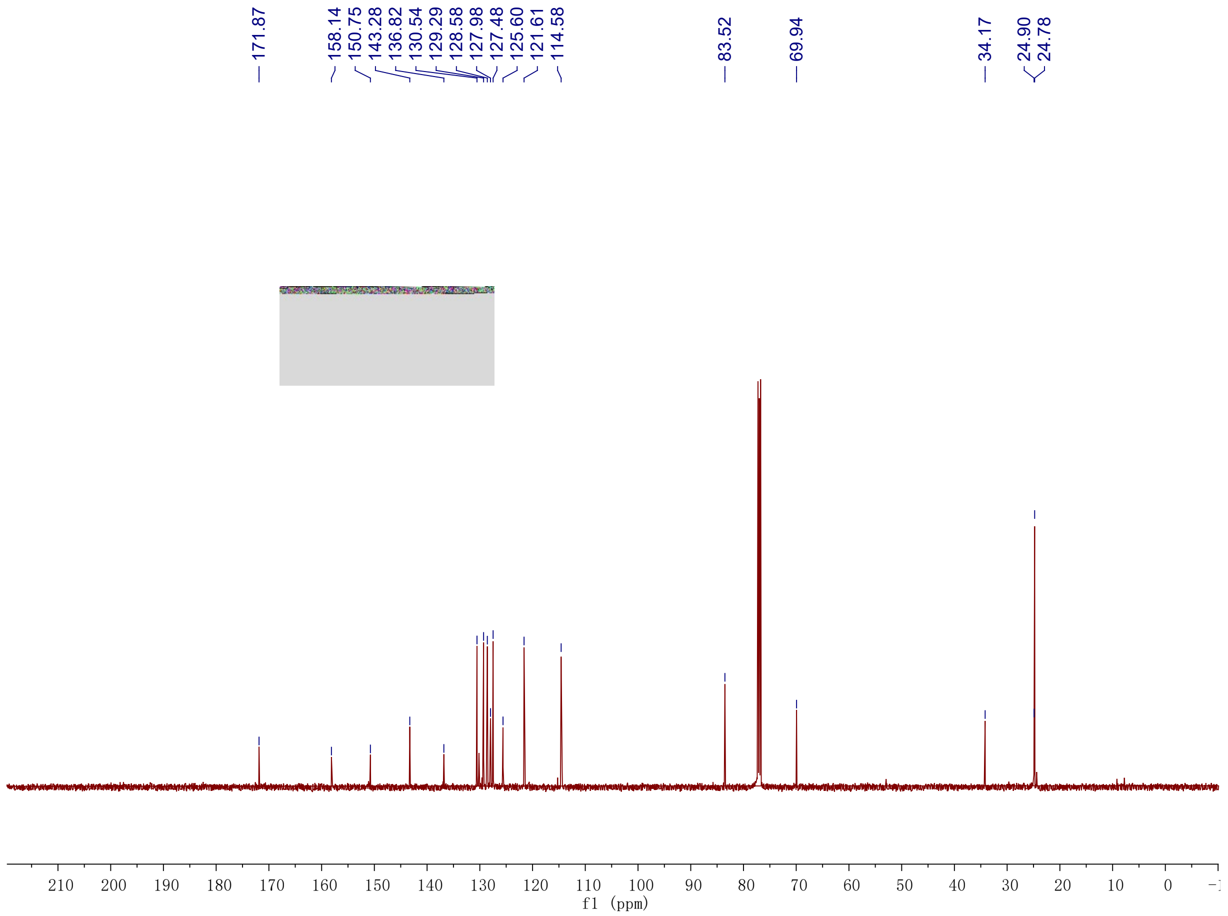
171.87
158.14
150.75
143.28
136.82
130.54
129.29
128.58
127.98
127.48
125.60
121.61
114.58
83.52
69.94
34.17
24.90
24.78
f1 (ppm)

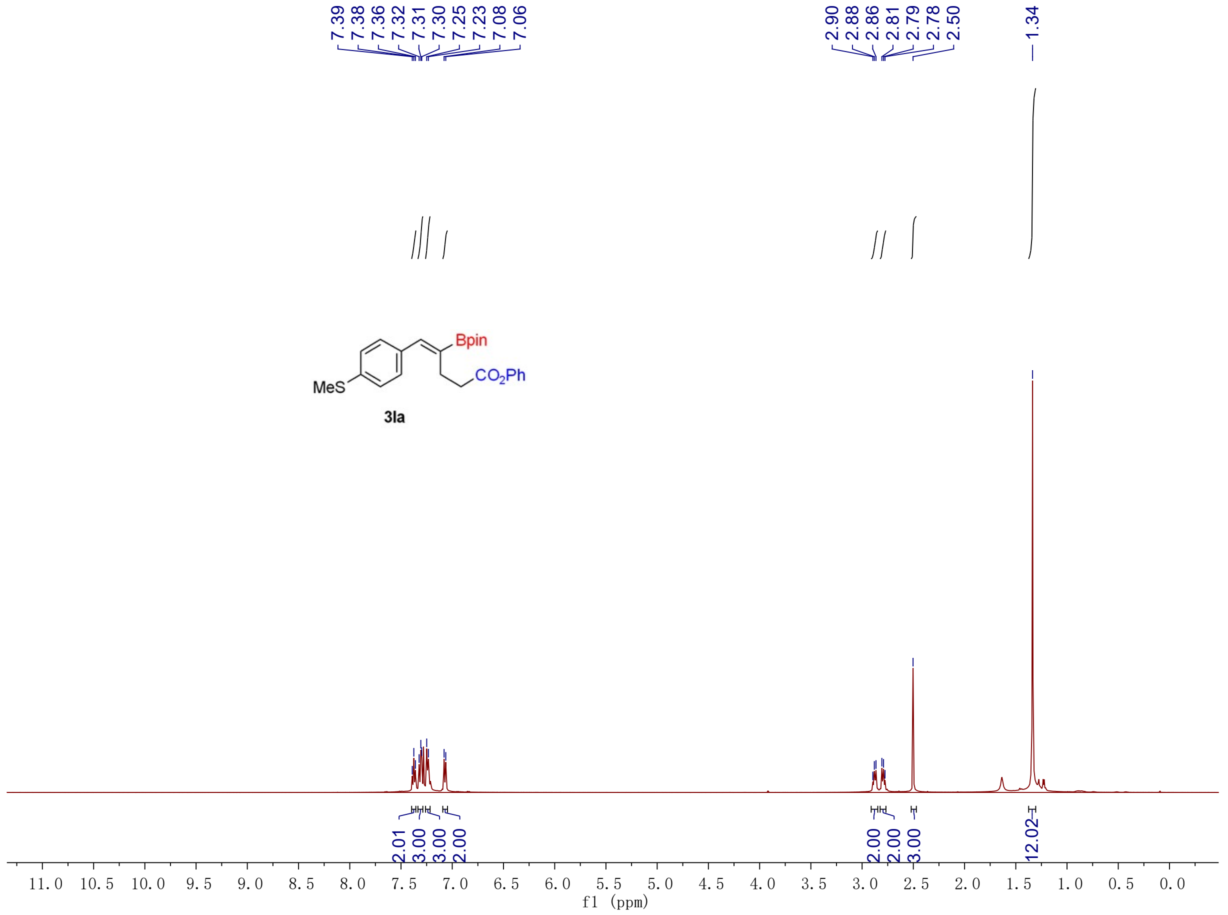
7.39
7.38
7.36
7.32
7.31
7.30
7.25
7.23
7.08
7.06
2.90
2.88
2.86
2.81
2.79
2.78
2.50
1.34
Bpin
CO2Ph
MeS
3la
2.01
3.00
3.00
2.00
2.00
2.00
3.00
12.02
f1 (ppm)

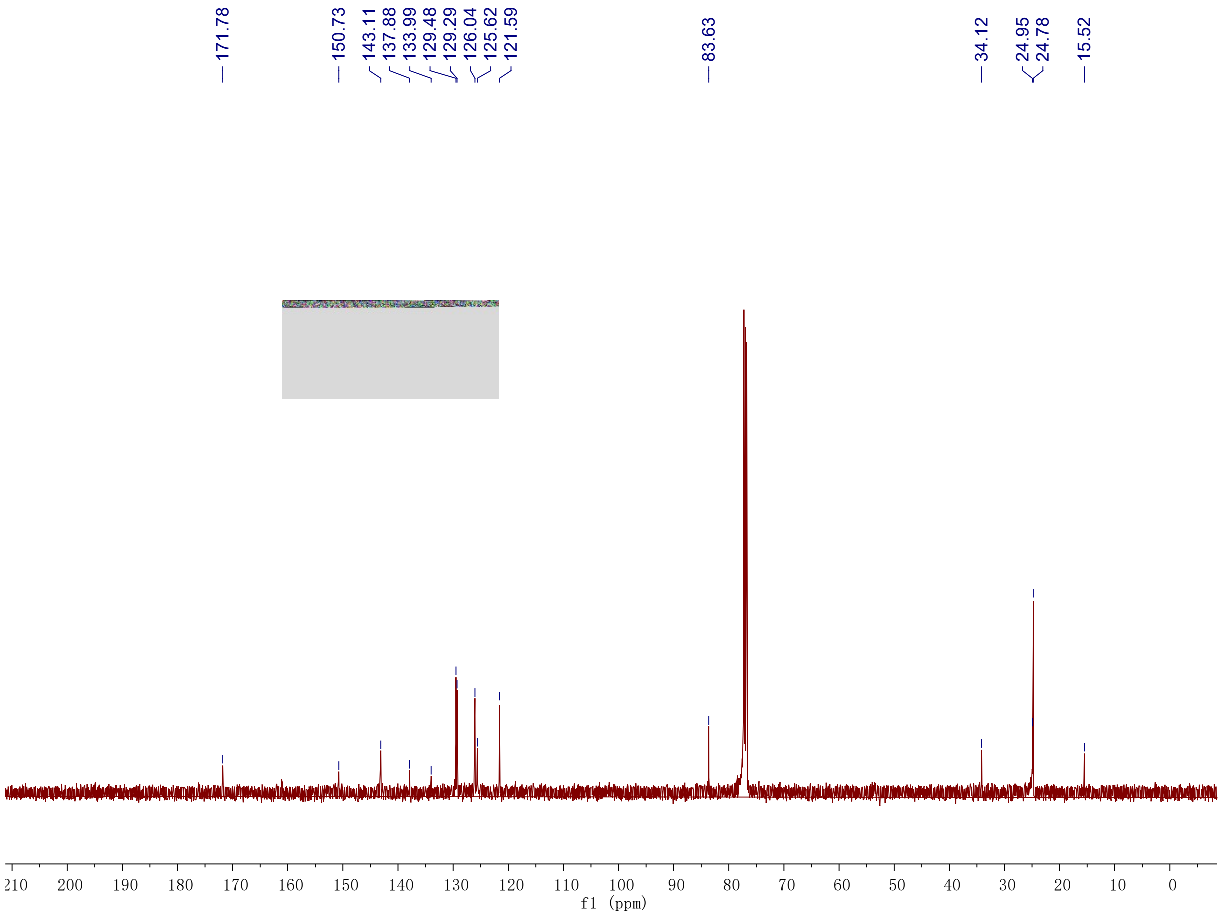
171.78
150.73
143.11
137.88
133.99
129.48
129.29
126.04
125.62
121.59
83.63
34.12
24.95
24.78
15.52
210 200 190 180 170 160 150 140 130 120 110 100 90 80 70 60 50 40 30 20 10 0
f1 (ppm)

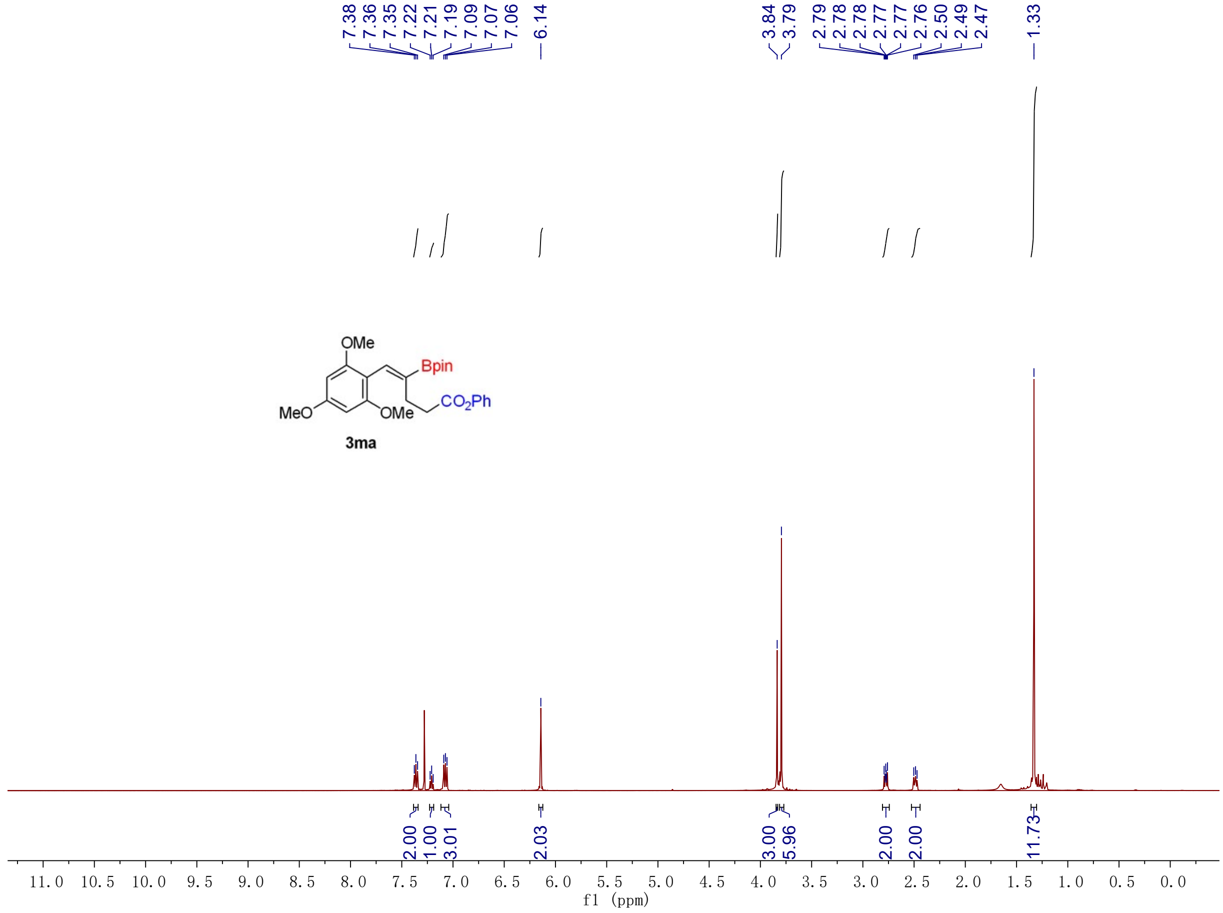
OMe
Bpin
MeO
OMe
CO2Ph
3ma
7.38
7.36
7.35
7.22
7.21
7.19
7.09
7.07
7.06
6.14
3.84
3.79
2.79
2.78
2.78
2.77
2.77
2.76
2.50
2.49
2.47
1.33
2.00
1.00
3.01
2.03
3.00
5.96
2.00
2.00
11.73
11.0 10.5 10.0 9.5 9.0 8.5 8.0 7.5 7.0 6.5 6.0 5.5 5.0 4.5 4.0 3.5 3.0 2.5 2.0 1.5 1.0 0.5 0.0
f1 (ppm)

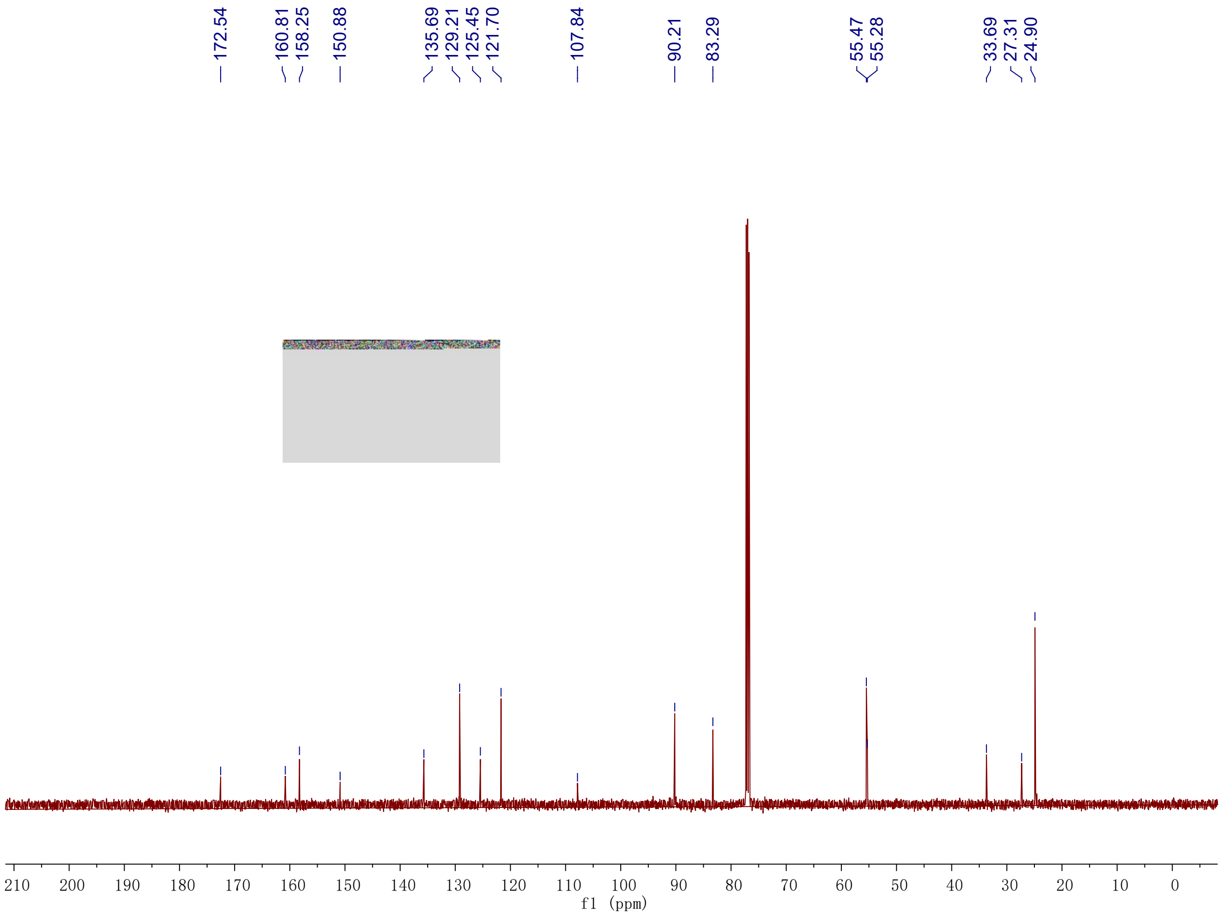
172.54
160.81
158.25
150.88
135.69
129.21
125.45
121.70
107.84
90.21
83.29
55.47
55.28
33.69
27.31
24.90
f1 (ppm)

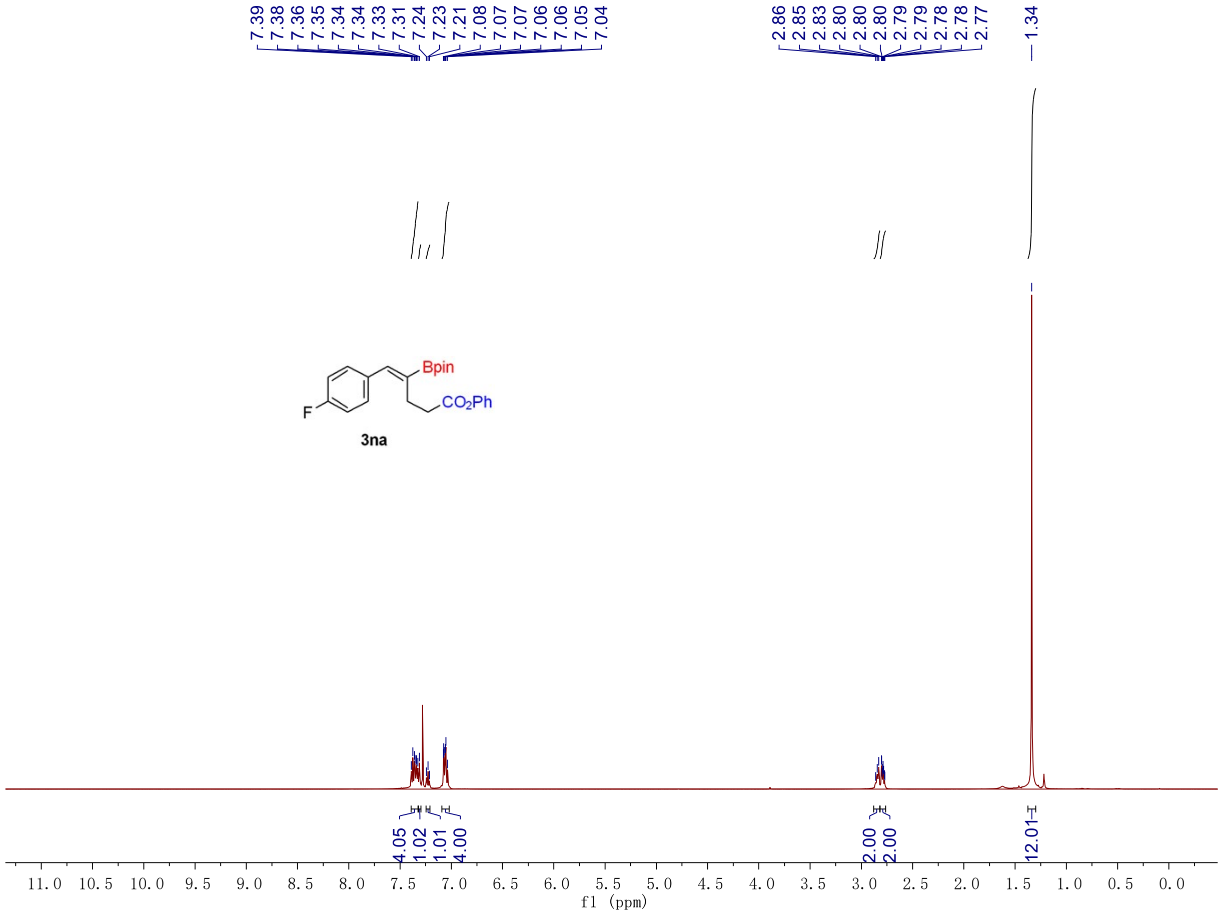
7.39 7.38 7.36 7.35 7.34 7.33 7.31 7.24 7.23 7.21 7.08 7.07 7.07 7.06 7.05 7.04
2.86 2.85 2.83 2.80 2.80 2.80 2.79 2.79 2.78 2.78 2.77
1.34
4.05
1.02
1.01
4.00
2.00
2.00
12.01
f1 (ppm)
Bpin
F
CO2Ph

3na

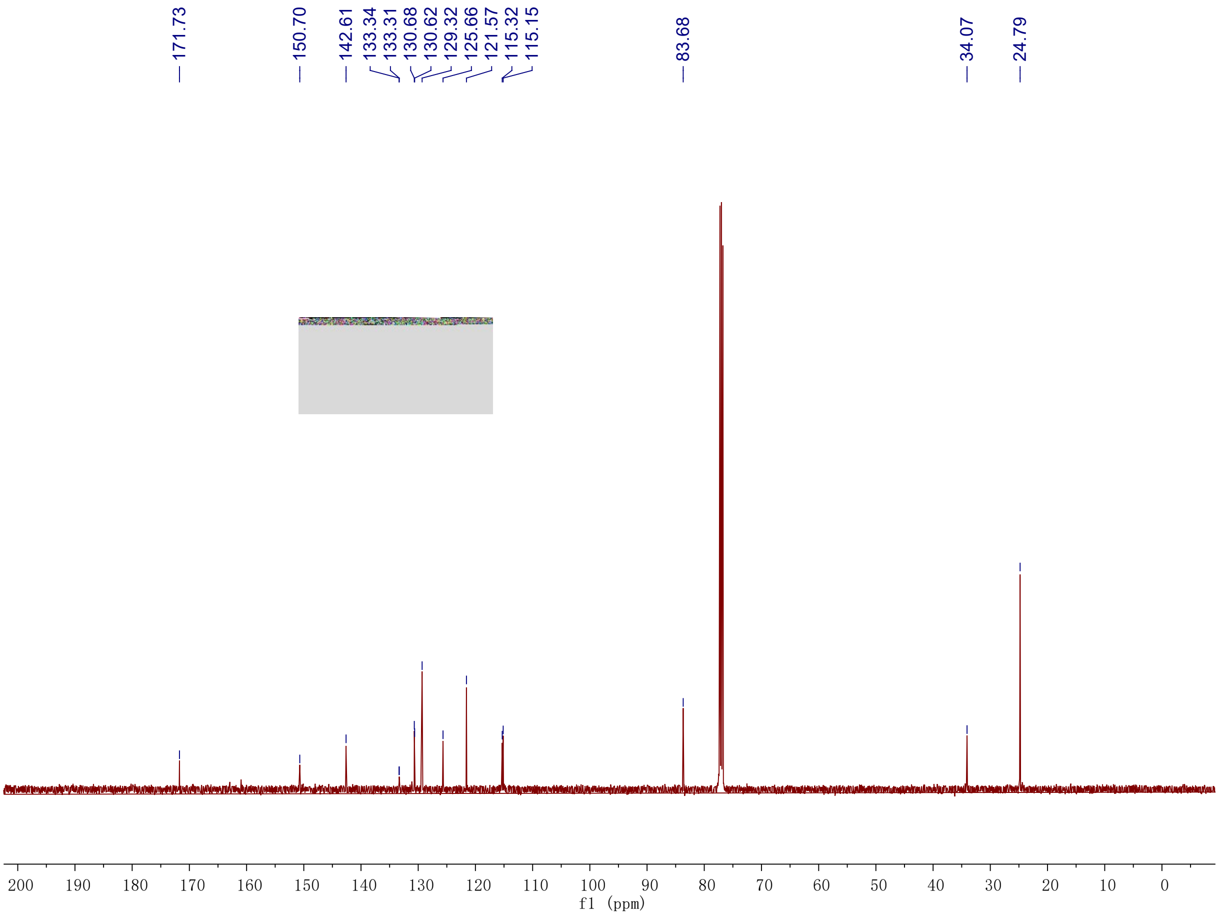
171.73
150.70
142.61
133.34
133.31
130.68
130.62
129.32
125.66
121.57
115.32
115.15
83.68
34.07
24.79
f1 (ppm)

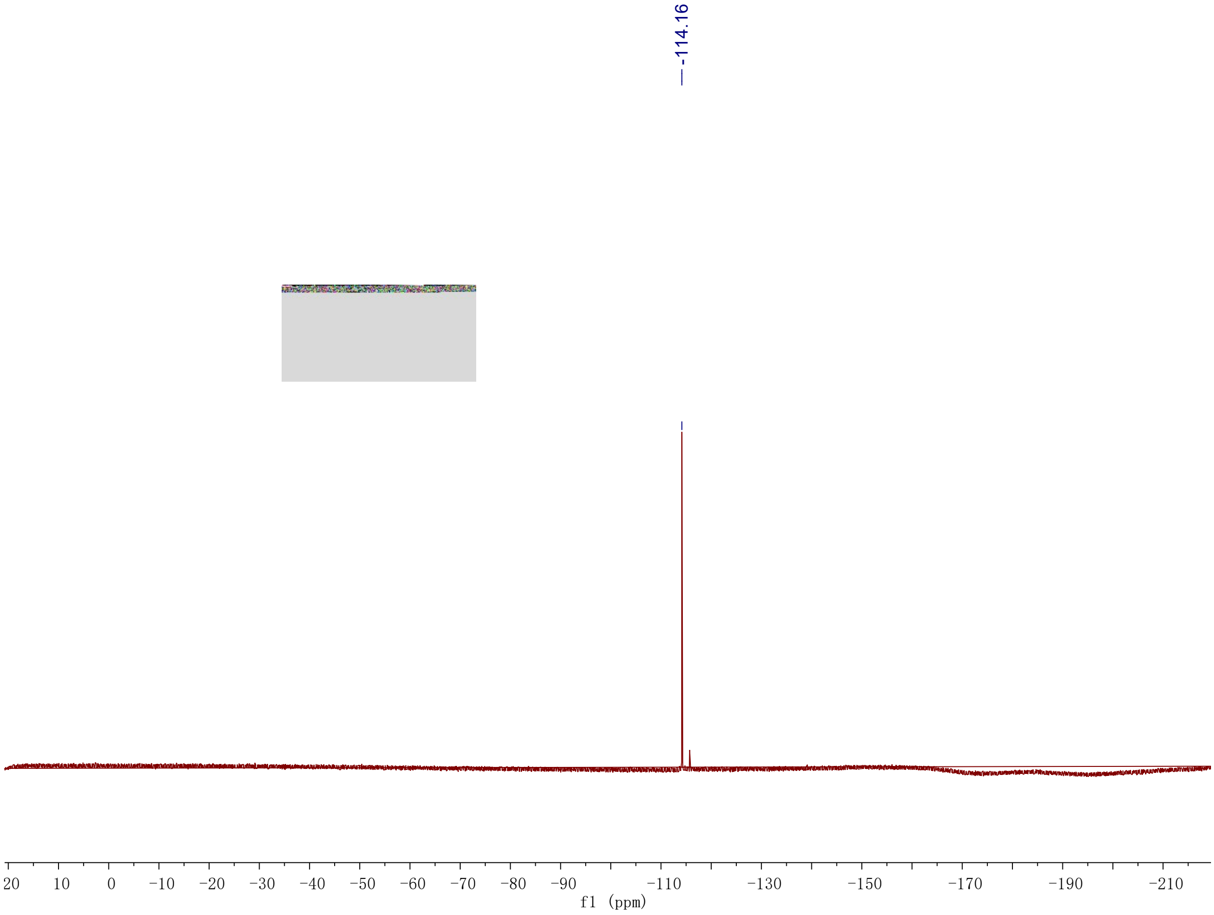
-114.16
f1 (ppm)

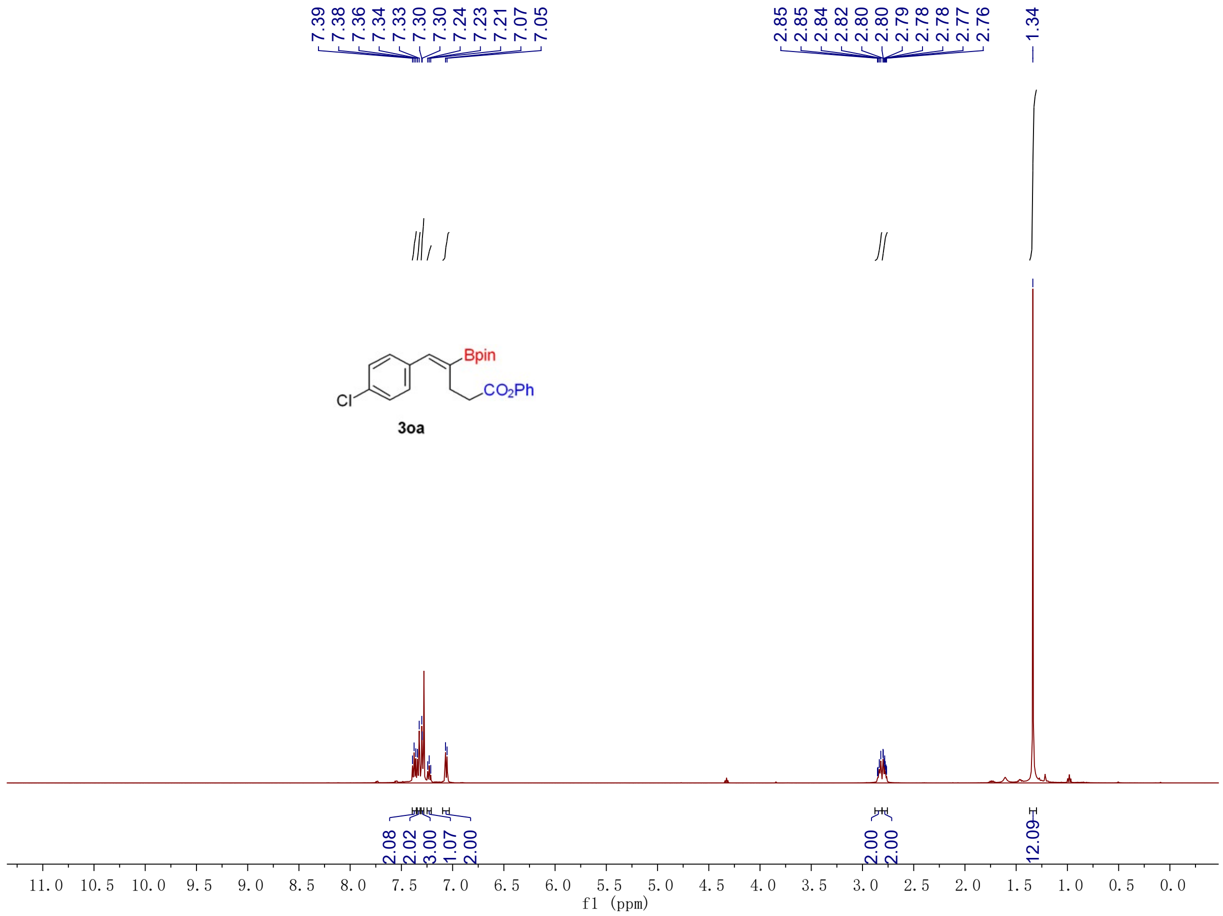
7.39 7.38 7.36 7.34 7.33 7.30 7.30 7.24 7.23 7.21 7.07 7.05
2.85 2.85 2.84 2.82 2.80 2.80 2.79 2.78 2.78 2.77 2.76
1.34
Bpin
Cl
CO2Ph
3oa
2.08 2.02 3.00 1.07 2.00
2.00 2.00
12.09
11.0 10.5 10.0 9.5 9.0 8.5 8.0 7.5 7.0 6.5 6.0 5.5 5.0 4.5 4.0 3.5 3.0 2.5 2.0 1.5 1.0 0.5 0.0
f1 (ppm)

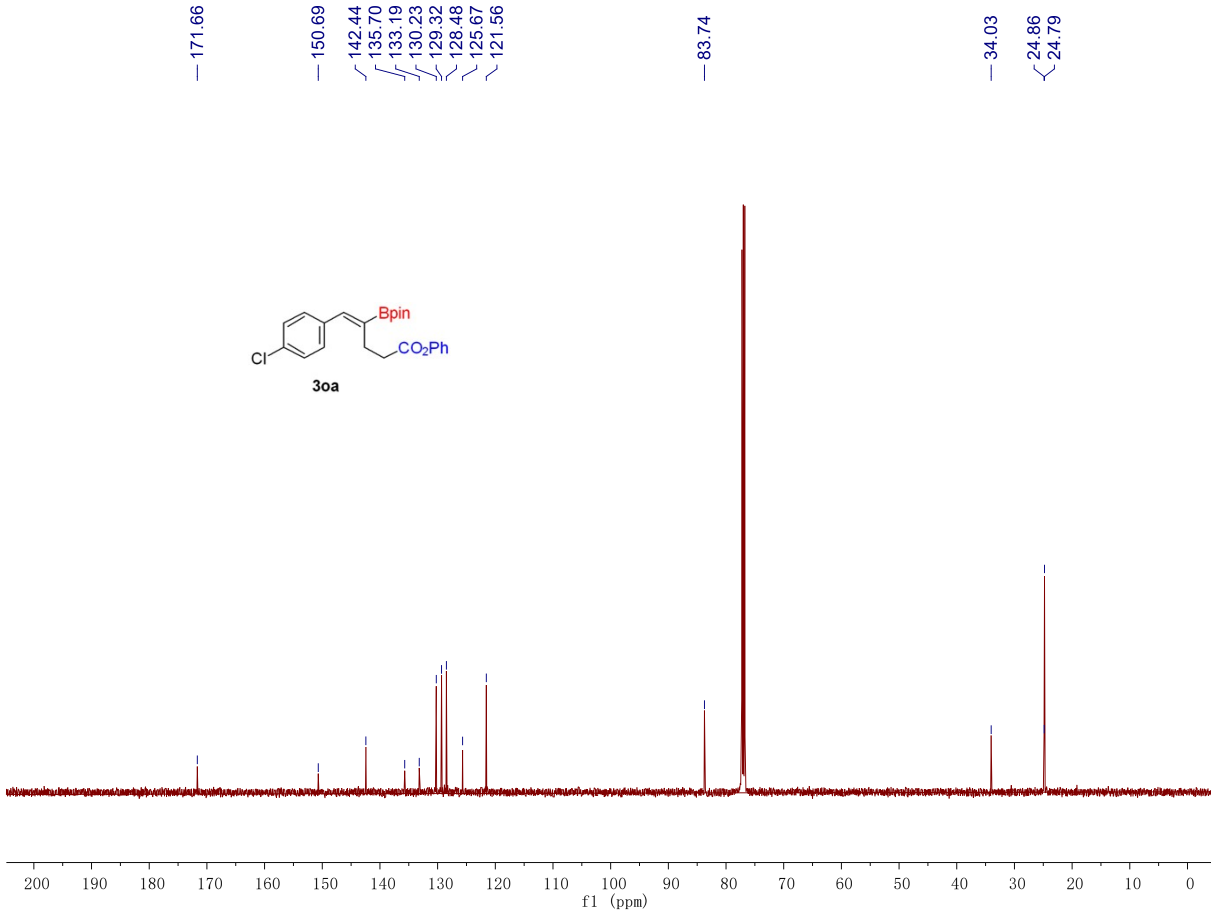
171.66
150.69
142.44 135.70 133.19 130.23 129.32 128.48 125.67 121.56
83.74
34.03
24.86 24.79
Bpin
Cl
CO2Ph
3oa
200 190 180 170 160 150 140 130 120 110 100 90 80 70 60 50 40 30 20 10 0
f1 (ppm)

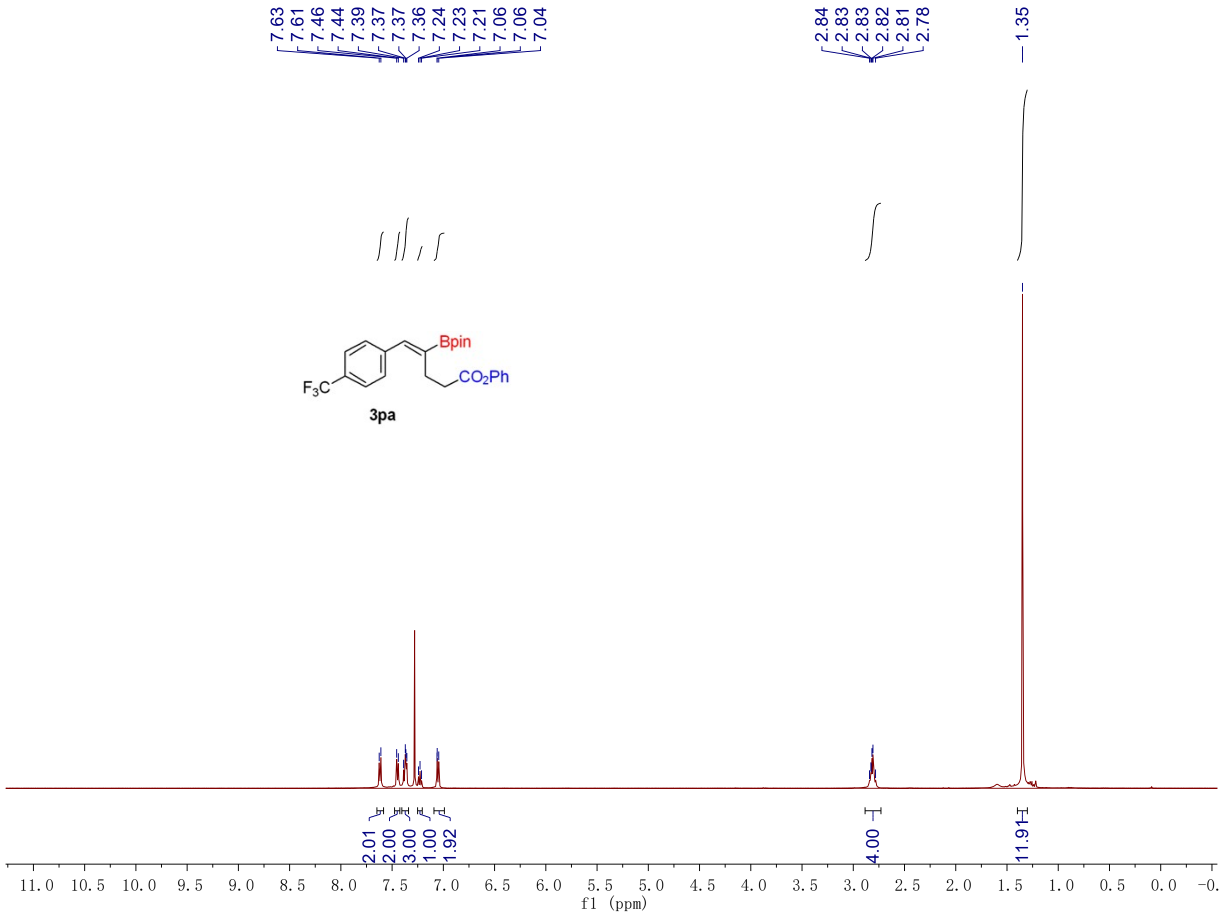
7.63
7.61
7.46
7.44
7.39
7.37
7.37
7.36
7.24
7.23
7.21
7.06
7.06
7.04
2.84
2.83
2.83
2.82
2.81
2.78
1.35
Bpin
CO_2Ph
F_3C
3pa
2.01
2.00
3.00
1.00
1.92
4.00
11.91
f1 (ppm)

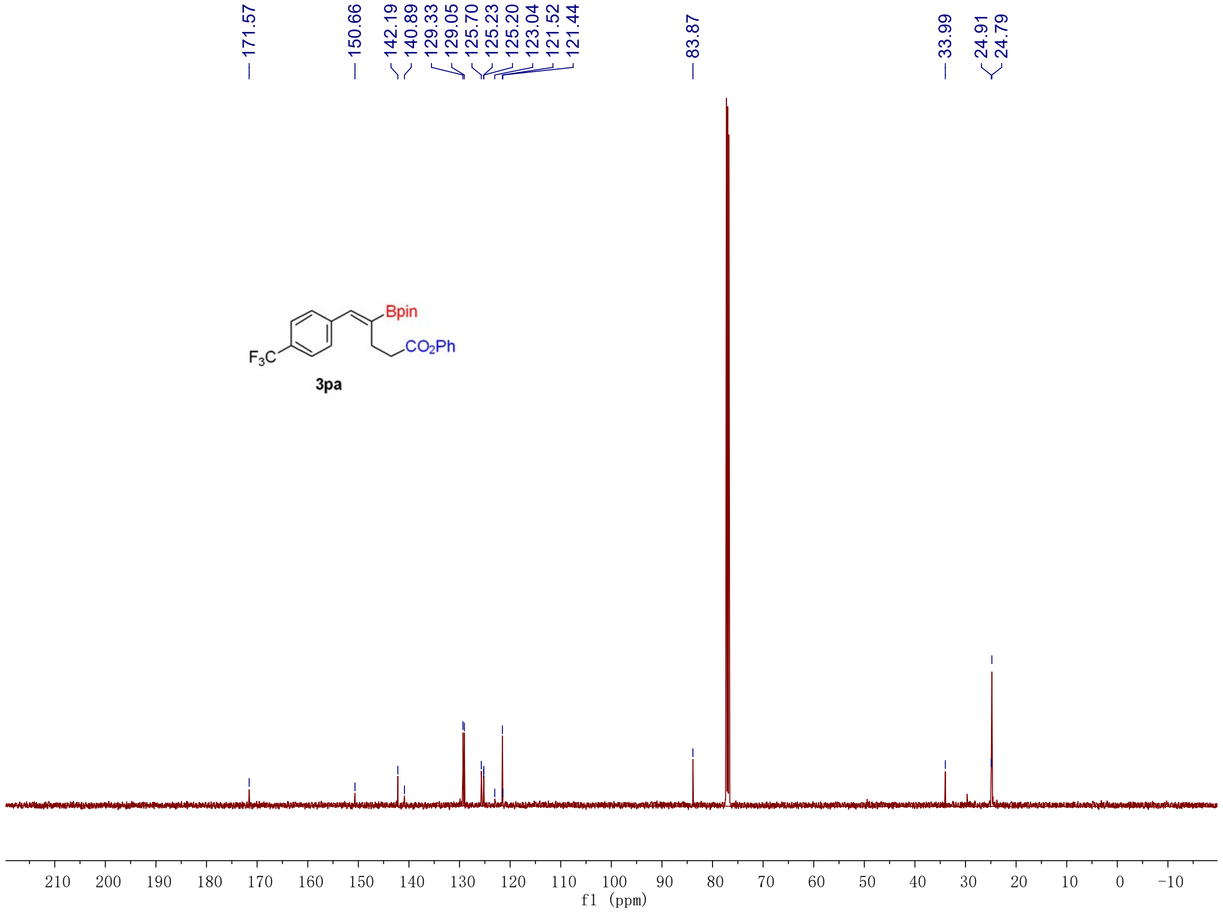
171.57
150.66
142.19
140.89
129.33
129.05
125.70
125.23
125.20
123.04
121.52
121.44
83.87
33.99
24.91
24.79
Bpin
CO_2Ph
F_3C
3pa
f1 (ppm)

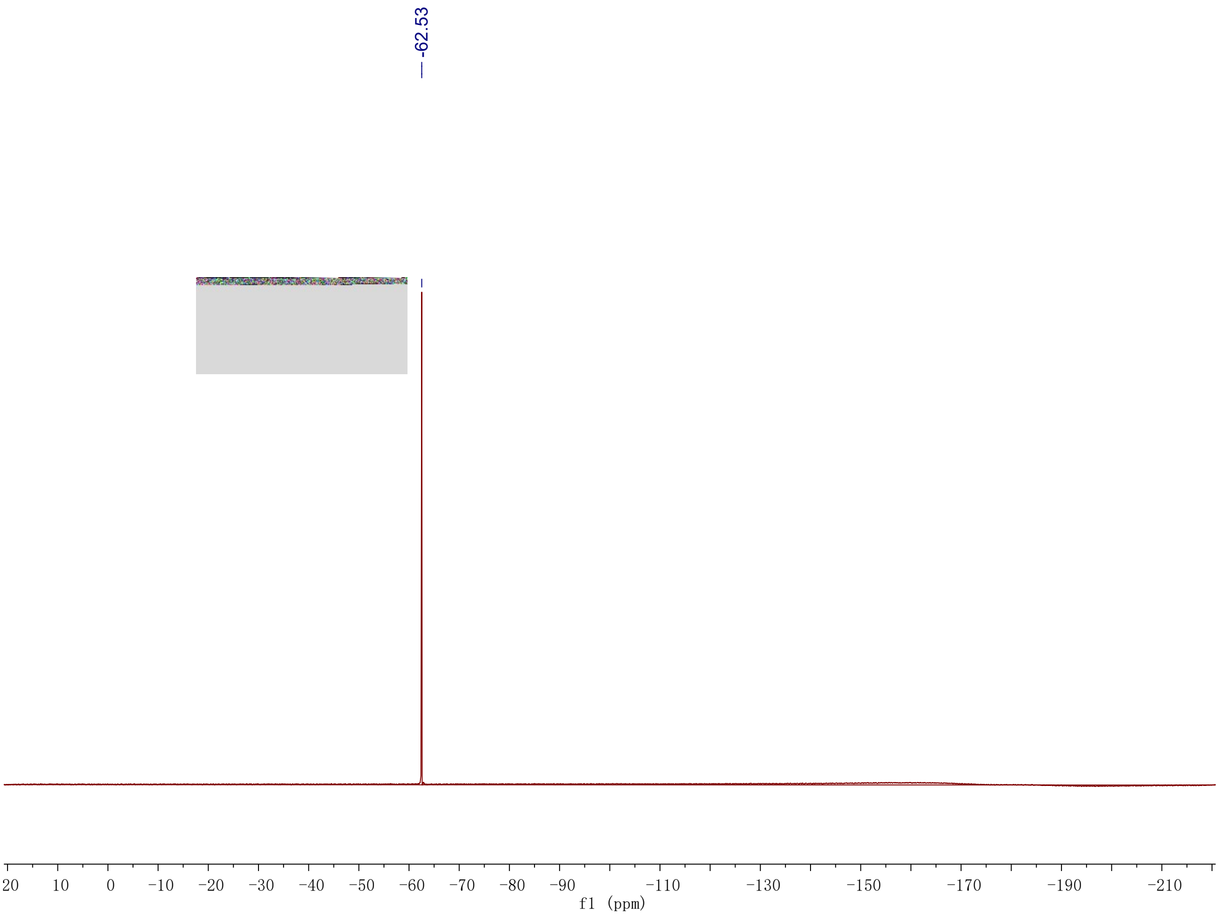
-62.53
20 10 0 −10 −20 −30 −40 −50 −60 −70 −80 −90 −110 −130 −150 −170 −190 −210
f1 (ppm)

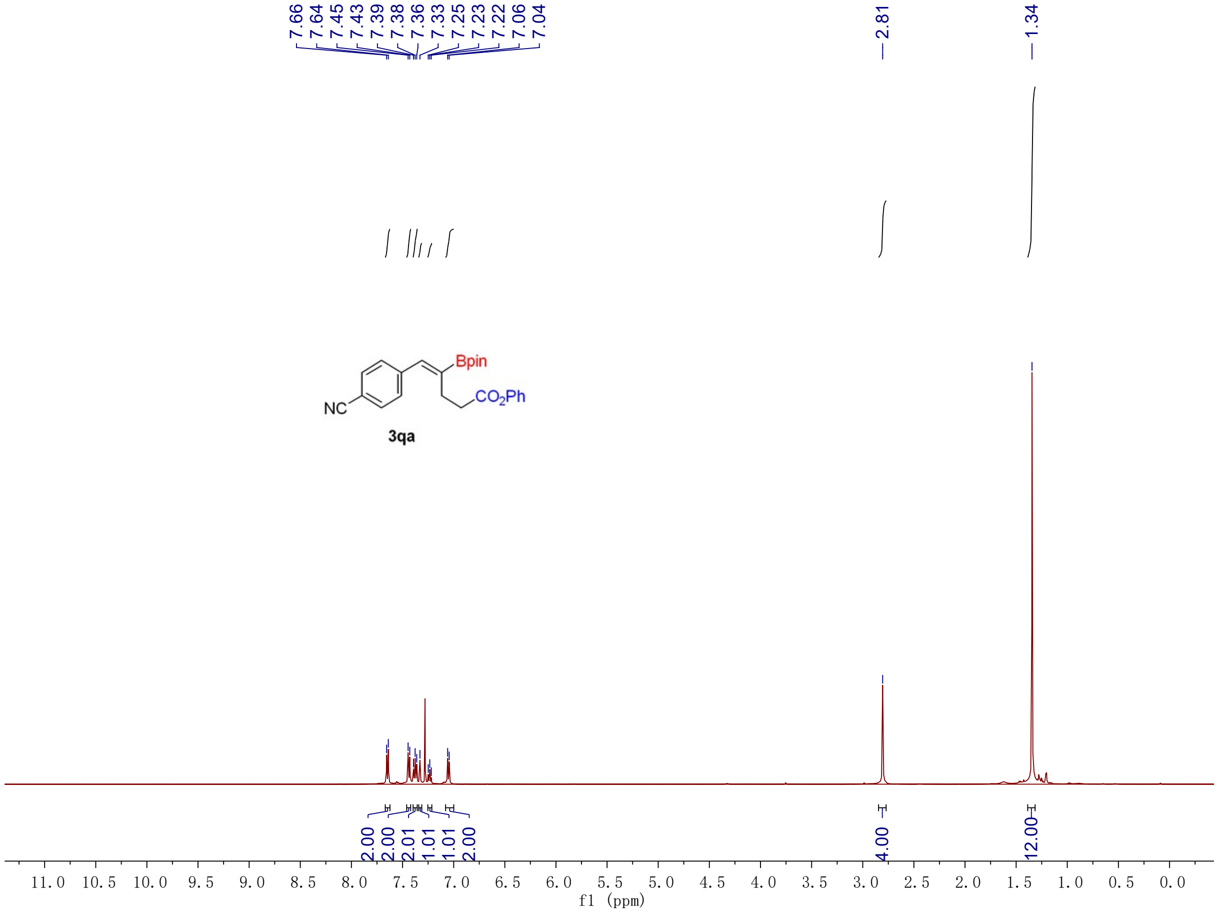
7.66 7.64 7.45 7.43 7.39 7.38 7.36 7.33 7.25 7.23 7.22 7.06 7.04
2.81
1.34
Bpin
NC
CO2Ph
3qa
2.00 2.00 2.01 1.01 1.01 2.00
4.00
12.00
11.0 10.5 10.0 9.5 9.0 8.5 8.0 7.5 7.0 6.5 6.0 5.5 5.0 4.5 4.0 3.5 3.0 2.5 2.0 1.5 1.0 0.5 0.0
f1 (ppm)

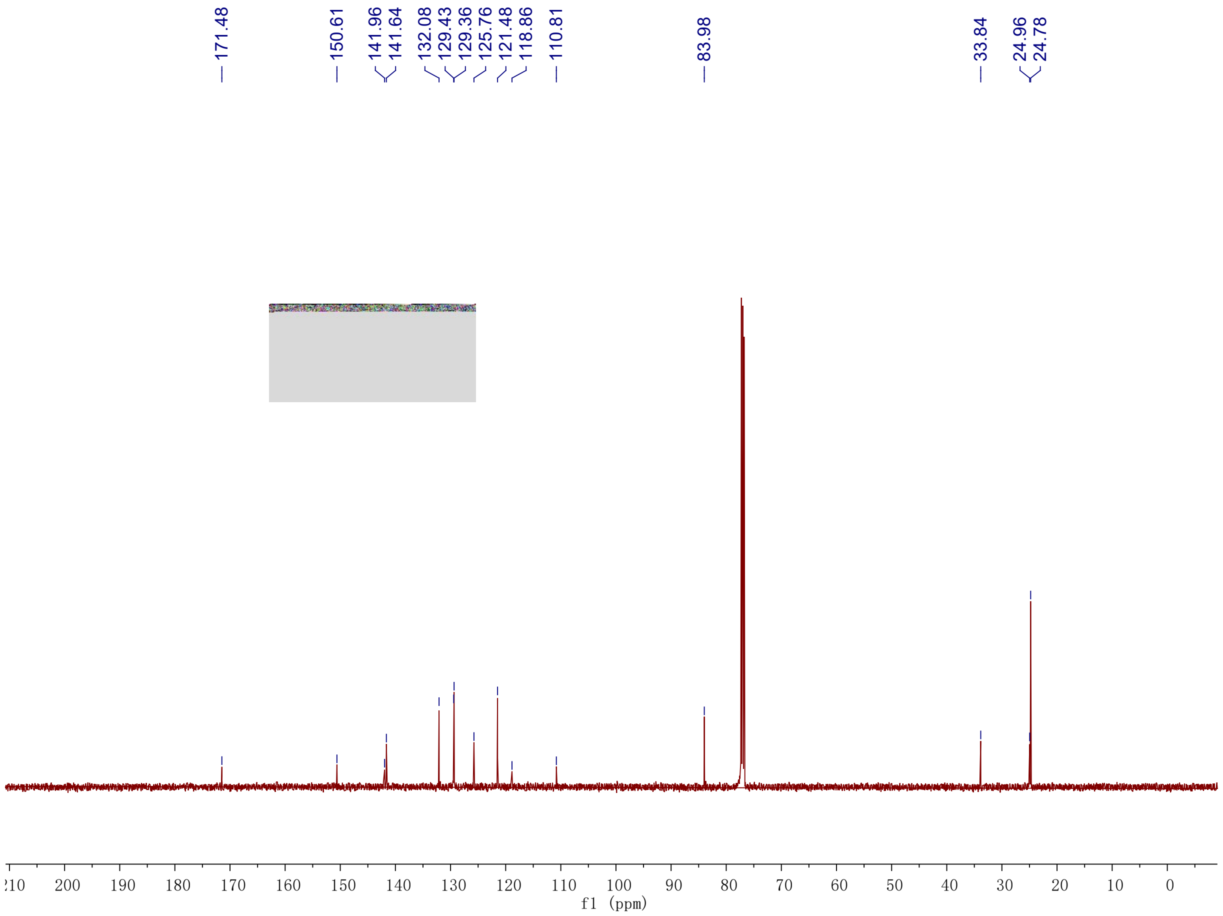
171.48
150.61
141.96
141.64
132.08
129.43
129.36
125.76
121.48
118.86
110.81
83.98
33.84
24.96
24.78
f1 (ppm)

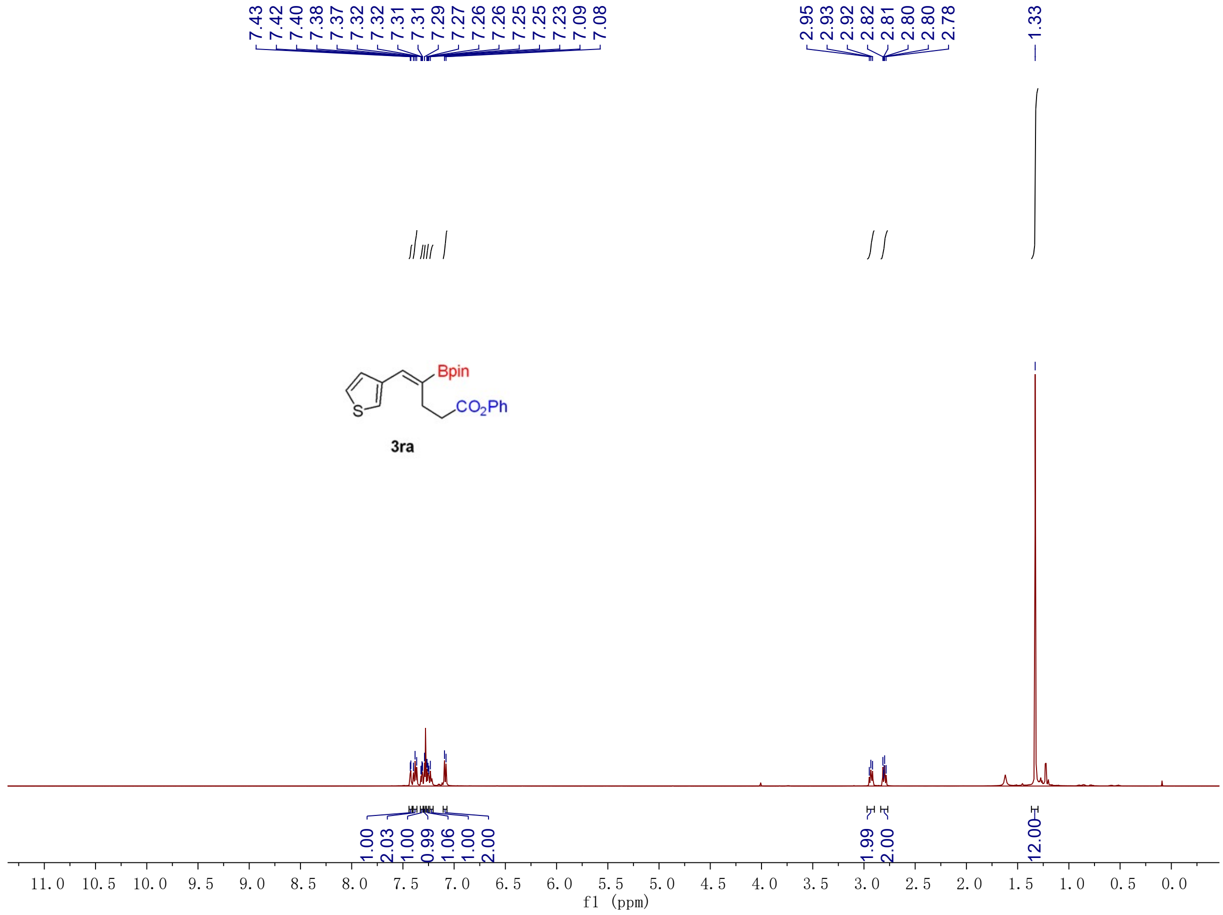
7.43
7.42
7.40
7.38
7.37
7.32
7.31
7.31
7.29
7.27
7.26
7.26
7.25
7.25
7.23
7.09
7.08
2.95
2.93
2.92
2.82
2.81
2.80
2.80
2.78
1.33
1.00
2.03
1.00
0.99
1.06
1.00
2.00
1.99
2.00
12.00
f1 (ppm)
Bpin
S
CO_2Ph

3ra

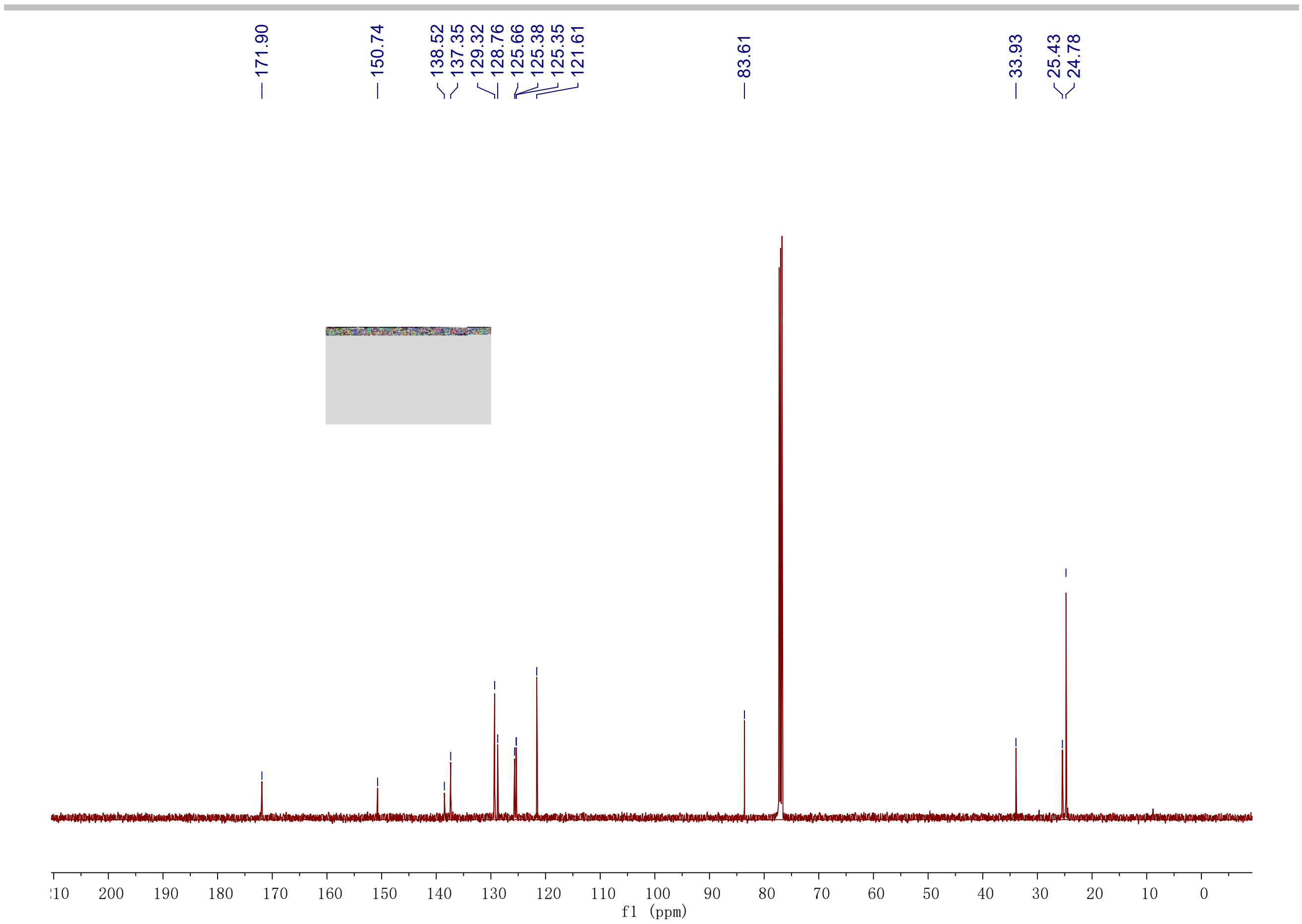
171.90
150.74
138.52
137.35
129.32
128.76
125.66
125.38
125.35
121.61
83.61
33.93
25.43
24.78
f1 (ppm)

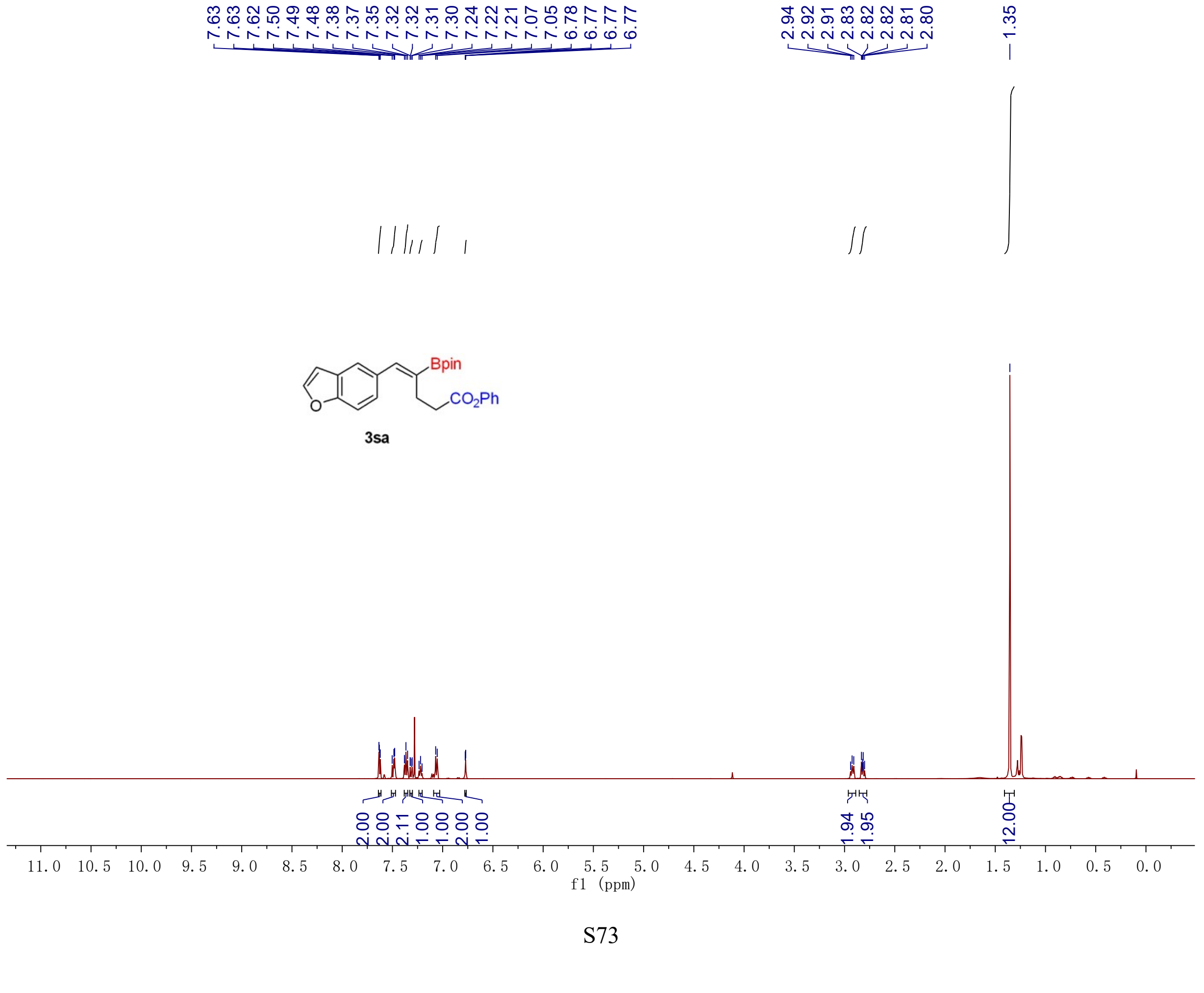
7.63
7.63
7.62
7.50
7.49
7.48
7.38
7.37
7.35
7.32
7.32
7.31
7.30
7.24
7.22
7.21
7.07
7.05
6.78
6.77
6.77
6.77
2.94
2.92
2.91
2.83
2.82
2.82
2.81
2.80
1.35
Bpin
CO_2Ph
3sa
2.00
2.00
2.11
1.00
1.00
2.00
1.00
1.94
1.95
12.00
f1 (ppm)

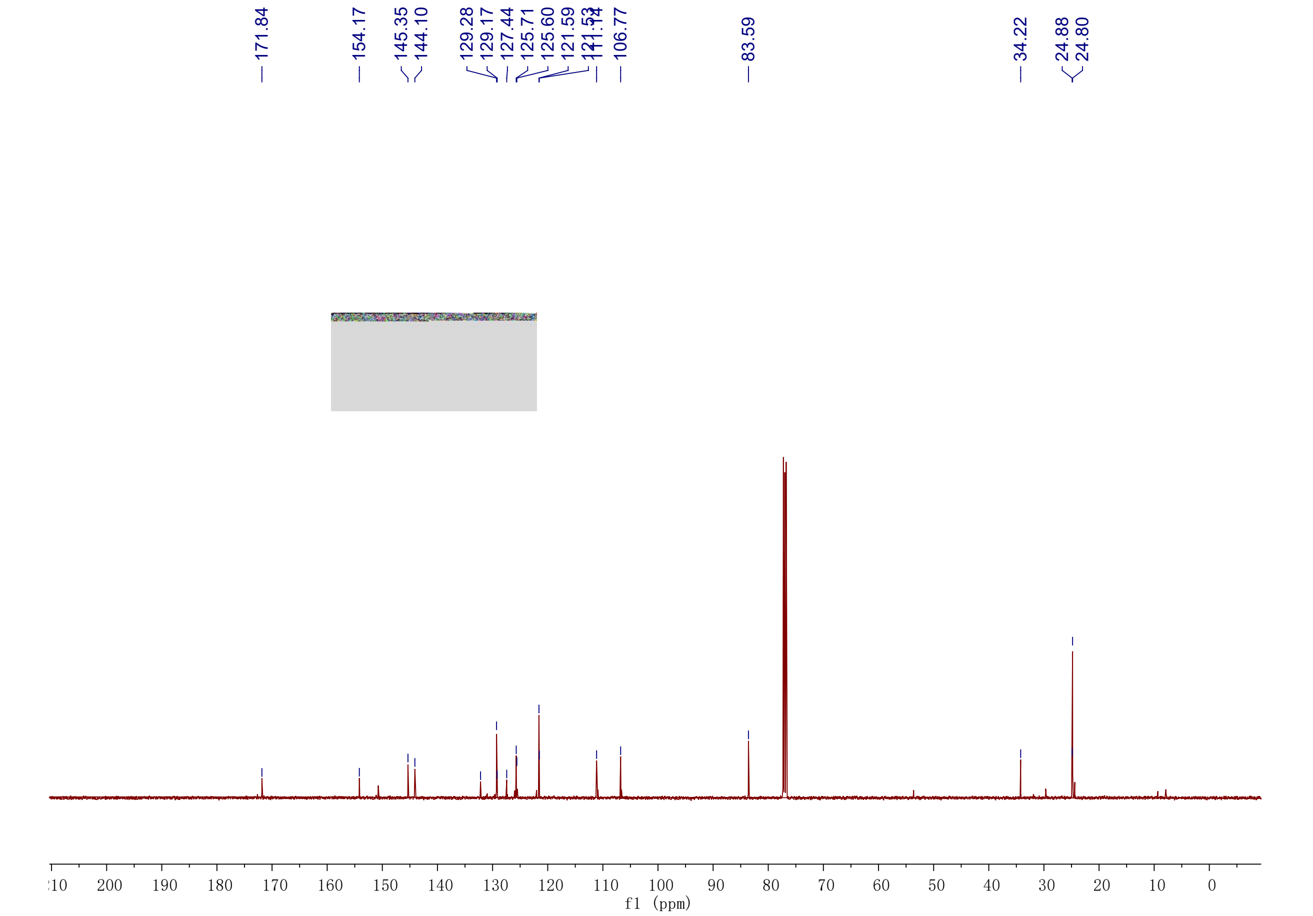
171.84
154.17
145.35
144.10
129.28
129.17
127.44
125.71
125.60
121.59
121.53
111.14
106.77
83.59
34.22
24.88
24.80
f1 (ppm)

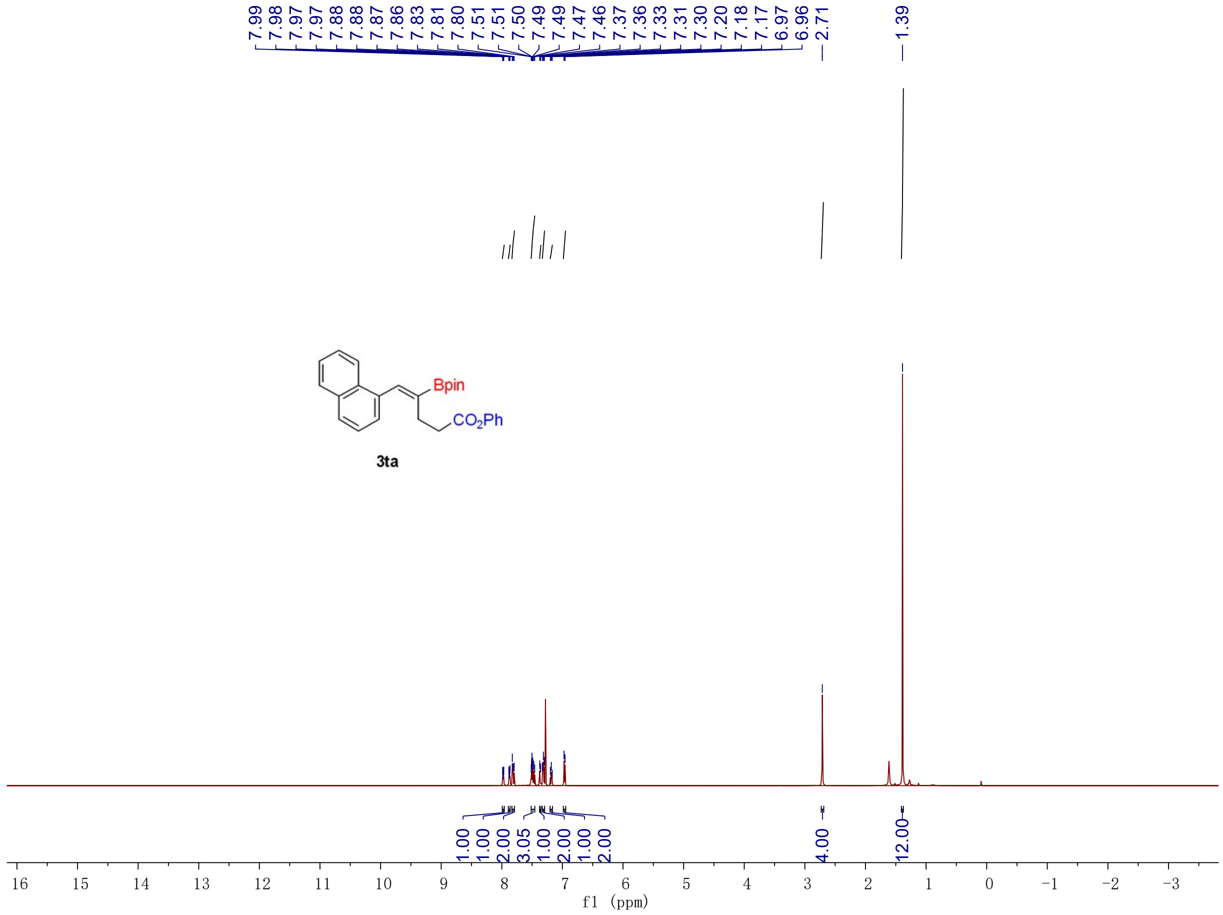
7.99
7.98
7.97
7.97
7.88
7.88
7.87
7.86
7.83
7.81
7.80
7.51
7.50
7.49
7.49
7.47
7.46
7.37
7.36
7.33
7.31
7.30
7.20
7.18
7.17
6.97
6.96
2.71
1.39
Bpin
CO2Ph
3ta
1.00
1.00
2.00
3.05
1.00
2.00
1.00
2.00
4.00
12.00
f1 (ppm)

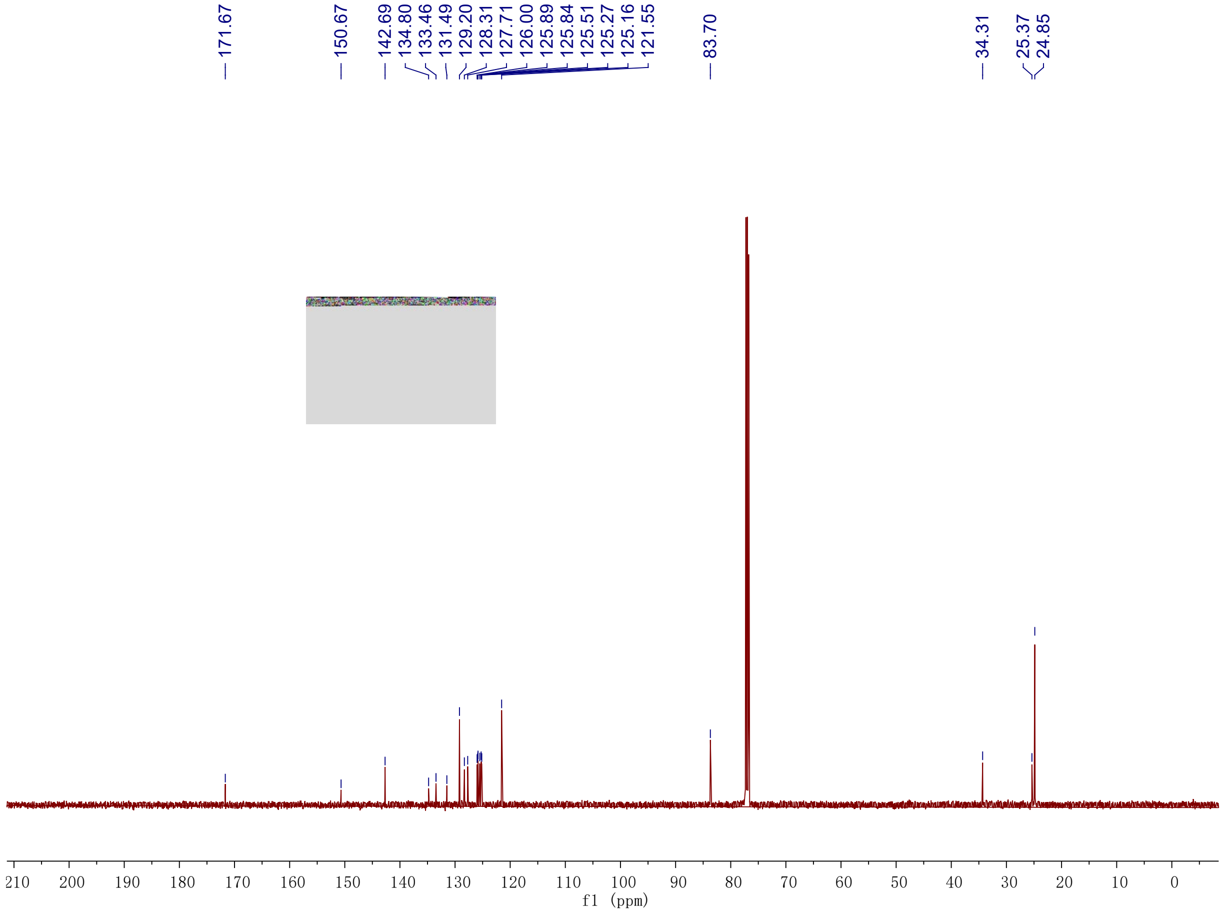

171.67
150.67
142.69
134.80
133.46
131.49
129.20
128.31
127.71
126.00
125.89
125.84
125.51
125.27
125.16
121.55
83.70
34.31
25.37
24.85
210 200 190 180 170 160 150 140 130 120 110 100 90 80 70 60 50 40 30 20 10 0
f1 (ppm)

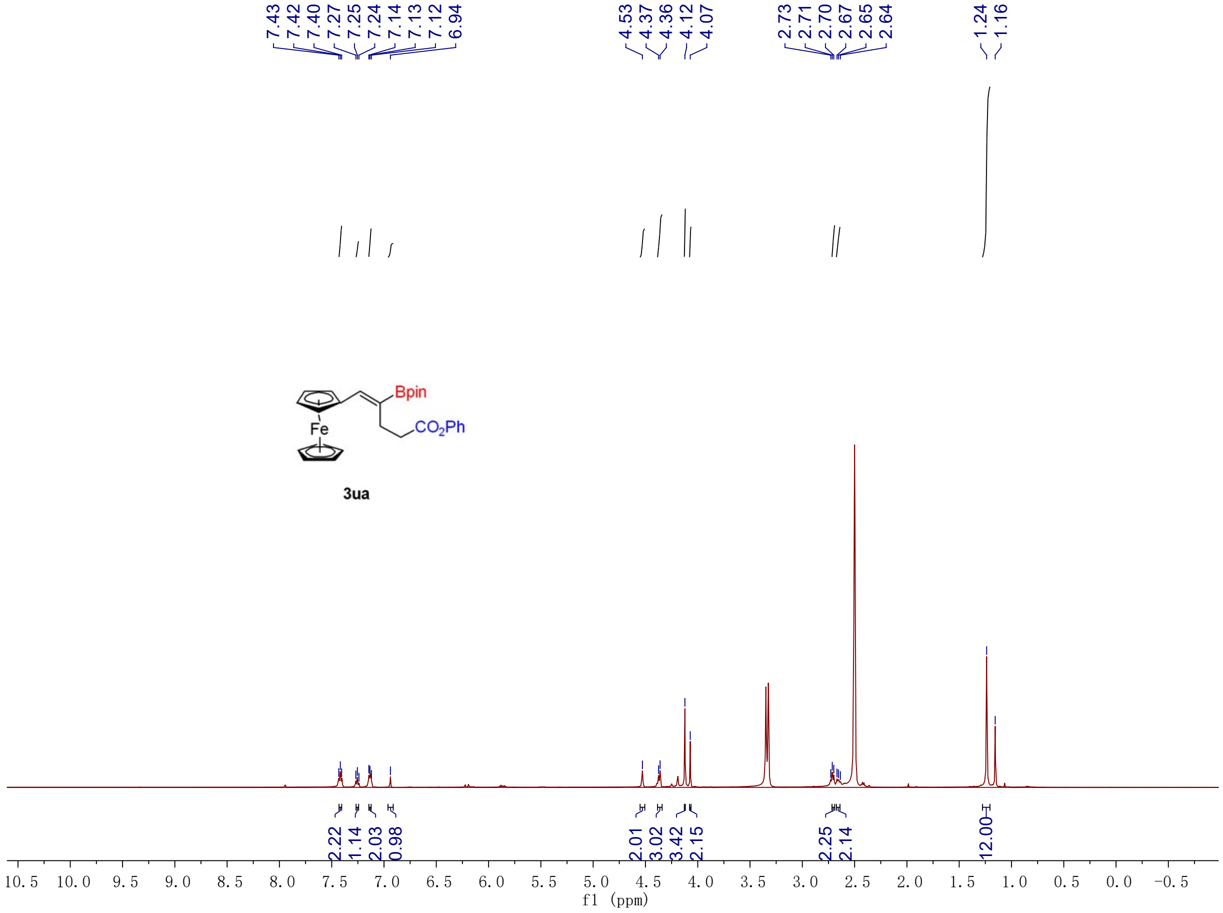

7.43
7.42
7.40
7.27
7.25
7.24
7.14
7.13
7.12
6.94
4.53
4.37
4.36
4.12
4.07
2.73
2.71
2.70
2.67
2.65
2.64
1.24
1.16
Bpin
Fe
CO2Ph
3ua
2.22
1.14
2.03
0.98
2.01
3.02
3.42
2.15
2.25
2.14
12.00
10.5 10.0 9.5 9.0 8.5 8.0 7.5 7.0 6.5 6.0 5.5 5.0 4.5 4.0 3.5 3.0 2.5 2.0 1.5 1.0 0.5 0.0 -0.5
f1 (ppm)

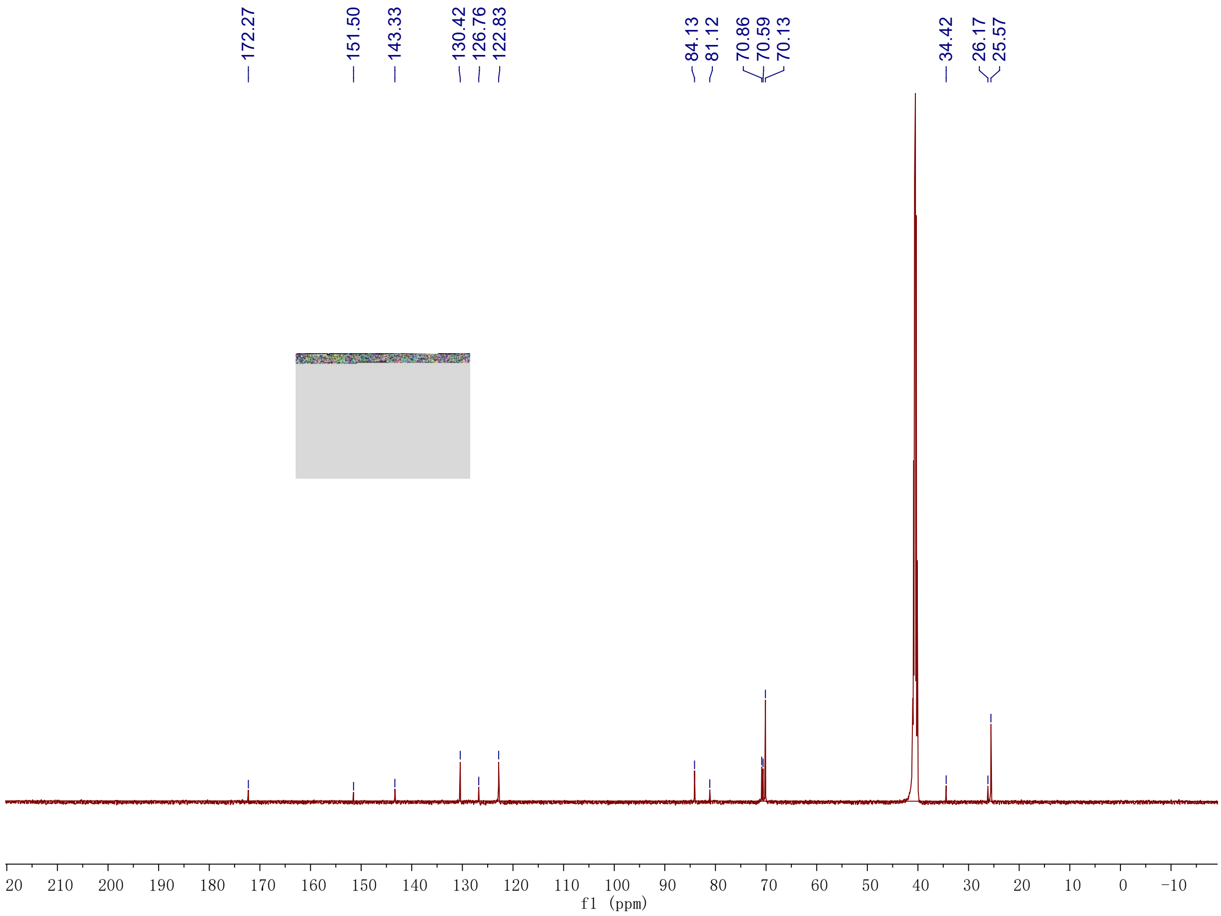
172.27
151.50
143.33
130.42
126.76
122.83
84.13
81.12
70.86
70.59
70.13
34.42
26.17
25.57
f1 (ppm)

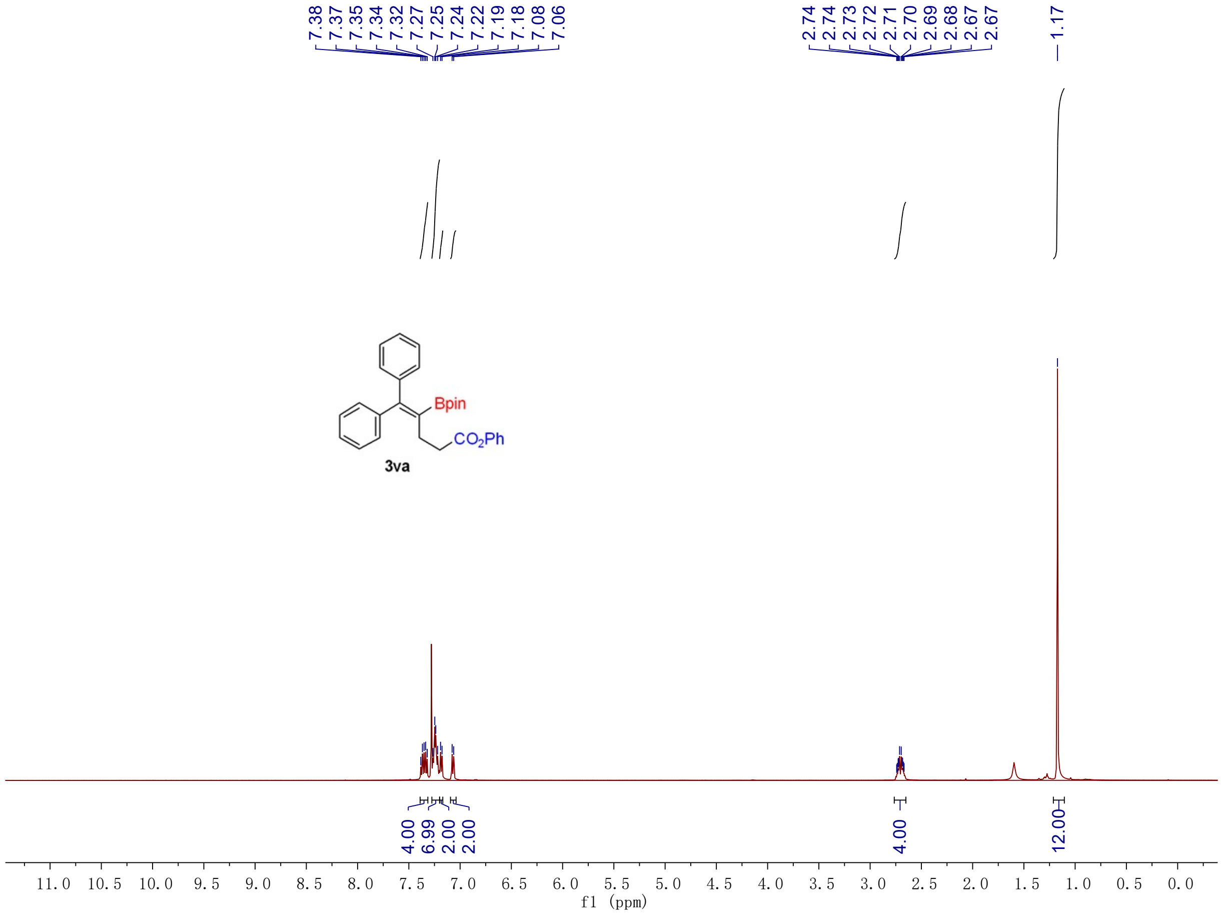
7.38
7.37
7.35
7.34
7.32
7.27
7.25
7.24
7.22
7.19
7.18
7.08
7.06
2.74
2.74
2.73
2.72
2.71
2.70
2.69
2.68
2.67
2.67
1.17
Bpin
CO2Ph
3va
4.00
6.99
2.00
2.00
4.00
12.00
f1 (ppm)

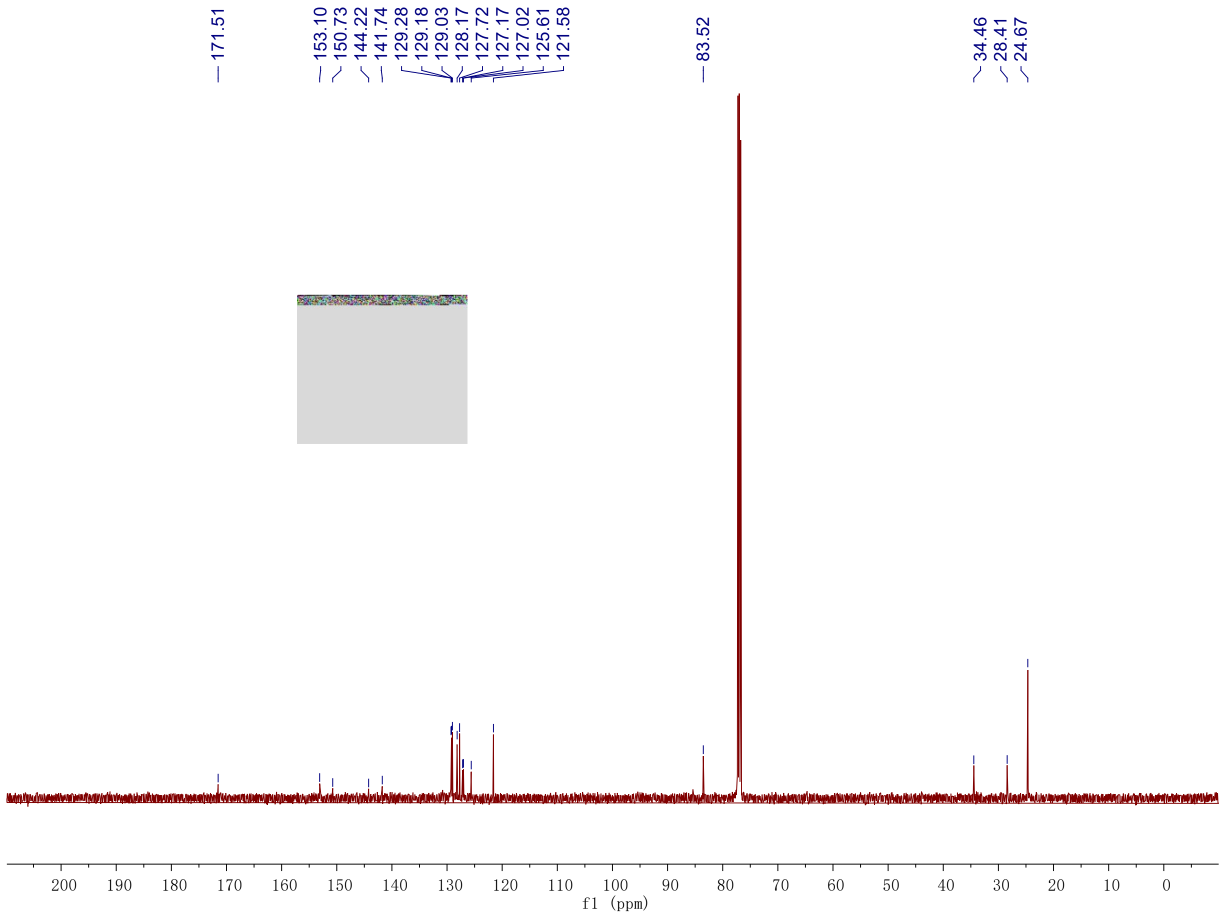
171.51
153.10
150.73
144.22
141.74
129.28
129.18
129.03
128.17
127.72
127.17
127.02
125.61
121.58
83.52
34.46
28.41
24.67
200 190 180 170 160 150 140 130 120 110 100 90 80 70 60 50 40 30 20 10 0
f1 (ppm)

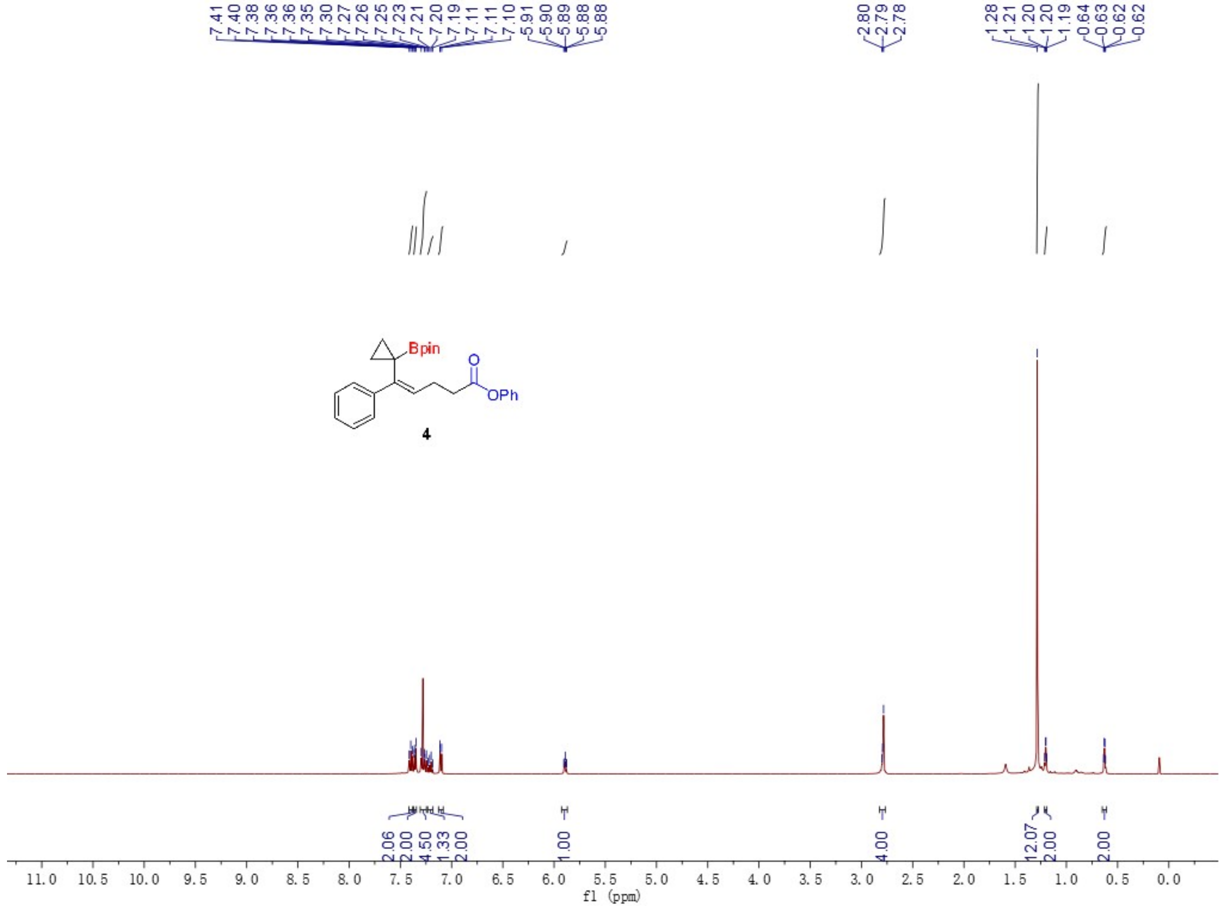
7.41 7.40 7.38 7.36 7.36 7.35 7.30 7.27 7.26 7.25 7.23 7.21 7.20 7.19 7.11 7.10
5.91 5.90 5.89 5.88 5.88
2.80 2.79 2.78
1.28 1.21 1.20 1.20 1.19
0.64 0.63 0.62 0.62
Bpin
O
OPh
4
2.06 2.00 4.50 1.33 2.00
1.00
4.00
12.07 2.00
2.00
11.0 10.5 10.0 9.5 9.0 8.5 8.0 7.5 7.0 6.5 6.0 5.5 5.0 4.5 4.0 3.5 3.0 2.5 2.0 1.5 1.0 0.5 0.0
f1 (ppm)

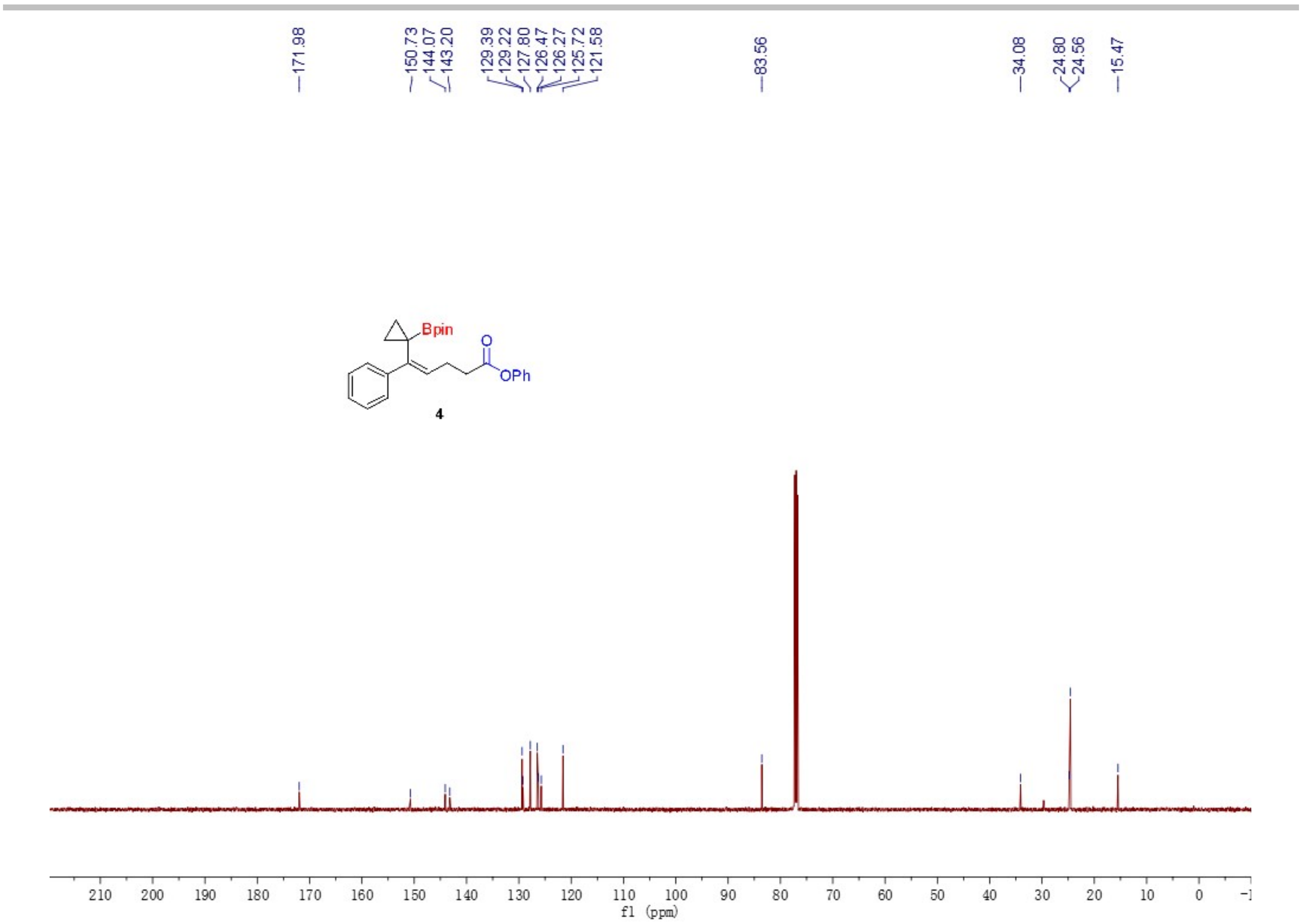
171.98
150.73
144.07
143.20
129.39
129.22
127.80
126.47
126.27
125.72
121.58
83.56
34.08
24.80
24.56
15.47
Bpin
O
OPh
4
210 200 190 180 170 160 150 140 130 120 110 100 90 80 70 60 50 40 30 20 10 0
f1 (ppm)

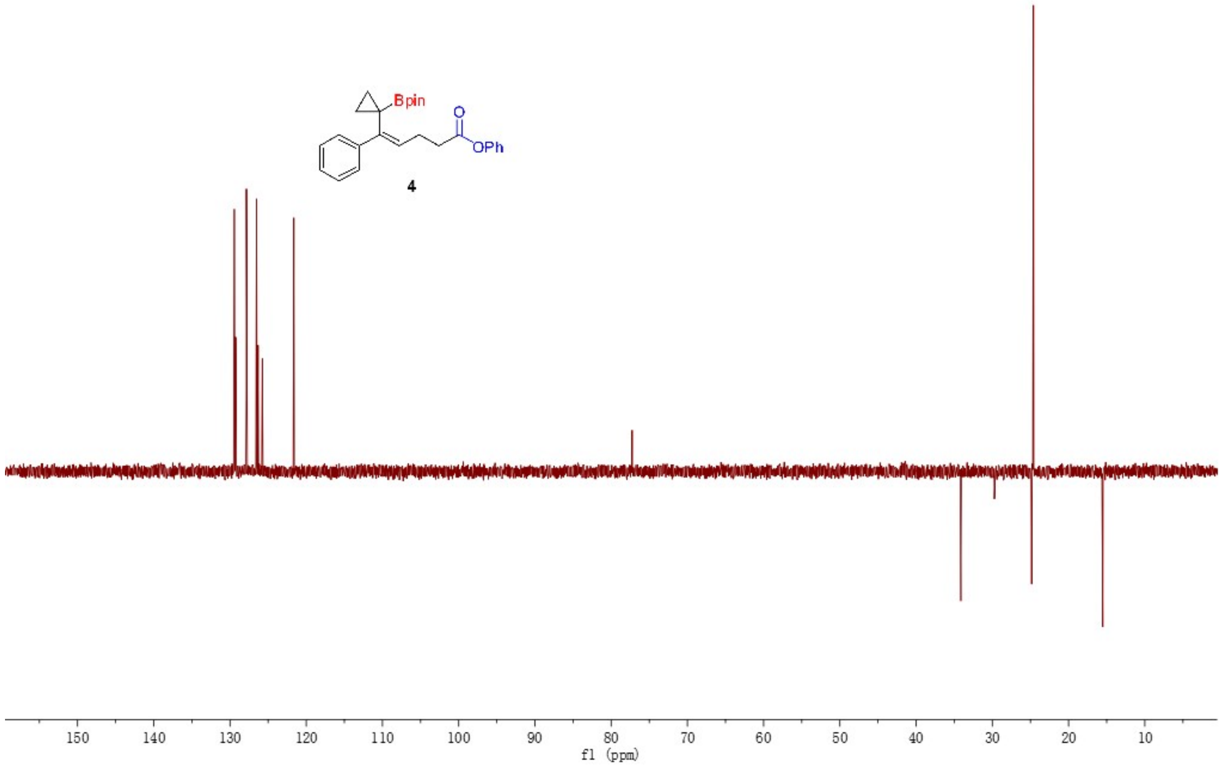
Bpin
O
OPh
4
150 140 130 120 110 100 90 80 70 60 50 40 30 20 10
f1 (ppm)

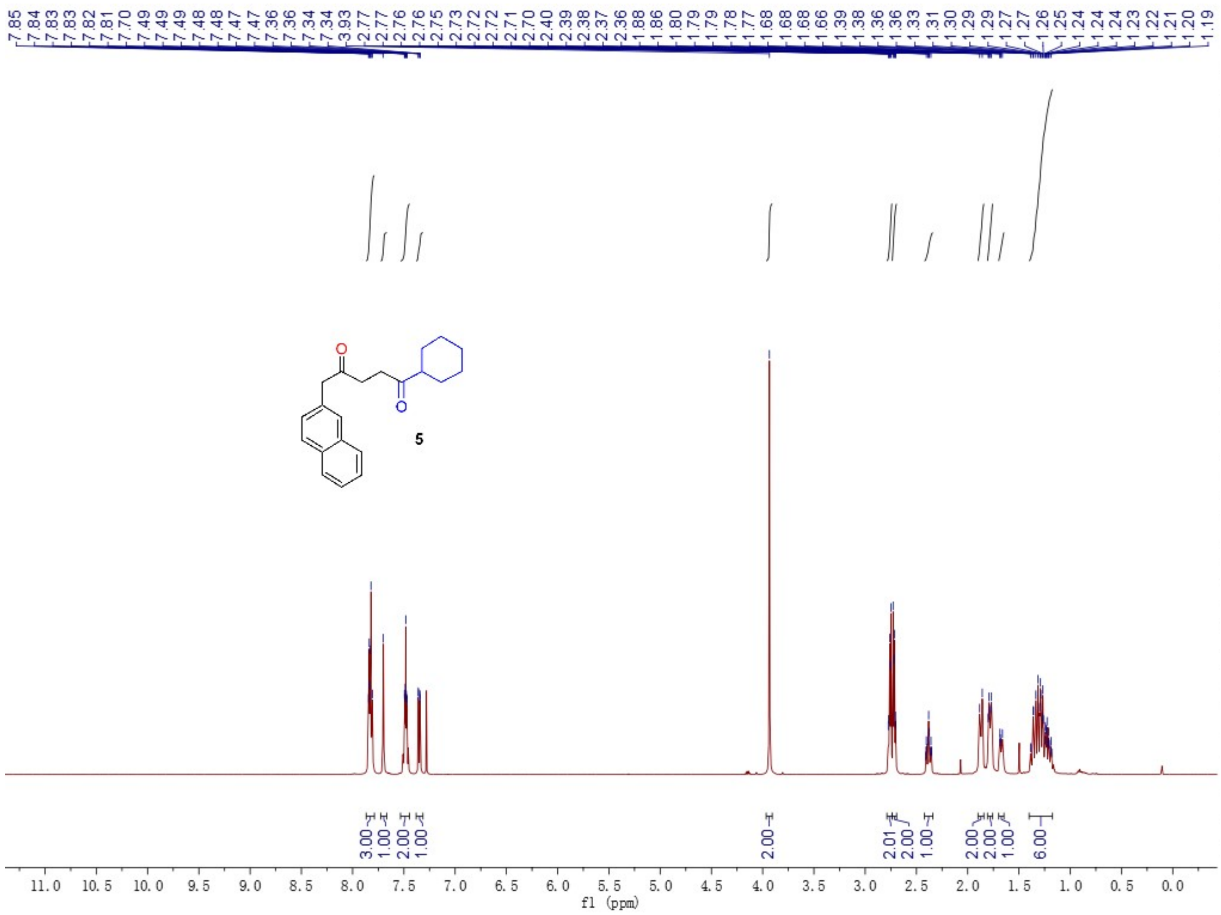
7.85 7.84 7.83 7.83 7.82 7.81 7.70 7.49 7.49 7.49 7.48 7.48 7.47 7.47 7.36 7.36 7.34 7.34 3.93 2.77 2.77 2.76 2.76 2.75 2.73 2.73 2.72 2.72 2.71 2.70 2.40 2.39 2.38 2.37 2.36 1.88 1.86 1.80 1.79 1.79 1.78 1.77 1.68 1.68 1.68 1.66 1.39 1.38 1.36 1.36 1.33 1.31 1.30 1.29 1.29 1.27 1.27 1.26 1.25 1.24 1.24 1.24 1.23 1.22 1.21 1.20 1.19
O
O
5
3.00 1.00 2.00 1.00
2.00
2.01 2.00 1.00
2.00 2.00 1.00
6.00
11.0 10.5 10.0 9.5 9.0 8.5 8.0 7.5 7.0 6.5 6.0 5.5 5.0 4.5 4.0 3.5 3.0 2.5 2.0 1.5 1.0 0.5 0.0
f1 (ppm)

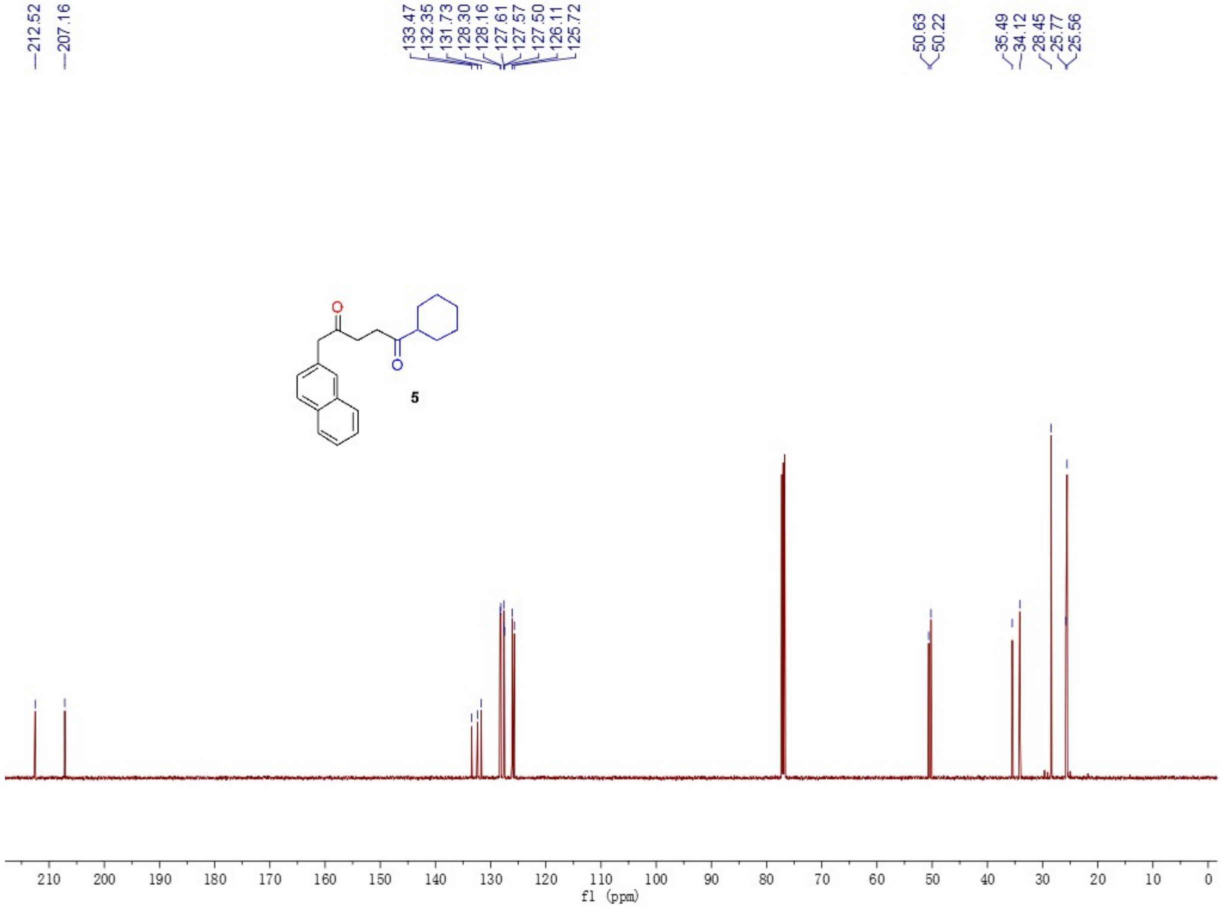
212.52 207.16
133.47 132.35 131.73 128.30 128.16 127.61 127.57 127.50 126.11 125.72
50.63 50.22
35.49 34.12 28.45 25.77 25.56
O
O
5
210 200 190 180 170 160 150 140 130 120 110 100 90 80 70 60 50 40 30 20 10 0
f1 (ppm)

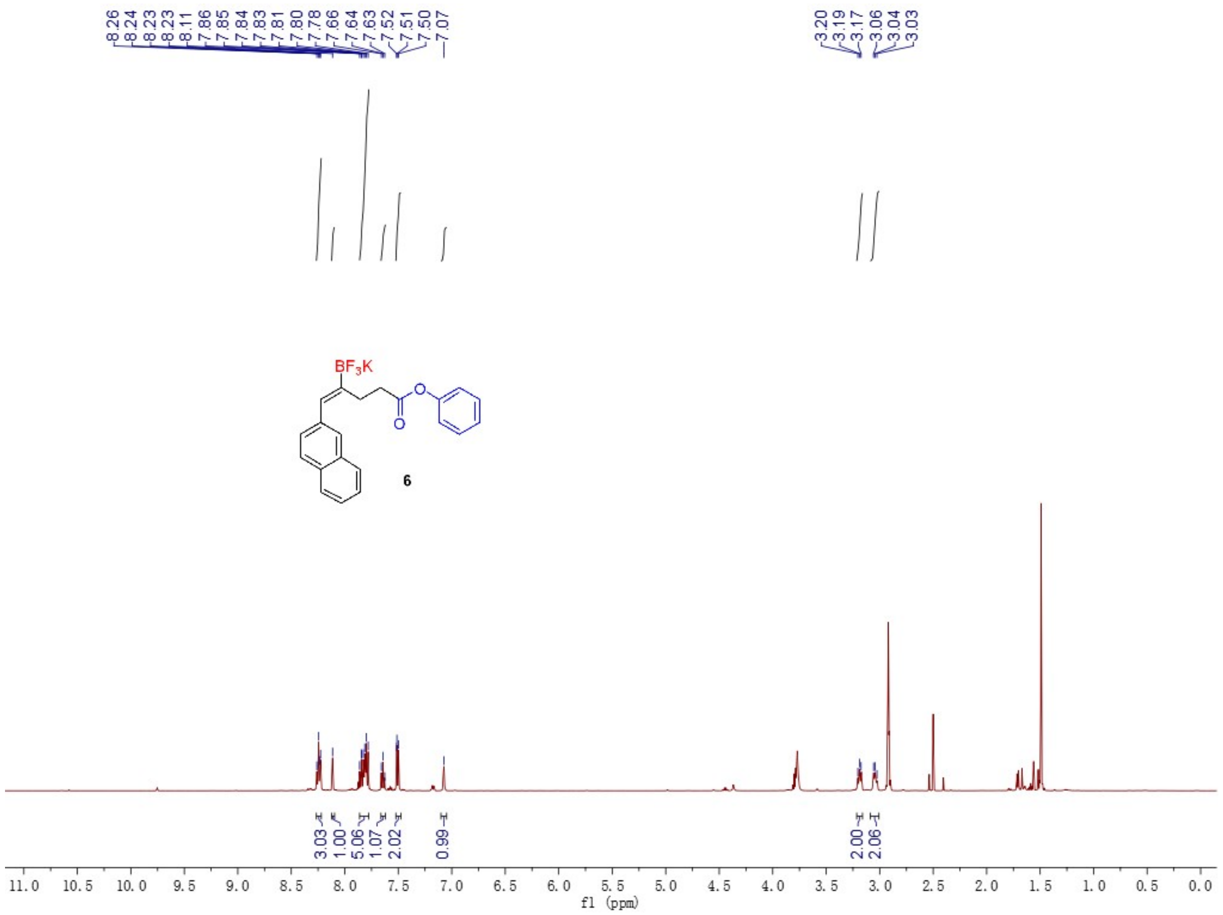
8.26
8.24
8.23
8.23
8.11
7.86
7.85
7.84
7.83
7.81
7.80
7.78
7.66
7.64
7.63
7.52
7.51
7.50
7.07
3.20
3.19
3.17
3.06
3.04
3.03
BF3K
O
O
6
3.03
1.00
5.06
1.07
2.02
0.99
2.00
2.06
11.0 10.5 10.0 9.5 9.0 8.5 8.0 7.5 7.0 6.5 6.0 5.5 5.0 4.5 4.0 3.5 3.0 2.5 2.0 1.5 1.0 0.5 0.0
f1 (ppm)

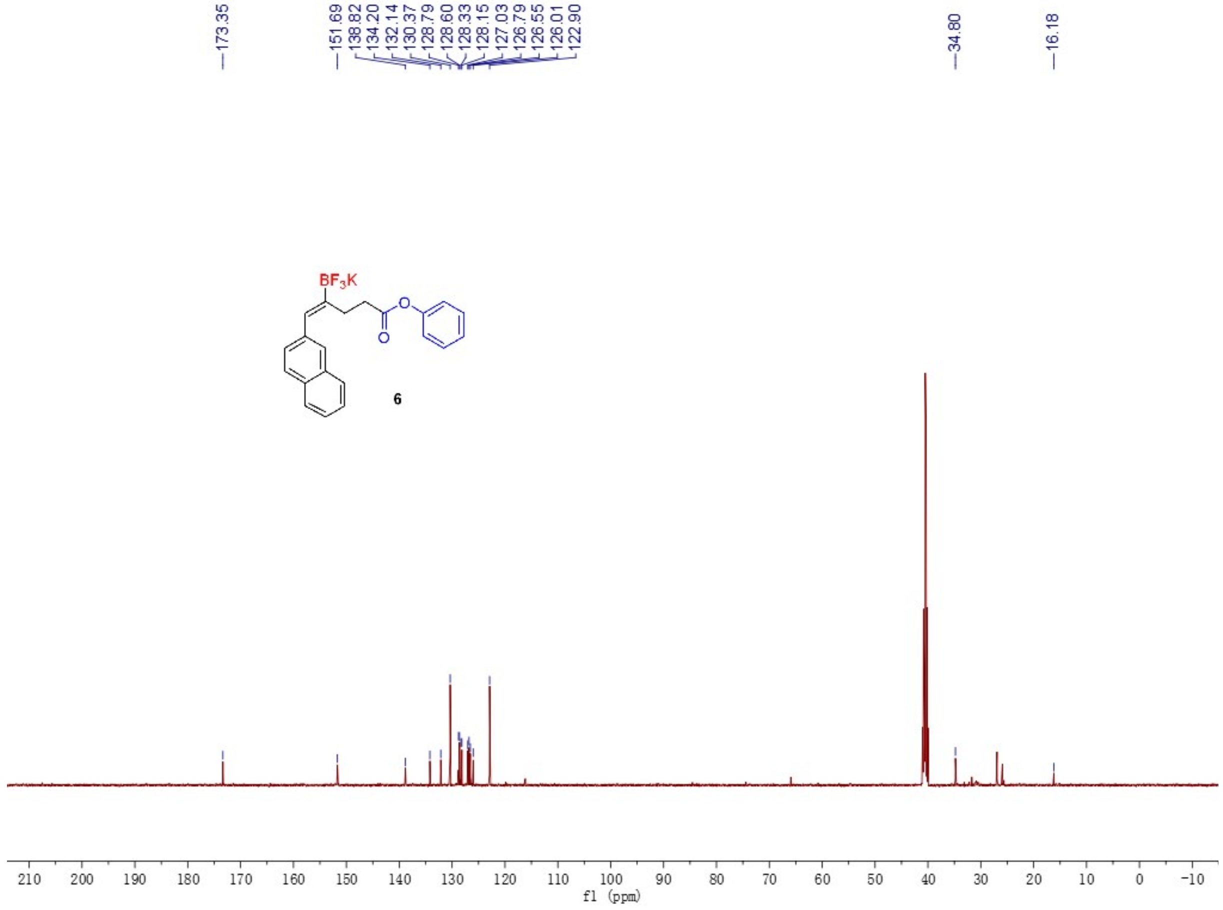
173.35
151.69
138.82
134.20
132.14
130.37
128.79
128.60
128.33
128.15
127.03
126.79
126.55
126.01
122.90
34.80
16.18
BF3K
O
O
6
210 200 190 180 170 160 150 140 130 120 110 100 90 80 70 60 50 40 30 20 10 0 -10
f1 (ppm)

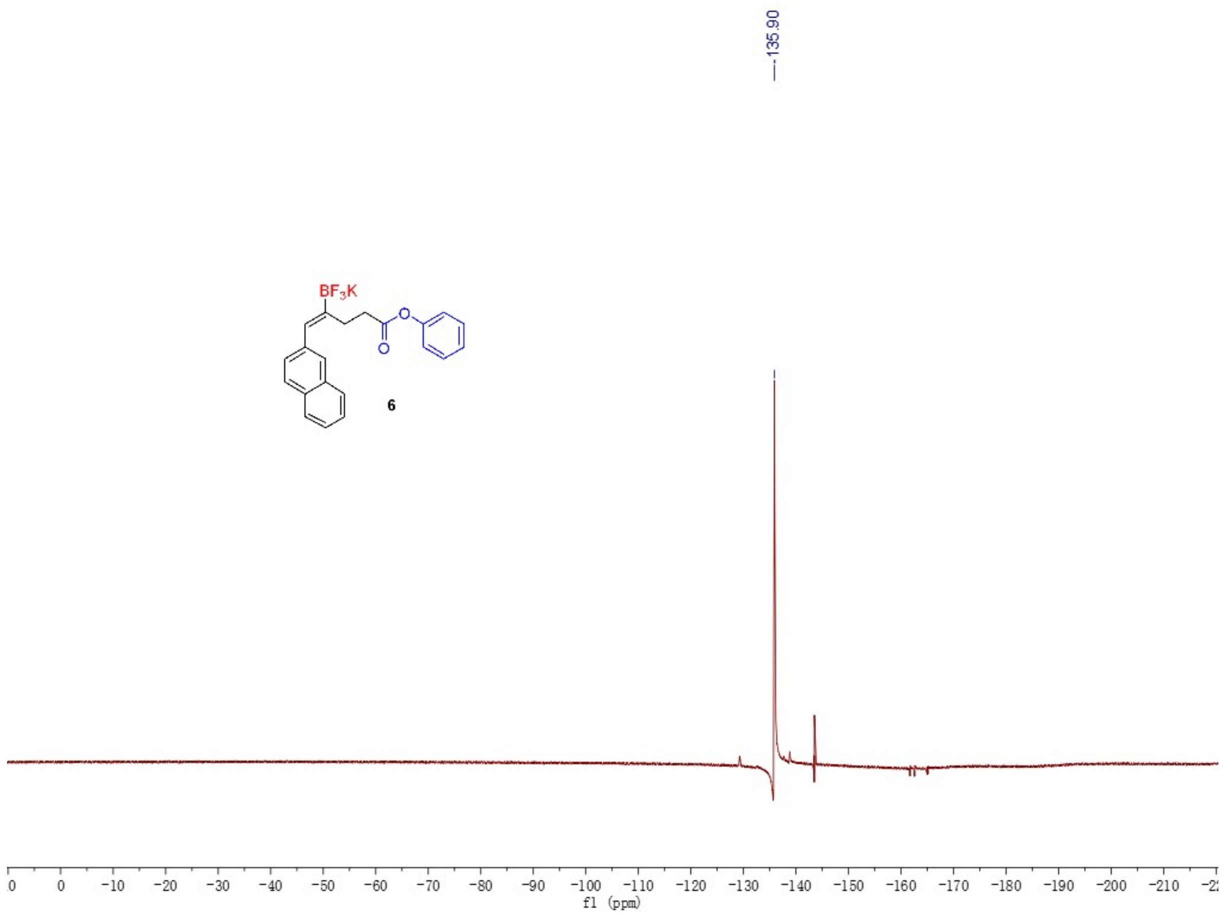

-135.90
BF3K
6
f1 (ppm)

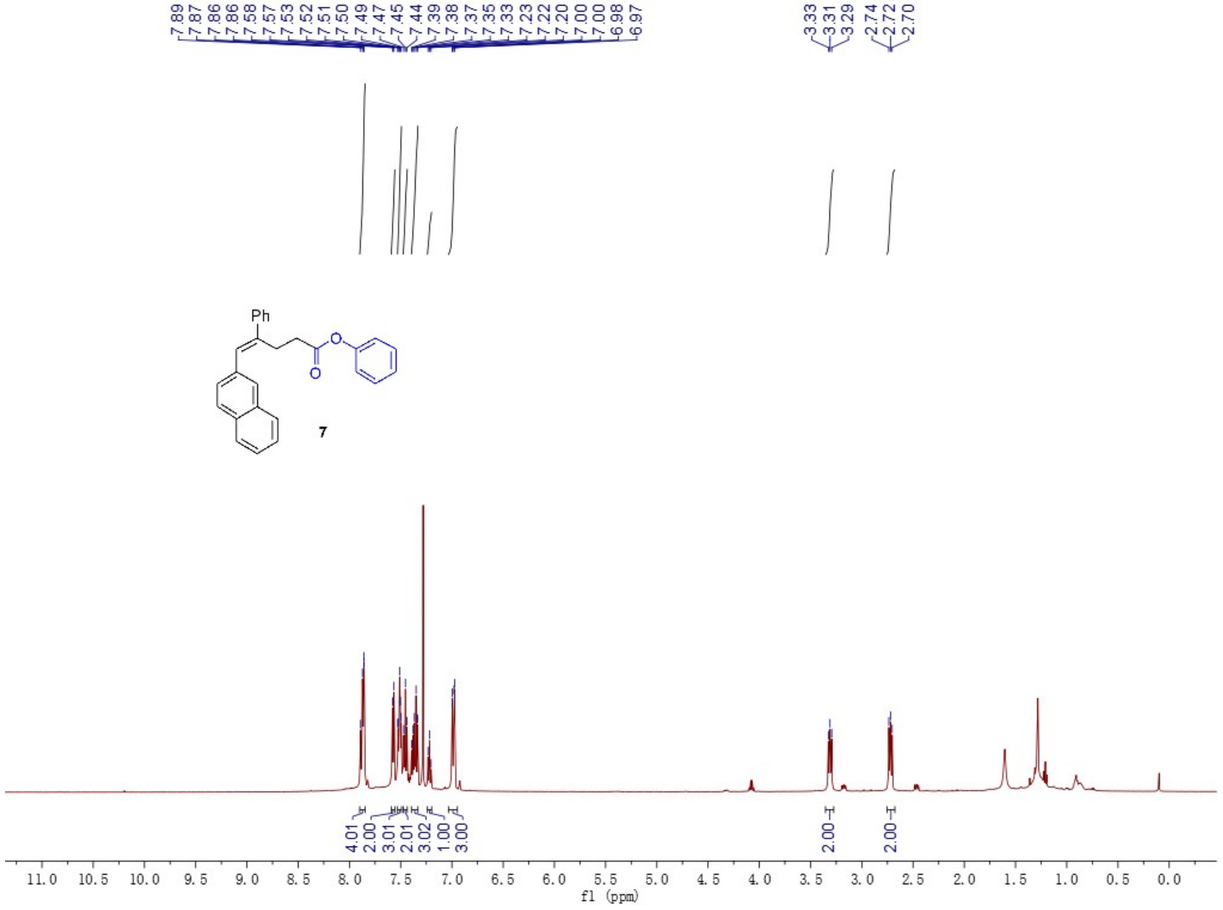

7.89 7.87 7.86 7.86 7.58 7.57 7.53 7.52 7.51 7.50 7.49 7.47 7.45 7.44 7.39 7.38 7.37 7.35 7.33 7.23 7.22 7.22 7.20 7.00 7.00 6.98 6.97
3.33 3.31 3.29 2.74 2.72 2.70
Ph
7
4.01 2.00 3.01 2.01 3.02 1.00 3.00
2.00
2.00
f1 (ppm)

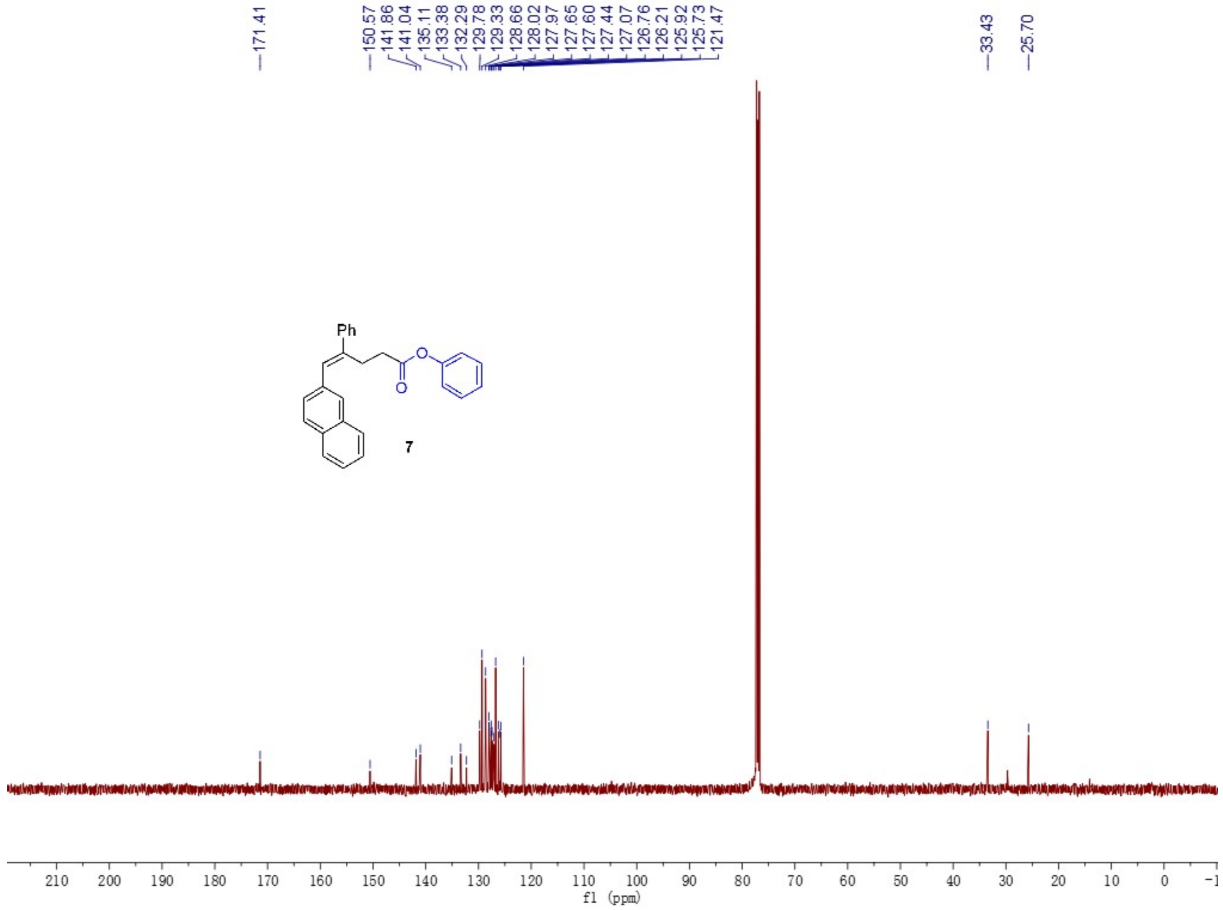
171.41
150.57
141.86
141.04
135.11
133.38
132.29
129.78
129.33
128.66
128.02
127.97
127.65
127.60
127.44
127.07
126.76
126.21
125.92
125.73
121.47
33.43
25.70
Ph
O
O
7
210 200 190 180 170 160 150 140 130 120 110 100 90 80 70 60 50 40 30 20 10 0
f1 (ppm)
